# Chlamydiae Has Contributed at Least 55 Genes to Plantae with Predominantly Plastid Functions

**DOI:** 10.1371/journal.pone.0002205

**Published:** 2008-05-21

**Authors:** Ahmed Moustafa, Adrian Reyes-Prieto, Debashish Bhattacharya

**Affiliations:** 1 Interdisciplinary Program in Genetics, University of Iowa, Iowa City, Iowa, United States of America; 2 Department of Biological Sciences and Roy J. Carver Center for Comparative Genomics, University of Iowa, Iowa City, Iowa, United States of America; American Museum of Natural History, United States of America

## Abstract

**Background:**

The photosynthetic organelle (plastid) originated via primary endosymbiosis in which a phagotrophic protist captured and harnessed a cyanobacterium. The plastid was inherited by the common ancestor of the red, green (including land plants), and glaucophyte algae (together, the Plantae). Despite the critical importance of primary plastid endosymbiosis, its ancient derivation has left behind very few “footprints” of early key events in organelle genesis.

**Methodology/Principal Findings:**

To gain insights into this process, we conducted an in-depth phylogenomic analysis of genomic data (nuclear proteins) from 17 Plantae species to identify genes of a surprising provenance in these taxa, Chlamydiae bacteria. Previous studies show that Chlamydiae contributed many genes (at least 21 in one study) to Plantae that primarily have plastid functions and were postulated to have played a fundamental role in organelle evolution. Using our comprehensive approach, we identify at least 55 Chlamydiae-derived genes in algae and plants, of which 67% (37/55) are putatively plastid targeted and at least 3 have mitochondrial functions. The remainder of the proteins does not contain a bioinformatically predicted organelle import signal although one has an N-terminal extension in comparison to the Chlamydiae homolog. Our data suggest that environmental Chlamydiae were significant contributors to early Plantae genomes that extend beyond plastid metabolism. The chlamydial gene distribution and protein tree topologies provide evidence for both endosymbiotic gene transfer and a horizontal gene transfer ratchet driven by recurrent endoparasitism as explanations for gene origin.

**Conclusions/Significance:**

Our findings paint a more complex picture of gene origin than can easily be explained by endosymbiotic gene transfer from an organelle-like point source. These data significantly extend the genomic impact of Chlamydiae on Plantae and show that about one-half (30/55) of the transferred genes are most closely related to sequences emanating from the genome of the only environmental isolate that is currently available. This strain, *Candidatus Protochlamydia amoebophila* UWE25 is an endosymbiont of *Acanthamoeba* and likely represents the type of endoparasite that contributed the genes to Plantae.

## Introduction

The origin of the photosynthetic organelle (plastid) in eukaryotes occurred via the capture and enslavement of a cyanobacterium (primary endosymbiosis) [1], [2]. This “primary” plastid is shared by the red, green (including land plants), and glaucophyte algae (together the Plantae) [3], [4]. Under the most parsimonious scenario, the Plantae share a unique common branch that defines the point of entry of the primary endosymbiont [1], [5], [6], [7], although the monophyly of this group remains to be unambiguously demonstrated using phylogenetic analysis of nuclear genes [8], [9]. The descendants of the first algae came to dominate many aquatic environments and ultimately gave rise to land plants [10]. Plastid characters shared by the Plantae include a complex protein import system (TIC-TOC translocons) and a similar genome architecture and gene content [2], [5], [11], [12], [13], [14].

Here we use phylogenomics to search for genes contributed to Plantae by a surprising source, Chlamydiae bacteria. These prokaryotes are well known as obligate intracellular vertebrate pathogens and encode a unique gene, the ADP/ATP translocase to parasitize energy from the host. This gene is shared by Chlamydiae, Rickettsiales, microsporidians, and photosynthetic eukaryotes. Phylogenetic analysis has demonstrated a chlamydial origin of the plastid-targeted ADP/ATP translocator in algae and plants (e.g., [15], [16], [17]). Interest in the Chlamydiae-plant connection was originally raised by the finding of an affinity for several genes in the sequenced genomes of *Chlamydia trachomatis* and UWE25 (i.e., *Candidatus Protochlamydia amoebophila*) to plant homologs [18], [19]. Later analyses showed that many of these proteins (not just the ADP/ATP translocator) are plastid-targeted in plants [15]. These findings led to a number of different hypotheses to explain chlamydial gene origin in photosynthetic eukaryotes including an ancient, unappreciated relationship between Chlamydiae and the cyanobacterial donor of the plastid [15], the possibility that infected insects may have been the vectors for introducing Chlamydiae genes into plants [20], and ancient horizontal gene transfer from Chlamydiae to the Plantae ancestor that may have played a role in plastid establishment (e.g., [17], [21]).

The most complete analysis to date of the Chlamydiae-Plantae connection is a phylogenomic study that, as reported, found at least 21 genes of chlamydial origin among the 4,771 predicted proteins in the genome of the extremophilic red alga *Cyanidioschyzon merolae* [22]. Virtually all of these Chlamydiae genes encode a plastid targeting signal, are involved in different plastid associated tasks such as fatty acid biosynthesis and ion transport, and are therefore postulated to have played a key role in the establishment of essential plastid functions [22]. Given the large number chlamydial genes that were uncovered, Huang and Gogarten [22] postulated an ancient symbiosis between an environmental chlamydial cell and the Plantae ancestor to account for gene origin rather than repeated horizontal gene transfers (HGTs) from different Chlamydiae. Under their view, the chlamydial endosymbiosis could have been a mutualistic, parasitic, or a commensal relationship but was long-term and stable in the Plantae host. This three-way partnership between the host, the cyanobacterial plastid ancestor, and an environmental Chlamydia-like cell was thought to have played a fundamental role in plastid evolution [22].

Here we reexamine the evolutionary relationship between Chlamydiae and Plantae genes using a phylogenomic approach that incorporates predicted proteins from 17 Plantae genomes to query >500 eukaryotic and prokaryotic genomes in a local database. We use our recently developed automated tree-sorting tool PhyloSort [23] to identify candidate trees (genes). Unlike the previous study [22] however, about two-thirds of the Chlamydiae genes we found are clearly of plastid function, whereas the remainder serve a diversity of potential functions including three that encode a putative mitochondrial targeting sequence. These data provide strong evidence for a long-term symbiotic association *vis a vis* Huang and Gogarten [22] of *Chlamydia*-like cells with the Plantae ancestor that extends beyond plastid establishment. The association may have been one of recurrent infections by one or more specific endoparasite(s) of the Plantae host, as occurs in modern-day environmental Chlamydiae and their eukaryotic hosts [16]. Another possibility (e.g., [22]) is an endosymbiotic, organelle-like association. Although it is currently unknown which (or both) of these explanations may be correct, a ratchet (e.g., [24], [25]) driven by horizontal gene transfer (HGT) from the parasite(s) using a type IV secretion system could readily explain the movement of many Chlamydiae genes into the host nucleus. In either case, the cyanobacterium provided the critical function (photosynthesis) and was retained as a compartment, whereas the Chlamydiae provided key genes through endosymbiotic gene transfer (EGT) and/or HGT. These latter cells were however eliminated by the host, due perhaps to costs they placed on host cell fitness (i.e., as a result of energy-parasitism, [17], [26]).

## Results

Our analyses identified at least 55 Plantae proteins (52 trees at ≥75% and 3 trees at ≥50% RAxML [27] bootstrap support) that are putatively derived from a *Chlamydia*-like source (Table 1). Of these, 37/55 (67%) encoded a putative plastid targeting sequence. The remainder of the proteins were putatively of non-plastid function (e.g., involved in protein translation; see Table 1) based on organelle targeting predictions using TargetP [28], Predotar [29], ChloroP [30], PSLDoc [31], and WoLF PSORT [32] and the gene ontology (GO) annotation of the *Arabidopsis* homolog when available, or other plants or algae when not. However, because one of these non-plastid proteins encoded an N-terminal extension (see Table 1) in comparison to the Chlamydiae and other prokaryotic homologs, it is possible that it also has an organellar target or alternatively, is a membrane protein.

**Table 1:** The 55 nuclear genes of chlamydial origin that we found in genome data from 17 Plantae species, and the putative cellular localizations of the encoded proteins.

Plantae gene annotation	Localization	Plantae gene annotation	Localization
Dimethyladenosine transferase (PFC1)	Chloroplast	4-diphosphocytidyl-2C-methyl-D-erythritol kinase	Chloroplast
Unknown protein (contains nucleotide-diphospho-sugar transferases domain)	Chloroplast	Na+/H+ antiporter, putative	Chloroplast
Phosphate transporter 2;1 (PHT2;1)	Chloroplast	Anthranilate phosphoribosyl transferase	Chloroplast
Phosphoglycerate/bisphosphoglycerate mutase family protein	Chloroplast	LL-diaminopimelate aminotransferase (AGD2)	Chloroplast
Exonuclease family protein	Chloroplast	Heavy metal ATPase 1 (HMA1)	Chloroplast
Pseudouridine synthase family protein	Chloroplast	Oligoendopeptidase F	Chloroplast
Malate dehydrogenase (NADP)	Chloroplast	Conserved hypothetical protein	Chloroplast
Phosphoribosylanthranilate isomerase (PAI2)	Chloroplast	Copper/Zinc superoxide dismutase family protein	Chloroplast
Granule-bound starch synthase I (Glycosyl transferase)	Chloroplast	Carbonic anhydrase 2 (CA2)	Chloroplast
D-alanine-D-alanine ligase B	Chloroplast	50S ribosomal protein-related	Mitochondrion
Plastidic ATP/ADP transporter	Chloroplast	Queuine tRNA-ribosyltransferase	Mitochondrion
Putative SAM dependent methyltransferases	Chloroplast	Manganese and iron superoxide dismutase	Mitochondrion
Cytidylyltransferase family	Chloroplast	Plasma membrane intrinsic protein 1c (PIP1C)	Membrane
tRNA/rRNA methyltransferase (SpoU) family protein	Chloroplast	Glycerol-3-phosphate transporter	Membrane
Unknown protein (S-adenosyl-L-methionine-dependent methyltransferases domain)	Chloroplast	Prolyl 4-hydroxylase, alpha subunit	N-terminal ext
Enoyl-[acyl-carrier-protein] reductase (MOD1)	Chloroplast	Unknown protein	
Rhodanese-like domain containing protein	Chloroplast	Sugar isomerase (SIS) domain-containing protein	
4-hydroxy-3-methylbut-2-en-1-yl diphosphate synthase (GcpE)	Chloroplast	Unknown protein (similar to zinc finger family protein)	
Pyrophosphate-dependent phosphofructokinase PfpB	Chloroplast	Dihydrouridine synthase, DuS	
UDP-glucuronate 4-epimerase 4 (GAE4)	Chloroplast	RNA-binding region containing protein	
3-oxoacyl-(acyl-carrier-protein) synthase I (KAS I)	Chloroplast	Lipoate protein ligase-like protein	
Isoamylase 3 (ISA3)	Chloroplast	Leucine rich repeat proteins	
Aminoacyl-tRNA synthetase, class Ib	Chloroplast	3′(2′),5′-bisphosphate nucleotidase (SAL2) (phosphatidylinositol phosphatase)	
2-C-methyl-D-erythritol 4-phosphate cytidyltransferase (ISPD)	Chloroplast	tRNA isopentenyltransferase (ATIPT9)	
Methylase-related	Chloroplast	FOG: PPR repeat	
Conserved hypothetical protein	Chloroplast	Cytidine/deoxycytidylate deaminase	
Glycerol-3-phosphate acyltransferase	Chloroplast	Predicted nucleic acid-binding protein ASMTL	
Polyribonucleotide phophorylase	Chloroplast		

**Figure 1:**
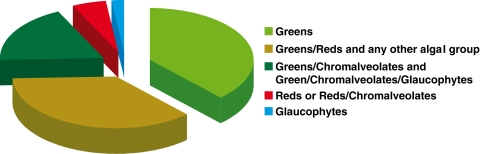
Pie chart showing the distribution of Chlamydiae-like genes among Plantae and chromalveolates.

Thirty-one of the Chlamydiae genes were present in the green algae plus plants clade (with or without chromalveolates) and 20 were shared by at least red and green algae, thereby suggesting their ancient origins in the Plantae (see Figure 1). An expanded list of protein characteristics is provided in Table S1 and the RAxML bootstrap trees are presented in Table S2. Our ability to identify a larger set of Chlamydiae genes than Huang and Gogarten [22] likely reflected the fact that we used the combined protein set from 17 Plantae genomes, thereby including as large a diversity of query sequences as possible. As also noted by Huang and Gogarten [22], *C. merolae* has a highly reduced nuclear genome (16.5 Mb; 5,331 genes [33]), therefore some genes (e.g., Fig. 1A, 1C) absent from this species could still be present in the “normal-sized” genomes of mesophilic green algae (e.g., *Chlamydomonas reinhardtii*, 120 Mb; >15,000 genes [34]) and plants. Consistent with this idea, 32 of the genes we found of Chlamydiae origin were undetected in red algae. Many of these genes may have been lost from the Cyanidiales, or diverged beyond detection using our bioinformatic pipeline, or are independent gains in the green lineage. More extensive data are needed from mesophilic red algae to address this issue. Currently we only had available partial EST data from non-Cyanidiales red algae.

**Figure 2:**
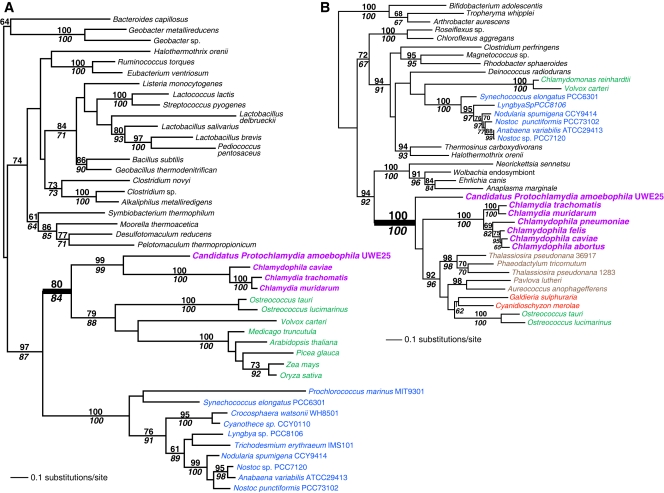
Maximum likelihood (RAxML) trees of Chlamydiae-derived genes in the Plantae. A) The tree of dimethyladenosine transferase (PFC1). B) The tree of queuine tRNA-ribosyltransferase. The results of a bootstrap analysis using RAxML are shown above the branches, whereas PHYML bootstrap support values are shown below the branches. Only bootstrap values ≥60% are shown. Branch lengths are proportional to the number of substitutions per site (see scale bars). Cyanobacteria are shown in blue text, green algae and land plants in green text, red algae in red, chromalveolates in brown, and Chamydiae in magenta. All other bacteria are shown in black text. The thick branches unite Chlamydiae and Plantae.

**Figure 3:**
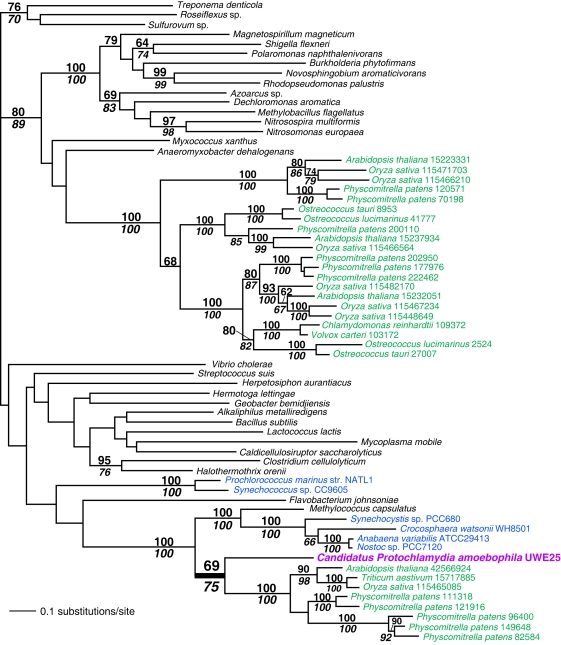
Maximum likelihood (RAxML) tree of the Chlamydiae-derived Plantae protein, glycogen synthase. The results of a bootstrap analysis using RAxML are shown above the branches, whereas PHYML bootstrap support values are shown below the branches. Only bootstrap values ≥60% are shown. Branch lengths are proportional to the number of substitutions per site (see scale bar). Cyanobacteria are shown in blue text, green algae and land plants in green text, and Chamydiae in magenta. All other bacteria are shown in black text. The thick branches unite Chlamydiae and Plantae.

**Figure 4:**
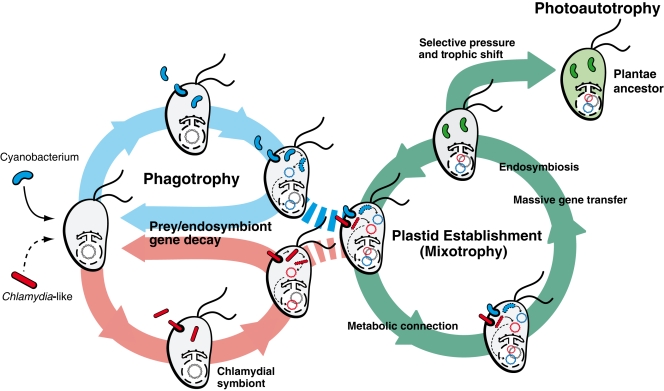
Mixotrophy hypothesis for the origin of the primary plastid in algae and plants. The ancestor of Plantae was a phagotrophic protist that consumed cyanobacteria as food and was parasitized by environmental Chlamydiae. Gene transfers from both prokaryotic sources to the host nucleus either resulted in their decay and loss or occasional gene replacement as observed in modern-day protists [25], [43]. The transition to mixotrophy was facilitated by the transfer and activation of key genes from the cyanobacterium such as those that regulate the cell cycle. The mixotrophic Plantae ancestor continued to consume bacterial prey but over time, harvesting genes from Chlamydiae and eventually developed a regulated metabolic connection (e.g., for the export of fixed carbon compounds) between the newly established endosymbiont and the host cytosol [21] and a system for protein import into the endosymbiont using the host secretory system [11]. These developments cemented the relationship and led to selection for massive gene loss in the endosymbiont and EGT to the host nucleus. Activation of at least 37 genes recruited from Chlamydaie further enhanced plastid functions. The final transition occurred in a prey-poor environment that favored phototrophy. Thereafter this ancestral alga lost the ability for phagotrophy and diversified into the extant lineages of green, red, and glaucophyte algae.

Examples of novel genes we found are shown in Figs. 2, 3, and 4. In Fig. 2A, we present the phylogeny of PFC1 (a plastid-targeted RNA methylase that is essential for low-temperature development of chloroplasts [35]) that shows a clear affiliation of green algae to Chlamydiae (RAxML bootstrap, RB = 80%, PHYML [36] bootstrap, PB = 84%) and this clade is sister to cyanobacteria (RB = 97%, PB = 87%). One possible explanation of cases with a cyanobacteria-Chlamydiae-Plantae connection is that Chlamydiae may be sister to or in the past exchanged genes with cyanobacteria (i.e., the plastid donor) and therefore their close relationship is a reflection of the bacterial tree rather than endosymbiotic gene transfer (EGT)/HGT from the former group (see [15] and [22] for a detailed discussion of this scenario). A different sort of topology is shown in Fig. 2B in which there are two types of queuine tRNA-ribosyltransferase genes (a tRNA-guanine transglycosylase; putatively mitochondrial targeted in algae and plants) in Plantae, one is putatively derived from cyanobacteria in chlorophytes (i.e., *Chlamydomonas* and *Volvox*) and another from Chlamydiae in red algae, chromalveolates, and prasinophytes (i.e., *Ostreococcus* spp.; PB, RB = 100%). This tree is likely explained by differential gene loss in green algae with red and prasinophyte algae retaining the Chlamydiae gene (that was subsequently transferred to chromalveolates via red or green algal secondary endosymbiosis) and green algae the cyanobacterial copy. A third example of the types of genes we found is *glg*A (glycogen synthase) that is shown in Fig. 3. This gene catalyzes starch synthesis and there are two gene copies in plants, one that is derived from Chlamydiae (specifically *Candidatus Protochlamydia amoebophila* UWE25; RB = 69%, PB = 75%) and another that is shared by many greens and is derived from an unknown prokaryotic source. Both genes function in the chloroplast. This is the second gene of chlamydial origin that is involved in a carbohydrate metabolic process (i.e., the first is the starch debranching enzyme ATISA3; Table 1). An interesting point about Fig. 3 is that it shows a specific relationship between UWE25 and Plantae (see also [22]). This environmental Chlamydiae species (symbiont in *Acanthamoeba*, [18]) was found to be sister to Plantae in 30/55 trees (Table S1) identifying it as the closest living relative in our data set of the endoparasite that donated the genes. Complete genomes from other environmental Chlamydiae are needed to more comprehensively address the issue of the potential gene donor(s) in Plantae. UWE25 is a member of ECL V clade of Chlamydiae [20] with a genome (2.4 Mb) that is twice the size of the more highly derived vertebrate pathogens and in contrast to the latter, retains a complete TCA cycle. UWE25 however, still encodes an ADP/ATP translocator and is presumably an endoparasite of *Acanthamoeba* [16]).

An important insight from our study is the identification of 18 genes of putative non-plastid functions that were contributed by Chlamydiae to Plantae. As described above, these are bioinformatic predictions for the cellular location that await verification using proteomic methods and many contain a N-terminal extension potentially indicating a plastid target. In spite of these caveats, our data suggest that the relationship between Chamydiae and their Plantae hosts was likely to not have been limited to plastid functions but rather affected the mitochondrion and other aspects of the nuclear genome. In addition, we found two proteins (cytidine/deoxycytidylate deaminase family protein and an unknown protein, similar to zinc finger family protein) in which only the C-terminal domain was of Chlamydiae origin. This suggests the fusion of a eukaryotic and a prokaryotic sequence gave rise to these genes. Finally, a number of proteins were identified (acid phosphatase survival protein, SurE, gi:15218620; embryo defective 2394 gi:15220443; oxoglutarate:malate antiporter, DIT1, gi:30684152; RAN GTPase activator, RANGAP2, gi:15239712; peptide deformylase, PDF1B, gi:15241461; ubiquitin-protein ligase, EBF1, gi:18400846; mechanosensitive ion channel domain-containing protein, gi:22328173; and ribosome recycling factor family protein, gi:42563413) that showed Plantae-Chlamydiae monophyly but fell below the 50% bootstrap threshold. These trees were not counted in our final tally but may in the future turn out to also be of chlamydial origin.

## Discussion

Although the origin of the 55 Chlamydiae-like genes in Plantae may appear to be most easily explained by a long-term endosymbiotic (e.g., organelle-like) association between these prokaryotes and the host, it is also worth considering whether these sequences may have arisen from multiple Chlamydiae sources and could simply reflect a long history of endoparasitism. For example, even though many (20) of the Chlamydiae-like genes were present in both red and green algae (and chromalveolates via EGT), others were detected only in red algae (3) and glaucophytes (1). This pattern could be explained either by wholesale gene loss in Plantae lineages or multiple HGTs in these taxa. Speaking against the latter scenario is the apparent absence of Chlamydiae endoparasites in extant algae and plants, although this certainly may not have been the case ca. 1 billion years ago when Plantae radiated [18]. UWE25 plays a key role in this discussion. This strain represents an anciently diverged lineage whose ancestors were likely contemporary with the Plantae ancestor. However the environmental Chlamydiae are represented by only a single genome in our analysis (i.e., UWE25). Therefore although many UWE25 genes had a sister group relationship with Plantae (i.e., 30/55), in other trees UWE25 was either not present (12 trees) or when in the tree did not show a specific affiliation with Plantae to the exclusion of other Chlamydiae (13 cases). An example of the latter group is shown in Figure 2A. Furthermore, as described above, because the vertebrate pathogens have highly reduced genomes, many Chlamydiae genes that are shared uniquely by UWE25 and Plantae (19 genes) may simply be cases of widespread gene loss in other endoparasites, giving the misleading impression of a specific relationship between UWE25 and algae/plants. The addition of many more environmental Chlamydiae genomes may help us determine whether the “big-genomed” environmental UWE25 shares a specific relationship with Plantae or whether other environmental strains that also contain these genes would break this relationship. We must however keep in mind the prospect that due to the long passage of time and HGT among bacteria, it may be impossible to find the specific donor(s) of the Chlamydiae genes, as is the case for the plastid primary endosymbiont (e.g., [37]). Our work does bring up the possibility that even if an endosymbiosis (i.e., EGT) explains the origin of the majority of Chlamydiae genes in Plantae [22], multiple HGTs may also have played a key role in gene acquisition. The relative contribution of these forces awaits future investigation.

The presence of a type IV secretion system (TFSS) in the sequenced genome of UWE25 that is missing in pathogenic strains provides a mechanism by which environmental Chlamydiae DNA could integrate into the host genome [38]. Horn *et al.* [18] suggested that UWE25 lacks the genes for this capacity but retains the ability to secrete effector bacterial proteins into the amoebal host. However, a subsequent study by Greub *et al.* [39] reported *tra* genes in UWE25 of proteobacterial origin and they proposed these could play a role in conjugative DNA transfer. The Chlamydiae genes that survived in the nucleus of Plantae either replaced host proteins (e.g., CSD1, copper/zinc superoxide dismutase 1) or provided novel functions (e.g., plastid metabolism or solute transport) and were therefore retained, whereas the majority of the endoparasite genes (2,031 protein coding genes exist in UWE25) that were potentially transferred were lost over time. If the Chlamydiae-like cell was in fact a *bona fide* organelle then its loss would have precipitated the decay of many genes that served this (albeit unknown) compartmental function. This explains why the Chlamydiae contribution to Plantae algal nuclear genomes pales in comparison to that of the permanent cyanobacterial endosymbiont, i.e., ca. 1,000–1,500 [40] vs. ca. 55 genes, respectively. The EGT or HGT ratchet clearly favored the survival of Chlamydiae-like genes that increased the metabolic connection between the host and the cyanobacterial endosymbiont. Consistent with this idea, Chlamydiae provided some key plastid translocators in Plantae such as the ADP/ATP translocator and the copper transporter Heavy Metal ATPase (for details, see [21]). As described above, we also identified the plastid dicarboxylate translocators DiT1, DiT2.1, and DiT2.2 that we previously hypothesized to be of Chlamydiae origin [21]. This clade however received only 41% bootstrap support in this study (results not shown) and was therefore not included in our final list. Another transporter (sodium:hydrogen antiporter) identified by Huang and Gogarten [22] also appears in our data set showing the monophyly of Chlamydiae and Plantae. However, a single prokaryote branches within this clade, the Deltaproteobacteria, *Plesiocystis pacifica* SIR-1 (see Table S1). This gene likely originated in *Plesiocystis* via an independent HGT from a Chlamydiae source. We found a few other examples in which a single prokaryote or excavate (e.g., *Naegleria*, *Acanthamoeba*) interrupted the Chlamydiae+Plantae clade and we interpret these also as examples of HGTs into the “contaminating” taxa.

### Model of plastid origin

Here we apply our knowledge of Chlamydiae gene transfers and other aspects of plastid evolution to propose a model for the origin of this organelle. We assume that in the phagotrophic Plantae ancestor, diverse bacteria were captured with many cyanobacteria retained for harvesting fixed carbons, whereas others were digested. It is therefore easy to imagine that over time prey DNA integrated via non-homologous recombination into the host genome. An analogous process operates in modern-day eukaryotes when organelles undergo degradation, thereby increasing the rate of DNA integration into the nuclear genome [41], [42]. A recent survey of genomes from protists that consume bacteria identified several prokaryote-derived genes (presumably through HGT) in these taxa [25], [43], [44]. This result is a prediction of the “you are what you eat” hypothesis [24]. Given that foreign genes are continually introduced into the nuclear genome of eukaryotes, what is their fate in nature? This issue has been most comprehensively studied in plants, where it has been demonstrated that loss is the prevalent destiny for transferred organelle DNA (i.e., in the absence of artificial selection). Recent plastid and mitochondrial DNA integrants in plants are rapidly lost to mutation, fragmentation, and shuffling that counteract gene activation [45], [46]. In summary, current data demonstrate that although plausible mechanisms exist for the transfer, integration, and activation of foreign genes in the nucleus, and there is clear proof for HGTs in predatory protists, these forces are counteracted by pervasive gene inactivation.

This scenario could change however if some randomly activated prey genes in the host nucleus were selected and fixed in the population [47], [48] allowing them to survive within populations. The leading theory to explain EGT is Muller’s ratchet which is the accumulation of deleterious mutations in non-recombining (i.e., organellar) genomes of small population size [49]. But what led to the establishment of the ratchet? The initial intracellular gene transfer was likely driven by the evolution of a metabolically stable connection between the predator and the photosynthetic prey that resulted in a mixotrophic life-style (Fig. 4). We suggest that mixotrophy provided the opportunity for the establishment of the first plastid with the accumulation of endosymbiont and Chlamydiae genes in the nucleus via EGT and HGT, with their activation and eventual retention (Fig. 4). Key genes such as those involved in cell division and the cell cycle or gene expression regulation (e.g., redox regulators) and nutrient/metabolite transport (e.g., triose-phosphates, ADP/ATP) are likely candidates to have been initially retained in the nuclear genome of these primitive mixotrophs. It is also formally possible that some key Chlamydiae genes may already have been transferred to the Plantae host via HGT prior to the cyanobacterial endosymbiosis, thereby increasing the probability of plastid fixation.

Once these pivotal events had taken place, the path was paved for the subsequent evolutionary innovations that characterize canonical plastids such as the TOC-TIC translocons, the origin of import signals at the N-terminus of many nuclear-encoded plastid targeted proteins, extensive EGT, and outright endosymbiont gene loss resulting in highly reduced plastid genomes (for details, see [11], [14]). The majority of proteins that comprise the plastid proteome arose from two sources; i.e., several hundred came from co-option of genes already present in the host’s nuclear genome and 1,000–1,500 came from the cyanobacterial endosymbiont (e.g., [11], [14]). Our analysis shows that a third source, Chlamydiae provided a relatively minor (ca. 55 genes) but essential set of sequences that provided the Plantae ancestor with many novel nuclear genes, two-thirds of which provided tools to harness its newly acquired plastid. This valuable commodity (i.e., eukaryotic photosynthesis) thereafter spread throughout the eukaryotic tree of life via secondary and tertiary endosymbioses.

## Materials and Methods

To identify genes of putative Chlamydiae origin in Plantae we first screened 12,061 predicted proteins from seven Chlamydiae genomes (*Candidatus Protochlamydia amoebophila* UWE25, *Chlamydia muridarum* Nigg, *Chlamydia trachomatis* A/HAR-13, *Chlamydophila abortus* S26/3, *Chlamydophila caviae* GPIC, *Chlamydophila felis* Fe/C-56, *Chlamydophila pneumoniae* AR39) using reciprocal BLAST (WU-BLAST with e-value <10^−3^) against a 17-species Plantae database derived from complete genome and EST data (a total of 104,495 sequences). The Plantae included 7 green algae and land plants *(Arabidopsis thaliana*, *Chlamydomonas reinhardtii*, *Oryza sativa, Ostreococcus lucimarinus, Ostreococcus tauri*, *Physcomitrella patens*, *Volvox carteri*), 8 red algae *(Chondrus crispus*, *Cyanidioschyzon merolae*, *Cyanidium caldarium*, *Galdieria sulphuraria*, *Gracilaria changii*, *G. tenuistipitata*, *Porphyra yezonesis, P. purpurea*,), and 2 glaucophytes (*Cyanophora paradoxa*, *Glaucocystis nostochinearum*). This search identified 16,173 candidate proteins.

Thereafter, we used PhyloGenie [50] to run a phylogenomic analysis of the 16,173 Plantae candidates against a local database comprised of >500 genomes (17 Plantae, 6 chromalveolates, 14 cyanobacteria, 3 animals, 5 fungi, 504 bacteria, and 2 Amoebozoa (Table S3) for a total of 2,629,817 protein sequences. The PhyloGenie BLAST e-value cut-off was set at <10^−10^ and distance trees were generated using neighbor-joining (NJ) with Poisson distance correction and 100 replicates of a bootstrap analysis. We then used our recently developed tree topology search tool PhyloSort to identify all NJ trees that showed the monophyly of Chlamydiae and Plantae (with or without chromalveolates included within the clade). PhyloSort is used to search for user-specified subtrees that contain a specified monophyletic group. Because a genome-wide analysis produces a significant number (i.e., 1000s) of trees that share multiple genes due to multiple gene copies and gene families, PhyloSort provides an estimate of the number of unique gene families by clustering the trees that contain overlapping genes to summarize these families. Using PhyloSort and a minimum threshold value of 50% bootstrap support we found 345 trees that fulfilled this topological criterion. These 345 alignments were then used for a second round of phylogenetic analysis using bootstrap (100 replicates) maximum likelihood (ML) phylogeny inference with RAxML using the hill-climbing algorithm and the WAG substitution model, the PROTGAMMA (+Γ) implementation with 4 discrete rate categories and starting from a random tree. The ML method was used to verify the results of the less robust NJ analysis and reduced the set of target trees to 291. Clustering of the RAxML bootstrap trees using PhyloSort resulted in a “unitree” set of 68 phylogenies each of which was manually inspected to verify the topology.

Plantae homologs of the 68 Chlamydiae proteins were then used to query a final data set that included all previous data but with the addition of excavate and other protist partial EST data downloaded from GenBank dbEST [51] and TBestDB [52]. This analysis included taxa such as jakobids and Rhizaria (see Table S3) that might branch within and disrupt the target Chlamydiae/Plantae/chromalveolate clade identified in our study. This procedure resulted in the further refinement of our data set to 55 trees that were submitted to RAxML bootstrap analysis (presented in Table S2). We chose 3 proteins to illustrate the variety of topologies that were found using our phylogenomic pipeline. These are dimethlyadenosine transferase (PFC1, 247 aa), queuine tRNA-ribosyltransferase (353 aa), and glycogen synthase (glgA, 430 aa). We used RAxML to infer the trees and used both RAxML (100 replicates) and PHYML (200 replicates) as described above to infer bootstrap support for the nodes in these trees.

## Supporting Information

Table S1 The list of 55 genes of Chlamydiae-like origin that were found in the 17 Plantae genomes analyzed in this study using phylogenomic methods. Shown are the tree IDs, the source of the sequences, the GI/accession numbers in the source database, the gene annotations in that database, the Arabidopsis homolog GI numbers and putative functions, the Chlamydiae homolog GI numbers and putative functions, the minimum bootstrap value that unites Chlamydiae and Plantae in each protein tree, the distribution of the genes in Plantae (G, R, C, and X indicate presence in green algae/plants, red algae, chromalveolates, and glaucophytes, respectively), the results of targeting predicitions using 5 different programs, our inferred predictions for cellular localization, and the phylogenetic position of UWE25 in each protein tree (i.e., Y, when UWE25 was sister to the Plantae and N, when not).
(0.33 MB PDF)

Table S2 The 55 RAxML bootstrap trees that were identified by our phylogenomic analysis to support the monophyly of Chlamydiae and Plantae (with or without chromalveolates). The tree ID (see Table S1) precedes each tree.
(1.56 MB PDF)

Table S3 Complete set of taxa present in our local genome database that was used to search for chlamydial genes in Plantae.
(0.24 MB PDF)
