# Supplementary material for: Chlamydiae Has Contributed at Least 55 Genes to Plantae with Predominantly Plastid Functions
Source: PLoS One. 2008 May 21;3(5):e2205. doi: 10.1371/journal.pone.0002205 (PMC2376095; doi:10.1371/journal.pone.0002205)
Supplement: Table S1 — The list of 55 genes of Chlamydiae-like origin that were found in the 17 Plantae genomes analyzed in this study using phylogenomic methods. Shown are the tree IDs, the source of the sequences, the GI/accession numbers in the source database, the gene annotations in that database, the Arabidopsis homolog GI numbers and putative functions, the Chlamydiae homolog GI numbers and putative functions, the minimum bootstrap value that unites Chlamydiae and Plantae in each protein tree, the distribution of the genes in Plantae (G, R, C, and X indicate presence in green algae/plants, red algae, chromalveolates, and glaucophytes, respectively), the results of targeting predicitions using 5 different programs, our inferred predictions for cellular localization, and the phylogenetic position of UWE25 in each protein tree (i.e., Y, when UWE25 was sister to the Plantae and N, when not). (0.33 MB PDF) [file pone.0002205.s001.pdf]

Tree ID	Database	Accession	Annotation	Arabidopsis homolog	Acession	Chlamydiae homolog	Acession	Bootstrap	Plantae distn.	WoLF PSORT	TargetP	Predotar	ChloroP	PSLDoc	Prediction	UWE25
Plantae-Arabidopsis_thaliana_15220982	GenBank	15220982	PFC1 (PALEFACE 1)	PFC1 (PALEFACE 1)	15220982	dimethyladenosine transferase	161611309	75	G	chlo: 10.0	chlo: 0.97	chlo: 0.59	chlo: 0.51	chlo: 0.67	Chloroplast	Y
Plantae-Arabidopsis_thaliana_15225451	GenBank	15225451	GAE4 (UDP-D-GLUCURONATE 4-EPIMERASE 4); catalytic	GAE4 (UDP-D-GLUCURONATE 4-EPIMERASE 4); catalytic	15225451	probable UDP-glucuronat epimerase	46445713	75	GR	chlo: 2.0	none: 0.85	none: 0.97	chlo: 0.54	chlo: 0.13	Chloroplast	Y
Plantae-Arabidopsis_thaliana_15237422	GenBank	15237422	KAS I (3-KETOACYL-ACYL CARRIER PROTEIN SYNTHASE I); fatty-acid synthase	KAS I (3-KETOACYL-ACYL CARRIER PROTEIN SYNTHASE I); fatty-acid synthase	15237422	3-oxoacyl-(acyl carrier protein) synthase II	46446872	75	GR	chlo: 9.5	chlo: 0.89	chlo: 0.54	chlo: 0.58	chlo: 0.82	Chloroplast	Y
Plantae-Arabidopsis_thaliana_15240411	GenBank	15240411	Exonuclease family protein	Exonuclease family protein	15240411	DNA polymerase III subunit epsilon	46446795	75	G	chlo_mito: 5.8	chlo: 0.78	chlo: 0.81		chlo: 0.05	Chloroplast	N
Plantae-Arabidopsis_thaliana_18395376	GenBank	18395376	ISPD (2-C-METHYL-D-ERYTHRITOL 4-PHOSPHATE CYTIDYLTRANSFERASE)	ISPD (2-C-METHYL-D-ERYTHRITOL 4-PHOSPHATE CYTIDYLTRANSFERASE)	18395376	putative 4-diphosphocytidyl-2C-methyl-D-erythritol synthase	46445961	75	GRC	chlo: 13.0	mito: 0.26	chlo: 0.5	chlo: 0.53	mito: 0.47	Chloroplast	Y
Plantae-Arabidopsis_thaliana_18396215	GenBank	18396215	MOD1 (MOSAIC DEATH 1); enoyl-[acyl-carrier-protein] reductase (NADH)/ oxidoreductase	MOD1 (MOSAIC DEATH 1); enoyl-[acyl-carrier-protein] reductase (NADH)/ oxidoreductase	18396215	enoyl-(acyl carrier protein) reductase	46446786	75	GC	chlo: 11.0	chlo: 0.89	chlo: 0.91	chlo: 0.55	chlo: 0.37	Chloroplast	N
Plantae-Arabidopsis_thaliana_18410414	GenBank	18410414	Unknown protein (contains nucleotide-diphospho-sugar transferases domain)	Unknown protein (contains nucleotide-diphospho-sugar transferases domain)	18410414	hypothetical protein pc0324	46445958	75	G	chlo: 7.0	chlo: 0.95	chlo: 0.63	chlo: 0.59	chlo: 0.06	Chloroplast	Y
Plantae-Arabidopsis_thaliana_18418270	GenBank	18418270	AGD2 (ABERRANT GROWTH AND DEATH 2); transaminase	AGD2 (ABERRANT GROWTH AND DEATH 2); transaminase	18418270	L,L-diaminopimelate aminotransferase	46446319	75	GRCX	chlo: 13.0	chlo: 0.86	chlo: 0.51	chlo: 0.57	chlo: 0.58	Chloroplast	Y
Plantae-Arabidopsis_thaliana_22328517	GenBank	22328517	ATISA3/ISA3 (ISOAMYLASE 3); alpha-amylase	ATISA3/ISA3 (ISOAMYLASE 3); alpha-amylase	22328517	probable isoamylase	46446740	75	GR	chlo: 11.5	chlo: 0.98	chlo: 0.67	chlo: 0.59	chlo: 0.14	Chloroplast	Y
Plantae-Arabidopsis_thaliana_30684861	GenBank	30684861	Methylase-related	Methylase-related	30684861	hypothetical protein pc1620	46447254	50	GRC	chlo: 6.0	chlo: 0.83	chlo: 0.79	chlo: 0.55	chlo: 0.07	Chloroplast	N
Plantae-Arabidopsis_thaliana_30687794	GenBank	30687794	Pseudouridine synthase family protein	Pseudouridine synthase family protein	30687794	putative ribosomal large chain pseudouridine synthase B	46445794	75	G	chlo: 11.0	chlo: 0.68	chlo: 0.97	chlo: 0.57	nucl: 0.14	Chloroplast	N
Plantae-Arabidopsis_thaliana_30688378	GenBank	30688378	PHT2;1 (phosphate transporter 2;1)	PHT2;1 (phosphate transporter 2;1)	30688378	Phosphate Permease	15618590	75	G	chlo: 8.0	none: 0.45	none: 0.99	chlo: 0.50	mito: 0.01	Chloroplast	Y
Plantae-Arabidopsis_thaliana_30691669	GenBank	30691669	tRNA/rRNA methyltransferase (SpoU) family protein	tRNA/rRNA methyltransferase (SpoU) family protein	30691669	hypothetical protein pc0141	46445775	75	GC	chlo: 12.0	none: 0.25	none: 0.86		chlo: 0.79	Chloroplast	Y
Plantae-Arabidopsis_thaliana_30697049	GenBank	30697049	Malate dehydrogenase (NADP)	Malate dehydrogenase (NADP)	30697049	malate dehydrogenase	46447406	75	G	chlo: 12.0	chlo: 0.96	chlo: 0.78	chlo: 0.56	chlo: 0.00	Chloroplast	N
Plantae-Arabidopsis_thaliana_42563306	GenBank	42563306	Phosphoglycerate/bisphosphoglycerate mutase family protein	Phosphoglycerate/bisphosphoglycerate mutase family protein	42563306	phosphoglyceromutase	46445795	75	G	chlo: 11.0	chlo: 0.86	chlo: 0.75	chlo: 0.56	nucl: 0.49	Chloroplast	Y
Plantae-Arabidopsis_thaliana_42565237	GenBank	42565237	Unknown protein (contains domain S-adenosyl-L-methionine-dependent methyltransferases)	Unknown protein (contains domain S-adenosyl-L-methionine-dependent methyltransferases)	42565237	putative 23S rRNA (Uracil-5-)-methyltransferase	46447632	75	GC	chlo: 5.0	chlo: 0.84	chlo: 0.77	chlo: 0.56	cyto: 0.22	Chloroplast	Y
Plantae-Arabidopsis_thaliana_42573275	GenBank	42573275	PAI2 (PHOSPHORIBOSYLANTHRANILATE ISOMERASE 2); phosphoribosylanthranilate isomerase	PAI2 (PHOSPHORIBOSYLANTHRANILATE ISOMERASE 2); phosphoribosylanthranilate isomerase	42573275	N-(5'-phosphoribosyl)anthranilate isomerase	15835220	75	G	chlo: 13.0	chlo: 0.83	none: 0.85	chlo: 0.56	chlo: 0.72	Chloroplast	N
Plantae-Arabidopsis_thaliana_42573371	GenBank	42573371	CA2 (BETA CARBONIC ANHYDRASE 2); carbonate dehydratase/ zinc ion binding	CA2 (BETA CARBONIC ANHYDRASE 2); carbonate dehydratase/ zinc ion binding	42573371	putative carbonate dehydratase, cynT	46446692	75	X	cyto: 6.0	none: 0.67	chlo: 0.52	chlo: 0.54	chlo: 0.97	Chloroplast	Y
Plantae-Chlamydomonas_reinhardtii_139405	JGI	139405	Aminoacyl-tRNA synthetase, class Ib	tRNA synthetase class I (W and Y) family protein	15232946	probable tyrosine-tRNA ligase	46446803	75	GR	chlo: 13.0	chlo: 0.86	chlo: 0.84	chlo: 0.58	chlo: 0.62	Chloroplast	Y
Plantae-Chlamydomonas_reinhardtii_155606	JGI	155606	Conserved hypothetical protein	YebC-related	18400953	hypothetical protein CPn0573	15618484	75	GRC	chlo: 8.0	chlo: 0.58	chlo: 0.45	chlo: 0.57	chlo: 0.16	Chloroplast	N
Plantae-Chlamydomonas_reinhardtii_503	JGI	503	Granule-bound starch synthase I (Glycosyl transferase)	Starch synthase	15223331	glycogen synthase	46447230	75	G	chlo: 13.0	chlo: 0.91	chlo: 0.92	chlo: 0.56	cyto: 0.10	Chloroplast	Y
Plantae-Cyanidioschyzon_merolae_CMO211C	*C. merolae*	CMO211C	Conserved hypothetical protein	Unknown protein	15238214	hypothetical protein pc1708	46447342	75	RC	chlo: 11.0	mito: 0.60	ER: 0.48		chlo: 0.74	Chloroplast	Y
Plantae-Cyanidioschyzon_merolae_CMR313C	*C. merolae*	CMR313C	Oligoendopeptidase F			putative oligoendopeptidase F	46446812	50	R	chlo: 12.0	chlo: 0.77	mito: 0.62	chlo: 0.58	cyto: 0.17	Chloroplast	N
Plantae-Oryza_sativa_115438861	GenBank	115438861	Plastidic ATP/ADP transporter	ADP/ATP translocase	15218214	ADP/ATP Translocase	15604784	75	G	plas: 10.0	mito: 0.45	none: 0.78	chlo: 0.50	chlo: 0.76	Chloroplast	N
Plantae-Oryza_sativa_115460026	GenBank	115460026	Copper/Zinc superoxide dismutase family protein	CCS1 (copper chaperone for superoxide dismutase 1); superoxide dismutase copper chaperone	145335439	putative Superoxide dismutase (Cu-Zn)	46447393	75	GCX	chlo: 6.0	none: 0.87	none: 0.98		cyto: 0.66	Chloroplast	Y
Plantae-Oryza_sativa_115463187	GenBank	115463187	Rhodanese-like domain containing protein	Rhodanese-like domain-containing protein	18405495	hypothetical protein CCA00010	29839779	75	GC	chlo: 9.5	mito: 0.58	mito: 0.47	chlo: 0.56	chlo: 0.40	Chloroplast	N
Plantae-Oryza_sativa_115470767	GenBank	115470767	Polyribonucleotide phophorylase	RIF10 (RESISTANT TO INHIBITION WITH FSM 10); 3'-5'-exoribonuclease/ RNA binding / nucleic acid binding	30678905	polynucleotide phosphorylase/polyadenylase	46446277	75	GRC	chlo: 14.0	chlo: 0.97	none: 0.86	chlo: 0.57	chlo: 0.60	Chloroplast	N
Plantae-Oryza_sativa_115474235	GenBank	115474235	D-alanine-D-alanine ligase B	D-alanine--D-alanine ligase family	42572325	putative D-alanine--D-alanine ligase	46446032	75	G	chlo: 4.0	none: 0.65	chlo: 0.63	chlo: 0.50	chlo: 0.35	Chloroplast	Y
Plantae-Oryza_sativa_115483650	GenBank	115483650	Glycerol-3-phosphate acyltransferase	ATS1 (ACYLTRANSFERASE 1)	15222600	glycerol-3-phosphate acyltransferase	76789550	75	GRC	chlo: 14.0	chlo: 0.68	chlo: 0.94	chlo: 0.59	chlo: 0.92	Chloroplast	Y
Plantae-Ostreococcus_tauri_21840	JGI	21840	4-diphosphocytidyl-2C-methyl-D-erythritol kinase	ATCDPMEK (PIGMENT DEFECTIVE 277); 4-(cytidine 5'-diphospho)-2-C-methyl-D-erythritol kinase	15225820	probable isopentenyl monophosphate kinase (IPK)	46447223	75	GRC	chlo: 13.0	chlo: 0.79	chlo: 0.95	chlo: 0.58	chlo: 0.90	Chloroplast	Y
Plantae-Ostreococcus_tauri_37113	JGI	37113	Cytidylyltransferase family	cytidylyltransferase family	30695393	3-deoxy-manno-octulosonate cytidylyltransferase	89898215	75	G	chlo: 11.0	chlo: 0.61	none: 0.77	chlo: 0.50	chlo: 0.35	Chloroplast	N
Plantae-Ostreococcus_tauri_8662	JGI	8662	Putative SAM dependent methyltransferases	mraW methylase family protein	15238896	probable S-adenosyl-methyltransferase	46445945	75	G	chlo: 11.0	chlo: 0.67	mito: 0.63	chlo: 0.53	chlo: 0.40	Chloroplast	Y
Plantae-Physcomitrella_patens_218794	JGI	218794	Pyrophosphate-dependent phosphofructokinase PfpB	pyrophosphate--fructose-6-phosphate 1-phosphotransferase beta subunit, putative / pyrophosphate-dependent 6-phosphofructose-1-kinase, putative	15221156	diphosphate--fructose-6-phosphate 1-phosphotransferase	46446514	75	GC	chlo: 8.0	chlo: 0.59	chlo: 0.63		cyto: 0.31	Chloroplast	N
Plantae-Physcomitrella_patens_219372	JGI	219372	Anthranilate phosphoribosyl transferase	TRP1 (TRYPTOPHAN BIOSYNTHESIS 1); anthranilate phosphoribosyltransferase	15238711	anthranilate phosphoribosyltransferase	89898246	75	GRC	chlo: 11.0	chlo: 0.81	chlo: 0.9	chlo: 0.56	chlo: 0.64	Chloroplast	N
Plantae-Physcomitrella_patens_55802	JGI	55802	4-hydroxy-3-methylbut-2-en-1-yl diphosphate synthase	GcpE (CHLOROPLAST BIOGENESIS 4); 4-hydroxy-3-methylbut-2-en-1-yl diphosphate synthase	30697395	probable gcpE protein	46446374	75	GC	chlo: 9.0	chlo: 0.81	chlo: 0.79	chlo: 0.53	cyto: 0.42	Chloroplast	Y
Plantae-Physcomitrella_patens_60901	JGI	60901	Sodium sulfate symporter and related arsenite permeases	ATNHD1 (Arabidopsis thaliana Na/H antiporter 1); sodium:hydrogen antiporter	18402254	Na+/H+ antiporter, putative	16752011	75	GRC	chlo: 11.5	chlo: 0.95	chlo: 0.83	chlo: 0.58	mito: 0.06	Chloroplast	N
Plantae-Arabidopsis_thaliana_15235511	GenBank	15235511	HMA1 (Heavy metal ATPase 1); copper-exporting ATPase	HMA1 (Heavy metal ATPase 1); copper-exporting ATPase	15235511	putative cation transport related membrane protein	62185469	75	GRX	plas: 7.0	mito: 0.84	none: 0.68	chlo: 0.54	chlo: 0.77	Chloroplast envelope	N
Plantae-Arabidopsis_thaliana_15236554	GenBank	15236554	50S ribosomal protein-related	50S ribosomal protein-related	15236554	50S ribosomal protein L25/general stress protein Ctc	76789542	50	G	mito: 10.0	mito: 0.74	mito: 0.84		cyto: 0.08	Mitochondrion	N
Plantae-Chlamydomonas_reinhardtii_196649	JGI	196649	Manganese and iron superoxide dismutase	MSD1 (MANGANESE SUPEROXIDE DISMUTASE 1); manganese superoxide dismutase	15228407	superoxide dismutase	166154505	75	RC	nucl: 7.0	none: 0.51	ER: 0.77		chlo: 0.57	Mitochondrion	N
Plantae-Ostreococcus_tauri_18978	JGI	18978	Queuine tRNA-ribosyltransferase			queuine tRNA-ribosyltransferase	46446428	75	GRC	mito: 12.5	mito: 0.64	mito: 0.67		chlo: 0.85	Mitochondrion	N
Plantae-Arabidopsis_thaliana_18408421	GenBank	18408421	glycerol-3-phosphate transporter, putative / glycerol 3-phosphate permease, putative	glycerol-3-phosphate transporter, putative / glycerol 3-phosphate permease, putative	18408421	probable regulatory protein uhpC	46446021	75	G	plas: 9.0	none: 0.92	none: 0.97		chlo: 0.03	Membrane	N
Plantae-Arabidopsis_thaliana_15223438	GenBank	15223438	PIP1C (PLASMA MEMBRANE INTRINSIC PROTEIN 1;3); water channel	PIP1C (PLASMA MEMBRANE INTRINSIC PROTEIN 1;3); water channel	15223438	putative tonoplast intrinsic protein (Aquaporin)	46446430	75	G	plas: 12.0	none: 0.94	none: 0.99		plas: 0.93	Membrane	Y
Chromalveolata-Aureococcus_anophagefferens_65597	JGI	65597	Prolyl 4-hydroxylase, alpha subunit			hypothetical protein pc1581	46447215	75	GC	nucl: 8.0	none: 0.81	none: 0.99		nucl: 0.95	N-terminal extension	Y
Plantae-Arabidopsis_thaliana_15219361	GenBank	15219361	Unknown protein	Unknown protein	15219361	hypothetical protein pc0850	46446484	75	G	nucl: 10.0	none: 0.86	none: 0.99		nucl: 0.63		Y
Plantae-Arabidopsis_thaliana_15232565	GenBank	15232565	Sugar isomerase (SIS) domain-containing protein	Sugar isomerase (SIS) domain-containing protein	15232565	putative Gut Q protein	46447416	75	G	nucl: 7.5	none: 0.83	none: 0.96		chlo: 0.50		Y
Plantae-Arabidopsis_thaliana_15237589	GenBank	15237589	SAL2; 3'(2'),5'-bisphosphate nucleotidase/ inositol or phosphatidylinositol phosphatase	SAL2; 3'(2'),5'-bisphosphate nucleotidase/ inositol or phosphatidylinositol phosphatase	15237589	putative sulfur metabolism-related protein	62185419	75	GRC	ER: 5.0	none: 0.26	none: 0.99		chlo: 0.17		N
Plantae-Arabidopsis_thaliana_18396238	GenBank	18396238	Unknown protein (similar to zinc finger family protein)	Unknown protein (similar to zinc finger family protein)	18396238	hypothetical protein pc1119	46446753	75	G	nucl: 10.0	none: 0.52	none: 0.89	chlo: 0.50	golgi: 0.10		Y
Plantae-Arabidopsis_thaliana_30687613	GenBank	30687613	ATIPT9 (Arabidopsis thaliana isopentenyltransferase 9); ATP binding / tRNA isopentenyltransferase	ATIPT9 (Arabidopsis thaliana isopentenyltransferase 9); ATP binding / tRNA isopentenyltransferase	30687613	tRNA delta(2)-isopentenylpyrophosphate transferase	46446877	75	GRC	chlo: 7.5	mito: 0.75	mito: 0.48	chlo: 0.51	nucl: 0.80		N
Plantae-Chlamydomonas_reinhardtii_104193	JGI	104193	Dihydrouridine synthase, DuS	Dihydrouridine synthase family protein	15234862	putative protein involved in tRNA-dihydrouridine synthesis	46447213	75	G	nucl: 13.0	none: 0.94	none: 0.98		nucl: 0.84		Y
Plantae-Oryza_sativa_115452347	GenBank	115452347	RNA-binding region containing protein	RNA recognition motif (RRM)-containing protein	15232579	probable nucleic acid-binding protein	46446527	75	G	nucl: 12.0	none: 0.97	none: 0.97		mito: 0.03		Y
Plantae-Oryza_sativa_115476574	GenBank	115476574	Lipoate protein ligase-like protein	Unknown protein	15228560	putative lipoate-protein ligase	46447472	75	GC	cyto: 9.0	none: 0.87	none: 0.98		chlo: 0.18		Y
Plantae-Ostreococcus_lucimarinus_31465	JGI	31465	FOG: PPR repeat	Pentatricopeptide (PPR) repeat-containing protein	15222150	tRNA (guanine-N(7))-methyltransferase	62185355	75	GRC	cyto: 9.0	none: 0.73	none: 0.99		chlo: 0.02		N
Plantae-Ostreococcus_tauri_31283	JGI	31283	Predicted nucleic acid-binding protein ASMTL	Maf family protein	22328154	hypothetical protein CTL0603	166154561	75	GRCX	cyto: 8.0	mito: 0.59	mito: 0.76		cyto: 0.30		N
Plantae-Physcomitrella_patens_70762	JGI	70762	Cytidine/deoxycytidylate deaminase	Cytidine/deoxycytidylate deaminase family protein	15221490	cytosine/adenosine deaminases	89898061	75	GRC	nucl: 11.0	mito: 0.68	chlo: 0.66		nucl: 0.40		N
Plantae-Volvox_carteri_99233_jgi	JGI	99233	Leucine rich repeat proteins	Leucine-rich repeat family protein	30684506	hypothetical protein pc1032	46446666	75	GC	nucl: 8.0	none: 0.76	none: 0.86		chlo: 0.08		Y

Table S1. The list of 55 genes of Chlamydiae-like origin that were found in the 17 Plantae genomes analyzed in this study using phylogeno mic methods. Shown are the tree IDs, the source of the sequences, the GI/accession numbers in the source database, the gene annotations in that database, the *Arabidopsis* homolog GI numbers and putative functions, the Chlamydiae homolog GI numbers and putative functions, the minimum bootstrap va lue that unites Chlamydiae and Plantae in each protein tree, the distribution of the genes in Plantae (G, R, C, and X indicate presenc e in green algae/plants, red algae, chromalveolates, and glaucophytes, respectively), the results of
