# Supplementary material for: Chlamydiae Has Contributed at Least 55 Genes to Plantae with Predominantly Plastid Functions
Source: PLoS One. 2008 May 21;3(5):e2205. doi: 10.1371/journal.pone.0002205 (PMC2376095; doi:10.1371/journal.pone.0002205)
Supplement: Table S2 — The 55 RAxML bootstrap trees that were identified by our phylogenomic analysis to support the monophyly of Chlamydiae and Plantae (with or without chromalveolates). The tree ID (see Table S1) precedes each tree. (1.56 MB PDF) [file pone.0002205.s002.pdf]

Table S2. The 55 RAxML bootstrap trees that were identified by our phylogenomic analysis to support the monophyly of Chlamydiae and Plantae (with or without chromalveolates). The tree ID (see Table S1) precedes each tree.

Chromalveolata-Aureococcus_anophagefferens_65597
(((Rhizaria-Bigelowiella_natans_DR040100_1:0.72724206751884379329,(Plantae-Volvox_carteri_88147_jgi:2.48901170768315482817,(Chromalveolata-Aureococcus_anophagefferens_16893:0.46299704618489145869,(Plantae-Ostreococcus_lucimarinus_92186:0.65128835950455521431,Chromalveolata-Phytophthora_sojae_131183:0.70796421820002231051)68:0.24663111912030225015)88:1.29031286259221977808)53:0.93860528452842373337)18:0.11803334696593120734,(((Chromalveolata-Paramecium_tetraurelia_124427262:0.00000121823947223981,Chromalveolata-Paramecium_tetraurelia_124393186:0.03857124897277592473)90:0.21417203907242679528,Chlamydia-Candidatus_Protochlamydia_46447215:4.26671113976660887346)25:0.13366462751010307897,((Chromalveolata-Paramecium_tetraurelia_124416421:0.00554515469942662691,Chromalveolata-Paramecium_tetraurelia_124411770:0.00000121823947223981)100:0.24864645618983666209,Chromalveolata-Tetrahymena_thermophila_118350222:0.20529505743830500641)63:0.08439645907271642322)38:0.38687807306049093459)5:0.00842713575275412519,(((Excavata-Jakoba_bahamiensis_109794533_3:0.44431278128517287529,Excavata-Naegleria_gruberi_45024-fgeneshHS_pg.scaffold_1000114:0.54962745797387013713)24:0.14710028044105155631,((Chromalveolata-Thalassiosira_pseudonana_2190:0.98114433124445010748,((Chromalveolata-Phytophthora_sojae_158858:0.33770456369476825609,Chromalveolata-Aureococcus_anophagefferens_65597:0.37787471851945414469)85:0.07954432360397857560,Chromalveolata-Isochrysis_galbana_ISE00003557_3:0.40176344105849065569)39:0.07553046064732503451)7:0.13526346014854057076,(Excavata-Giardia_lamblia_157434715:0.00000121823947223981,Excavata-Giardia_lamblia_159112778:0.00000121823947223981)99:0.89158333844675108359)0:0.00352602529421938661)5:0.09889030710058813178,(Chromalveolata-Karenia_brevis_Kb_AUAO_B_77772_3:0.70810630266416207945,Plantae-Volvox_carteri_90652_jgi:0.70435779615160931666)28:0.12835105389802867193)16:0.12877910973152320584,Chromalveolata-Aureococcus_anophagefferens_66530:3.33756598858181963152);

Plantae-Arabidopsis_thaliana_15219361
(Plantae-Arabidopsis_thaliana_15219361:0.22724140108382268055,(Plantae-Physcomitrella_patens_183222:0.56910705899345537251,(Bacteria-Geobacter_sp._110600188:0.55751803908851116365,(Chlamydia-Candidatus_Protochlamydia_46446484:0.82175508704329891518,Excavata-Naegleria_gruberi_52320-fgeneshHS_pg.scaffold_54000069:1.01884054710522153897)89:0.19670993057324157038)100:0.34868798350678809062)82:0.14028396556514940996,Plantae-Oryza_sativa_115444151:0.39929798345346095845);

Plantae-Arabidopsis_thaliana_15220982
(((Cyanobacteria-Synechocystis_sp._PCC_6803_16329260:0.46430878511364293004,((((Cyanobacteria-Nodularia_spumigena_CCY9414_119511388:0.07970893397303675798,(Cyanobacteria-Nostoc_punctiforme_PCC_73102_23129538:0.09597481911553663869,(Cyanobacteria-Nostoc_sp._PCC_7120_17230721:0.03242126657370179854,Cyanobacteria-Anabaena_variabilis_ATCC_29413_75911082:0.04556992333520352295)100:0.08559577375968345503)56:0.04582518318655576239)100:0.19227918331689192177,Cyanobacteria-Lyngbya_sp._PCC_8106_119485101:0.18394725451969443086)57:0.03738889381054830846,Cyanobacteria-Trichodesmium_erythraeum_IMS101_113474554:0.27177886634745318517)61:0.05806861945551508586,(Cyanobacteria-Crocosphaera_watsonii_WH_8501_67923799:0.08562000339192932241,Cyanobacteria-Cyanothece_sp._CCY0110_126657589:0.04459684422158278344)100:0.34427710788546722620)93:0.19247768789385283350)91:0.15737904808717664840,(((((Bacteria-Orientia_tsutsugamushi_148284255:1.17665242350063015131,((Bacteria-Aurantimonas_sp._90419497:0.32159846307254397191,(Bacteria-Xanthobacter_autotrophicus_154246772:0.22664851493933119153,(Bacteria-Methylobacterium_sp._149124266:0.22896315416496096362,((Bacteria-Nitrobacter_hamburgensis_92117868:0.11796573649582223209,Bacteria-Rhodopseudomonas_palustris_90423811:0.10834386705545895335)93:0.06290914007808375008,Bacteria-Bradyrhizobium_sp._146340299:0.11326271919103295116)100:0.21310216080922478321)81:0.11422355313422376255)89:0.14404337659811047856)93:0.13501964201120533748,(Bacteria-Dinoroseobacter_shibae_118736372:0.13238030844186238211,((((Bacteria-Jannaschia_sp._89054300:0.23330036664320741302,Bacteria-Roseovarius_sp._149201838:0.14303611059412973949)53:0.06013667134350776655,Bacteria-Silicibacter_sp._99080785:0.09649672738532150129)12:0.02833223840423683415,((Bacteria-alpha_proteobacterium_114768929:0.44205442827501106873,Bacteria-

Rhodobacterales_bacterium_126725345:0.19933485096928013625)21:0.06365794624165822813,Bacteria-
Roseobacter_sp._126736396:0.16459090892657213256)13:0.07089847924689525460)16:0.05430080510005571431,Bacteria-
Oceanicola_granulosus_89070361:0.17749527067667975055)25:0.05657635820321537529)92:0.25403324297546975297)97:0.
24289338741831956181)96:0.34776267231038870964,Bacteria-
Magnetococcus_sp._117925614:0.65852028903220816147)30:0.18553912892364138321,((Bacteria-
Pseudomonas_entomophila_104779701:0.41115674193627221866,((Bacteria-
Haemophilus_ducreyi_33152288:0.72784731430408089459,Bacteria-
Psychrobacter_sp._148652714:0.54234404383271939132)44:0.07911273891967920968,Bacteria-
Rickettsiella_grylli_94494115:0.69799871132032109688)48:0.10904403184195013421)94:0.29832867217959985195,Bacteria-
Syntrophobacter_fumaroxidans_116750733:1.09262017166248970312)22:0.09725885892908270325)4:0.07877321557226779625,((((((Bacteria-Actinomyces_odontolyticus_154509599:0.52131644555943346120,Bacteria-
Bifidobacterium_adolescentis_154486326:0.44938523071680258614)88:0.23943109662295219975,Bacteria-
Acidothermus_cellulolyticus_117927388:0.68627040036790365463)100:0.58345536836519429347,((((Bacteria-
Bacteroides_capillosus_154498148:0.77728944038964731256,Bacteria-
Thermoanaerobacter_ethanolicus_114845363:0.67934023627986006577)13:0.10002930889427169570,((((Bacteria-
Eubacterium_ventriosum_154482621:0.17694070680583273703,(Bacteria-
Ruminococcus_torques_153815218:0.11102587569007202339,Bacteria-
Dorea_longicatena_153854785:0.13667331582185271444)82:0.08751813602147676119)100:0.25748930989399948510,Bacteria-Halothermothrix_orenii_89209586:0.63920611787753922606)44:0.06701208181902613970,(Bacteria-
Clostridium_novyi_118445093:0.45330525554947248557,Bacteria-
Alkaliphilus_metalliredigens_150387987:0.32920065274388171650)84:0.12248323613508252383)28:0.03003539784831465650,((Bacteria-Staphylococcus_saprophyticus_73663572:0.47724592725741615817,(Bacteria-
Bacillus_pumilus_157690824:0.21983171639170220879,Bacteria-
Geobacillus_thermodenitrificans_138893714:0.20125844699894629430)89:0.04860423816576050354)53:0.05437286408791058151,(Bacteria-Listeria_monocytogenes_153200624:0.31634829673962322483,((Bacteria-
Streptococcus_pyogenes_28895107:0.17983234185073826428,Bacteria-
Lactococcus_lactis_116511486:0.24025080750535071639)100:0.24874794341054781710,(Bacteria-
Leuconostoc_mesenteroides_116618707:0.54280783548235156388,(Bacteria-
Pediococcus_pentosaceus_116492073:0.43301917016741603428,Bacteria-
Lactobacillus_johnsonii_42518291:0.78501906879698279962)79:0.15095311081167378520)58:0.16457909867997563835)21:0.07135002820110798727)22:0.05382588534624331567)53:0.21027935922107621192)32:0.12479645014853216201)10:0.03686840760326833694,(Bacteria-Thermosinus_carboxydivorans_121534378:0.42517851064985090481,Bacteria-
Symbiobacterium_thermophilum_51894384:0.41352986479072489923)57:0.13567213565499672279)21:0.06907031480050464245,((Bacteria-Desulfotomaculum_reducens_134297956:0.33782653191574074647,Bacteria-
Pelotomaculum_thermopropionicum_147676415:0.38397740462597318434)62:0.07732120945889216224,Bacteria-
Moorella_thermoacetica_83588925:0.46698617138170778196)36:0.07166883777000709888)17:0.13005656250132183183)8:0.08179764754049233255,((((Plantae-Physcomitrella_patens_161405:0.34557151043006006930,(Plantae-
Oryza_sativa_115443901:0.64284285385453332484,Plantae-
Arabidopsis_thaliana_15220982:0.14120784219439938556)97:0.31536032943207759072)100:0.49127441805984012335,(Plantae-Ostreococcus_lucimarinus_6704:0.09149890370068532175,Plantae-
Ostreococcus_tauri_5557:0.07401148692241008020)100:0.75705065552935091322)33:0.12721274816519664852,Plantae-
Volvox_carteri_103245_jgi:0.99798181289762166735)96:0.29958086998071453699,Chlamydia-
Candidatus_Protochlamydia_46446029:0.76161367418664926987)93:0.26668754865937555065)4:0.05106949991074172940,((
Chromalveolata-Aureococcus_anophagefferens_13505:1.03186874213741019979,Bacteria-
Mycoplasma_capricolum_83319298:1.06803652746106170213)28:0.33954229585278400405,Bacteria-
Syntrophomonas_wolfei_114565628:1.05181646277964313541)1:0.08375189879151631600)10:0.07844918042233962885,(Bacteria-Pelobacter_propionicus_118580905:0.51012433158654413301,Bacteria-
Geobacter_sp._110600956:0.47755656667676049665)100:0.42618389724441668731)1:0.02815886943071267690)100:0.53889177089080420036,(((Cyanobacteria-
Prochlorococcus_marinus_str._AS9601_123968460:0.02891996553954414781,(Cyanobacteria-
Prochlorococcus_marinus_str._MIT_9301_126696263:0.00759699840696371510,Cyanobacteria-
Prochlorococcus_marinus_str._MIT_9312_78779250:0.00709828203212395717)92:0.00771548217842576642)85:0.08038806905015247195,(Cyanobacteria-
Prochlorococcus_marinus_subsp._pastoris_str._CCMP1986_33861490:0.06433002198955424522,Cyanobacteria-
Prochlorococcus_marinus_str._MIT_9515_123966249:0.04247968884591959982)55:0.08456582692326682082)100:0.67185241423446251385,((((Cyanobacteria-
Prochlorococcus_marinus_str._MIT_9303_124023319:0.00419201031342754469,Cyanobacteria-
Prochlorococcus_marinus_str._MIT_9313_33862894:0.00798495082911257481)100:0.17112389042348036261,((Cyanobacteria-Synechococcus_sp._RCC307_148242414:0.28657287917397888943,Cyanobacteria-
Synechococcus_sp._WH_8102_33865586:0.13781370682391869131)23:0.05787002838481640488,((Cyanobacteria-
Synechococcus_sp._CC9311_113953175:0.21320069144778641679,Cyanobacteria-
Synechococcus_sp._RS9917_87124270:0.11465264454941545558)23:0.03750946555096288521,(((Cyanobacteria-

Synechococcus_sp._WH_7805_88808454:0.08592099528890924376,Cyanobacteria-
Synechococcus_sp._WH_7803_148239702:0.00658583037345314946)100:0.16433401642401374021,Cyanobacteria-
Synechococcus_sp._RS9916_116074644:0.05435115900217706020)70:0.04472446468904963529,(Cyanobacteria-
Synechococcus_sp._BL107_116070730:0.03475489760881422807,Cyanobacteria-
Synechococcus_sp._CC9902_78184856:0.01198638530631938176)98:0.24855070943188112276)49:0.04440481189177405974
)22:0.03378758002816931866)29:0.09333855000504862054)35:0.15862695047585467778,(Cyanobacteria-
Prochlorococcus_marinus_subsp._marinus_str._CCMP1375_33240213:0.29136802645905340858,Cyanobacteria-
Prochlorococcus_marinus_str._MIT_9211_84518233:0.27144954071603794032)45:0.12491062139589716173)22:0.174868662
35046086282,(Cyanobacteria-Prochlorococcus_marinus_str._NATL1A_124025654:0.00000121823947223981,Cyanobacteria-
Prochlorococcus_marinus_str._NATL2A_72382118:0.02769799166177893540)100:0.48758692833921551957)38:0.150143579
48858952918)100:0.33257247840305931286)78:0.14209265610152219117)100:0.41882730631502601559,Cyanobacteria-
Synechococcus_elongatus_PCC_7942_81299122:0.00000121823947223981,Cyanobacteria-
Synechococcus_elongatus_PCC_6301_56751211:0.00000121823947223981);

Plantae-Arabidopsis_thaliana_15223438
((Bacteria-Dinoroseobacter_shibae_118737397:0.45543936494912928836,(Bacteria-
Brucella_ovis_148560117:0.02710065679074320777,Bacteria-
Ochrobactrum_anthropi_153008316:0.03331410502493362125)93:0.08265819334262312912)38:0.03131131661748781658,((B
acteria-Rhizobium_leguminosarum_116253043:0.15123432228419436685,(Bacteria-
Agrobacterium_tumefaciens_15887822:0.21680463142141151445,Bacteria-
Sinorhizobium_medicae_150397279:0.23962959878961717641)48:0.04443220786478007528)88:0.06678121104095559313,(((
Bacteria-Yersinia_enterocolitica_123443069:0.37715164271239798310,(Bacteria-
Enterobacter_sakazakii_156933054:0.19414869639000656432,Bacteria-
Serratia_proteamaculans_157369252:0.09766310826658038124)82:0.05258590289716134902)28:0.05913168208968864331,(B
acteria-Flavobacterium_johnsoniae_146300119:0.41905823170522610299,(Bacteria-
Actinobacillus_pleuropneumoniae_126208921:0.10605829798926413865,(Bacteria-
Acinetobacter_baumannii_126643256:0.20620818122237180581,Bacteria-
Campylobacter_concisus_157165442:0.18279900500118537177)33:0.03957864864904749730)40:0.04467183044024773675)1
0:0.02512002725462915767)4:0.03127848961013436624,(((((Bacteria-
Chromobacterium_violaceum_34498319:0.10922270915571131999,(Bacteria-
Aeromonas_salmonicida_145298297:0.10829954986346933266,(Bacteria-
Janthinobacterium_sp._152980783:0.11520113233578692380,(Bacteria-
Methylobacillus_flagellatus_91774535:0.16555672112043598454,(Bacteria-
Vibrionales_bacterium_148977691:0.15289233219224815374,Bacteria-
Psychromonas_sp._90409060:0.18382293194807095005)29:0.04176601036358439101)20:0.04084573978934322169)15:0.034
34171972351262453)15:0.04631751851725931374)19:0.05327395639328122434,(Cyanobacteria-
Cyanothece_sp._CCY0110_126657504:0.18144261299267511189,(Cyanobacteria-
Synechocystis_sp._PCC_6803_16330455:0.27245073266934816125,Cyanobacteria-
Synechococcus_sp._WH_5701_87302992:0.35402647725992852923)52:0.11308333049020991012)73:0.150026496377601653
58)3:0.03774893264850671254,(((Bacteria-Photobacterium_profundum_90414810:0.14256094989067341627,Bacteria-
Vibrio_angustum_90579002:0.09783954335465082364)86:0.09594611797893685956,(((Bacteria-
Myxococcus_xanthus_108762920:0.15695180276306075484,Bacteria-
Frankia_alni_111220867:0.58095341020746782412)99:0.37435921362201418638,((Bacteria-
Bacteroides_caccae_153807854:0.57290690352105377858,(((Chromalveolata-
Aureococcus_anophagefferens_29268:1.08426843276093509694,(((Plantae-
Physcomitrella_patens_135286:0.00000121823947223981,Plantae-
Physcomitrella_patens_83986:0.00000121823947223981)100:0.07336777467881502868,((((((Plantae-
Oryza_sativa_115480437:0.33050698275504525325,(Plantae-Oryza_sativa_115459470:0.03064481392442429050,Plantae-
Oryza_sativa_115447423:0.03680295832555538493)58:0.01041247702820917963)37:0.03626660205126711750,(Plantae-
Oryza_sativa_115456759:0.40445625550725472497,((Plantae-Oryza_sativa_115471891:0.31203332337152367426,Plantae-
Oryza_sativa_115471887:0.02166597177773124314)29:0.00000121823947223981,Plantae-
Oryza_sativa_115471889:0.01243191533894922110)75:0.06461456536687988639)29:0.03636325639439792062)11:0.0291232
1649702720797,(((Plantae-Arabidopsis_thaliana_15233102:0.10266716642338809262,Plantae-
Arabidopsis_thaliana_15224999:0.10107491910462151896)42:0.04880974396461426612,(((Plantae-
Arabidopsis_thaliana_79314872:0.00000121823947223981,Plantae-
Arabidopsis_thaliana_15231810:0.00000121823947223981)98:0.03531560291415470482,(Plantae-
Arabidopsis_thaliana_15228095:0.00553435385150433199,Plantae-
Arabidopsis_thaliana_15228096:0.04038690293803239018)65:0.00000121823947223981)56:0.04475979126215817033,Plantae
-
Arabidopsis_thaliana_15239397:0.09090818928578363323)20:0.01813891997205947793)14:0.02196133066911864357,Plantae
-Oryza_sativa_115471893:0.06902549733041744850)3:0.01370382487441103130)18:0.03225818537569167394,(Plantae-
Oryza_sativa_115457384:0.12131783242153405811,(Plantae-

Arabidopsis_thaliana_15236267:0.06738598654501574969,Plantae-
Arabidopsis_thaliana_15227302:0.02438167728887671512)72:0.03966003946052355095)69:0.05676457210650422830)21:0.0
6678646881512532996,((((Plantae-Oryza_sativa_115447785:0.03817040221369502606,Plantae-
Oryza_sativa_115449847:0.06470806338175347228)84:0.03570466156943093161,Plantae-
Oryza_sativa_115459882:0.02359091828472030875)94:0.05439784260699126461,(((Plantae-
Arabidopsis_thaliana_42572785:0.05790068224402989544,Plantae-
Arabidopsis_thaliana_18411332:0.00000121823947223981)86:0.01662333315778075082,((Plantae-
Arabidopsis_thaliana_15233109:0.01662811186340811334,Plantae-
Arabidopsis_thaliana_15225913:0.01116067450184797701)98:0.03435350399802312993,Plantae-
Arabidopsis_thaliana_15223438:0.00560027477996302616)10:0.00000121823947223981)49:0.02797569840863609394,Plantae
-
Arabidopsis_thaliana_15236485:0.00625057139821447172)40:0.02130164441723034902)81:0.07263817655754324643,(Planta
e-Physcomitrella_patens_171662:0.15543063456149669443,(Plantae-
Physcomitrella_patens_62169:0.04778845650489971925,Plantae-
Physcomitrella_patens_166091:0.04259575737165642584)96:0.07408535810350351525)53:0.02801256135750945439)98:0.17
815610431516440415)17:0.06740141896565267354,(Plantae-
Physcomitrella_patens_202226:0.04548333316600312176,(Plantae-
Physcomitrella_patens_209703:0.03071800145050631800,(Plantae-
Physcomitrella_patens_68172:0.51794022051932997464,Plantae-
Physcomitrella_patens_196472:0.02335224880884619741)10:0.02646995199885174982)6:0.01450514808349711963)6:0.0465
4277627742928192)27:0.04347820297918023047)100:0.65638382475751388956,((((Opisthokonta-
Apis_mellifera_110758861:1.28293778200189967365,(Opisthokonta-
Apis_mellifera_66514115:0.60458269256926522317,(Opisthokonta-
Drosophila_melanogaster_45551084:0.00000121823947223981,Opisthokonta-
Drosophila_melanogaster_24652747:0.00000121823947223981)100:0.55942820925188396775)85:0.17157170745390712407)2
5:0.12654866020605498589,(Opisthokonta-Apis_mellifera_48095246:0.44381554835551162430,(Opisthokonta-
Drosophila_melanogaster_24652751:0.02680390387455394707,Opisthokonta-
Drosophila_melanogaster_19922038:0.01036119097302500534)100:0.70782842693162262293)90:0.28677565190039883136)3
7:0.08448393421013519755,((Opisthokonta-Drosophila_melanogaster_17136672:0.46700908754697778580,Opisthokonta-
Apis_mellifera_66553045:0.93977222850738617588)100:0.52003932469418723272,((((Opisthokonta-
Danio_rerio_46309487:0.24906041790707636463,Opisthokonta-
Danio_rerio_125805208:0.25915942477966619206)93:0.13058254779242839394,Opisthokonta-
Mus_musculus_6680710:0.27728622642514927366)97:0.31307634548791746232,((Opisthokonta-
Mus_musculus_6857757:0.32659649701191656446,Opisthokonta-
Mus_musculus_28201966:0.51063748536869757455)89:0.12680695053704832409,(((Opisthokonta-
Danio_rerio_125851556:0.00000121823947223981,Opisthokonta-
Danio_rerio_66472262:0.01212337449661886335)100:0.13874726065443968026,Opisthokonta-
Danio_rerio_57525964:0.07099175146883501819)100:0.20538070176807038392,Opisthokonta-
Mus_musculus_31543250:0.17599181515532211217)100:0.28841884591468996968)72:0.13446829410608096556)75:0.15620
847573509244466,(Opisthokonta-Mus_musculus_33563244:0.17397476900870564864,Opisthokonta-
Danio_rerio_54261805:0.22328845866657798247)100:0.34731305609108936716)71:0.14008326351102240448)46:0.12343963
619836123558)23:0.14094445010814232733,((Plantae-Physcomitrella_patens_144131:0.67580756450413570491,(Chlamydia-
Candidatus_Protochlamydia_46446430:1.03737868734064120346,(((Plantae-
Oryza_sativa_115435484:0.51208455230636995559,Plantae-
Arabidopsis_thaliana_15225195:0.31236544037769659043)99:0.18071589231838533496,((((Plantae-
Oryza_sativa_115459542:0.44074340600210676344,Plantae-
Oryza_sativa_115482680:0.23405590110231946910)92:0.13245419795701918186,(Plantae-
Arabidopsis_thaliana_15219403:0.13457478153045654423,(Plantae-
Arabidopsis_thaliana_30685536:0.11116270525731004881,Plantae-
Arabidopsis_thaliana_15220848:0.00000121823947223981)99:0.08919961325602124291)95:0.19185806449796477535)98:0.3
1584597377744783042,((Plantae-Arabidopsis_thaliana_15234189:0.18090721403202203454,Plantae-
Oryza_sativa_115442565:0.19657356039598203323)79:0.11669723921453595272,(Plantae-
Oryza_sativa_115450711:0.16928016900364645236,(Plantae-
Arabidopsis_thaliana_15228041:0.06336061086324908109,Plantae-
Arabidopsis_thaliana_15231569:0.13511645840212216374)92:0.07871329322630131464)32:0.02635785432365100531)80:0.1
3887122961630754547)73:0.11778444628444910414,((Plantae-
Arabidopsis_thaliana_15232798:0.67274903133281582779,Plantae-
Oryza_sativa_115459786:0.54309388839247152436)97:0.36766541709128880422,((Plantae-
Oryza_sativa_115467914:0.26823442091989574410,Plantae-
Arabidopsis_thaliana_15233320:0.09746239908386673811)77:0.06649339795999324731,((Plantae-
Arabidopsis_thaliana_15236043:0.02050956818651594391,Plantae-
Arabidopsis_thaliana_15238100:0.08357275973502495403)100:0.15327387107737847072,(Plantae-

Oryza_sativa_115459788:0.1940329235975269683 8,Plantae-
Oryza_sativa_115447705:0.0280994600628471508 1)97:0.15361254868673956953)72:0.0491022918058754 6982)88:0.0799526
2554793551579)83:0.10591199732627119834)71:0.09798581089229722874)98:0.30021407460575222359,(Plantae-
Physcomitrella_patens_73809:0.08190637138944972806,((Plantae-
Physcomitrella_patens_219971:0.10476787653519900601,Plantae-
Physcomitrella_patens_214518:0.03333336977513678273)68:0.03336645995941282111,Plantae-
Physcomitrella_patens_191107:0.05833369761817410598)42:0.01371395450340228903)100:0.34969785842662071129)69:0.2
2739695818823962847)80:0.12667093955573163755)81:0.17147615637199686844,((Amoebozoa-
Dictyostelium_discoideum_1562532:0.79648422255363304778,Amoebozoa-
Dictyostelium_discoideum_6012182:0.74697282948005228054)98:0.43763608421654165426,(((Opisthokonta-
Ustilago_maydis_71003379:0.74980962331004030741,(Opisthokonta-
Cryptococcus_neoformans_58264756:0.55003806899605345304,Opisthokonta-Sporobolomyces_roseus_27302-
fgenesh1_pg.C_scaffold_5000191:0.47544103446142682579)73:0.17856983793837807872)40:0.05697931958968334432,Opist
hokonta-Saccharomyces_cerevisiae_6325450:1.08126784378930995700)48:0.13648771011408772358,Opisthokonta-
Magnaporthe_grisea_39944302:0.45539480367554824314)100:0.65377959544718711182)56:0.24868113085135834517)13:0.0
6075983657636981061)18:0.12301883239998852304)13:0.14029316561775703742)23:0.28678201169989436803,Chromalveo
lata-Phaeodactylum_tricornutum_42538:1.50175620514090990554)95:0.38366670819670917192,((Bacteria-
Streptococcus_pneumoniae_111657305:0.82051428956947958238,Bacteria-
Bacillus_anthracis_47530041:0.26798585836498539647)58:0.12816639138685978483,Bacteria-
Staphylococcus_saprophyticus_73663798:0.62196811460002277006)77:0.15877000717757530635)33:0.141759755449578056
52)22:0.07948342477377141890,(((Bacteria-Xanthobacter_autotrophicus_154246262:0.39420359522356573923,Bacteria-
Mesorhizobium_sp._110632827:0.50202400082818821048)59:0.13945823869462231626,(Bacteria-
Hyphomonas_neptunium_114798833:0.36173289950253234570,(Bacteria-
Rhodobacter_sphaeroides_146277087:0.51646927450071900001,Bacteria-
Roseovarius_sp._149203311:0.30550433200396698297)80:0.11604493303740882892)79:0.15961740626323361880)26:0.0892
1817383190408512,Bacteria-
unidentified_eubacterium_149372322:0.54649819913643726821)5:0.01160238588108256259)50:0.18124917293447070921)72
:0.26629921882135820788,(Bacteria-Algoriphagus_sp._126645866:0.19120509438346669495,Bacteria-
Methylobacterium_chloromethanicum_156449619:0.46644363372522273936)14:0.03720939300264046457)1:0.048962068237
63149529)8:0.03817097852702884542,((Bacteria-Geobacter_bemidjiensis_145621257:0.13530161064812976091,(Bacteria-
marine_gamma_90417017:0.19836615257702247539,(Bacteria-
Shewanella_woodyi_118074177:0.17531841700521039185,Bacteria-
Syntrophobacter_fumaroxidans_116749465:0.23410619218754480375)31:0.04422015561592649441)11:0.02976242475567415
433)8:0.04642965230398230642,((Cyanobacteria-
Gloeobacter_violaceus_PCC_7421_37519936:0.21312300507329151600,(Bacteria-
Acidobacteria_bacterium_94967157:0.22913802897782453716,Cyanobacteria-
Lyngbya_sp._PCC_8106_119486832:0.24744128637058981912)21:0.05782331133851623117)5:0.03364561597817449651,((B
acteria-Bradyrhizobium_sp._148252484:0.32694423939575528726,Bacteria-
Pseudomonas_putida_148546824:0.15480378405488165461)50:0.10226478093518462376,(Bacteria-
Anaeromyxobacter_dehalogenans_86160023:0.21940655574706502406,Bacteria-
Burkholderia_vietnamiensis_134294322:0.18023786896558249837)79:0.09938368097748236096)11:0.068904541894851623 0
6)6:0.05752530227984026245)4:0.01459545007050284428)0:0.03821741579220469531)0:0.06089105527525352085,(Bacteria
-Ralstonia_metallidurans_94314624:0.46085822310396817869,Bacteria-
Sphingopyxis_alaskensis_103485731:0.27187567497001230432)51:0.14071996614568685335)0:0.04556133855508675418,((B
acteria-Clavibacter_michiganensis_148271568:0.75880800215275423515,Bacteria-
Delftia_acidovorans_118730753:0.27375293265871292192)9:0.11922468432753655088,Bacteria-
Bordetella_bronchiseptica_33601128:0.32405348173076531371)2:0.09150981108830939792)2:0.04180198816745388235)62:0
.10799213929720964666)68:0.08562793606991055129,Bacteria-
Sphingomonas_wittichii_148552957:0.16720048009326676786);

Plantae-Arabidopsis_thaliana_15225451
(Plantae-Cyanidioschyzon_merolae_CMR075C:0.75655030811775403343,(((Plantae-
Chlamydomonas_reinhardtii_151368:0.09396607105999751908,Plantae-
Volvox_carteri_55127_jgi:0.18334992994923318688)100:0.54054179698876581028,(((Plantae-
Ostreococcus_tauri_15605:0.07230586546879974597,Plantae-
Ostreococcus_lucimarinus_14357:0.07046251382067175717)93:0.07945507966903302133,Plantae-
Ostreococcus_lucimarinus_18839:0.24280882928795149978)100:0.36972839276298569144,((((Plantae-
Physcomitrella_patens_166362:0.05163917940654866312,(Plantae-
Physcomitrella_patens_163131:0.03718570693248270803,(Plantae-
Physcomitrella_patens_60904:0.11842947210322359031,Plantae-
Physcomitrella_patens_165615:0.02144787825170767215)90:0.03956334909585830506)98:0.05057687066712956980)100:0.1
0815085992940498549,((Plantae-Physcomitrella_patens_108048:0.05588345521738639909,Plantae-

Physcomitrella_patens_56808:0.02408349897329282313)100:0.05817922218227255388,(Plantae-
Physcomitrella_patens_179857:0.06773380587007250819,(Plantae-
Physcomitrella_patens_55001:0.03440271899973951913,Plantae-
Physcomitrella_patens_55414:0.02695259123675286875)55:0.02038545844841998037)98:0.05380059283971511119)50:0.022
32289043092217443)99:0.07007837459164120708,(Plantae-
Arabidopsis_thaliana_15234745:0.08550817030797305818,(Plantae-
Oryza_sativa_115449159:0.02826283304528044715,Plantae-
Oryza_sativa_115466832:0.17663433507011278012)98:0.06467541975768859031)99:0.09856667712007707860)71:0.0356748
7600321434665,((Plantae-Oryza_sativa_115451921:0.25448006728295996526,(Plantae-
Arabidopsis_thaliana_15234486:0.15851185665657899215,(Plantae-
Arabidopsis_thaliana_15225451:0.08924260207487165475,(Plantae-
Arabidopsis_thaliana_15217591:0.04605704846155125182,Plantae-
Arabidopsis_thaliana_15236712:0.01272330813821974903)85:0.02280977715461360647)60:0.03868746097547885038)44:0.0
3147689684599269677)39:0.03458417639861605802,((Plantae-Oryza_sativa_115477399:0.08484407697455917385,Plantae-
Oryza_sativa_115480003:0.06007455367795605516)100:0.26602828644093179378,Plantae-
Arabidopsis_thaliana_15229524:0.13503950947701551244)82:0.10536793834986722673)73:0.03659673126754418859)99:0.2
7809097949902567004)62:0.12060809115444494355)96:0.31477051756315060915,((Excavata-Naegleria_gruberi_81312-
estExt_fgeneshNG_pg.C_560068:0.92399322545860651523,(Chromalveolata-
Aureococcus_anophagefferens_19538:0.89950074404000668338,(Chromalveolata-
Aureococcus_anophagefferens_2757:0.50887179716609420854,Chromalveolata-
Aureococcus_anophagefferens_18438:0.21718967825490151369)98:0.28311939343808478764)81:0.23023045238722697481)7
7:0.23423510634591082624,((Cyanobacteria-
Crocosphaera_watsonii_WH_8501_67925414:0.34623528489528965224,(Bacteria-
Clostridium_thermocellum_125972751:0.37042834723823664378,(Bacteria-
Desulfotomaculum_reducens_134300858:0.50770747757838130543,Bacteria-
Bacillus_weihenstephanensis_89206665:0.31748064351379429171)90:0.14351649032123067751)56:0.08371211883232830553
)17:0.05731957498190562439,(Bacteria-Thiomicrospira_crunogena_78485850:0.39088659909542394777,(Bacteria-
Geobacter_uraniumreducens_148264642:0.30038773600867585412,((Cyanobacteria-
Gloeobacter_violaceus_PCC_7421_37520637:0.40020575187769802383,(Bacteria-
Halorhodospira_halophila_121998958:0.38543830466929313028,Bacteria-
Candidatus_Desulfococcus_121541242:0.30461526924944726247)28:0.08318715980229385298)6:0.03626181605773991234,(
((Bacteria-Syntrophobacter_fumaroxidans_116747629:0.38238333466811996741,Bacteria-
Coxiella_burnetii_154706468:0.41537821582679324006)18:0.06267470360340085533,((Bacteria-
Bradyrhizobium_sp._146342016:0.19023200284793576054,Bacteria-
Rhodopseudomonas_palustris_90422944:0.39104951373673008108)98:0.19395517997742756089,((Bacteria-
Anaeromyxobacter_dehalogenans_86156650:0.27949726475400377135,(Cyanobacteria-
Synechococcus_sp._RCC307_148241284:0.29031142616505201115,(Cyanobacteria-
Synechococcus_sp._WH_5701_87300564:0.21287722175071527309,(Cyanobacteria-
Synechococcus_sp._WH_7805_88808092:0.06856919373534582640,(Cyanobacteria-
Synechococcus_sp._RS9916_116075546:0.06165972803271677766,((((((((Cyanobacteria-
Prochlorococcus_marinus_str._NATL2A_72383517:0.00000121823947223981,Cyanobacteria-
Prochlorococcus_marinus_str._NATL1A_124025108:0.00683081831124306756)100:0.34933171232518783755,(Cyanobacteria
-Prochlorococcus_marinus_str._MIT_9301_126696737:0.39946617307379272965,((Cyanobacteria-
Prochlorococcus_marinus_str._MIT_9312_78779696:0.44436067567483078511,Cyanobacteria-
Prochlorococcus_marinus_str._MIT_9515_123966604:0.61569939591245925747)36:0.14890591795420374255,(Cyanobacteria
-Prochlorococcus_marinus_str._AS9601_123968930:0.52322720937078825987,Cyanobacteria-
Prochlorococcus_marinus_subsp._pastoris_str._CCMP1986_33861766:0.50575513694087670391)38:0.08102146382191066643
)12:0.06222414199012062802)25:0.13021696025565229760)23:0.07988649866337932393,Cyanobacteria-
Prochlorococcus_marinus_subsp._marinus_str._CCMP1375_33240764:0.29191143022675081209)20:0.0827095961981629962
4,Cyanobacteria-
Prochlorococcus_marinus_str._MIT_9211_84517646:0.27744324184811963718)50:0.18120772418122357839,(Cyanobacteria-
Prochlorococcus_marinus_str._MIT_9303_124024227:0.01110507930273704064,Cyanobacteria-
Prochlorococcus_marinus_str._MIT_9313_33864169:0.01836329384330652806)100:0.09251937266615284372)15:0.09038702
589606628646,Cyanobacteria-
Synechococcus_sp._RS9917_87123083:0.07434622387311760894)6:0.02014310090271436912,((Cyanobacteria-
Synechococcus_sp._WH_8102_33864736:0.08079292999025577959,Cyanobacteria-
Synechococcus_sp._CC9605_78211743:0.13644422009790208028)42:0.01459000523607242687,(Cyanobacteria-
Synechococcus_sp._CC9902_78183807:0.03487761462695310871,Cyanobacteria-
Synechococcus_sp._BL107_116071583:0.02246645308239939021)100:0.05083456022607100305)75:0.0405216091843497230
9)5:0.01898441525916991918,(Cyanobacteria-
Synechococcus_sp._WH_7803_148238581:0.09022995675162799600,Cyanobacteria-
Synechococcus_sp._CC9311_113953729:0.09546348003772046897)20:0.01310444790887487984)13:0.01990229828358382253

8)12:0.02671392834391728838)27:0.10334405002144141095)20:0.09310466583476464941)87:0.25502815344747908899)24:
0.04295397651536272260,(Bacteria-Novosphingobium_aromaticivorans_87201163:0.43303969870474245951,Bacteria-
Parvibaculum_lavamentivorans_154252370:0.44344648674526321308)94:0.24418675125566785544)18:0.04064526143963442
806)15:0.06653947057941171639)1:0.03253028610138862520,(((Bacteria-
Pseudomonas_fluorescens_77460292:0.36527243135060755153,(Bacteria-
Mariprofundus_ferrooxydans_114777522:0.22443703358939071091,(Bacteria-
Chlorobium_phaeobacteroides_119357538:0.13194128076313393905,(Bacteria-
Prosthecochloris_vibrioformis_145220024:0.05492459522572248898,Bacteria-
Pelodictyon_luteolum_78187259:0.09637872529465471572)89:0.06739159372659862812)98:0.09525647222195428310)19:0.0
4529874542300196816)15:0.05267309574086315171,(((Bacteria-
Hahella_chejuensis_83647583:0.15360595756063849526,Bacteria-
Marinobacter_aquaeolei_120554607:0.11417205536003179056)81:0.06417128831105295417,(((Bacteria-
Nitrosomonas_europaea_30250207:0.28859284227516623922,Bacteria-
Magnetococcus_sp._117925406:0.28190263177250474147)40:0.08635585774508006129,(Bacteria-
Nitrosococcus_oceani_77166096:0.29915646896743763872,Bacteria-
delta_proteobacterium_94264686:0.21963677804081790934)13:0.02121193104487383713)6:0.03065006134714570729,Bacteri
a-
Methylococcus_capsulatus_53803405:0.24555088214390788370)20:0.04627764349201829441)31:0.09372707117112752395,(
Cyanobacteria-Thermosynechococcus_elongatus_BP-1_22298743:0.36985874578616934061,Bacteria-
Alkalilimnicola_ehrlichei_114321825:0.42648361920100308353)5:0.04938410720533582821)18:0.02925500531928372505)8:
0.07065521190530243967,(((Bacteria-Desulfotalea_psychrophila_51246568:0.34485753038867150710,Bacteria-
Saccharophagus_degradans_90020265:0.32297576408859379749)20:0.05299519505004219450,(((Cyanobacteria-
Nostoc_punctiforme_PCC_73102_23128940:0.22733957166637411285,Cyanobacteria-
Lyngbya_sp._PCC_8106_119493841:0.28808978614640401439)11:0.03485579786796696306,Cyanobacteria-
Nodularia_spumigena_CCY9414_119509616:0.17674198148205477743)34:0.06707141239036121250,(Bacteria-
Lawsonia_intracellularis_94987022:0.37749734222345759926,Bacteria-
Desulfovibrio_desulfuricans_78355080:0.16811232240252174663)96:0.18635975691636127993)1:0.02548705601594410813)2
:0.04458349913148351717,(((Bacteria-Azoarcus_sp._119897143:0.13990488258923913789,(Bacteria-
Dechloromonas_aromatica_71909541:0.12634803462484089920,Bacteria-
Rhodoferax_ferrireducens_89899467:0.12777966336574261841)99:0.06254873444769794288)100:0.13812259729500589445,(
((Bacteria-Klebsiella_pneumoniae_152971031:0.15059088036894910378,((Bacteria-
Enterobacter_sakazakii_156933709:0.14545382047157079386,Bacteria-
Serratia_proteamaculans_157370947:0.10772685075224540385)92:0.07447120364546354343,Bacteria-
Sodalis_glossinidius_85059347:0.21103550503774637370)56:0.02964774132007997123)87:0.06814084738397158114,(((Bacte
ria-Moritella_sp._149909354:0.20836821895979476538,(Bacteria-
Photobacterium_sp._89076363:0.01925794219895904727,Bacteria-
Vibrio_angustum_90580916:0.00529882165787510465)100:0.06549195211969813246)100:0.08733423346522023456,(Bacteri
a-Aeromonas_hydrophila_117618758:0.25875736193677001395,Bacteria-
Shewanella_oneidensis_24376158:0.21465314106810950978)53:0.04173920291454157050)41:0.02260254014206589807,Bact
eria-
Psychromonas_sp._90407559:0.43873181533993310666)80:0.06598658809849219931)95:0.14804615719984981492,(Bacteria-
Oceanospirillum_sp._89094083:0.21797662989180116577,(Bacteria-
Alteromonadales_bacterium_119469403:0.08224800641862654860,Bacteria-
Pseudoalteromonas_tunicata_88860035:0.11220322225709714048)100:0.28157430234080765841)99:0.1667890184164835809
5)55:0.10645306114974120715)7:0.03773457029339739865,((Bacteria-
Beggiatoa_sp._153868858:0.26534805141408485119,Bacteria-
Pelobacter_carbinolicus_77919068:0.18131435884828692173)27:0.02916414108155028148,Bacteria-
Syntrophus_aciditrophicus_85858261:0.24785265543665216392)9:0.02914400248076419453)0:0.02879261721837854393)0:0.
03691161261496725410)0:0.00000121823947223981)0:0.01536155649035269785)2:0.05281258726356598959)2:0.060553634
67615998418)5:0.04504214017031179379)99:0.29158315875490042890)27:0.07431156872346662834)46:0.068110877894213
13432,Chlamydia-Candidatus_Protochlamydia_46445713:0.57115155133578043856);

Plantae-Arabidopsis_thaliana_15232565
(Bacteria-Moritella_sp._149910598:0.28853564694837896543,(((Bacteria-
Vibrionales_bacterium_148977769:0.14157280622193116337,Bacteria-
Vibrio_sp._116185625:0.09485735204610550031)100:0.18338260116715976444,Bacteria-
Photobacterium_sp._89075262:0.28200471588824838065)87:0.10347199247257279908,(((((Bacteria-
Hahella_chejuensis_83647978:0.30164389604154862035,(Bacteria-
Marinobacter_sp._126668780:0.22006037218713481174,Bacteria-
marine_gamma_119477364:0.25295106905191683255)26:0.07769221376557759740)7:0.04083422135920387885,((Bacteria-
Chromohalobacter_salexigens_92114343:0.39240478382016485970,(Bacteria-
Oceanospirillum_sp._89093952:0.23354318536244902949,Bacteria-

Oceanobacter_sp._94501782:0.31866450980148453009)39:0.05858908494480694062)8:0.03524695943612595611,(Bacteria-Saccharophagus_degradans_90022816:0.43267130923773960971,Bacteria-Pseudomonas_fluorescens_70728302:0.23056169941999424267)50:0.07346145524285385697)5:0.05022900418008610646)26:0.05967389089222702170,(Bacteria-Coxiella_burnetii_153208981:0.34038439128454989202,Bacteria-Legionella_pneumophila_148360514:0.35863995513135871196)69:0.12180186770437934074)6:0.03528813700677958104,(((Bacteria-Granulibacter_bethesdensis_114327364:0.67006411406319488222,((Bacteria-Anaeromyxobacter_sp._153003237:0.79629294126242344820,(Bacteria-Halothermothrix_orenii_89211044:0.51902966978210596238,(Bacteria-Geobacter_sp._110600646:0.45986977862481226431,Bacteria-Thermosinus_carboxydivorans_121534986:0.22130518662279710651)36:0.07508304166807588942)56:0.07835143184958565099)10:0.05171650635010232450,(((Bacteria-Sulfurovum_sp._152993936:0.28493193464934185588,Bacteria-Caminibacter_mediatlanticus_149194802:0.30206659404698499305)100:0.28753517581314474993,Bacteria-Marinomonas_sp._152995154:0.60595885416548878055)74:0.13189773672265919791,(((Bacteria-Prosthecochloris_vibrioformis_145220332:0.12654385497090139823,Bacteria-Pelodictyon_luteolum_78187601:0.11824005945765125869)100:0.14570135511296267161,Bacteria-Chlorobium_chlorochromatii_78188445:0.18723885059875497450)100:0.44226203859302287702,((Bacteria-Hydrogenobaculum_sp._156718597:0.50055544701623788661,Bacteria-Leptospira_borgpetersenii_116329828:0.68564254769657773103)50:0.23722590800924855947,(((Cyanobacteria-Synechococcus_elongatus_PCC_7942_81301100:0.00000121823947223981,Cyanobacteria-Synechococcus_elongatus_PCC_6301_56751818:0.00000121823947223981)100:0.40323605124573497216,(Cyanobacteria-Synechococcus_sp._RCC307_148241383:0.27272151046453618761,((((Cyanobacteria-Synechococcus_sp._RS9916_116074469:0.09499079371051809273,Cyanobacteria-Synechococcus_sp._RS9917_87123156:0.02929988994670923441)38:0.01640169019825607824,(Cyanobacteria-Synechococcus_sp._WH_7803_148238574:0.06238333206185411178,Cyanobacteria-Synechococcus_sp._WH_7805_88808099:0.02953094579171497772)91:0.06216322306322596969)73:0.0646451995723560934 9,((Cyanobacteria-Synechococcus_sp._WH_8102_33864723:0.04748325150345399787,(((Cyanobacteria-Synechococcus_sp._CC9902_78183795:0.02665986687693360641,Cyanobacteria-Synechococcus_sp._BL107_116071595:0.02580930928364224414)99:0.05849132943025232845,Cyanobacteria-Synechococcus_sp._CC9605_78211735:0.07544847618991998295)52:0.01662432352857294571)96:0.05778725729434109476,(Cyanobacteria-Prochlorococcus_marinus_str._MIT_9313_33864177:0.00643531627983267348,Cyanobacteria-Prochlorococcus_marinus_str._MIT_9303_124024239:0.00310282836696896321)99:0.12424993435589475055)76:0.02684355112297439477)33:0.07391184714678515622,Cyanobacteria-Synechococcus_sp._WH_5701_87301993:0.16798880490446072011)43:0.06897628867067202851)100:0.48469408760375171097)100:0.22214162805206744133,((((Chlamydia-Chlamydophila_felis_89898595:0.05204019792079444401,(Chlamydia-Chlamydophila_caviae_29839986:0.06257284527090384296,Chlamydia-Chlamydophila_abortus_62184858:0.06299270616582014481)64:0.02000345428018934779)100:0.17172924812152076668,(Chlamydia-Chlamydia_muridarum_15835294:0.06531303695538073772,(Chlamydia-Chlamydia_trachomatis_76789130:0.00000121823947223981,Chlamydia-Chlamydia_trachomatis_15605124:0.00000121823947223981)100:0.07661473825017110773)100:0.21789225062589118176)88:0.10808676432144624646,(Chlamydia-Chlamydophila_pneumoniae_15836057:0.00000121823947223981,(Chlamydia-Chlamydophila_pneumoniae_16752515:0.00000121823947223981,(Chlamydia-Chlamydophila_pneumoniae_33241878:0.00000121823947223981,Chlamydia-Chlamydophila_pneumoniae_15618437:0.00000121823947223981)26:0.00000121823947223981)19:0.00000121823947223981)100:0.30255550719396839821)100:0.68833798446102001378,(Chlamydia-Candidatus_Protochlamydia_46447416:0.47181785095741257674,((Plantae-Arabidopsis_thaliana_15232565:0.18440154043059778233,Plantae-Oryza_sativa_115444351:0.29061050813060146369)100:0.18777329477161741611,(Plantae-Physcomitrella_patens_140682:0.26607877962051534881,Plantae-Physcomitrella_patens_149317:0.28533900915786880592)71:0.09314595109915216331)100:0.51876034939207782415)64:0.0600984742331514 9012)100:0.48486471067213282593)63:0.12776459145156571218)23:0.04688066202726002413)18:0.10606489770929783401)4:0.04098732214385593375)14:0.02904958735832310554)22:0.09008802280546428232,((Bacteria-Acinetobacter_baumannii_126641282:0.41310317725887502061,Bacteria-Francisella_tularensis_118497807:0.49144808278183471639)72:0.14817703664669346075,Bacteria-Neisseria_meningitidis_121635512:0.54812194541615055776)8:0.11877130910893039795)1:0.04374028240398702971,((Bacteria-Nitrosococcus_oceani_77166240:0.29459969871940278052,(Bacteria-Alkalilimnicola_ehrlichei_114321374:0.19739986401022402429,Bacteria-Halorhodospira_halophila_121998900:0.17945116270718100560)81:0.05365773201532186149)65:0.07782614744630945991,((Bacteria-Xanthomonas_campestris_78048669:0.10731317030584686112,Bacteria-Xylella_fastidiosa_28198550:0.14039687391370286873)100:0.24706066935140519325,((Bacteria-Chromobacterium_violaceum_34498782:0.35453934977066076106,Bacteria-Delftia_acidovorans_118731176:0.41079297448366847156)10:0.10415616843557055915,((Bacteria-Dechloromonas_aromatica_71909030:0.26131421488567246891,Bacteria-

Azoarcus_sp._56476147:0.26275092167903080220)27:0.05415957912891224135,((Bacteria-
Klebsiella_pneumoniae_152971565:0.89639734755099420571,Bacteria-
Limnobacter_sp._149925780:0.36866349834456119128)10:0.07727059715245490623,((Bacteria-
Bordetella_bronchiseptica_33603475:0.36241718802832390001,(Bacteria-
Ralstonia_eutropha_113866420:0.18376790352461389033,(Bacteria-
Janthinobacterium_sp._152980996:0.09652131704537683654,Bacteria-
Herminiimonas_arsenicoxydans_134096259:0.05426181985011926251)100:0.17855271346553935885)84:0.076386402675271
91385)39:0.07421121596193228087,(Bacteria-Polynucleobacter_sp._145590086:0.40419869577074823974,Bacteria-
Burkholderia_phytofirmans_118044264:0.22822429014604730946)70:0.04160653213671251793)29:0.05578666395744757284
)2:0.02651148229745446672)9:0.08137854642099520697)5:0.11222600721317033123)3:0.06119089944118411006)1:0.08344
787537782148712)9:0.06583527725134935560)83:0.13637150083598753580,(Bacteria-
Alteromonadales_bacterium_119469170:0.17808584670714008857,Bacteria-
Pseudoalteromonas_tunicata_88861159:0.13267565438210107698)100:0.17325534877431028602)61:0.0517471388847947425
0)64:0.05521782630084864224,Bacteria-Shewanella_sediminis_157373870:0.33437382757111300258);

Plantae-Arabidopsis_thaliana_15235511
(Chromalveolata-Phytophthora_sojae_111707:0.01246258153784937303,(((Plantae-
Ostreococcus_lucimarinus_203:0.66501089057567142060,((((Cyanobacteria-
Anabaena_variabilis_ATCC_29413_75907770:0.08293179511720584485,Cyanobacteria-
Nostoc_punctiforme_PCC_73102_23130092:0.08748013539878335409)100:0.36549511426518793700,((Plantae-
Physcomitrella_patens_215914:0.31930773545075225028,Plantae-
Volvox_carteri_64450_jgi:0.43041335985057960212)96:0.12024428047741932135,((Plantae-
Ostreococcus_tauri_18425:0.10999490955319916885,Plantae-
Ostreococcus_lucimarinus_611:0.05015803625092930124)100:0.53940038481901431133,(Plantae-
Physcomitrella_patens_142921:0.29594698076783437779,(Plantae-
Oryza_sativa_115451097:0.22522550656229547994,(Plantae-
Arabidopsis_thaliana_79328347:0.01622880047240846113,Plantae-
Arabidopsis_thaliana_42570031:0.00247487584770720572)100:0.18877211520183720395)99:0.14447973707578287694)100:0
.17548666905379647907)97:0.13045635005323752420)100:0.23405480619709145840)100:0.27630975285531694796,((((Bact
eria-Nocardioides_sp._119715897:0.53525063619944635018,Bacteria-
Oenococcus_oeni_116490441:0.70409374072883346152)100:0.30065308188737432937,((((Bacteria-
Serratia_proteamaculans_157368458:0.32601615499970931777,Bacteria-
Vibrio_cholerae_153824990:0.32392839774184611912)100:0.50115005214781049858,Bacteria-
Chloroflexus_aggregans_118045776:0.54282937098427541667)67:0.09478098849850900842,((((Bacteria-
Desulfotomaculum_reducens_134299385:0.38869857462293161410,(Cyanobacteria-
Nostoc_sp._PCC_7120_17158758:0.07635809319327882538,Cyanobacteria-
Anabaena_variabilis_ATCC_29413_75907348:0.13992794047212525621)100:0.56861376750127612389)41:0.0805314914911
7512506,((Bacteria-Geobacillus_thermodenitrificans_138894309:0.27900242915383333120,((Bacteria-
Staphylococcus_aureus_49482927:0.21712288564999540630,Bacteria-
Lactococcus_lactis_146322228:0.29448068053190262772)100:0.11186444232909042995,(Bacteria-
Bacillus_coagulans_124519839:0.09359099418284345939,Bacteria-
Streptococcus_agalactiae_22537407:0.11069626203553814547)100:0.10829180080052880353)100:0.11625826290158078336)
100:0.21426267682299407480,(Bacteria-Francisella_tularensis_118497001:0.67768491416153264773,Chlamydia-
Candidatus_Protochlamydia_46445921:0.86730352020779155620)48:0.11976013667268184393)28:0.04560058721148928240)
44:0.10441741329319596809,(Bacteria-Ralstonia_solanacearum_17548540:0.52512376425299145399,(Bacteria-
Methylobacillus_flagellatus_91776900:0.36425888835202163696,(Cyanobacteria-
Synechococcus_sp._WH_5701_87302472:0.32587412148101224663,(Bacteria-
Polaromonas_naphthalenivorans_121606885:0.18053833255604298524,Bacteria-
Comamonas_testosteroni_118053356:0.48309743024168816383)86:0.09395749958872084973)100:0.20503908992316313697)
44:0.07943611482220570841)100:0.31987708180722990159)47:0.06628772714372969943,((Chromalveolata-
Phaeodactylum_tricornutum_52367:0.40994071491743766522,Chromalveolata-
Thalassiosira_pseudonana_261657:0.39015174918169043261)100:0.66162223123486707532,(Bacteria-
Desulfitobacterium_hafniense_89897147:0.42488788173308844609,((Cyanobacteria-
Synechocystis_sp._PCC_6803_16331908:0.19372149201259544760,(Cyanobacteria-
Lyngbya_sp._PCC_8106_119486614:0.17914553799363841513,Cyanobacteria-
Nostoc_punctiforme_PCC_73102_23126943:0.22427220494381835958)78:0.05497321958793216501)100:0.195433350557620
95509,(Bacteria-Treponema_denticola_42526892:0.82764264838565038129,((Bacteria-
Petrotoga_mobilis_145622585:0.01037869665110825387,Bacteria-
Caldicellulosiruptor_saccharolyticus_146295535:0.00758375433760161057)100:0.23229187730570011183,(((Bacteria-
Ruminococcus_obeum_153809757:0.19833087971182533638,Bacteria-
Dorea_longicatena_153855119:0.11455296129776280922)100:0.25565815038445915963,Bacteria-
Bacteroides_capillosus_154500246:0.24525056386213930915)92:0.09235197551925514170,Bacteria-

Clostridium_cellulolyticum_118726401:0.26188165338689151085)99:0.14954241012359045793)98:0.09350993789515349430
)27:0.06299613466729794831)20:0.09221563711270996944)100:0.54816265294657828822)28:0.09828108444118859033)12:
0.02294942915166439668)59:0.07561065957926250969,((Bacteria-
Hyphomonas_neptunium_114799122:0.29028401484908145092,((Bacteria-
Mycobacterium_sp._119855063:0.35498514693541399767,Bacteria-
Caulobacter_sp._113933097:0.24307899947494299164)36:0.05543955924722877154,((Bacteria-
Corynebacterium_glutamicum_145294191:0.57032518091400230542,(Bacteria-
Gramella_forsetii_120434636:0.39262649013736833359,Bacteria-
Pedobacter_sp._149280156:0.20990716432487191434)100:0.16515248378063998591)18:0.08047786450512176004,((Bacteria-
Pseudomonas_putida_119857683:0.26140995443999970327,Bacteria-
Delftia_acidovorans_118730278:0.15608337467044886004)93:0.06557730751262784430,((Bacteria-
Novosphingobium_aromaticivorans_87200161:0.16305101157096313358,Bacteria-
Erythrobacter_litoralis_85373712:0.22003787758324713786)59:0.03471502624126940328,((Bacteria-
Mesorhizobium_sp._110347229:0.09451718724845560582,(Bacteria-
Parvibaculum_lavamentivorans_154253804:0.15474775582533398244,(Bacteria-
Bradyrhizobium_sp._148258486:0.06751194983223771096,Bacteria-
Rhodopseudomonas_palustris_91976614:0.15410510429237850305)84:0.05030664081166413837)39:0.0269117303554666648
1)45:0.03207856824497930248,Bacteria-
Sphingomonas_sp._113473737:0.10550825283991674408)38:0.03054287101079858693)35:0.03477705504345687121)96:0.10
594688871681474940)12:0.03214759595144974641)47:0.09882332295708988468)100:0.40480593083953614331,(((((Chlamy
dia-Chlamydophila_abortus_62185469:0.04923317839359828141,(Chlamydia-
Chlamydophila_caviae_29840655:0.03314692739108971581,Chlamydia-
Chlamydophila_felis_89897922:0.04911246425659005627)59:0.01394579932500311301)100:0.09740272949467655705,(((Chl
amydia-Chlamydophila_pneumoniae_15836401:0.00000121823947223981,Chlamydia-
Chlamydophila_pneumoniae_33242228:0.00000121823947223981)32:0.00000121823947223981,Chlamydia-
Chlamydophila_pneumoniae_15618777:0.00000121823947223981)16:0.00000121823947223981,Chlamydia-
Chlamydophila_pneumoniae_16752171:0.00000121823947223981)100:0.14738561660424576161)92:0.0715969804065454268
3,((Chlamydia-Chlamydia_trachomatis_15605460:0.00000121823947223981,Chlamydia-
Chlamydia_trachomatis_76789467:0.00000121823947223981)100:0.03012149980741188338,Chlamydia-
Chlamydia_muridarum_15834725:0.02633972736155441516)100:0.12489316995375472630)100:0.66947066790861031738,((
Plantae-Cyanophora_paradoxa_Contig1441_4:0.62019717512083694100,Plantae-
Cyanidioschyzon_merolae_CMS330C:0.78140729351441540196)73:0.16014032840189340590,(((Plantae-
Ostreococcus_lucimarinus_27426:0.02337545583303713911,Plantae-
Ostreococcus_lucimarinus_43082:0.00000121823947223981)100:0.69993518298547974243,Plantae-
Volvox_carteri_117840_jgi:1.08177568527647838259)81:0.14345585482897768581,((Plantae-
Physcomitrella_patens_148958:0.19573447183146625017,Plantae-
Physcomitrella_patens_125638:0.16267238491670027445)100:0.15205510620856721449,(Plantae-
Arabidopsis_thaliana_15235511:0.20515762921862265578,Plantae-
Oryza_sativa_115469636:0.17574077517038499963)100:0.16741634328693208444)100:0.46166282184857404536)41:0.09297
295116822909544)62:0.15466938401472829501)100:0.49423193023439032645,((((Bacteria-
Listeria_welshimeri_116872028:0.44831884264930615958,Bacteria-
Lactobacillus_casei_116493695:0.78913803206120758382)99:0.36739722463002721931,((Cyanobacteria-
Nodularia_spumigena_CCY9414_119509788:0.11065606698903063254,((Cyanobacteria-
Anabaena_variabilis_ATCC_29413_75910063:0.02922165058579600377,Cyanobacteria-
Nostoc_sp._PCC_7120_17230653:0.04151439933207710237)100:0.06879080034519730047,Cyanobacteria-
Nostoc_punctiforme_PCC_73102_23130381:0.11804540122786683076)75:0.03563856187349884186)100:0.145091108152126
11367,(Cyanobacteria-Synechocystis_sp._PCC_6803_16331905:0.22245545757562260047,Cyanobacteria-
Lyngbya_sp._PCC_8106_119483255:0.24148373678436380052)40:0.05077929644076890903)100:0.32717343842459362113)
76:0.09233021247834950762,Bacteria-
Streptomyces_coelicolor_21224766:0.82766147557579528016)80:0.09935826858064079892,Bacteria-
Roseiflexus_sp._148654633:0.55798606820816665230)58:0.07554207171844172508)29:0.04909502398626779812)62:0.10425
489423165400227)65:0.11879850020740474748)99:0.32425931084394932213,((Bacteria-
Psychrobacter_sp._148653417:1.35069724749648889528,Bacteria-
delta_proteobacterium_94266488:0.58628036367585656397)94:0.25987224796241764624,(Bacteria-
Burkholderia_xenovorans_91777199:0.30886385997356158306,Bacteria-
Acidovorax_sp._121592455:0.32667570984467125816)100:0.26095071685004583850)56:0.06459931450600697733)98:0.1291
6310619589083108,((Bacteria-Desulfovibrio_vulgaris_120601982:0.53499883255929481241,Bacteria-
Deinococcus_radiodurans_15807440:0.50222953273843584654)45:0.06976672359061084072,Bacteria-
Shewanella_sp._114048146:0.68172474047566722355)82:0.07445746064327415925)62:0.06794119405324357264)93:0.16724
637940860884178,((Opisthokonta-Saccharomyces_cerevisiae_6320475:0.66850272919853415132,Excavata-
Trypanosoma_brucei_71754605:0.91764695820109176694)43:0.15094222497109069381,(Excavata-Naegleria_gruberi_275-
gw1.12.7.1:0.74744372974597783621,Opisthokonta-

Schizosaccharomyces_pombe_19112621:0.65467661904730900524)68:0.16631269708413648489)9:0.04084353419705822469
)10:0.04987687384396571888)16:0.05468905333253711032,((Plantae-
Physcomitrella_patens_192723:0.24998836665623833109,((Plantae-
Oryza_sativa_115444507:0.05566886776681691240,Plantae-
Oryza_sativa_115469412:0.25922744566240457642)100:0.11171845568368790613,Plantae-
Arabidopsis_thaliana_15241582:0.13096655623867828333)100:0.14481103574773854237)100:0.14654997497869401424,Plan
tae-
Chlamydomonas_reinhardtii_121438:0.47321035450766196773)52:0.08335866302685793205)51:0.12240051390082427873,C
hromalveolata-Thalassiosira_pseudonana_263051:0.66505817017396973156)100:0.56613230958531446380,Chromalveolata-
Phytophthora_sojae_129168:0.00000121823947223981);

Plantae-Arabidopsis_thaliana_15236554
(Plantae-Oryza_sativa_115462913:0.02030347321143023340,((Plantae-
Physcomitrella_patens_47148:0.31643458539383906292,((Plantae-
Arabidopsis_thaliana_15240108:0.24283398515828519271,Plantae-
Oryza_sativa_115454113:0.53528741062950124796)100:0.41333705658279273898,((((Bacteria-
Plesiocystis_pacifica_149921897:0.89151996604516758893,(Chlamydia-
Chlamydia_trachomatis_15605533:0.00548635165049896935,Chlamydia-
Chlamydia_trachomatis_76789542:0.00000121823947223981)100:1.11657393865327403404)61:0.28409389617738584066,((((
Bacteria-Magnetococcus_sp._117924129:0.50437548799578935110,Bacteria-
Mariprofundus_ferrooxydans_114777713:0.72652188193795996796)63:0.10790141837985921247,(((Bacteria-
Dechloromonas_aromatica_71909343:0.33017164425633505065,Bacteria-
Ralstonia_solanacearum_17545113:0.32490180642662108701)90:0.13348951733994937618,((Bacteria-
Nitrosococcus_oceani_77164043:0.38601896329189394619,(Bacteria-
Legionella_pneumophila_148358613:0.46745554950142437223,(Bacteria-
Methylococcus_capsulatus_53804648:0.32739779670854657345,Bacteria-
Pseudomonas_stutzeri_146283515:0.39847425740875352940)35:0.07262680012072669777)29:0.08787207389886600439)31:0
.07902181959038533099,Bacteria-
Psychrobacter_sp._148653903:0.62681729713547629235)43:0.08135854808294887275)84:0.24535316757355993267,((Bacteri
a-Anaplasma_marginale_56417306:0.43262696925039212381,Bacteria-
Ehrlichia_ruminantium_58578753:0.30854628112121179617)100:0.40740158005619153680,Bacteria-
Thermosipho_melanesiensis_150020070:0.82366068666404890219)44:0.12359477121302955838)26:0.1028606953103368276
7)26:0.13904187939224035842,(Chromalveolata-Phytophthora_sojae_143144:1.20546248405775369150,((((Bacteria-
alpha_proteobacterium_114769270:0.24099230980948904701,(Bacteria-
Rhodobacter_sphaeroides_126463245:0.18454260956924653514,(Bacteria-
Jannaschia_sp._89056087:0.10897825726449936190,Bacteria-
Roseobacter_denitrificans_110681037:0.17002086282042569443)99:0.11124643812722785630)86:0.10052311820580354940)
100:0.38294675769488389916,(Bacteria-Maricaulis_maris_114569308:0.31352712693028861324,(Bacteria-
Caulobacter_crescentus_16124740:0.50708828632009128601,Bacteria-
Hyphomonas_neptunium_114797515:0.36053691932724074576)60:0.11722184839324169270)82:0.19690903870617157412)1
7:0.06482553168617710693,(((Bacteria-Methylobacterium_sp._149118651:0.50345567101070165528,(Bacteria-
Xanthobacter_autotrophicus_154246548:0.20106784312005243631,(Bacteria-
Bradyrhizobium_japonicum_27382552:0.06762513788533354442,(Bacteria-
Rhodopseudomonas_palustris_90422926:0.16134094840032825457,Bacteria-
Nitrobacter_winogradskyi_75676696:0.11662255074838004387)69:0.04665713559597396004)100:0.33049270346707199097)
89:0.11047100814703320060)90:0.16328052412413507355,(Bacteria-
Parvibaculum_lavamentivorans_154252704:0.37775685485075277681,(((Bacteria-
Mesorhizobium_sp._110634506:0.14323481319810205115,((Bacteria-
Brucella_ovis_148559338:0.02714547359939447138,Bacteria-
Ochrobactrum_anthropi_153008961:0.03236507816444732927)100:0.09784336444112283482,Bacteria-
Bartonella_bacilliformis_121602891:0.18532837120811332388)94:0.09194690599916616192)61:0.03181530815893735681,((
Bacteria-Sinorhizobium_medicae_150397463:0.17418959848343962271,(Bacteria-
Rhizobium_etli_86358627:0.11477518536983427611,Bacteria-
Agrobacterium_tumefaciens_15889503:0.09523598177110072227)97:0.08425412195927718673)92:0.08484781849695501033,
(Bacteria-Fulvimarina_pelagi_114707023:0.19189493878110785996,Bacteria-
Aurantimonas_sp._90418284:0.09411584253342919482)100:0.15522018608414364649)22:0.04037672078959722577)58:0.054
93115833817326793,Bacteria-
Stappia_aggregata_118591923:0.34868355849992077600)56:0.07578769552084557715)36:0.07997935619138767105)21:0.093
26750259848737290,((Bacteria-Magnetospirillum_magneticum_83313447:0.59385238767351111733,(Bacteria-
Rhodospirillum_rubrum_83595075:0.29201399968047303535,(Bacteria-
Acidiphilium_cryptum_148260671:0.17851001099098887104,Bacteria-
Granulibacter_bethesdensis_114328980:0.11412530799891416888)100:0.40671354969348899600)69:0.0771122292570670525

6)53:0.1077730762976587125,(((Bacteria-Novosphingobium_aromaticivorans_87200131:0.14822597319445338093,Bacteria-
Sphingomonas_sp._94498282:0.16181142548250529445)41:0.06422133361750662073,Bacteria-
Sphingopyxis_alaskensis_103487102:0.11409128387424297135)56:0.06807858546606825045,Bacteria-
Erythrobacter_sp._149184486:0.19890600853814704996)99:0.33215637282669818742)4:0.00059432893944164479)12:0.0671
6784114760591951)11:0.03724620218499700985,(Bacteria-
Candidatus_Pelagibacter_71082803:0.17241924269285829885,Bacteria-
Psychroflexus_torquis_91220193:0.06846452475374879831)100:0.57798430051168936394)25:0.03066046051904691705)36:0
.09381892465479378662)13:0.07506565620308067177,(Bacteria-
Orientia_tsutsugamushi_148284981:0.44168564756816086625,Bacteria-
Rickettsia_akari_157825998:0.37534423153895335457)100:0.39305504347124520237)53:0.11451212994578217896)74:0.173
25714016139731610,(Plantae-Ostreococcus_tauri_35604:0.13399787630270701411,Plantae-
Ostreococcus_lucimarinus_93468:0.29299357109952528511)100:0.65710185542009436066)66:0.07424208539954089969,(Pla
ntae-Volvox_carteri_106036_jgi:0.51243152783657486271,Plantae-
Chlamydomonas_reinhardtii_186521:0.16136294966814318519)100:0.99652644508374099708)99:0.31473374054248570708)
52:0.19045772206257932657)96:0.21689471326087089720,Plantae-
Arabidopsis_thaliana_15236554:0.20671045593178166699)98:0.17804360593053802919,Plantae-
Oryza_sativa_115438116:0.00467149677673165190);

Plantae-Arabidopsis_thaliana_15237422

(Bacteria-Bdellovibrio_bacteriovorus_42523485:0.59109141181568625178,((((((Bacteria-
Algoriphagus_sp._126648065:0.17699381976750577783,(Bacteria-
Psychroflexus_torquis_91215537:0.13446376260290116011,Bacteria-
Flavobacteria_bacterium_89889944:0.17591790823729919202)100:0.16908224870468399614)100:0.41997851991768875290,(
((Bacteria-Pelotomaculum_thermopropionicum_147678074:0.25581172023880160982,Bacteria-
Desulfotomaculum_reducens_134299916:0.23313974950021079668)78:0.09261064613230178544,((Bacteria-
Thermosinus_carboxydivorans_121533803:0.21451695523079827854,Bacteria-
Moorella_thermoacetica_83589800:0.31206985230630818196)51:0.06217144767778709502,(Bacteria-
Alkaliphilus_metalliredigens_150390514:0.21401370413860829056,Bacteria-
Clostridium_sp._106895571:0.22692719214695736274)100:0.15861213179784702798)16:0.03017199498879639258)62:0.051
44678942863968596,(Cyanobacteria-Synechococcus_sp._JA-2-3Ba2-
13_86609126:0.23422863709171798385,(((Cyanobacteria-
Nostoc_punctiforme_PCC_73102_23124097:0.10127778963451937455,(Cyanobacteria-
Nostoc_sp._PCC_7120_17230835:0.00246891652589342021,Cyanobacteria-
Anabaena_variabilis_ATCC_29413_75909851:0.00797721612785045470)100:0.07151685837906902876)63:0.0385042254858
2720095,(Cyanobacteria-Thermosynechococcus_elongatus_BP-1_22299414:0.17084931462344193909,((Cyanobacteria-
Synechocystis_sp._PCC_6803_16329903:0.19010116372830432319,(Cyanobacteria-
Crocosphaera_watsonii_WH_8501_67920030:0.05564772609339342646,Cyanobacteria-
Cyanothece_sp._CCY0110_126657283:0.02825438719494655082)98:0.06739390793692535964)72:0.05937641241940428555,
Cyanobacteria-
Trichodesmium_erythraeum_IMS101_113474322:0.16969948200421536288)53:0.05222613238384239687)52:0.030844157304
34945241)54:0.06659882414044782095,((Cyanobacteria-
Synechococcus_sp._WH_5701_87301978:0.10856347284747867865,(Cyanobacteria-
Synechococcus_sp._RCC307_148241230:0.09947788166153237466,(((Cyanobacteria-
Prochlorococcus_marinus_str._MIT_9515_123967030:0.06917626934793023874,((Cyanobacteria-
Prochlorococcus_marinus_str._MIT_9301_126697140:0.02252210345545444317,Cyanobacteria-
Prochlorococcus_marinus_str._AS9601_123969351:0.02285369644540620818)61:0.01923495109414415788,Cyanobacteria-
Prochlorococcus_marinus_str._MIT_9312_78780087:0.02849252420286479612)78:0.01716730342914042640)100:0.29796133
519817746826,(Cyanobacteria-Synechococcus_sp._RS9917_87123205:0.10801502774232671356,Cyanobacteria-
Synechococcus_sp._RS9916_116074418:0.04941720968996076574)68:0.02605552831096730887)33:0.021863564421382961 7
7,((Cyanobacteria-Synechococcus_sp._CC9605_78211677:0.03325272343344303633,Cyanobacteria-
Synechococcus_sp._WH_8102_33864678:0.02100948613982499616)86:0.03408496279713991994,Cyanobacteria-
Synechococcus_sp._BL107_116071647:0.05303187045558688945)84:0.03236203851070561244)59:0.06287256366040272493
)76:0.09226219394987131284)100:0.31130282691396066186,(Cyanobacteria-
Synechococcus_elongatus_PCC_6301_56750993:0.00000121823947223981,Cyanobacteria-
Synechococcus_elongatus_PCC_7942_81299348:0.00000121823947223981)100:0.13826643658074969090)52:0.06004060551
460830425)80:0.13690123247518190541)100:0.22258239674241039885)66:0.03712312100525237690)20:0.043382107934144
92422,Bacteria-Thermus_thermophilus_46198353:0.39615654105288117259)12:0.04904203431843665723,(Bacteria-
Hydrogenobaculum_sp._156719562:0.35677659015873286474,((((Chlamydia-
Chlamydophila_pneumoniae_33242279:0.00000121823947223981,Chlamydia-
Chlamydophila_pneumoniae_16752120:0.00000121823947223981)30:0.00000121823947223981,(Chlamydia-
Chlamydophila_pneumoniae_15618825:0.00000121823947223981,Chlamydia-
Chlamydophila_pneumoniae_15836449:0.00000121823947223981)19:0.00000121823947223981)100:0.06944938190388059998

4,((Chlamydia-Chlamydia_muridarum_15834771:0.0149792322189251821 1,(Chlamydia-
Chlamydia_trachomatis_15605503:0.0000121823947223981,Chlamydia-
Chlamydia_trachomatis_76789512:0.0053598801195131615 5)99:0.0065972457558810286 2)98:0.0809700674681335247 4,((Ch
lamydia-Chlamydophila_caviae_29840610:0.0029490500418456202 2,Chlamydia-
Chlamydophila_felis_89897970:0.0105321678402593432 7)77:0.0099231252318601574 6,Chlamydia-
Chlamydophila_abortus_62185423:0.0205755272556613355 6)69:0.0349555083625480919 6)32:0.0248020604869186103 7)100:
0.4032774771462299678 9,(Chlamydia-Candidatus_Protochlamydia_46446872:0.3089887736562739739 6,((((Plantae-
Ostreococcus_tauri_25038:0.0357226606785182657 1,Plantae-
Ostreococcus_lucimarinus_35878:0.0266709185369720866 4)100:0.1602890690216594860 0,(Plantae-
Volvox_carteri_83790_jgi:0.0209662959640300808 3,Plantae-
Chlamydomonas_reinhardtii_132552:0.1634575322933793373 2)100:0.1790140711847672261 3)99:0.1005411253267481791 5,(
(Plantae-Physcomitrella_patens_124905:0.0000121823947223981,(Plantae-
Physcomitrella_patens_220915:0.0123630999188688132 9,Plantae-
Physcomitrella_patens_125854:0.0584103176709834040 5)93:0.0156027616772186367 8)100:0.0875921735755829933 3,((Plant
ae-Arabidopsis_thaliana_79329956:0.0030249379468599620 8,Plantae-
Arabidopsis_thaliana_15237422:0.0000121823947223981)100:0.0909643008818169507 3,(Plantae-
Oryza_sativa_115458608:0.0805430090564780987 2,Plantae-
Oryza_sativa_115466908:0.0454731427463128598 5)85:0.0423509835898077460 8)73:0.0461918616373171581 1)87:0.0514376
1199960728697)100:0.1988372302827599302 8,(((Plantae-Ostreococcus_tauri_13792:0.0288509316641010447 9,(Plantae-
Ostreococcus_lucimarinus_13257:0.0000121823947223981,Plantae-
Ostreococcus_lucimarinus_18112:0.0027123070321553423 6)100:0.0522367062890025726 8)100:0.4491075142721243085 3,((P
lantae-Volvox_carteri_104947_jgi:0.0640286127278604094 7,Plantae-
Chlamydomonas_reinhardtii_139619:0.0842817251888883650 6)100:0.2298302305133906320 3,((Plantae-
Physcomitrella_patens_230580:0.1285881598501793510 2,(Plantae-
Physcomitrella_patens_214865:0.0364955844949491764 5,(Plantae-
Physcomitrella_patens_87584:0.0292331840268146153 6,Plantae-
Physcomitrella_patens_182892:0.0373693948057347846 7)93:0.0200931694328098861 5)92:0.0355603556966544859 2)85:0.05
041976931157626796,(Plantae-Oryza_sativa_115473351:0.1596028474047925804 8,(Plantae-
Arabidopsis_thaliana_18410739:0.0000121823947223981,Plantae-
Arabidopsis_thaliana_30699063:0.0000121823947223981)100:0.1192944425595650476 1)93:0.0658154747071128393 1)100:0
.1833948788910121363 6)77:0.0743975704611962857 0)99:0.1402520560391540271 9,Plantae-
Cyanidioschyzon_merolae_CMM286C:0.5203021001594851924 2)30:0.0487628967114933434 3)34:0.0508299891702514369 8)
45:0.1209728971131407010 4)100:0.3702335566799811461 5)23:0.0999569754642233299 1)85:0.0745813810338290111 8,((Ba
cteria-Pelobacter_carbinolicus_77919041:0.3022507049888927266 3,(Bacteria-
Stigmatella_aurantiaca_115377154:0.1156603358474717208 0,Bacteria-
Myxococcus_xanthus_108761812:0.1138645442764876225 5)100:0.2544677996946232978 5)94:0.0825764031038916102 0,Bac
teria-
Anaeromyxobacter_dehalogenans_86159168:0.3046051448426926322 5)88:0.0692018320279859516 8)68:0.0452028270100629
9308,(Bacteria-Xylella_fastidiosa_28199381:0.3939776904574264215 8,((Bacteria-
Coxiella_burnetii_153207958:0.3934025806380093692 7,((((Bacteria-
Psychromonas_sp._90406891:0.2882984809511012214 1,(((((Bacteria-
Vibrionales_bacterium_148980542:0.0440428808854037359 7,Bacteria-
Vibrio_cholerae_116215506:0.0658865086565633140 1)100:0.0695102208597951909 4,Bacteria-
Photobacterium_profundum_54308390:0.1160631612152630554 0)100:0.1071699580635168264 1,(Bacteria-
Sodalis_glossinidius_85059040:0.1195950599773948352 9,((Bacteria-
Serratia_proteamaculans_157370150:0.0656430044422770531 1,Bacteria-
Yersinia_enterocolitica_123441943:0.0320186150760570359 4)41:0.0239394097279518915 1,(Bacteria-
Salmonella_enterica_62179715:0.0287819346692598371 2,((Bacteria-
Shigella_flexneri_24112501:0.0000121823947223981,Bacteria-
Escherichia_coli_110641271:0.0000121823947223981)100:0.0291165903821556659 7,(Bacteria-
Enterobacter_sp._146311266:0.0217483217054926791 8,Bacteria-
Klebsiella_pneumoniae_152969647:0.0156669163893359310 3)69:0.0167167340734320837 8)48:0.0080520406246170524 2)91:
0.0432678036820357062 0)58:0.0245938689622589891 4)100:0.0963210737974771746 8)37:0.0300985613252305779 5,(Bacteri
a-Shewanella_amazonensis_119775113:0.2313223207123688562 9,Bacteria-
Pseudoalteromonas_atlantica_109898437:0.2849966329914038754 0)29:0.0327767316786587784 0)29:0.0303628681728433935
7,Bacteria-
Aeromonas_salmonicida_145299026:0.1868691895159934612 8)56:0.0409707385552915345 8)73:0.0618183788263831357 5,(B
acteria-marine_gamma_119478167:0.2650592226329846479 2,Bacteria-
Chromohalobacter_salexigens_92113727:0.2175225010636625822 0)55:0.0681726422095627893 1)42:0.0655440588417454395
0,((Bacteria-Marinobacter_aquaeolei_120554783:0.3551206060222828475 6,Bacteria-
Pseudomonas_putida_148549004:0.2195553854885066436 0)65:0.0732331367881368100 5,(Bacteria-
Nitrosococcus_oceani_77165140:0.2636894621988657982 9,Bacteria-

Methylococcus_capsulatus_53803736:0.20536110925567907515)96:0.12052186395721306567)13:0.01754952088693003537)2
3:0.04117015031192367003,Bacteria-
Marinomonas_sp._152996156:0.31343679665369361675)44:0.02625527367234199369)64:0.06228995781736944987,((Bacteri
a-Limnobacter_sp._149926453:0.18622612908305094526,(((Bacteria-
Nitrosospira_multiformis_82702205:0.09625242615357675058,Bacteria-
Nitrosomonas_europaea_30249614:0.17549100526638761188)100:0.11238500832488668157,Bacteria-
Azoarcus_sp._56478544:0.17361424245228790353)95:0.10460506471701047637,((Bacteria-
Dechloromonas_aromatica_71907647:0.21114268506066957709,(Bacteria-
Methylibium_petroleiphilum_124265833:0.18911439032444854469,Bacteria-
Ralstonia_solanacearum_17545773:0.09220337717943938305)81:0.04124914063566992306)66:0.05656846100408211703,Bac
teria-
Burkholderia_phymatum_118029130:0.12238080199463442777)91:0.07994150789179854666)21:0.02786289895281223589)6
0:0.07493029168115840355,Bacteria-
Neisseria_meningitidis_121634093:0.31784051436327770412)94:0.10771385951593166752)64:0.05968528986685280729)84:
0.10459950852686974998)41:0.08449302141840261959,Bacteria-Magnetococcus_sp._117925169:0.35045258190447375890);

Plantae-Arabidopsis_thaliana_15237589
(((((Plantae-Ostreococcus_lucimarinus_26206:1.45340104090238275703,(Plantae-
Physcomitrella_patens_15369:0.76521208984685984422,(Plantae-
Arabidopsis_thaliana_15234590:0.30582997721446447681,(Plantae-
Oryza_sativa_115472751:0.16395644466137396233,Plantae-
Oryza_sativa_115472749:0.10577676427759462319)100:0.21500437824725379676)100:0.37513445926673594233)79:0.23825
759054980605312)99:0.44032834968399353626,(((((Cyanobacteria-
Lyngbya_sp._PCC_8106_119492930:0.12117278853895861734,Cyanobacteria-
Trichodesmium_erythraeum_IMS101_113475463:0.15384003344840868621)99:0.21086223444315618791,((Cyanobacteria-
Synechococcus_elongatus_PCC_7942_81299774:0.00000121823947223981,Cyanobacteria-
Synechococcus_elongatus_PCC_6301_56750586:0.00000121823947223981)100:0.66486642268720208548,(Cyanobacteria-
Cyanothece_sp._CCY0110_126656987:0.05105363410518552747,Cyanobacteria-
Crocosphaera_watsonii_WH_8501_67922379:0.12898398888453196931)100:0.43300189264037547421)41:0.07448874964048
075864)94:0.18738608336244746977,Bacteria-
Salinibacter_ruber_83815205:0.48197043413409046408)85:0.20277501349990165180,((Amoebozoa-
Entamoeba_histolytica_67472180:0.70921755409116704616,Opisthokonta-
Saccharomyces_cerevisiae_6324508:0.68079627999792557969)39:0.08495507530093419912,((((Chromalveolata-
Alexandrium_tamarense_UI-D-GC1-aac-d-12-0-UI.s1_6:0.33501945767299534662,Excavata-
Euglena_gracilis_109783578_1:0.14255494598134607620)100:0.54469935396658064608,Opisthokonta-
Sporobolomyces_roseus_31017-
estExt_fgenesh1_kg.C_90003:0.21606189448957777155)16:0.08068493967050860416,(Opisthokonta-
Ustilago_maydis_71006290:0.22825689954676067761,Opisthokonta-
Cryptococcus_neoformans_58259485:0.59450493842994522886)63:0.09578183643253679325)24:0.12298452262745934727,(
Opisthokonta-Schizosaccharomyces_pombe_19075730:0.48533565984431653595,Opisthokonta-
Magnaporthe_grisea_39944600:0.38891348030064742947)27:0.05726148421261940957)34:0.12278893951614444635)50:0.12
331966427751238358)52:0.08237411987454289208,((((Plantae-
Ostreococcus_lucimarinus_4650:0.06765298642932611461,Plantae-
Ostreococcus_tauri_27888:0.04408407395257035039)100:0.39522932769149843324,((Plantae-
Arabidopsis_thaliana_15239631:0.24119764023298959077,Plantae-
Oryza_sativa_115449335:0.16209877289360771768)96:0.14817712389044468413,(Plantae-
Physcomitrella_patens_191256:0.02440239519247265967,Plantae-
Physcomitrella_patens_162819:0.03949578908804328436)99:0.12831557973980445286)99:0.25277895668261368689)86:0.21
02212827838564612 7,((Plantae-Physcomitrella_patens_130451:0.20413633254813734474,((Plantae-
Arabidopsis_thaliana_15237567:0.09028451423160728517,((Plantae-
Arabidopsis_thaliana_18424775:0.00255101151606240912,Plantae-
Arabidopsis_thaliana_42573784:0.00451548961458517542)100:0.23214084764215675571,(Plantae-
Arabidopsis_thaliana_15237589:0.12419158374869412875,Plantae-
Arabidopsis_thaliana_42567747:0.19544552593058142631)64:0.03759457703359345843)100:0.14312883752271859938)97:0.
07919089750148368412,(Plantae-Oryza_sativa_115487678:0.08349054712807121859,Plantae-
Oryza_sativa_115487676:0.17558139572121594929)100:0.11364430194481991987)100:0.14523371068061438627)99:0.17213
887449504314264,((Plantae-Ostreococcus_tauri_13112:0.18468937778015614981,Plantae-
Ostreococcus_lucimarinus_4881:0.09004071529300770871)100:0.48686242867125512079,(Plantae-
Volvox_carteri_65837_jgi:0.10697820010474537455,Plantae-
Chlamydomonas_reinhardtii_146533:0.11813665357022117008)100:0.32598314854278936936)43:0.07025717428051082847)
33:0.05117459696733066277)66:0.10931390141255098447,(Plantae-
Chondrus_crispus_Contig163_2:0.73247291961582350339,(Plantae-

Cyanidioschyzon_merolae_CMR198C:0.57793060726319800402,(Chromalveolata-
Aureococcus_anophagefferens_70779:0.81012434977244174306,(Chromalveolata-
Phaeodactylum_tricornutum_41875:0.49317911832080701950,Chromalveolata-
Thalassiosira_pseudonana_263722:0.60635988787711114778)96:0.21617333972104590956)84:0.17620039527200087592)67:0
.06837776701031117454)83:0.18746057605331276075)32:0.08520322793109362525)34:0.08282546450589953202)100:1.753
85369745594021929,((Chlamydia-Chlamydia_trachomatis_15605507:0.00000121823947223981,Chlamydia-
Chlamydia_trachomatis_76789516:0.00000121823947223981)100:0.07795904904215761322,Chlamydia-
Chlamydia_muridarum_15834775:0.11165577515343927395)100:0.36526544848378372787)95:0.42817418002227314311,Chl
amydia-Chlamydophila_felis_89897974:0.05717520897506589428)74:0.03614982945106461065,Chlamydia-
Chlamydophila_caviae_29840606:0.05427036943543742109,Chlamydia-
Chlamydophila_abortus_62185419:0.10180727810348884355);

Plantae-Arabidopsis_thaliana_15240411
(((((Bacteria-Frankia_alni_111224492:1.23442248381510677468,Bacteria-
Ruminococcus_gnavus_154505436:0.88314643369781797855)33:0.24229924514543804692,((Bacteria-
Orientia_tsutsugamushi_148284671:0.59831145018328402152,Bacteria-
Ehrlichia_canis_73667124:0.58269952676652114754)100:0.67862370150588902096,Bacteria-
Corynebacterium_jeikeium_68535768:1.20446391170155298056)29:0.16703475293117200917)89:0.27471950130894373654,(
(((Plantae-Arabidopsis_thaliana_30690468:0.00000121823947223981,Plantae-
Arabidopsis_thaliana_15240411:0.00000121823947223981)23:0.00000121823947223981,(Plantae-
Arabidopsis_thaliana_30690464:0.00000121823947223981,Plantae-
Arabidopsis_thaliana_42573491:0.00000121823947223981)26:0.00000121823947223981)100:0.39818053259459523785,(Plant
ae-Oryza_sativa_115460648:0.63233159386929471690,Plantae-
Oryza_sativa_115452527:0.43734440312226102732)96:0.20979931907933715984)95:0.32228647663332693485,Plantae-
Physcomitrella_patens_64757:0.78034396392036664558)71:0.17017713161299505287)100:0.65418766454384269515,Chlamy
dia-Candidatus_Protochlamydia_46446795:0.48479199085938756708)100:0.49468618672905378952,Chlamydia-
Chlamydophila_felis_89898431:0.06314768129728050294,Chlamydia-
Chlamydophila_caviae_29840147:0.00000121823947223981);

Plantae-Arabidopsis_thaliana_18395376
(((Bacteria-Psychromonas_ingrahamii_119944444:0.47144861409534727370,((Bacteria-
Shewanella_pealeana_157961015:0.31354687895057592462,Bacteria-
Oceanobacter_sp._94500740:0.49813697459514977295)27:0.10634117960313325957,Bacteria-
Aeromonas_salmonicida_145300348:0.19962852087761179254)16:0.03586764056944039686)27:0.06623337635316201011,(B
acteria-alpha_proteobacterium_114771622:0.51739674491496345787,((((Bacteria-
Photobacterium_sp._89075074:0.27229026956723040298,Bacteria-
Vibrio_alginolyticus_91227840:0.33510032725292898670)37:0.07699951889551388817,(Bacteria-
Yersinia_pseudotuberculosis_51595120:0.14466599366734630983,Bacteria-
Serratia_proteamaculans_157369071:0.12776365753911297918)100:0.28236877357227085916)16:0.05795561248334876736,(
Bacteria-Actinobacillus_succinogenes_152979684:0.23318153309665623496,Bacteria-
Haemophilus_influenzae_145631998:0.24635704308745887126)100:0.19071433591741213109)3:0.01625768556658750003,B
acteria-
Pseudoalteromonas_atlantica_109900156:0.37707344453824653829)7:0.04419127472239695736)3:0.03795446813185249929)
15:0.08809970523368308715,((Bacteria-marine_gamma_119504834:0.55296628852018092637,(Bacteria-
Marinomonas_sp._152995330:0.73246438662503055017,Bacteria-
Pseudomonas_stutzeri_146281933:0.34426041190899014932)69:0.11632142515462612353)37:0.08398559953337897399,((((((
(Plantae-Cyanidioschyzon_merolae_CMH115C:0.37042741449834254430,(Chromalveolata-Emiliania_huxleyi_UI-EH-IJ0-abj-
i-21-0-UI.s1_4:0.35405640720877740169,Plantae-
Galdieria_sulphuraria_A4_36H10_2:0.35398286442149640152)33:0.08855498159889556298)35:0.05658249749767156611,((P
lantae-Gracilaria_changii_120463063_2:0.30252646025450485867,(Chromalveolata-
Phaeodactylum_tricornutum_21829:0.13554095006663954837,Chromalveolata-
Thalassiosira_pseudonana_3622:0.14239967105432305150)100:0.20752385322878783014)34:0.09678686195062752295,Chro
malveolata-
Aureococcus_anophagefferens_23670:0.26896315419995686646)14:0.06811843746113280251)56:0.14580050472499317404,(
Chlamydia-Candidatus_Protochlamydia_46445961:0.64216421916058841202,((Plantae-
Ostreococcus_lucimarinus_24843:0.28048512489770338618,(Plantae-
Volvox_carteri_84386_jgi:0.01104209535545158487,Plantae-
Chlamydomonas_reinhardtii_196604:0.08225208633715794249)100:0.24755045255382912561)82:0.09758321771985870030,(
Plantae-Oryza_sativa_115441507:0.14891086090936575537,(Plantae-
Arabidopsis_thaliana_18395376:0.14900570697919879315,(Plantae-
Physcomitrella_patens_213502:0.01221630600365485042,Plantae-
Physcomitrella_patens_144079:0.03651801208181743724)100:0.19494872189236039928)48:0.01912491871552266312)91:0.1

2394700493611503223)57:0.0856914073990090361)14:0.0704799127491621302)11:0.0310651126758660495 2,Chromalveo
lata-Alexandrium_tamarense_Contig1446_4:0.3326838052983695504 0)37:0.0573610829540247210 7,((Chlamydia-
Chlamydophila_pneumoniae_15836111:0.0000012182394722398 1,(Chlamydia-
Chlamydophila_pneumoniae_16752459:0.0000012182394722398 1,(Chlamydia-
Chlamydophila_pneumoniae_15618489:0.0000012182394722398 1,Chlamydia-
Chlamydophila_pneumoniae_33241934:0.0000012182394722398 1)31:0.0000012182394722398 1)13:0.0000012182394722398 1
)100:0.1825076248051654970 2,((Chlamydia-Chlamydia_muridarum_15835361:0.0770533354959646588 1,(Chlamydia-
Chlamydia_trachomatis_76789198:0.0000012182394722398 1,Chlamydia-
Chlamydia_trachomatis_15605189:0.0000012182394722398 1)100:0.0895809891292710658 3)100:0.3318734533860847046 9,(
Chlamydia-Chlamydophila_abortus_62184805:0.0762129446042938141 2,(Chlamydia-
Chlamydophila_caviae_29839929:0.0859955871516665526 5,Chlamydia-
Chlamydophila_felis_89898652:0.0621296851264805730 1)80:0.0413901281054882691 5)100:0.1371139748776447064 8)72:0.0
9386554485798757896)100:0.5784172863336419778 7)97:0.2865571951775248704 7,((Bacteria-
Magnetococcus_sp._117925957:0.7437559264492892285 9,(Bacteria-
Xanthobacter_autotrophicus_154248322:0.5751081496765693135 1,Bacteria-
Mariprofundus_ferrooxydans_114777097:0.6520462588020543570 6)59:0.0750783556471957963 5)89:0.283124320991509303
98,((Bacteria-Desulfitobacterium_hafniense_109647497:0.5095858122663838862 8,((Bacteria-
Collinsella_aerofaciens_139438522:0.9453175178739692263 7,(((Cyanobacteria-
Prochlorococcus_marinus_str._MIT_9515_123965752:0.1156980718971032939 8,(Cyanobacteria-
Prochlorococcus_marinus_str._MIT_9312_78778839:0.0060980665313668941 4,(Cyanobacteria-
Prochlorococcus_marinus_str._AS9601_123968047:0.0304722716042229724 3,Cyanobacteria-
Prochlorococcus_marinus_str._MIT_9301_126695817:0.0070879823808580617 2)98:0.0246149502720617759 1)96:0.10481166
644496452134)100:0.4134669840725509470 9,(((((Cyanobacteria-
Synechococcus_sp._RS9916_116073467:0.1551557767893651518 2,(Cyanobacteria-
Synechococcus_sp._CC9311_113953866:0.1921277990536886526 3,((Cyanobacteria-
Synechococcus_sp._CC9902_78185308:0.0086912269030237562 8,Cyanobacteria-
Synechococcus_sp._BL107_116072811:0.0064680818277076607 5)100:0.1793999443172093200 0,Cyanobacteria-
Synechococcus_sp._WH_8102_33866381:0.0986833859750304159 8)77:0.0491942467031319316 5)64:0.022908022582009225
64)71:0.0333347524418667745 5,(Cyanobacteria-
Prochlorococcus_marinus_str._MIT_9303_124022365:0.0053360282817037901 1,Cyanobacteria-
Prochlorococcus_marinus_str._MIT_9313_33863597:0.0094980669291200459 8)100:0.1244978980464345325 0)51:0.03391270
331240450048,Cyanobacteria-
Synechococcus_sp._RS9917_87123748:0.0951156687308474108 1)55:0.0537096726958818798 7,Cyanobacteria-
Synechococcus_sp._WH_5701_87302092:0.2514432271802762297 6)63:0.0559877436797263025 6,(Cyanobacteria-
Prochlorococcus_marinus_str._MIT_9211_84518498:0.2119222863967208658 8,Cyanobacteria-
Prochlorococcus_marinus_subsp._marinus_str._CCMP1375_33239905:0.2417606167394547866 9)73:0.0809497760320100484
5)79:0.1163155628002036784 4)76:0.0453101015374215709 8,((Cyanobacteria-Synechococcus_sp._JA-3-
3Ab_86606172:0.2518932593925072849 2,((((Cyanobacteria-
Crocosphaera_watsonii_WH_8501_67924037:0.1541907293911999876 6,Cyanobacteria-
Synechocystis_sp._PCC_6803_16331009:0.2075017135922487543 4)42:0.0424281790033420383 4,(Cyanobacteria-
Trichodesmium_erythraeum_IMS101_113474465:0.2216841414068559112 9,Cyanobacteria-
Lyngbya_sp._PCC_8106_119483239:0.1414215394186695562 4)28:0.0382666972962292656 3)68:0.0648714762082339691 0,(
Cyanobacteria-Nodularia_spumigena_CCY9414_119510035:0.0775451859321866637 8,(Cyanobacteria-
Nostoc_sp._PCC_7120_17232659:0.0030136805333971189 3,Cyanobacteria-
Anabaena_variabilis_ATCC_29413_75908630:0.0078026535807645784 6)99:0.0740064207507123150 7)100:0.1473683308857
9115447)80:0.0811390582456113507 2,Cyanobacteria-Thermosynechococcus_elongatus_BP-
1_22298148:0.3136021719572135158 0)35:0.0277650045414046774 1)58:0.0762388892338817375 5,(Cyanobacteria-
Gloeobacter_violaceus_PCC_7421_37522360:0.3943997615687980795 6,(Cyanobacteria-
Synechococcus_elongatus_PCC_6301_56750857:0.0111612873162948347 1,Cyanobacteria-
Synechococcus_elongatus_PCC_7942_81299492:0.0000012182394722398 1)100:0.2140229458187787314 7)29:0.03653005361
648018617)73:0.0833273827859968008 2)99:0.2884884147537046805 7)67:0.1580865121177271459 5,((Bacteria-
Listeria_welshimeri_116871619:0.5353265604728960624 9,(Bacteria-
Bacillus_amyloliquefaciens_154684608:0.3203982617167177027 2,Bacteria-
Geobacillus_thermodenitrificans_138893760:0.3168755938491720214 3)54:0.0743254419825046946 9)100:0.222012125147001
11034,(((Bacteria-Symbiobacterium_thermophilum_51894258:0.4785012593774772837 7,(Bacteria-
Bacteroides_capillosus_154497964:0.6031016542455360784 9,Bacteria-
Thermosinus_carboxydivorans_121535692:0.2902317135750585586 1)34:0.0769775114422445450 0)33:0.14094826819590744
460,(Bacteria-Syntrophomonas_wolfei_114567867:0.5299681167475265297 6,Bacteria-
Moorella_thermoacetica_83591303:0.4335250422705752004 8)42:0.1281688284629135810 0)4:0.0485304650540469700 9,((((B
acteria-Pelobacter_carbinolicus_77917721:0.2652902971562036138 5,Bacteria-
Desulfuromonas_acetoxidans_95929124:0.3461435753969911988 1)96:0.1851589979824250775 7,Bacteria-
Geobacter_uraniumreducens_148266173:0.3194321697228590384 3)75:0.1586852885185117967 3,(Bacteria-

Alkaliphilus_metalliredigens_150392188:0.48324431920889987513,(Bacteria-
Clostridium_perfringens_110803205:0.47919478402374743320,(Bacteria-
Eubacterium_ventriosum_154483094:0.40820734493586463554,(Bacteria-
Dorea_longicatena_153856040:0.41965292720029401252,Bacteria-
Ruminococcus_gnavus_154502854:0.18633444519790220983)96:0.12890449242552362574)81:0.13330722767948030572)71:
0.12935328081743180384)31:0.05967060593065264679)6:0.04289941683126283212,(Bacteria-
Dehalococcoides_ethenogenes_57233576:0.58219853499733631441,((Bacteria-
Syntrophobacter_fumaroxidans_116749072:0.59590265209065618190,(Bacteria-
Solibacter_usitatus_116620981:0.60849793576327859679,(Bacteria-
delta_proteobacterium_94263737:0.53515596172120871721,Bacteria-
Desulfotalea_psychrophila_51244109:0.44257219282377907588)69:0.13426742953171860728)26:0.11710061755990237853)6
:0.04163118078787234749,(Bacteria-Syntrophus_aciditrophicus_85859809:0.45611996147464112239,((Bacteria-
Lactobacillus_casei_116494597:1.17599079642140069701,Bacteria-
Carboxydothermus_hydrogenoformans_78043736:0.93378229716059646393)8:0.04940192105388534899,(Bacteria-
Cytophaga_hutchinsonii_110639473:0.41009457293366885500,Bacteria-
Microscilla_marina_124008760:0.35905757867079629575)100:0.44604685213206285432)19:0.07685256378632643326)7:0.06
34486767171 2194068)2:0.05037643769996497101)1:0.06690726882114894636)0:0.05093028540628854323)4:0.04963289273
194793766)3:0.02847546974174226808)4:0.03362293574941853380)2:0.01873425865646077826,(Bacteria-
Desulfotomaculum_reducens_134298065:0.39526410186191218710,Bacteria-
Pelotomaculum_thermopropionicum_147676624:0.36602299450128100711)80:0.14806433648677358450)8:0.06618116192197
928682)13:0.04583249222349643909)88:0.17718191114326001245,Bacteria-
Psychrobacter_sp._148653459:0.70758204367197308216)95:0.27724560785222818193)28:0.06195073067410042378,Bacteria-
Alteromonadales_bacterium_119468983:0.48263779465046124928);

Plantae-Arabidopsis_thaliana_18396215

(((Cyanobacteria-Nostoc_punctiforme_PCC_73102_23128764:0.02540840998443057164,(Cyanobacteria-
Nodularia_spumigena_CCY9414_119513395:0.04693748624034605604,(Cyanobacteria-
Nostoc_sp._PCC_7120_17231883:0.00000121823947223981,Cyanobacteria-
Anabaena_variabilis_ATCC_29413_75909491:0.00000121823947223981)100:0.07157307212745864233)54:0.0234143771833
0478118)93:0.06212514429610464101,(Cyanobacteria-
Trichodesmium_erythraeum_IMS101_113475683:0.15539775430824781521,((((Cyanobacteria-
Synechococcus_sp._WH_5701_87300686:0.09989752547201567745,(((Cyanobacteria-
Prochlorococcus_marinus_str._MIT_9313_33864147:0.00000121823947223981,Cyanobacteria-
Prochlorococcus_marinus_str._MIT_9303_124024202:0.00414179747575596415)100:0.06145940441408788424,(Cyanobacteri
a-Synechococcus_sp._CC9311_113955167:0.02179560049938040686,(((Cyanobacteria-
Synechococcus_sp._WH_7805_88808064:0.00829899713397501231,Cyanobacteria-
Synechococcus_sp._WH_7803_148238604:0.00000121823947223981)78:0.01387624601312129578,Cyanobacteria-
Synechococcus_sp._RS9917_87123059:0.02351766524235401543)54:0.00610634724521529493,((Cyanobacteria-
Synechococcus_sp._RCC307_148243388:0.02116438146653241981,((Cyanobacteria-
Synechococcus_sp._CC9902_78183830:0.00857705360368886741,Cyanobacteria-
Synechococcus_sp._BL107_116071561:0.00409901212491606444)96:0.02465369429247122809,(Cyanobacteria-
Synechococcus_sp._WH_8102_33864761:0.00421598627739433896,Cyanobacteria-
Synechococcus_sp._CC9605_78211771:0.00000121823947223981)99:0.00639730816989434424)97:0.03018682474847673644
)73:0.02050153578436885934,Cyanobacteria-
Synechococcus_sp._RS9916_116075574:0.00711955297964624034)78:0.03197060913172927038)28:0.00871143910161011233
3)54:0.02952648699433812335)27:0.01206794904115603834,((Cyanobacteria-
Prochlorococcus_marinus_subsp._marinus_str._CCMP1375_33239766:0.04667833385539917751,Cyanobacteria-
Prochlorococcus_marinus_str._MIT_9211_84518644:0.02972853241877811831)31:0.02651155480725414862,((Cyanobacteria-
Prochlorococcus_marinus_str._MIT_9515_123965550:0.02481557939306060223,(Cyanobacteria-
Prochlorococcus_marinus_str._MIT_9312_78778669:0.00417799876902441667,(Cyanobacteria-
Prochlorococcus_marinus_str._MIT_9301_126695644:0.00410032809879689979,(Cyanobacteria-
Prochlorococcus_marinus_str._AS9601_123967842:0.01240883764269817315,Cyanobacteria-
Prochlorococcus_marinus_subsp._pastoris_str._CCMP1986_33860840:0.00820810815413561788)27:0.00000121823947223981
)63:0.01233566584379889794)38:0.00000121823947223981)100:0.08901062363596845550,(Cyanobacteria-
Prochlorococcus_marinus_str._NATL1A_124025075:0.00000121823947223981,Cyanobacteria-
Prochlorococcus_marinus_str._NATL2A_72383484:0.00405068427533424123)100:0.04379216006327996819)13:0.003202326
32416135414)29:0.02018008074927401738)96:0.06897702822831032476)99:0.12829535702449745838,((((Chlamydia-
Candidatus_Protochlamydia_46446786:0.16337078849605821129,(((Chromalveolata-
Plasmodium_falciparum_86171049:0.61970692758041512338,((((Chromalveolata-
Phaeodactylum_tricornutum_10068:0.04918917661558808163,Chromalveolata-
Thalassiosira_pseudonana_32860:0.08496171824524567651)93:0.07919796982517976480,Chromalveolata-
Aureococcus_anophagefferens_19845:0.13895462801302976841)44:0.02310185586525940679,(((Chromalveolata-

Toxoplasma_gondii_122921324:0.00000121823947223981,Chromalveolata-
Toxoplasma_gondii_34555736:0.00000121823947223981)42:0.00000121823947223981,Chromalveolata-
Toxoplasma_gondii_122921325:0.00000121823947223981)83:0.00000121823947223981,(Chromalveolata-
Toxoplasma_gondii_122921416:0.00000121823947223981,Chromalveolata-
Toxoplasma_gondii_122921415:0.00000121823947223981)89:0.00000121823947223981)100:0.21231400470078662379)35:0.
03325626798020533265,Chromalveolata-
Isochrysis_galbana_ISE00008579_3:0.17576477359389086619)72:0.04522977928493889660)56:0.07038451240073305415,(((
Chlamydia-Chlamydophila_pneumoniae_15835939:0.00000121823947223981,(Chlamydia-
Chlamydophila_pneumoniae_16752634:0.00000121823947223981,Chlamydia-
Chlamydophila_pneumoniae_15618321:0.00000121823947223981)18:0.00000121823947223981)17:0.00000121823947223981
,Chlamydia-Chlamydophila_pneumoniae_33241753:0.00000121823947223981)100:0.03176415355419537345,(((Chlamydia-
Chlamydia_trachomatis_15604823:0.00000121823947223981,Chlamydia-
Chlamydia_trachomatis_76788818:0.00000121823947223981)100:0.02961182953488699851,Chlamydia-
Chlamydia_muridarum_15834999:0.01295876598059629917)99:0.05929253405135464366,((Chlamydia-
Chlamydophila_caviae_29840151:0.01265364044753804207,Chlamydia-
Chlamydophila_felis_89898427:0.03025589395702240475)91:0.01801872717670233506,Chlamydia-
Chlamydophila_abortus_62185007:0.05655972433193984239)97:0.06508756637043974780)85:0.03776495357122770097)100:
0.20171157171354983650)20:0.02811652168608399713,(((Rhizaria-
Bigelowiella_natans_DR039063_2:0.25620725527644910846,(Plantae-
Volvox_carteri_83516_jgi:0.01822610628035757629,Plantae-
Chlamydomonas_reinhardtii_122688:0.14833769446872024922)100:0.11934457256455030527)38:0.04993565459775249626,(
Plantae-Ostreococcus_tauri_28591:0.04881814640159715113,Plantae-
Ostreococcus_lucimarinus_32790:0.05189545094247557250)77:0.09012319412959035603)75:0.05556156337460863709,(((Pla
ntae-Arabidopsis_thaliana_18396215:0.00000121823947223981,Plantae-
Arabidopsis_thaliana_30678246:0.00000121823947223981)100:0.08035770246531336036,(Plantae-
Oryza_sativa_115475922:0.04179305416702477211,Plantae-
Oryza_sativa_115478314:0.03780659648150160729)100:0.04913814936907244524)93:0.05191943259660181109,(Plantae-
Physcomitrella_patens_119545:0.02097883214970002602,Plantae-
Physcomitrella_patens_132935:0.05455306825412724325)99:0.10125980276020535298)49:0.05280653155856844527)39:0.06
077693332847258767)19:0.03007289280841004067)100:0.93367202953851091696,Cyanobacteria-
Gloeobacter_violaceus_PCC_7421_37523757:0.24090518768696242424)71:0.10888549459182697565,(((Bacteria-
Mariprofundus_ferrooxydans_114777289:0.41077748011727549526,(Bacteria-
Pelobacter_carbinolicus_77919640:0.18453849833715627038,Bacteria-
Desulfuromonas_acetoxidans_95931158:0.14465928905010314032)100:0.19117951849248454588)84:0.11617282637660422684,(((Bacteria-Desulfovibrio_desulfuricans_78356053:0.17293987426097581772,Bacteria-
Lawsonia_intracellularis_94986794:0.25641129229123305056)100:0.20992821410926038039,(((Bacteria-
Hydrogenobaculum_sp._156718930:0.44308381292970594467,((Bacteria-
Xanthobacter_autotrophicus_154247184:0.08899021576123349042,Bacteria-
Methylobacterium_sp._149123892:0.16828204094846005434)100:0.32021249546515673234,(((Bacteria-
Dechloromonas_aromatica_71906617:0.47885600089877627950,Bacteria-
Azoarcus_sp._119898421:0.45323699647875009822)70:0.12864607051564394546,(Bacteria-
Pseudomonas_stutzeri_146281083:0.40394171426392888602,Bacteria-
Acidovorax_avenae_120610730:0.20768416936675748730)59:0.11794035006268234478)20:0.04105760498038270312,Bacteri
a-
Polaromonas_naphthalenivorans_121583099:0.42617390655410308797)19:0.08008715839716418206)100:0.26364788741117389792)26:0.07820060051687605096,Bacteria-
Marinobacter_aquaeolei_120554462:0.48739358138628097183)11:0.05606549440525675221,(((Bacteria-
Parvibaculum_lavamentivorans_154251242:0.18712512240739823111,(((Bacteria-
Rhodospirillum_rubrum_83593956:0.21082055038932612234,Bacteria-
Stappia_aggregata_118591455:0.37886130756792757435)35:0.05299320987899178159,((Bacteria-
Roseobacter_denitrificans_110680382:0.10241314965644765511,(Bacteria-
Sagittula_stellata_126731271:0.10849292099378009258,Bacteria-
Paracoccus_denitrificans_119383609:0.16191479278839909783)28:0.02108542324305436727)21:0.03390228854904950295,B
acteria-
Oceanicola_granulosus_89068843:0.07694270728322624708)99:0.11745882535330244567)22:0.03113415607887919101,((Ba
cteria-Fulvimarina_pelagi_114707939:0.18152393785070353172,(Bacteria-
Agrobacterium_tumefaciens_15888100:0.08779223047778250688,Bacteria-
Sinorhizobium_medicae_150395736:0.04407704906801849370)78:0.05025921188593035493)82:0.04135879304209685858,(B
acteria-Mesorhizobium_sp._110633084:0.14051193844729337190,((Bacteria-
Rhodopseudomonas_palustris_39934274:0.03531692871355492785,Bacteria-
Bradyrhizobium_japonicum_27377740:0.09861852607543579452)73:0.06833937435580207664,(Bacteria-
Bartonella_bacilliformis_121602192:0.14153695913375449744,(Bacteria-

Brucella_ovis_148560363:0.06104753643965380588,(Bacteria-
Aurantimonas_sp._90420122:0.08151832197158602467,Bacteria-
Rhizobium_leguminosarum_116250784:0.13665089123149587458)32:0.01439833633565386177)69:0.03142820965915884418
)88:0.05277328537895614041)40:0.03564079764915382281)30:0.05164538394211808797)39:0.03783898993194863281)8:0.0
3456693713025703329)47:0.11265609501373421986,(Bacteria-
Neorickettsia_sennetsu_88608759:0.41864681633346945144,Bacteria-
Rickettsia_typhi_51473557:0.37939803076121841885)89:0.14611645541024237405)89:0.11491494483421439698,((Bacteria-
Caminibacter_mediatlanticus_149195105:0.24977140438977446513,(((Bacteria-
Helicobacter_pylori_108562614:0.26466023624305323736,Bacteria-
Wolinella_succinogenes_34556852:0.15208995923216447888)73:0.07428770334690004695,(Bacteria-
Campylobacter_concisus_157165165:0.20783752687148918104,Bacteria-
Thiomicrospira_denitrificans_78777945:0.20681786271061863847)39:0.04942443732218210900)12:0.01108542466850900507
,Bacteria-
Nitratiruptor_sp._152991231:0.17365031074249559495)51:0.04806496627699624602)51:0.03888669766465757682,(Bacteria-
Arcobacter_butzleri_157738335:0.08311246958133997054,Bacteria-
Sulfurovum_sp._152992312:0.12505318866504075426)100:0.16095539254956800490)100:0.12430168045168539515)64:0.11
732900516960656501)4:0.03492023840386650896)8:0.08406188893946466012,(Bacteria-
Roseiflexus_castenholzii_156743345:0.31680546784790902803,Bacteria-
Herpetosiphon_aurantiacus_113941789:0.35386540440437497246)81:0.11341540949648599990)10:0.02437647384829337918
)25:0.04932625084812038152,((Bacteria-Solibacter_usitatus_116626829:0.66876424295153757971,(Bacteria-
Clostridium_thermocellum_125975647:0.37035637792622921882,Bacteria-
Bacillus_cereus_152974732:0.24186995134671498975)55:0.08603860691376270797)49:0.06822893204737276451,((Bacteria-
Symbiobacterium_thermophilum_51894063:0.38798731957477400334,Bacteria-
Acidobacteria_bacterium_94970306:0.40489968834979112033)41:0.10680188692177813858,(Chromalveolata-
Phytophthora_sojae_137179:0.78194449123160780779,(Bacteria-
Thermus_thermophilus_55980273:0.14640245206807839251,Bacteria-
Deinococcus_geothermalis_94984368:0.38734639748929394765)100:0.23934277478989898769)52:0.12734765431030692362)
11:0.05371562840229358177)12:0.03737829814457056116)70:0.21532432345978200217)82:0.05683301241276633720,(Plant
ae-Cyanidioschyzon_merolae_CMT381C:0.35319921182040470153,(Cyanobacteria-Synechococcus_sp._JA-2-3Ba2-
13_86608636:0.04471214140026957828,Cyanobacteria-Synechococcus_sp._JA-3-
3Ab_86606338:0.00682148272663900788)100:0.20363200856994465027)54:0.05066815307418634506)64:0.07151179792420
844783)66:0.06774363298887724205,(Cyanobacteria-Thermosynechococcus_elongatus_BP-
1_22299236:0.18868652259008844574,(Cyanobacteria-
Synechococcus_elongatus_PCC_6301_56751387:0.00408948712054983156,Cyanobacteria-
Synechococcus_elongatus_PCC_7942_81298937:0.00000121823947223981)100:0.12656204545728938493)29:0.02617067882
961570183)51:0.07361557159420491214,Cyanobacteria-
Lyngbya_sp._PCC_8106_119484731:0.10590206647860976596)29:0.03446282396603933540)57:0.02489384414661652625)9
2:0.07028953732532042253,(Cyanobacteria-
Crocosphaera_watsonii_WH_8501_67925372:0.05178250662210150818,Cyanobacteria-
Cyanothece_sp._CCY0110_126660445:0.03656608456474170349)100:0.09194920224135406939,Cyanobacteria-
Synechocystis_sp._PCC_6803_16329628:0.18995741883595176902);

Plantae-Arabidopsis_thaliana_18396238
(Plantae-Physcomitrella_patens_190433:0.15640942236438115653,(Plantae-
Physcomitrella_patens_59338:0.24795282304051152744,(((Plantae-
Oryza_sativa_115470080:0.46857360174914158835,Plantae-
Arabidopsis_thaliana_15235543:0.55276826155804759111)100:0.37747075546528852108,((Chlamydia-
Candidatus_Protochlamydia_46446753:1.90303196891456072315,((Bacteria-
Granulibacter_bethesdensis_114326726:0.96142292501717496744,Bacteria-
Methylobacterium_extorquens_153898673:0.89811491112473584852)100:0.96064971849082103628,Bacteria-
Sinorhizobium_medicae_150377140:1.37450964762918270701)87:0.44052746483474425343)100:0.86128985204082031402,((
Plantae-Arabidopsis_thaliana_15237478:0.10352357494798256066,Plantae-
Arabidopsis_thaliana_15232143:0.08524038286161485234)100:0.24003594856780041678,(Plantae-
Oryza_sativa_115474573:0.26890684379749246746,Plantae-
Arabidopsis_thaliana_18396238:0.42874950584178045387)25:0.06680508516101099636)92:0.25556573479354749434)65:0.1
1939211311018980977)98:0.36048037086754980418,Plantae-
Physcomitrella_patens_16888:0.26371659511798656306)49:0.03984845765614593305)89:0.10411001295498471808,Plantae-
Physcomitrella_patens_151210:0.15673619512293132661);

Plantae-Arabidopsis_thaliana_18408421
(Bacteria-Haemophilus_influenzae_145629620:0.06653823201336148130,((Bacteria-
Escherichia_coli_110642447:0.02118545810239788954,(((Bacteria-

Serratia_proteamaculans_157368448:0.03829123025908183486,Bacteria-
Yersinia_enterocolitica_123440604:0.03209746320738906394)93:0.04290337347293807918,Bacteria-
Klebsiella_pneumoniae_152971184:0.03836899174635374082)34:0.02108654205982908592,Bacteria-
Enterobacter_sakazakii_156933153:0.03767943841841138453)49:0.01945635484054130879)98:0.06872090156310543818,(((((
Bacteria-Bacillus_weihenstephanensis_89206181:0.23257794606691847838,((Bacteria-
Clavibacter_michiganensis_148271582:0.63691928626787153167,((Bacteria-
Shigella_flexneri_31983644:0.39013501891069990446,Bacteria-
Streptococcus_agalactiae_22537582:0.28393601173917104585)100:0.65903420258654243913,(((Bacteria-
Photobacterium_profundum_90414501:0.02971803766137730285,(Bacteria-
Vibrio_parahaemolyticus_153836851:0.05235587909107222715,((Bacteria-
Citrobacter_koseri_157144301:0.00445028582277629675,Bacteria-
Salmonella_enterica_62182278:0.00544477609609505826)99:0.08287704604425116806,Bacteria-
Aeromonas_salmonicida_145298404:0.12256012531519731856)92:0.07407466678307651764)64:0.03826932539852875154)1
00:0.40855335509100554336,(Bacteria-Staphylococcus_aureus_148266646:0.32614436970276561301,(Bacteria-
Actinomyces_odontolyticus_154508774:0.71946979680990563732,Bacteria-
Listeria_monocytogenes_153173750:0.51628866054027966470)25:0.04776013395363205849)70:0.18995821335873971925)10
0:0.88853870595913175823,((Bacteria-Bacteroides_thetaiotaomicron_29345606:1.20240280703199808876,((((Chromalveolata-
Phaeodactylum_tricornutum_43611:0.12724988066883155535,Chromalveolata-
Thalassiosira_pseudonana_22565:0.19631438881616583569)100:0.49214800781393602813,Chromalveolata-
Aureococcus_anophagefferens_71036:0.83756561543665430936)100:0.71496707328919306690,((((Opisthokonta-
Mus_musculus_23346457:0.37597234667887208248,(Opisthokonta-
Danio_rerio_47087636:0.21285387306718642209,Opisthokonta-
Mus_musculus_31560349:0.16864264472495366087)70:0.07411668351738937011)100:0.18914589348062280094,(Opisthoko
nta-Apis_mellifera_66530901:0.34947612263891669748,(Opisthokonta-
Drosophila_melanogaster_24656866:0.00000121823947223981,(Opisthokonta-
Drosophila_melanogaster_45550478:0.00000121823947223981,Opisthokonta-
Drosophila_melanogaster_24656861:0.00000121823947223981)35:0.00000121823947223981)100:0.39634424261535999845)9
9:0.18115003965919260209)100:0.17968863463687559379,((Plantae-
Chlamydomonas_reinhardtii_144519:0.11462530840594391257,Plantae-
Volvox_carteri_57773_jgi:0.09874179188240254923)100:0.43434724901742105541,((Plantae-
Ostreococcus_lucimarinus_29537:0.07500620986956044178,Plantae-
Ostreococcus_tauri_21081:0.12767938233270298420)100:0.32415714962264430277,((Plantae-
Oryza_sativa_115447643:0.42714405775676106414,Plantae-
Arabidopsis_thaliana_30684088:0.24658304588852295036)68:0.08025470457868587315,((Plantae-
Physcomitrella_patens_55740:0.11151566347366353993,Plantae-
Physcomitrella_patens_2560:0.06170822126694196219)100:0.24121518783443821055,((Plantae-
Oryza_sativa_115466748:0.41319151344048848928,Plantae-
Arabidopsis_thaliana_30678883:0.28190945987452764587)100:0.27165051086213742870,((Plantae-
Arabidopsis_thaliana_15234973:0.08029528656276516130,Plantae-
Arabidopsis_thaliana_15221441:0.09960356129931847990)100:0.30369591483430274925,((Plantae-
Oryza_sativa_115459830:0.18611712887756531876,Plantae-
Arabidopsis_thaliana_18408421:0.18623263876559847940)54:0.05860304294957225518,Plantae-
Oryza_sativa_115474855:0.25883104351587626901)65:0.04711236215661595422)89:0.07754669538778545534)26:0.0608160
0221166774678)17:0.03443480104949405568)24:0.07975247288432314730)29:0.07772775084275461432)63:0.125490402584
31556905)97:0.39935889927630768836,(((Plantae-Ostreococcus_tauri_12406:0.60624901770302497273,Plantae-
Ostreococcus_lucimarinus_31082:0.49160331517587307903)100:1.66450182068097141119,((Chromalveolata-
Tetrahymena_thermophila_118394637:0.46457232087311606250,Chromalveolata-
Tetrahymena_thermophila_118354920:0.47862669120732703743)100:0.33141116044511786942,((Chromalveolata-
Tetrahymena_thermophila_118401808:1.02241515201331401030,Chromalveolata-
Tetrahymena_thermophila_118366967:0.50692558850694768857)62:0.13277474336830799584,((Chromalveolata-
Tetrahymena_thermophila_118400994:0.62871284255880632852,Chromalveolata-
Tetrahymena_thermophila_118375659:0.76229833926369827690)100:0.68586797811350652676,((Chromalveolata-
Paramecium_tetraurelia_124426426:0.43379016704517803227,(Chromalveolata-
Paramecium_tetraurelia_124416855:0.00000121823947223981,(Chromalveolata-
Paramecium_tetraurelia_124403291:0.11322397919870355598,Chromalveolata-
Paramecium_tetraurelia_124429506:0.35178678434524918162)93:0.04701885434627032373)100:0.43985016372029517306)1
00:0.82945638359480711177,(Chromalveolata-Paramecium_tetraurelia_124427774:0.04204324704649028271,Chromalveolata-
Paramecium_tetraurelia_124407176:0.00000121823947223981)100:1.13003416330478367868)32:0.11607222797858406105)2
1:0.08394009736244534281)34:0.09571697333926036666)100:0.47760196243252484027)19:0.06618454002264384517,(Opist
hokonta-Mus_musculus_71143150:0.65259355456303957954,Chromalveolata-
Phytophthora_sojae_139174:1.04281679759789258632)91:0.22846033213182184718)34:0.06976397143262301481)61:0.1972
825927248312571 9)90:0.36666094469618715213,((Plantae-Ostreococcus_tauri_19202:0.14581342002739303143,Plantae-

Ostreococcus_lucimarinus_94723:0.0541415363438216071 2)100:1.2992569190895628672 4,(Bacteria-
Pseudomonas_aeruginosa_116049513:1.3870028493591040064 1,(Bacteria-
Burkholderia_pseudomallei_126443546:0.3477050457510928449 5,Bacteria-
Stigmatella_aurantiaca_115377967:0.6169809367802996380 4)100:1.9272358902077837417 0)22:0.1037598872837460772 8)14
:0.0817456054538927001 7)98:0.3968998986394797756 6)88:0.2342603704357922589 4,(Plantae-
Ostreococcus_lucimarinus_30179:1.1007449318596587151 1,(Chlamydia-
Candidatus_Protochlamydia_46446021:0.3346996238289726144 4,((Chlamydia-
Chlamydia_muridarum_15835445:0.0335449963783232135 7,(Chlamydia-
Chlamydia_trachomatis_76789281:0.0000012182394722398 1,Chlamydia-
Chlamydia_trachomatis_15605273:0.0000012182394722398 1)100:0.0242697830747013523 8)100:0.1061074756479681857 6,(((
(Chlamydia-Chlamydophila_abortus_62184720:0.0278579613280132351 6,Chlamydia-
Chlamydophila_caviae_29839842:0.0318854346440458902 3)87:0.0164982930443693318 1,Chlamydia-
Chlamydophila_felis_89898737:0.0140650734017526684 4)68:0.0417769989581850409 4,((Chlamydia-
Chlamydophila_pneumoniae_16752375:0.0000012182394722398 1,Chlamydia-
Chlamydophila_pneumoniae_15618575:0.0000012182394722398 1)20:0.0000012182394722398 1,(Chlamydia-
Chlamydophila_pneumoniae_15836197:0.0000012182394722398 1,Chlamydia-
Chlamydophila_pneumoniae_33242022:0.0000012182394722398 1)18:0.0000012182394722398 1)100:0.1433617450913029778
3)30:0.0317786259407157184 5)100:0.2344191538168872157 6)82:0.2214215301847554384 6)98:0.2068514022062528934 2)60
:0.1329628412032977380 4)77:0.1219806289003062677 0)57:0.1103909112597823150 0)74:0.1935092835567323310 6,((Bacteri
a-Clostridium_novyi_118444722:0.1875491462019928390 3,Bacteria-
Francisella_tularensis_110670313:0.7004231905133679880 7)82:0.1408619707847103275 1,(Bacteria-
Leptospira_borgpetersenii_116329394:0.9120111541084940931 5,Bacteria-
Thermosinus_carboxydivorans_121535100:0.2178511166087121753 2)64:0.1697449717570639060 6)49:0.1010510388551150 3
015)65:0.0919814166214995426 7)99:0.1497554363632241647 4,Bacteria-
marine_gamma_119477676:0.3654809578344302978 3)89:0.1020614491846681681 8,Bacteria-
Pseudoalteromonas_atlantica_109899603:0.2025815434374630408 2)69:0.0626687642474199613 2,(Bacteria-
Moritella_sp._149909897:0.0872894606282467455 7,Bacteria-
Vibrionales_bacterium_148981435:0.0751920017391270040 2)100:0.0929763709636019231 0)87:0.1176730158064778347 7,Ba
cteria-
Chromobacterium_violaceum_34495708:0.1587744099153833854 4)78:0.0892569083086196396 5)100:0.4310343614023440 23
96,Bacteria-Actinobacillus_succinogenes_152978273:0.0948762654005818884 0);

Plantae-Arabidopsis_thaliana_18410414
(((Plantae-Volvox_carteri_59132_jgi:0.1895798695089708496 6,Plantae-
Chlamydomonas_reinhardtii_196478:0.0999939226132776914 9)100:0.4562413156762034627 3,(Plantae-
Ostreococcus_tauri_20960:0.1742994172081318293 7,Plantae-
Ostreococcus_lucimarinus_88889:0.1186067482826388824 7)100:0.6483218700945138079 9)100:0.1414974604191386597 9,(Pl
antae-Oryza_sativa_115464401:0.3218489592431498791 4,Plantae-
Arabidopsis_thaliana_18410414:0.3626890914669975707 3)100:0.3541714770120552024 9,Chlamydia-
Candidatus_Protochlamydia_46445958:1.0708061045479344564 1);

Plantae-Arabidopsis_thaliana_18418270
(((Chlamydia-Chlamydia_muridarum_15835284:0.1128690177569069003 1,(Chlamydia-
Chlamydia_trachomatis_15605115:0.0000012182394722398 1,Chlamydia-
Chlamydia_trachomatis_76789121:0.0000012182394722398 1)100:0.1083024636802465828 8)100:0.2874643705556905182 2,(((
(Chlamydia-Chlamydophila_pneumoniae_15618406:0.0000012182394722398 1,Chlamydia-
Chlamydophila_pneumoniae_16752548:0.0000012182394722398 1)20:0.0000012182394722398 1,(Chlamydia-
Chlamydophila_pneumoniae_15836026:0.0000012182394722398 1,Chlamydia-
Chlamydophila_pneumoniae_33241846:0.0000012182394722398 1)20:0.0000012182394722398 1)100:0.4346514310013811499
5,(((Bacteria-Ralstonia_pickettii_153888148:0.6052664026837066835 2,Bacteria-
Limnobacter_sp._149927771:0.4799632866055167923 5)100:1.0624232764316050570 1,((Bacteria-
Bacillus_amyloliquefaciens_154687887:0.5541902930201217936 1,((Bacteria-
Listeria_welshimeri_116871680:0.2440057732783318666 9,((Bacteria-
Streptococcus_thermophilus_116627354:0.2766668868159891658 8,Bacteria-
Lactobacillus_reuteri_92088613:0.1889861702644027308 1)93:0.0758737736504388055 0,Bacteria-
Leuconostoc_mesenteroides_116617192:0.2573544944901201003 7)97:0.1445643045241831736 7)80:0.1061214124250672158
3,(Plantae-Physcomitrella_patens_158207:0.3435633776857797694 3,Bacteria-
Geobacillus_thermodenitrificans_138894510:0.1832706124974635697 5)44:0.0498238738506426165 3)89:0.2144589714570159
2335)100:0.2505650725513598464 6,((((Bacteria-Hyphomonas_neptunium_114798447:0.2328759329495512231 4,((Bacteria-
Sphingomonas_sp._94495925:0.0734309836718441411 0,Bacteria-
Sphingopyxis_alaskensis_103485999:0.0190404433767828451 5)100:0.0581629518048196944 6,Bacteria-
Erythrobacter_sp._149186438:0.1342361006908414644 6)100:0.2196867936683134237 0)45:0.0572092737979436744 0,((((Bact

eria-Ochrobactrum_anthropi_153009246:0.00304721932744037808,Bacteria-
Brucella_ovis_148559151:0.01346026943012510087)100:0.06813354802632920482,(Bacteria-
Mesorhizobium_sp._110634273:0.07604149724246531128,(Bacteria-
Sinorhizobium_medicae_150396667:0.02582763243016675259,(Bacteria-
Rhizobium_etli_86357503:0.02337920211642816049,Bacteria-
Agrobacterium_tumefaciens_15888903:0.04810837956737772092)47:0.00997903725951900140)99:0.05254369616000410803)
40:0.01269977465345626354)95:0.03787499766756535075,(Bacteria-
Fulvimarina_pelagi_114707058:0.14720327355068787978,Bacteria-
Aurantimonas_sp._90419167:0.10064102201326625974)90:0.07014106560769148291)98:0.07638016685821825968,(Bacteria-
Methylobacterium_sp._149120131:0.11406549455914069546,Bacteria-
Xanthobacter_autotrophicus_154247450:0.09878465085876987628)98:0.07902218361536665625)73:0.045878992075247537
5)100:0.57896874442004453432,((Cyanobacteria-Synechococcus_sp._JA-2-3Ba2-
13_86608738:0.03516127173559494695,Cyanobacteria-Synechococcus_sp._JA-3-
3Ab_86605900:0.04096926311841735113)100:0.13641714058080869898,(((Cyanobacteria-
Nostoc_punctiforme_PCC_73102_23128688:0.02813013312242954034,(Cyanobacteria-
Nodularia_spumigena_CCY9414_119512462:0.03451262388571309930,(Cyanobacteria-
Anabaena_variabilis_ATCC_29413_75906242:0.00395355093503886416,Cyanobacteria-
Nostoc_sp._PCC_7120_17230257:0.00452886188780086876)95:0.04843346429051389318)54:0.00796305573241419713)86:0.
05056009442392113418,(Cyanobacteria-Lyngbya_sp._PCC_8106_119486833:0.14698275535155866378,Cyanobacteria-
Trichodesmium_erythraeum_IMS101_113474811:0.11992170670164060964)85:0.06482162266957666730)81:0.057989449605
47589705,(Cyanobacteria-Synechocystis_sp._PCC_6803_16331407:0.21108740017702731784,Cyanobacteria-
Thermosynechococcus_elongatus_BP-
1_22299593:0.13490526294506530181)61:0.04776289229673618547)73:0.08477944057344873052)100:0.4558451882360454
1771)49:0.08276446361473499547,(((Bacteria-Syntrophus_aciditrophicus_85857917:0.51052802473844893782,Bacteria-
Caldicellulosiruptor_saccharolyticus_146295811:0.82201784076075501417)39:0.15610667376105347159,(Bacteria-
Halothermothrix_orenii_89209988:0.37186191229289267124,(Bacteria-
Dehalococcoides_ethenogenes_57234493:0.45040573816028439680,(Bacteria-
Moorella_thermoacetica_83589740:0.22259119593898130107,((Bacteria-
Desulfotomaculum_reducens_134299551:0.17550310147258932347,Bacteria-
Pelotomaculum_thermopropionicum_147678134:0.21976732253639397197)99:0.09775546549026400966,Bacteria-
Carboxydothermus_hydrogenoformans_78045045:0.17380665777590273424)89:0.06390275750998379578)87:0.106258773686
17266117)46:0.05492408018799152525)29:0.03962970816055593620)12:0.06793078444726424747,((Bacteria-
Syntrophobacter_fumaroxidans_116747505:0.34732790052928169677,(Bacteria-
Hydrogenobaculum_sp._156719834:0.35102841685574742714,Bacteria-
Desulfovibrio_desulfuricans_78357013:0.35526515108919448638)77:0.10423867490385485546)75:0.10196237913547676057,
(Cyanobacteria-Gloeobacter_violaceus_PCC_7421_37523677:0.23666004188704267697,(((Cyanobacteria-
Nostoc_sp._PCC_7120_17231819:0.01740227809971516340,Cyanobacteria-
Anabaena_variabilis_ATCC_29413_75907500:0.02462924263473174197)100:0.12505096834761558755,Cyanobacteria-
Lyngbya_sp._PCC_8106_119485467:0.21395542985648205447)59:0.04830586689070065498,Cyanobacteria-
Trichodesmium_erythraeum_IMS101_113474355:0.28006644037084887922)57:0.06065215776167971751)100:0.28796819589
819228069)25:0.03119565438758615736)46:0.11501974917941086562)53:0.13960689180883412153)100:0.305356460161579
85683)100:0.75605353189072588282,((Chlamydia-
Candidatus_Protochlamydia_46446319:0.42159674968331278810,((Plantae-
Cyanophora_paradoxa_Contig1999_6:0.26402102811614563960,((((Plantae-
Arabidopsis_thaliana_18397344:0.34874849153542636371,Plantae-
Oryza_sativa_115451309:0.23971587250950568415)90:0.11871881145627954934,((Plantae-
Arabidopsis_thaliana_18418270:0.10561601148804122818,Plantae-
Oryza_sativa_115452503:0.06784274787907063065)90:0.06055011781241657204,Plantae-
Physcomitrella_patens_121808:0.15327817658785122812)43:0.05263960734900963861)90:0.09601474839891313562,((Planta
e-Ostreococcus_tauri_22755:0.14357988479963260109,Plantae-
Ostreococcus_lucimarinus_30412:0.02904085644726243920)100:0.28105450486397653975,(Plantae-
Volvox_carteri_81843_jgi:0.00455270255117826150,Plantae-
Chlamydomonas_reinhardtii_129557:0.05406560458219782095)100:0.17120355905850700995)71:0.07893976310662341456)
73:0.09672178824793237206,(Plantae-Galdieria_sulphuraria_A4_33D11_2:0.30116543806236212300,(Plantae-
Cyanidioschyzon_merolae_CMN323C:0.24364224565621231400,(((Chromalveolata-
Aureococcus_anophagefferens_36703:0.23809498795533473592,(Chromalveolata-
Phaeodactylum_tricornutum_22909:0.11928928571552679938,Chromalveolata-
Thalassiosira_pseudonana_31394:0.09415822499347728525)96:0.06938981530520774155)93:0.08644121826098324912,Chro
malveolata-Pavlova_lutheri_Contig338_2:0.17648650351345862575)48:0.02940724008559220809,Chromalveolata-
Laminaria_digitata_62990747_2:0.20428412716334504173)61:0.07288140123472465481)48:0.05518410698349311000)48:0.0
5755281137168213879)34:0.02664353097188303446)33:0.05741503363498230011,Chromalveolata-
Guillardia_theta_57335937:0.35899138540886799120)28:0.03052495186889479101)92:0.18814019699833464672,((Cyanobact

eria-Lyngbya_sp._PCC_8106_119488916:0.32240503571154860429,Bacteria-
Beggiatoa_sp._153868447:0.44302818981955832678)100:0.39634899006036439228,((Bacteria-
Desulfotalea_psychrophila_51246017:0.44347827828464958522,Bacteria-
Candidatus_Desulfococcus_121542099:0.27461605626297691529)95:0.18291457964671536951,(((Bacteria-
Parabacteroides_merdae_154492519:0.30956465965836943433,(Bacteria-
Desulfitobacterium_hafniense_109648263:0.20468179086534643574,(Bacteria-
Lawsonia_intracellularis_94986878:0.41410117604357610244,Bacteria-
Thermosinus_carboxydivorans_121535392:0.09481365820503168118)24:0.02992687889355696157)49:0.05297754312121435
299)77:0.09201522735382879070,(Bacteria-Bacteroides_capillosus_154499894:0.26845924561684997300,(Bacteria-
Ruminococcus_obeum_153813418:0.14910973874028904396,(Bacteria-
Dorea_longicatena_153852917:0.15716351163519687306,(Bacteria-
Eubacterium_ventriosum_154483444:0.08625666668629079770,Bacteria-
Clostridium_phytofermentans_106886313:0.13309938067151955154)92:0.06786035727127107120)53:0.053947341376302276
88)100:0.19431987158248587022)59:0.05899925967733590793)37:0.02577332942268668534,(Bacteria-
Leptospira_interrogans_24213476:0.31745739153641644448,((Bacteria-
delta_proteobacterium_94264240:0.15049955658835059702,(Bacteria-
Desulfuromonas_acetoxidans_95928626:0.12039808835545773302,(Bacteria-
Geobacter_metallireducens_78221437:0.03991316121051127763,Bacteria-
Pelobacter_propionicus_118581464:0.05304648365561217410)100:0.13321266328566971904)100:0.07385151492721585276)
100:0.13983369718362939627,((((((Cyanobacteria-
Prochlorococcus_marinus_str._MIT_9301_126697029:0.02018770252388078432,(Cyanobacteria-
Prochlorococcus_marinus_str._AS9601_123969235:0.01849443900149560366,Cyanobacteria-
Prochlorococcus_marinus_str._MIT_9312_78779976:0.03434500354498570829)60:0.01535654674521491625)98:0.059422560
20165586489,(Cyanobacteria-Prochlorococcus_marinus_str._MIT_9515_123966913:0.07544315290315180278,Cyanobacteria-
Prochlorococcus_marinus_subsp._pastoris_str._CCMP1986_33862056:0.06408891906468694444)97:0.07470422144668970033
)100:0.18537332282305374331,(Cyanobacteria-
Prochlorococcus_marinus_str._NATL1A_124026646:0.01567209032683201941,Cyanobacteria-
Prochlorococcus_marinus_str._NATL2A_72382904:0.00000121823947223981)100:0.13964372243172137877)53:0.073081684
88589160405,(Cyanobacteria-
Prochlorococcus_marinus_subsp._marinus_str._CCMP1375_33241104:0.14071267677664894835,Cyanobacteria-
Prochlorococcus_marinus_str._MIT_9211_84519195:0.15256591481074405214)33:0.01632373308435457887)42:0.051394495
95281309069,((((Cyanobacteria-Synechococcus_sp._CC9311_113953665:0.07109131763280038263,(Cyanobacteria-
Synechococcus_sp._RS9916_116075700:0.01989190536954008742,(Cyanobacteria-
Prochlorococcus_marinus_str._MIT_9303_124024065:0.00465166904201762092,Cyanobacteria-
Prochlorococcus_marinus_str._MIT_9313_33864058:0.00611533976538845229)100:0.10078966888866287832)91:0.02066429
813130424981)31:0.01394449819012867631,Cyanobacteria-
Synechococcus_sp._RS9917_87125031:0.06101175975978519184)57:0.01592884540015874692,((((Cyanobacteria-
Synechococcus_sp._WH_7805_88807953:0.02716316126622536631,Cyanobacteria-
Synechococcus_sp._WH_7803_148238706:0.00640076461091729741)93:0.01512300182080985461,((Cyanobacteria-
Synechococcus_sp._CC9902_78185598:0.01893897878584576908,Cyanobacteria-
Synechococcus_sp._BL107_116072121:0.00000121823947223981)100:0.07150270458340851620,Cyanobacteria-
Synechococcus_sp._CC9605_78211863:0.02409286176439429800)50:0.01011752069797824255)36:0.00704781700860726296
,Cyanobacteria-
Synechococcus_sp._WH_8102_33866679:0.03145389714278354343)81:0.02484217907952679036,Cyanobacteria-
Synechococcus_sp._RCC307_148243278:0.08538248035304515926)57:0.02192388750482839044)67:0.037283226372411597
26,Cyanobacteria-
Synechococcus_sp._WH_5701_87301876:0.08157749579407395923)89:0.04251358674225867024)73:0.09749044117269568
21,(((((((Cyanobacteria-Anabaena_variabilis_ATCC_29413_75908571:0.00245151993415336153,Cyanobacteria-
Nostoc_sp._PCC_7120_17232595:0.01340962637991552873)100:0.02829081904301637349,Cyanobacteria-
Nostoc_punctiforme_PCC_73102_23124300:0.04158792442309919984)60:0.00704675135319387299,Cyanobacteria-
Nodularia_spumigena_CCY9414_119512643:0.05008254645354308365)78:0.01778307930338187542,(((Cyanobacteria-
Cyanothece_sp._CCY0110_126657512:0.01871340883713615288,Cyanobacteria-
Crocosphaera_watsonii_WH_8501_67924273:0.04027601526937501369)100:0.06693816448466453906,Cyanobacteria-
Lyngbya_sp._PCC_8106_119492452:0.06545351579199092107)51:0.01581217472722076453,Cyanobacteria-
Trichodesmium_erythraeum_IMS101_113476813:0.08512184903681048465)52:0.01743485057434285626)48:0.024867971884
97471070,Cyanobacteria-
Synechocystis_sp._PCC_6803_16331785:0.13429233778580101921)73:0.03776410326642763021,(Cyanobacteria-
Synechococcus_elongatus_PCC_6301_56750696:0.00000121823947223981,Cyanobacteria-
Synechococcus_elongatus_PCC_7942_81299662:0.00000121823947223981)100:0.08688915080114852085)35:0.02676558616
371362692,((Cyanobacteria-Synechococcus_sp._JA-2-3Ba2-13_86608889:0.03319244105594772870,Cyanobacteria-
Synechococcus_sp._JA-3-3Ab_86607082:0.02089857985029158066)100:0.12268415747164117258,Cyanobacteria-
Thermosynechococcus_elongatus_BP-

1_22299645:0.11322229397739250190)64:0.03215189041027573941)98:0.06082438753904149953)99:0.12760756190197430904)59:0.03442984072646353144)38:0.03245678755226725359)14:0.01778812591844788904)100:0.18558674986589926426)93:0.13727883681503499913)59:0.09277449672389863344)100:0.43266438287476011171)51:0.11425924130929351885)91:0.12805783495200276367,(Chlamydia-Chlamydophila_abortus_62184887:0.12369303747148806261,Chlamydia-Chlamydophila_caviae_29840016:0.10984304768429874188)42:0.03012350418388727580,Chlamydia-Chlamydophila_felis_89898564:0.06218348185414238755);

Plantae-Arabidopsis_thaliana_22328517
((Chlamydia-Chlamydia_muridarum_15834932:0.09682261641741785052,(((Chlamydia-Candidatus_Protochlamydia_46446740:0.51713836569528637277,Plantae-Cyanidioschyzon_merolae_CMS197C:0.72973414219393917257)56:0.09008500147685889747,(((Plantae-Physcomitrella_patens_177616:0.26792690189846440907,(Plantae-Arabidopsis_thaliana_22328517:0.14829948140729945494,Plantae-Oryza_sativa_115479679:0.20725509117328044795)100:0.14751272700191964127)100:0.09966241245877700827,((Plantae-Ostreococcus_tauri_35733:0.07151037089411566017,Plantae-Ostreococcus_lucimarinus_19757:0.10888231975996966183)100:0.30373965175526324023,(Plantae-Volvox_carteri_117715_jgi:0.11442421127801828684,Plantae-Chlamydomonas_reinhardtii_132067:0.17421018741813315556)100:0.36280377191555107652)51:0.05544068031091507059)100:0.34208029718341287362,(((Bacteria-Treponema_denticola_42525620:0.79075559141384277329,Bacteria-delta_proteobacterium_94271193:0.58208873503544500405)21:0.08711038522177860788,((((Bacteria-Psychromonas_ingrahamii_119944654:0.44010087200413061659,Cyanobacteria-Synechococcus_sp._CC9605_78212359:0.39493104283132812737)100:0.18831012407164912648,(Bacteria-Burkholderia_phymatum_118031470:0.47076275237986925370,Bacteria-Syntrophobacter_fumaroxidans_116750378:0.32134283375282962636)100:0.22920144220684818270)98:0.08587324804748409923,(((Bacteria-Aeromonas_hydrophila_117621011:0.62666106104903673835,((Bacteria-Acidobacteria_bacterium_94970322:0.30973167697787229447,(Bacteria-Solibacter_usitatus_116622006:0.24736611024673477166,Cyanobacteria-Synechococcus_sp._WH_5701_87303008:0.30459292035796403653)84:0.06785674136669887779)91:0.06283475782381377217,(Bacteria-Pelobacter_propionicus_118578861:0.21832543456348452171,Bacteria-Geobacter_bemidjiensis_145618532:0.24268004339808599279)100:0.10337734404296768098)99:0.10702163647644051536)46:0.05462665113503403780,Bacteria-Photobacterium_profundum_90412499:0.59771179244148842091)45:0.07236791752772575581,(((Cyanobacteria-Lyngbya_sp._PCC_8106_119483335:0.28488858961427704353,Cyanobacteria-Trichodesmium_erythraeum_IMS101_113475562:0.28537067750264138999)99:0.09264374793949309295,(Cyanobacteria-Synechocystis_sp._PCC_6803_16329290:0.28485703773312365250,(Cyanobacteria-Crocosphaera_watsonii_WH_8501_67920688:0.07882102656861975087,Cyanobacteria-Cyanothece_sp._CCY0110_126658778:0.06048014805142547246)100:0.19956889609035521893)71:0.05473242453139320896)90:0.07315970604559743351,(((Cyanobacteria-Prochlorococcus_marinus_subsp._marinus_str._CCMP1375_33240852:0.23911632134611138234,Cyanobacteria-Prochlorococcus_marinus_str._MIT_9211_84517554:0.24559837955188137948)100:0.22157719085943561321,((Cyanobacteria-Prochlorococcus_marinus_str._MIT_9313_33863664:0.02242411223707175064,Cyanobacteria-Prochlorococcus_marinus_str._MIT_9303_124022279:0.01295606355130923294)100:0.18882239594879535183,(((Cyanobacteria-Synechococcus_sp._CC9605_78213610:0.10090439033938292113,(Cyanobacteria-Synechococcus_sp._BL107_116071209:0.02541219863554435021,Cyanobacteria-Synechococcus_sp._CC9902_78184154:0.01680197939972263160)100:0.08757362297846467414)100:0.16710135912105628098,((Cyanobacteria-Synechococcus_sp._RS9917_87123691:0.09985371665579598210,Cyanobacteria-Synechococcus_sp._RS9916_116073589:0.12583326114230414250)75:0.04947376124242333639,(Cyanobacteria-Synechococcus_sp._CC9311_113954322:0.17052572646646416143,(Cyanobacteria-Synechococcus_sp._WH_7805_88807466:0.07801173414236844961,Cyanobacteria-Synechococcus_sp._WH_7803_148240266:0.04633954748994115885)100:0.09278556996576912963)83:0.03870612817084097268)94:0.07440430485197437049)58:0.04587296392501443582,(Cyanobacteria-Synechococcus_sp._WH_5701_87303116:0.23782315301597650725,Cyanobacteria-Synechococcus_sp._RCC307_148241578:0.29109613844542364669)83:0.14168016914410228035)53:0.08108560987318469415)56:0.09896426887984292875)100:0.77269077365574767580,(Cyanobacteria-Synechococcus_elongatus_PCC_6301_56751427:0.00000121823947223981,Cyanobacteria-Synechococcus_elongatus_PCC_7942_81298897:0.00000121823947223981)100:0.20895029130233208625)56:0.19809774917360373214)37:0.06257739944540693933)99:0.17988455324187777040)93:0.12125832946612218055,(Bacteria-Methylococcus_capsulatus_53802413:0.71697945272329077770,(((Bacteria-Thermosipho_melanesiensis_150020446:0.09429645502720343520,Bacteria-Thermotoga_lettingae_157363006:0.17205375812083709142)100:0.18862683369114735532,Bacteria-Petrotoga_mobilis_145622245:0.34697036056770985679)100:0.30593197037785507808,(Bacteria-Chloroflexus_aggregans_118048461:0.37833079225432691972,(((Bacteria-

Rubrobacter_xylanophilus_108803168:0.26778107545742912965,(Bacteria-
Rhodoferax_ferrireducens_89900942:0.43353396233443064478,((Bacteria-
Magnetococcus_sp._117924812:0.39217850256177783086,Bacteria-
Rhodospirillum_rubrum_83592941:0.35717151010444148085)64:0.07601532325904374054,(Bacteria-
Magnetospirillum_magneticum_83312163:0.44077352226152188841,((((Bacteria-
Methylobacterium_chloromethanicum_156452274:0.24523329429911908117,(Bacteria-
Fulvimarina_pelagi_114704731:0.29710240353187195028,(Bacteria-
Rhodopseudomonas_palustris_90422739:0.21570817626974314596,(Bacteria-
Aurantimonas_sp._90420728:0.14883051301266037036,Bacteria-
Bradyrhizobium_sp._146341960:0.12815986319023531248)100:0.12095068284934420988)57:0.04838421714042327737)26:0.
02973556034533418602)42:0.04113840338918148265,Bacteria-
Pseudomonas_fluorescens_70730249:0.26576202235525414608)68:0.06087615190584589470,Bacteria-
Granulibacter_bethesdensis_114328836:0.26169537199630871216)96:0.11689792797936326973,(Bacteria-
Rhodobacter_sphaeroides_77463006:0.45692267769935274169,(Bacteria-
Saccharophagus_degradans_90020635:0.54522708298785260705,Bacteria-
Enterobacter_sakazakii_156936659:0.44737045393168961560)58:0.09820357653473278658)54:0.06069262095238171090)99:
0.09838184010552264436)17:0.02344832321355207194)70:0.05875997921190359574)57:0.05713569578555008355)31:0.035
22298141542960087,((((Cyanobacteria-
Nodularia_spumigena_CCY9414_119509674:0.17934818317200043603,((Cyanobacteria-
Trichodesmium_erythraeum_IMS101_113476935:0.16541842510471263528,Cyanobacteria-
Lyngbya_sp._PCC_8106_119493471:0.17659577427411252137)65:0.03749206097885779410,(Cyanobacteria-
Nostoc_punctiforme_PCC_73102_23126243:0.09155731208554300504,Cyanobacteria-
Nodularia_spumigena_CCY9414_119513193:0.11746297928559026880)100:0.12698063591515781900)51:0.03224578795812
566012)100:0.22430669021945595643,(Bacteria-Pedobacter_sp._149278115:0.38726751810607956683,(Bacteria-
Nitrosospira_multiformis_82702530:0.31552759226958271288,Bacteria-
Nitrosococcus_oceani_77165217:0.23751772103728877039)53:0.04151568660063206795)28:0.03092478302516859720)66:0.0
6555517941791103032,((Bacteria-Streptomyces_coelicolor_21225613:0.30144191289571242720,(Bacteria-
Actinomyces_odontolyticus_154508931:0.32366599134422890938,Bacteria-
Bifidobacterium_adolescentis_119025701:0.32957890745100798524)85:0.05199607240325909707)94:0.062081003894757715
02,(Bacteria-Thermobifida_fusca_72162290:0.33101978671519516340,(Bacteria-
Mycobacterium_smegmatis_118473311:0.19243885555626052963,(Bacteria-
Arthrobacter_sp._116671455:0.13673509205266734523,Bacteria-
Clavibacter_michiganensis_148272612:0.18737428340294862150)100:0.10529141658532185921)93:0.0722407556721101906
7)42:0.03465056924498094437)67:0.09269960237628849242)39:0.07299078700676436826,(Bacteria-
Acidothermus_cellulolyticus_117927889:0.51103004230776938321,(Bacteria-
Deinococcus_geothermalis_94984648:0.42571875794269742332,(Bacteria-
Anaeromyxobacter_sp._153003973:0.35112599748725803472,(Bacteria-
Myxococcus_xanthus_108757366:0.10413010047559373428,Bacteria-
Stigmatella_aurantiaca_115372407:0.14044553178802762772)100:0.20317713362561534707)100:0.15733769014547452358)3
4:0.04326214034547581910)18:0.03777828058171038561)48:0.08662611946626894155)32:0.05442251040899646897,Bacteri
a-
Salinibacter_ruber_83815924:0.55803127277879216450)63:0.05415846980359188129)100:0.38522958144638369538)28:0.075
19403986920401850)6:0.03976369276432961675)3:0.04091971338653641738)32:0.06418584401041463328,((Bacteria-
Beggiatoa_sp._153871798:0.33216904681013798006,(Cyanobacteria-
Synechocystis_sp._PCC_6803_16330244:0.21716035412097203783,Cyanobacteria-
Trichodesmium_erythraeum_IMS101_113478340:0.13215401547247820901)100:0.15201608516364475965)99:0.12615541308
265856846,(Bacteria-Clostridium_beijerinckii_150017328:0.28322930144103564132,Bacteria-
Ruminococcus_obeum_153812634:0.43625460623152073625)100:0.32397568726396985817)100:0.23780181023474628033)1
00:0.18579567782822906108)88:0.10673566899359779314)100:0.62075841741698822851,(((Chlamydia-
Chlamydophila_felis_89898407:0.09109908455616430589,Chlamydia-
Chlamydophila_abortus_62185023:0.10707205546629995629)71:0.03269484575927184694,Chlamydia-
Chlamydophila_caviae_29840170:0.07983957524983777143)99:0.12399690480648815905,(Chlamydia-
Chlamydophila_pneumoniae_33241733:0.00000121823947223981,((Chlamydia-
Chlamydophila_pneumoniae_15618303:0.00000121823947223981,Chlamydia-
Chlamydophila_pneumoniae_15835921:0.00000121823947223981)45:0.00000121823947223981,Chlamydia-
Chlamydophila_pneumoniae_16752650:0.00000121823947223981)92:0.00000121823947223981)100:0.3317384699194211861
2)88:0.09865224701190196399)100:0.23140292742944290616)100:0.05458428945791760772,Chlamydia-
Chlamydia_trachomatis_15604761:0.00000121823947223981,Chlamydia-
Chlamydia_trachomatis_76788755:0.00167643673321496889);

Plantae-Arabidopsis_thaliana_30684861

(((Chlamydia-Chlamydophila_pneumoniae_15618895:0.00000121823947223981,Chlamydia-
Chlamydophila_pneumoniae_33242355:0.00000121823947223981)33:0.00000121823947223981,Chlamydia-
Chlamydophila_pneumoniae_15836518:0.00000121823947223981)41:0.00000121823947223981,((Chlamydia-
Chlamydophila_caviae_29840531:0.08797980277130081073,Chlamydia-
Chlamydophila_felis_89898047:0.06734921044525934986)100:0.16214734336762226530,((((Chromalveolata-
Emiliania_huxleyi_UI-EH-HG2-abg-k-20-0-UI.s1_5:1.05013942533947446378,((Chromalveolata-
Thalassiosira_pseudonana_10198:0.59458770985072884052,Chromalveolata-
Phaeodactylum_tricornutum_42440:0.53739599934672388493)99:0.52008558906697144320,((Plantae-
Volvox_carteri_88325_jgi:0.54978483521434007297,(Plantae-Ostreococcus_tauri_36724:0.95156428099240564222,((Plantae-
Arabidopsis_thaliana_30684861:0.13778282402493893799,Plantae-
Oryza_sativa_115464835:0.24588353370923513030)92:0.11087408932981095144,Plantae-
Physcomitrella_patens_57897:0.41257598023834402001)97:0.24956336461793302006)10:0.06004125519887337781)16:0.057
93230564278831207,(((((Bacteria-Desulfitobacterium_hafniense_109645426:0.45481459854429928669,Bacteria-
Desulfuromonas_acetoxidans_95928571:0.76937300288097121292)20:0.10022050845923717588,((Bacteria-
Geobacter_bemidjiensis_145620126:0.48351281410256807813,Bacteria-
Pelobacter_propionicus_118579003:0.34603716829511910191)57:0.12496269208722662891,(Bacteria-
Desulfotomaculum_reducens_134298946:0.37176262920324332217,Bacteria-
Pelotomaculum_thermopropionicum_147677534:0.40578611761513433409)44:0.09615977638925296556)9:0.01753274956351
632413)6:0.04383498713179669742,((((Bacteria-
Thermosinus_carboxydivorans_121534492:0.71395432192844321406,Bacteria-
Halothermothrix_orenii_89211509:0.40515370368332259687)2:0.07968726846456515578,((((Bacteria-
Pediococcus_pentosaceus_116492413:0.35875463377736416248,Bacteria-
Lactobacillus_plantarum_28378064:0.41000612522868318255)50:0.13355824405973040769,(Bacteria-
Bacillus_sp._126651273:0.30065977879278327212,(Bacteria-
Listeria_monocytogenes_153202577:0.34968511170620486528,(Bacteria-
Geobacillus_thermodenitrificans_138896384:0.35937560621437875108,Bacteria-
Staphylococcus_saprophyticus_73662312:0.32585229983483354754)70:0.09329653035543561401)55:0.090709422680945381
30)25:0.04319734502445263941)26:0.09179237427529483850,(Bacteria-
Lactococcus_lactis_15674247:0.35872362541777547129,Bacteria-
Streptococcus_agalactiae_22537547:0.36802679053576248247)84:0.11148109541011821944)11:0.06691099530508218263,(B
acteria-Clostridium_difficile_145954058:0.37315116484370630667,(Bacteria-
Dorea_longicatena_153854162:0.41440707858218767123,Bacteria-
Ruminococcus_gnavus_154503156:0.23704826749800914087)100:0.38062937942200603114)39:0.04793040540934627447)10
:0.05221834417146267726)7:0.07550787539244536994,Bacteria-
Carboxydothermus_hydrogenoformans_78043668:0.46965701206575499738)0:0.08517141981787766658,(Bacteria-
Neisseria_meningitidis_15793917:0.41865365671878235210,Bacteria-
Salinibacter_ruber_83814943:0.44699092916752225513)25:0.07395873668496914799)0:0.03946559292130174396)4:0.061272
65410008530394,(Bacteria-Desulfovibrio_desulfuricans_78355142:0.54784843878020039742,Bacteria-
Lawsonia_intracellularis_94986639:0.63430017007573824550)24:0.12220359540962519307)10:0.03342948619266872951,(Ba
cteria-Halorhodospira_halophila_121998449:0.68154094414078547626,Bacteria-
Alkalilimnicola_ehrlichei_114320671:0.40182890108037089272)73:0.27607060875189987081)25:0.05850704749396330034)5
:0.09987554889986277606)3:0.01720635547464643977)10:0.07982332791532907590,Plantae-
Cyanidioschyzon_merolae_CMS370C:0.71009015268464348303)74:0.15017693855678998505,Chlamydia-
Candidatus_Protochlamydia_46447254:0.55074590648402055315)100:0.37095491376136385808,((Chlamydia-
Chlamydia_trachomatis_15605565:0.00000121823947223981,Chlamydia-
Chlamydia_trachomatis_76789575:0.00000121823947223981)100:0.13632454961403084326,Chlamydia-
Chlamydia_muridarum_15834837:0.04298300105777667218)95:0.10854787153905078034)71:0.16745557084900150602)100:
0.31385074673566087933,Chlamydia-Chlamydophila_pneumoniae_16752040:0.00000121823947223981);

Plantae-Arabidopsis_thaliana_30687613

(Plantae-Arabidopsis_thaliana_30687613:0.00000121823947223981,((((((Excavata-Naegleria_gruberi_51690-
fgeneshHS_pg.scaffold_47000084:1.30539753047465478808,(((Chlamydia-
Chlamydophila_pneumoniae_15836443:0.00000121823947223981,((Chlamydia-
Chlamydophila_pneumoniae_15618819:0.00000121823947223981,Chlamydia-
Chlamydophila_pneumoniae_16752126:0.00000121823947223981)17:0.00000121823947223981,Chlamydia-
Chlamydophila_pneumoniae_33242273:0.00000121823947223981)23:0.00000121823947223981)100:0.1668547589538428577
2,((Chlamydia-Chlamydophila_caviae_29840615:0.06505688146614632683,(Chlamydia-
Chlamydophila_abortus_62185427:0.08649672199412412887,Chlamydia-
Chlamydophila_felis_89897965:0.09667730167734163438)63:0.01096222303828774981)98:0.12307292230342928296,(Chlam
ydia-Chlamydia_muridarum_15834767:0.04911290580163828190,(Chlamydia-
Chlamydia_trachomatis_76789508:0.00332571878601896917,Chlamydia-
Chlamydia_trachomatis_15605499:0.00655776008668350341)100:0.05711449526290978546)100:0.25240598072208847436)3

7:0.0655519753076841l274)100:0.50273245211473938099,Chlamydia-
Candidatus_Protochlamydia_46446877:0.35737121291623452635)65:0.19698835925479360465)72:0.22075285777713166135,
((Bacteria-Thermus_thermophilus_46198512:0.68914191227555643415,Bacteria-
Deinococcus_geothermalis_94984967:0.65788194232322017019)100:0.32118399806311881406,((Bacteria-
Halorhodospira_halophila_121997464:0.50978080356625932179,(((Bacteria-
Methylococcus_capsulatus_53804021:0.34663370593696479771,Bacteria-
Thiomicrospira_crunogena_78485429:0.69110282250899079948)39:0.11418171894247730602,(((((Bacteria-
Hahella_chejuensis_83648043:0.40959911328688375365,Bacteria-
Marinobacter_sp._126666950:0.39415252333323375078)41:0.12499410420961262758,(Bacteria-
marine_gamma_90416480:0.60137757150294601782,((Bacteria-
Psychromonas_ingrahamii_119946845:0.33745271114075908736,(Bacteria-
alpha_proteobacterium_114773230:0.40331734064532598349,(Bacteria-
Alteromonadales_bacterium_119468155:0.35428241225089818212,(Bacteria-
Shewanella_frigidimarina_114564473:0.29568923807575220319,Bacteria-
Pseudoalteromonas_atlantica_109900276:0.22603944778435872132)45:0.08841757715163119091)21:0.0556989975832629796
7)7:0.03648626748718213769)34:0.09164169008057541921,(Bacteria-
Pseudomonas_fluorescens_70734069:0.26054443050083481159,Bacteria-
Oceanobacter_sp._94500523:0.36935884884324754474)40:0.07722778028531206151)28:0.05502457887657324215)16:0.0493
0240887472566585)22:0.05095493599829694492,((Bacteria-
Polynucleobacter_sp._145589940:0.65823936066319410632,((Bacteria-
Neisseria_meningitidis_121634738:0.59267854480094361858,Bacteria-
Chromobacterium_violaceum_34498844:0.34254192186661147623)31:0.09511437614911935623,((Bacteria-
Ralstonia_metallidurans_94311841:0.22828212394586089307,Bacteria-
Burkholderia_pseudomallei_76809606:0.23222632124696821921)64:0.06605039502551447728,(Bacteria-
Bordetella_bronchiseptica_33603033:0.30415437747082985087,(((Bacteria-
Polaromonas_sp._91789072:0.23475884787180964497,Bacteria-
Rhodoferax_ferrireducens_89900327:0.20537639328012174955)94:0.10340805745871306520,(Bacteria-
Comamonas_testosteroni_118052499:0.25589900774746282552,Bacteria-
Verminephrobacter_eiseniae_121611143:0.16853923262258349580)89:0.06984194384848027570)71:0.0539707327246545590
1,Bacteria-
Methylibium_petroleiphilum_124267223:0.27423331857646121579)100:0.17053445226455957173)39:0.040272901850603802
51)74:0.06682110139815866656)20:0.10214954386133784370)43:0.08397817202900800804,(Bacteria-
Nitrosomonas_eutropha_114330407:0.37011900233192174259,Bacteria-
Nitrosospira_multiformis_82701227:0.34686670274273567749)100:0.20782428771286165503)37:0.12577631514517717548)1
7:0.07995130265694429250,Bacteria-
Coxiella_burnetii_95927868:0.56327332186288270321)14:0.04664562331160519715,Bacteria-
Nitrosococcus_oceani_77166049:0.47944967108203589978)10:0.04207775094055126591)80:0.09996578259476324169,(Bacte
ria-Xylella_fastidiosa_28198004:0.34072749462624746242,(Bacteria-
Xanthomonas_oryzae_58582570:0.20118151129253558640,Bacteria-
Stenotrophomonas_maltophilia_119877720:0.20807944460265009878)89:0.15191476661287728334)100:0.1914541695373703
5004)66:0.04886502636165327568)100:0.21880863154226651290,((((Bacteria-
Chloroflexus_aggregans_118047175:0.45122554539576770205,Bacteria-
Roseiflexus_sp._148657440:0.21028502934116505285)100:0.43799604114302065350,(Bacteria-
delta_proteobacterium_94264315:0.78637015196100468195,(Bacteria-
Desulfitobacterium_hafniense_89894333:0.77592808598915941065,(((Bacteria-
Bacillus_amyloliquefaciens_154686146:0.36125687134675132928,Bacteria-
Geobacillus_thermodenitrificans_138894831:0.23826059231303217678)98:0.21699478770738087063,(Bacteria-
Clostridium_novyi_118444282:0.58242652835967267944,(Bacteria-
Carboxydothermus_hydrogenoformans_78043010:0.33802186987852134159,Bacteria-
Thermoanaerobacter_ethanolicus_114843646:0.45058458318250027563)70:0.10411217293023290742)68:0.098787628493786
52788)78:0.10958148460573507998,Bacteria-
Pelotomaculum_thermopropionicum_147677679:0.57181443855631974849)71:0.07688055595318266100)77:0.1248392861512
8224437)19:0.04628067244733394953)37:0.09664088185403409137,((Bacteria-
Pelobacter_propionicus_118581368:0.38932914865853429642,Bacteria-
Geobacter_bemidjiensis_145621179:0.35452531929545849643)100:0.22631779856114672689,(Bacteria-
Candidatus_Desulfococcus_121541057:0.56865184937905122187,Bacteria-
Syntrophobacter_fumaroxidans_116749353:0.49342652167447409450)73:0.08477563871652184224)46:0.11605059128953101
877)33:0.10247398034568140635,(Bacteria-Acidobacteria_bacterium_94971585:0.86708479042129060765,(Bacteria-
Myxococcus_xanthus_108761579:0.45936095521179232115,Bacteria-
Anaeromyxobacter_dehalogenans_86157937:0.43523951096058344223)89:0.17691807085939964539)29:0.0785148569803003
9040)59:0.08903878027485981339)56:0.09558625085873269367)99:0.27438244468977118773)84:0.20926797950170941687,
(Plantae-Cyanidioschyzon_merolae_CMS475C:0.54836248447823865426,Chromalveolata-

Aureococcus_anophagefferens_61466:0.89302376160154361973)72:0.09024240087375463848)75:0.13835649337842323425,(
Plantae-Volvox_carteri_90025_jgi:0.78709821006630076301,(Plantae-
Ostreococcus_tauri_33635:0.46949189148772391134,Plantae-
Ostreococcus_lucimarinus_3723:0.18177615428149709875)100:0.56559970760469768791)59:0.11192256116321368242)56:0.
06705862916778110305,(Plantae-Physcomitrella_patens_165772:0.29080727697141967036,((Plantae-
Physcomitrella_patens_77322:0.14745099002119144149,(Plantae-
Physcomitrella_patens_67435:0.17097712682166330000,(Plantae-
Physcomitrella_patens_172286:0.16057595793870002576,Plantae-
Physcomitrella_patens_95404:0.14340573164872560952)91:0.06084445779635179463)46:0.02928896368877680068)95:0.160
88261689822536127,(Plantae-Physcomitrella_patens_231686:0.04217568634454531284,Plantae-
Physcomitrella_patens_85626:0.22284311315308355517)100:0.53557303573803227703)86:0.12105063874742216790)91:0.19
372094227224501140)100:0.21633800964203350747,Plantae-
Oryza_sativa_115470104:0.26467229837404399051)100:0.26320330991227453010,Plantae-
Arabidopsis_thaliana_22326902:0.00000121823947223981);

Plantae-Arabidopsis_thaliana_30687794
((((Cyanobacteria-Synechococcus_sp._JA-2-3Ba2-13_86607697:0.06672605904341068206,Cyanobacteria-
Synechococcus_sp._JA-3-3Ab_86605480:0.06707299933037530471)100:0.28398737029398346543,((Cyanobacteria-
Synechococcus_elongatus_PCC_7942_81299819:0.00000121823947223981,Cyanobacteria-
Synechococcus_elongatus_PCC_6301_56750544:0.00000121823947223981)100:0.35603793521343712891,(Cyanobacteria-
Prochlorococcus_marinus_str._MIT_9211_84517829:0.84927867258801270633,((Cyanobacteria-
Gloeobacter_violaceus_PCC_7421_37523966:0.42748615628831720947,Cyanobacteria-Thermosynechococcus_elongatus_BP-
1_22298198:0.33784628433101321354)38:0.08935776527260155810,((Cyanobacteria-
Nostoc_punctiforme_PCC_73102_23125432:0.09801074014677642332,(Cyanobacteria-
Nodularia_spumigena_CCY9414_119513289:0.07345674749333316877,(Cyanobacteria-
Nostoc_sp._PCC_7120_17231365:0.01607507784996152836,Cyanobacteria-
Anabaena_variabilis_ATCC_29413_75908041:0.02530957014534982938)98:0.10688733979681691588)69:0.05302336429727
112083)97:0.22090194766162327134,((Cyanobacteria-
Synechocystis_sp._PCC_6803_16331284:0.32073049371448475453,(Cyanobacteria-
Cyanothece_sp._CCY0110_126659748:0.13395399469950314275,Cyanobacteria-
Crocosphaera_watsonii_WH_8501_67920373:0.04614848721779248736)100:0.15156947997130923445)85:0.11527831154724
631213,Cyanobacteria-
Trichodesmium_erythraeum_IMS101_113475036:0.32197103364316986829)40:0.05854284551947368936)41:0.089952601275
44908724)17:0.01945813359474423201)16:0.05278420805315253012)34:0.06228708867909880931)98:0.18687697933118813
109,((((((Bacteria-Hydrogenobaculum_sp._156718477:0.60617477635622118992,Bacteria-
Pedobacter_sp._149280291:0.71162649812352440915)80:0.26236293510688057928,(Bacteria-
Carboxydothermus_hydrogenoformans_78045168:0.37464870190504906411,Bacteria-
Thermosinus_carboxydivorans_121535833:0.32082485919929332185)18:0.06507324313575518770)3:0.04238700531105062 1
19,((Bacteria-Pelotomaculum_thermopropionicum_147677969:0.37614061719822006191,(Bacteria-
Symbiobacterium_thermophilum_51892921:0.27144290336518833717,Bacteria-
delta_proteobacterium_94263642:0.49940877504415465138)54:0.10410801377420154934)19:0.08767355766113610338,(((Ba
cteria-Caldicellulosiruptor_saccharolyticus_146296765:0.37686825284932773394,((((Plantae-
Volvox_carteri_93239_jgi:0.50966725633003651108,((((Chlamydia-
Chlamydia_trachomatis_76789463:0.00000121823947223981,Chlamydia-
Chlamydia_trachomatis_15605456:0.00000121823947223981)100:0.05917166432742073517,((Chlamydia-
Chlamydophila_abortus_62185473:0.01957853503592776531,Chlamydia-
Chlamydophila_felis_89897918:0.00582109705324843595)99:0.05921012212154695004,(Chlamydia-
Chlamydophila_pneumoniae_15618773:0.00398557893782571036,((Chlamydia-
Chlamydophila_pneumoniae_15836397:0.00000121823947223981,Chlamydia-
Chlamydophila_pneumoniae_33242224:0.00000121823947223981)13:0.00000121823947223981,Chlamydia-
Chlamydophila_pneumoniae_16752175:0.00000121823947223981)79:0.00000121823947223981)100:0.1146053106278395461
4)77:0.09688107697678438601)100:0.51952501016903951392,Chlamydia-
Candidatus_Protochlamydia_46445794:0.44992856639214362513)59:0.13159770900772016500,(((Plantae-
Oryza_sativa_115450787:0.41395680206253282485,(Plantae-
Arabidopsis_thaliana_15229266:0.05455977798698625342,Plantae-
Arabidopsis_thaliana_30687794:0.02573280824674500228)96:0.12321846019189032495)99:0.24240035139120669205,Plantae
-Physcomitrella_patens_164744:0.29687943130465760389)98:0.13282854600156943081,(Plantae-
Ostreococcus_lucimarinus_10314:0.09552341660247407051,Plantae-
Ostreococcus_tauri_6904:0.04297731989140891884)100:0.51447115223272710249)84:0.11045788097740420752)43:0.086948
47642380082453)95:0.27087909254417569249,Bacteria-
Treponema_denticola_42526387:0.76874601225503880197)70:0.14375543212747901589,(Cyanobacteria-
Prochlorococcus_marinus_subsp._pastoris_str._CCMP1986_33861215:0.08480802621665828323,(Cyanobacteria-

Prochlorococcus_marinus_str._MIT_9301_126696606:0.03833746032974448548,Cyanobacteria-
Prochlorococcus_marinus_str._AS9601_123968794:0.02031923998846420568)45:0.03887754212644547319)100:0.83596771867038144332)42:0.16666699613897778542,Bacteria-
Clostridium_botulinum_148379772:0.40477292902087291804)10:0.08439143064698211016)26:0.16883588635864507643,((Bacteria-Lactobacillus_salivarius_90961915:0.26630514425408530199,(Bacteria-
Bacillus_sp._89099642:0.41622191153753285153,Bacteria-
Streptococcus_thermophilus_116628203:0.45090767846940288388)100:0.56256477424421724720)36:0.0512146903340882017 6,Bacteria-
Leuconostoc_mesenteroides_116618395:0.46959599149747516256)38:0.07643183417892188136)6:0.08059532357317224227,((Bacteria-Leifsonia_xyli_50954353:0.10167807478304610735,(Bacteria-
Clavibacter_michiganensis_148273158:0.12365198717670278228,Bacteria-
marine_actinobacterium_88855489:0.21685001069649717520)61:0.04290007822585183844)100:0.37344918514583047564,(Bacteria-Propionibacterium_acnes_50842867:0.36803304022592520095,(((((Bacteria-
Arthrobacter_aurescens_119962911:0.30164171726462235545,(Bacteria-
Salinispora_tropica_145594471:0.39060095387749427243,(Bacteria-
Rhodococcus_sp._111017942:0.20278937128635080023,(Bacteria-
Corynebacterium_jeikeium_68535954:0.36656161863259251188,Bacteria-
Mycobacterium_vanbaalenii_120404251:0.16412961617097440725)69:0.07045171887641403508)92:0.09139754070992470170)47:0.06392223061431305531)38:0.05089492409773543891,Bacteria-
Frankia_alni_111221683:0.37169073982962302916)37:0.03936070371068154522,Bacteria-
Thermobifida_fusca_72161610:0.30749799859879123343)31:0.04684689096890026078,Bacteria-
Actinomyces_odontolyticus_154508819:0.36653123518237906708)67:0.11452579815463194424,Bacteria-
Nocardioides_sp._119716723:0.25456796487754096425)31:0.05721165725482278697)58:0.13985722027964392344)99:0.26516169832672320528)1:0.07845148650820300829)0:0.04291473371307147344)2:0.03993104180234685607,(Bacteria-
Desulfuromonas_acetoxidans_95931320:0.53781506200024953301,(Bacteria-
Bacteroides_capillosus_154497957:0.65470207845257177848,(Bacteria-
Pelobacter_propionicus_118580486:0.26859146197938238432,Bacteria-
Geobacter_metallireducens_78222432:0.24886929869310248087)78:0.05718470358171234141)64:0.14336696051341918379)12:0.05173105263630679157)1:0.06390118593232387012,(Bacteria-
Desulfitobacterium_hafniense_89895026:0.44315540777495737723,((((Bacteria-
Sphingomonas_wittichii_148555617:0.17979092908700630704,Bacteria-
Erythrobacter_litoralis_85374350:0.26454098220646615758)100:0.25622515716146199161,((Bacteria-
Methylobacterium_sp._149125635:0.27527281714020263026,(Bacteria-
Mesorhizobium_sp._110632789:0.24726152570785586704,(Bacteria-
Nitrobacter_hamburgensis_92118637:0.07389969655053447994,Bacteria-
Bradyrhizobium_sp._148253231:0.07833582981535655576)100:0.13998391310271346422)40:0.06970080612180128143)74:0.10155631322757467661,Bacteria-
Rhodospirillum_rubrum_83591991:0.28623360970837619099)51:0.12165363387178845800)39:0.06983882968227904386,((Chromalveolata-Tetrahymena_thermophila_118369895:0.67981011585594663948,Excavata-Naegleria_gruberi_61621-fgeneshNG_pg.scaffold_1000555:0.63989030962986293005)88:0.20296881622050833016,Chromalveolata-
Phytophthora_sojae_143343:0.65832629306083267906)31:0.11507353373445845801)100:0.34432821231790439187,((Bacteria-Azoarcus_sp._119898401:0.35714166148182741045,Bacteria-
Chromobacterium_violaceum_34498841:0.32727883816017450114)77:0.10153125082307991323,((Bacteria-
Thiomicrospira_crunogena_78485572:0.35795578747533662867,(Bacteria-
alpha_proteobacterium_114772921:0.15324797086400018808,Bacteria-
Pseudoalteromonas_atlantica_109899133:0.09782664387984042753)100:0.16593935017346539418)60:0.07607626723736019003,(Bacteria-Pseudomonas_aeruginosa_152987584:0.28174853929893767246,(Bacteria-
Saccharophagus_degradans_90021367:0.32359281159302799580,Bacteria-
Chromohalobacter_salexigens_92112979:0.28870656869627120411)28:0.04462711595205517967)50:0.07065268713537409262)69:0.09331507664035902649)100:0.38320047671944434953)77:0.18653342618348719983)17:0.02988835328378612946)8:0.10756352454448069034,Bacteria-
Aster_yellows_85057763:0.83904456855834275153)60:0.09806549191649578667)99:0.33010483463015227734,Bacteria-
Lawsonia_intracellularis_94987310:0.46376205059111441642,Bacteria-
Desulfovibrio_desulfuricans_78357001:0.36900693739070578037);

Plantae-Arabidopsis_thaliana_30688378
((Bacteria-Pseudomonas_mendocina_146305392:0.21363176200131248073,(((((((Bacteria-
Photobacterium_sp._89075910:0.09855076091712013098,(Bacteria-
Vibrionales_bacterium_148979387:0.03948190879146549342,Bacteria-
Vibrio_vulnificus_27364078:0.02446905051846398615)99:0.06681475742224354808)100:0.12784331662059314483,(Bacteria-Actinobacillus_pleuropneumoniae_126208199:0.21594354676952853822,Bacteria-
Haemophilus_ducreyi_33152441:0.21787548070571138847)100:0.17233133816968843055)91:0.06193628175937360147,(Bact

eria-Aeromonas_salmonicida_145297591:0.1771161299725168558 6,Bacteria-
Moritella_sp._149907766:0.18741213790542679551)44:0.02467545631569239423)92:0.06492779797763173488,((Bacteria-
Shewanella_frigidimarina_114561752:0.16640976337682744646,(Bacteria-
Pseudoalteromonas_tunicata_88858520:0.09867783567234802988,Bacteria-
Alteromonadales_bacterium_119468107:0.08559674810687797897)100:0.09940576581001608902)72:0.04827505466318242344,Bacteria-
Psychromonas_ingrahamii_119943967:0.13472679309553686755)92:0.05796401915515805164)96:0.12600064398176163971,(
Bacteria-Legionella_pneumophila_148360770:0.75042551572606153165,(((((((Chlamydia-
Chlamydia_trachomatis_76789432:0.00254675213807268371,Chlamydia-
Chlamydia_trachomatis_15605425:0.00507805266924921377)100:0.05880597452154263938,Chlamydia-
Chlamydia_muridarum_15834689:0.08750113131524621390)100:0.21947974510273929316,((Chlamydia-
Chlamydophila_felis_89898751:0.02931880903162939825,(Chlamydia-
Chlamydophila_abortus_62184708:0.03839870142701905242,Chlamydia-
Chlamydophila_caviae_29839828:0.03398044674414053140)64:0.01750666990057030188)100:0.08791585152500398792,(((Chlamydia-Chlamydophila_pneumoniae_16752361:0.00000121823947223981,Chlamydia-
Chlamydophila_pneumoniae_15618590:0.00000121823947223981)25:0.00000121823947223981,Chlamydia-
Chlamydophila_pneumoniae_33242038:0.00000121823947223981)79:0.00000121823947223981,Chlamydia-
Chlamydophila_pneumoniae_15836212:0.00501546538293586811)100:0.10960880371141847045)100:0.2075280591516671380 2)100:0.53453198220300068844,(Chlamydia-Candidatus_Protochlamydia_46445733:0.32950003876438821449,(Plantae-
Physcomitrella_patens_144831:0.13964379495938350484,(Plantae-
Oryza_sativa_115447039:0.10568591948758930543,(Plantae-
Arabidopsis_thaliana_30688383:0.00000121823947223981,Plantae-
Arabidopsis_thaliana_30688378:0.00000121823947223981)100:0.07665326284023651904)100:0.15513396445285082548)100:0.55850039162135245441)88:0.09032321003832297079)93:0.16996593398705034383,((Plantae-
Cyanidioschyzon_merolae_CMT476C:1.86053621442967198618,(((Bacteria-
Psychrobacter_sp._148652597:0.25772018146142833261,Bacteria-
Neisseria_meningitidis_121635484:0.21060923381404403343)81:0.05178778415074051372,(Bacteria-
Campylobacter_hominis_154148399:0.30163577929885682094,(Bacteria-
Mycobacterium_bovis_121638161:0.17027611185324620369,Bacteria-
Corynebacterium_jeikeium_68535688:0.30391281656925528409)66:0.06597001678075233022)56:0.07568082299841370764)100:0.56853918974614858772,(Bacteria-Sulfurovum_sp._152993850:0.25868933667280102906,(((((Bacteria-
alpha_proteobacterium_114769509:0.20732980381179980101,(((Bacteria-
Rhodobacterales_bacterium_126724755:0.09494102079438646036,(Bacteria-
Silicibacter_sp._99080449:0.09847820072792946855,(Bacteria-
Sagittula_stellata_126730938:0.12416572444955417676,Bacteria-
Paracoccus_denitrificans_119383417:0.18452058890133563041)82:0.05648419221018816799)72:0.03429565127342097675)96:0.08031816483495941850,Bacteria-
Roseobacter_sp._126739952:0.10310251612167931068)85:0.04317939774741043346,(Bacteria-
Rhodobacter_sphaeroides_126461611:0.17797146201793864750,Bacteria-
Roseovarius_sp._114763997:0.21514524502795867589)79:0.05406436508863177171)72:0.03180110089286147107)44:0.0246752902868568 0817,(Bacteria-Stappia_aggregata_118588885:0.15621778963926308270,Bacteria-
Mesorhizobium_sp._110633686:0.28044130558440183432)56:0.04804209506848602235)67:0.05275920754187488765,Bacteria-Hyphomonas_neptunium_114799704:0.19215581873262957413)81:0.08763012374305238772,(Bacteria-
Rhodospirillum_rubrum_83593369:0.31876102434401187846,Bacteria-
Aurantimonas_sp._90419406:0.37367885846704784747)97:0.16033277021297911991)71:0.16607348728979162700,(((Bacteria-Thiomicrospira_denitrificans_78776703:0.29345540096740635461,Bacteria-
Marinobacter_algicola_149377695:0.30381860018219980502)22:0.05171786573009929527,Bacteria-
Wolinella_succinogenes_34558163:0.35756799830809238294)14:0.04285850480089947734,Bacteria-
Nitratiruptor_sp._152990317:0.21921626690568690732)24:0.05886010635795650703)34:0.09015859914353697990)100:0.39363864157830102952)72:0.12241770788681191617)49:0.08091349499698823056,((((((Chromalveolata-
Aureococcus_anophagefferens_10532:0.22012624850858616976,Chromalveolata-
Aureococcus_anophagefferens_10551:0.13188761711994109738)100:0.36827347852741010303,(Plantae-
Ostreococcus_tauri_8163:0.17376322188156098636,(Plantae-
Ostreococcus_lucimarinus_36108:0.00467879340868943353,Plantae-
Ostreococcus_lucimarinus_42057:0.01478033779969834156)100:0.12504737718757574783)100:0.25508325752924643215)100:0.29259569581460853627,(((Excavata-Trypanosoma_brucei_115503937:0.01019038697345296847,Excavata-
Trypanosoma_brucei_115503941:0.00636282327435390253)100:0.23604741392641107045,((Excavata-
Trypanosoma_cruzi_71419198:0.00000121823947223981,Excavata-
Trypanosoma_cruzi_71419196:0.00000121823947223981)99:0.00376643673536886364,(Excavata-
Trypanosoma_cruzi_71654882:0.00237349975526313572,Excavata-
Trypanosoma_cruzi_71654884:0.00000121823947223981)99:0.01295874995914064395)100:0.15479168350679023414)100:0.41903250491843274661,((((Plantae-Chlamydomonas_reinhardtii_196237:0.00579163391126812782,(Plantae-

Chlamydomonas_reinhardtii_196335:0.0000012182394722398l,Plantae-
Chlamydomonas_reinhardtii_118310:0.0000012182394722398l)100:0.00847959984109306064)100:0.1092388815l418438552,
Plantae-Chlamydomonas_reinhardtii_196472:0.11036251939750843509)97:0.08119522059336510555,((Plantae-
Chlamydomonas_reinhardtii_196438:0.02707563498922218453,Plantae-
Chlamydomonas_reinhardtii_196439:0.03028211131362041481)85:0.06039777033791528249,(Plantae-
Volvox_carteri_79831_jgi:0.11507408312754370538,Plantae-
Volvox_carteri_79832_jgi:0.14770333684875258662)48:0.03678787614793641719)41:0.03881046411513481426)100:0.29814
376331331104986,((Plantae-Chlamydomonas_reinhardtii_196464:0.01056791109007469698,Plantae-
Chlamydomonas_reinhardtii_196465:0.02802743063694704273)100:0.63627984309288487808,(Plantae-
Volvox_carteri_70155_jgi:0.14211543186411215722,(Plantae-
Chlamydomonas_reinhardtii_196333:0.19922656243680098020,((Plantae-
Chlamydomonas_reinhardtii_181195:1.09865224733939470525,Plantae-
Chlamydomonas_reinhardtii_196306:0.00000121823947223981)100:0.16732878911348625373,Plantae-
Volvox_carteri_105976_jgi:0.25120192509504984946)73:0.05352519952243587481)40:0.02257202065173777653)100:0.2340
4101816924899326)36:0.04248176635107886456)83:0.13118438318227720885)94:0.13565761056683428576)84:0.137282244
95426080698,((Opisthokonta-Magnaporthe_grisea_39973343:0.73215740289568886912,(Opisthokonta-
Magnaporthe_grisea_39942536:0.45491278573361437765,((Opisthokonta-
Cryptococcus_neoformans_58261344:0.26668776991531106813,Opisthokonta-Sporobolomyces_roseus_9234-
e_gw1.1.322.1:0.23930181814111037286)100:0.15342403150863531680,Opisthokonta-
Ustilago_maydis_71018783:0.27152518446029011434)100:0.11585720816704657421)100:0.18846704267892838702)100:0.36
760675340882054130,(Chromalveolata-Thalassiosira_pseudonana_261260:0.40445076918355743478,(Chromalveolata-
Phaeodactylum_tricornutum_23830:0.34777871216856948156,Chromalveolata-
Thalassiosira_pseudonana_36201:0.30188525660132259354)99:0.16008704420924407819)100:0.23661667518953566347)50:0
.06728169309843008705)69:0.10214932486882279117,((Excavata-Naegleria_gruberi_55679-
estExt_fgeneshHS_pm.C_100012:0.68432863786440090159,Excavata-
Euglena_gracilis_109789009_2:1.05735900851638420228)59:0.19627915246877422617,(((Opisthokonta-
Danio_rerio_117606240:0.26970864140102068784,Opisthokonta-
Mus_musculus_30794520:0.07246239697156639514)100:0.07806981766494031083,(Opisthokonta-
Mus_musculus_7657579:0.11060589231202253013,((Opisthokonta-
Danio_rerio_125824283:1.12893786056507217097,Opisthokonta-
Danio_rerio_47085961:0.00000121823947223981)100:0.11444860457541548848,Opisthokonta-
Danio_rerio_47086851:0.06187847153520493332)100:0.08569496031869963670)98:0.08944352152720365379)99:0.18088134
634082969621,(Opisthokonta-Apis_mellifera_48099304:0.34134633877720155226,Opisthokonta-
Drosophila_melanogaster_21356511:0.25224836925843358015)100:0.16161574406071027576)100:0.4400488956186201506)
23:0.07268294488306221990)91:0.27375252029751390159,((((Bacteria-
Thermosipho_melanesiensis_150021785:0.35112082650405790352,Bacteria-
Fervidobacterium_nodosum_154249188:0.35235472801534184484)91:0.11766422692719895327,Bacteria-
Thermotoga_lettingae_157363424:0.33147296817986282580)100:0.30047752864925553196,Bacteria-
Desulfovibrio_desulfuricans_78358819:0.58745446135575574154)97:0.15618008927241050499,((Cyanobacteria-
Nostoc_punctiforme_PCC_73102_23126971:0.10296285896204283861,((Cyanobacteria-
Anabaena_variabilis_ATCC_29413_75906380:0.03090961553941280748,Cyanobacteria-
Nostoc_sp._PCC_7120_17229828:0.03129127425385798411)80:0.09232206315101339211,Cyanobacteria-
Nodularia_spumigena_CCY9414_119512007:0.12504530433202049045)36:0.05428701267584411239)100:0.80026601851458
267767,(Plantae-Galdieria_sulphuraria_Contig393_6:0.62482623048762164775,Plantae-
Cyanidioschyzon_merolae_CMS220C:0.55018947889662239703)100:0.32725120773780319583)37:0.11294437914101676523
)28:0.09128527818386514281)12:0.06648225595843496039)85:0.14640095422599538888)95:0.20720441389036234336,(Bact
eria-Magnetococcus_sp._117923545:0.22393267153065948838,Bacteria-
Desulfotalea_psychrophila_51246480:0.22018728501463544212)100:0.11616504924808331023)48:0.06815165486594006172,
Bacteria-
Candidatus_Ruthia_118602176:0.36345461048452304409)16:0.00876291086874031108)2:0.05044704062789685112)6:0.0318
1890752937226174,(Bacteria-Coxiella_burnetii_154706571:0.39191956091884888602,(Bacteria-
Alkalilimnicola_ehrlichei_114321973:0.16993228587596015400,Bacteria-
Halorhodospira_halophila_121997844:0.31931333946639495203)100:0.24517110894729476911)17:0.06597633057829511982
)39:0.07078568154103791199,(Bacteria-Chromohalobacter_salexigens_92112844:0.25442751862868651092,Bacteria-
Oceanospirillum_sp._89095476:0.15434186310550404575)74:0.09167334484188985333)25:0.02504507050171548774)28:0.04
816218954134945246,Bacteria-marine_gamma_119505430:0.31561033904905316794,Bacteria-
Alcanivorax_borkumensis_110835166:0.23496996228811378726);

Plantae-Arabidopsis_thaliana_30691669

((Chromalveolata-Thalassiosira_pseudonana_39062:1.00629010661898865564,Chromalveolata-
Phaeodactylum_tricornutum_49741:0.41842327761947395448)99:0.48954070611875522001,((Bacteria-
Francisella_tularensis_118497361:1.17555152972337517703,(((Bacteria-

Symbiobacterium_thermophilum_51892235:0.72960850802181842489,(Bacteria-
Salinibacter_ruber_83816316:0.89426194977730577573,(((Cyanobacteria-Synechococcus_sp._JA-2-3Ba2-
13_86610253:0.04223618328943880978,Cyanobacteria-Synechococcus_sp._JA-3-
3Ab_86605012:0.07162533065358779594)100:0.24414769362709648481,Cyanobacteria-
Gloeobacter_violaceus_PCC_7421_37523750:0.37741983839279347190)98:0.31996094582042389209,(Bacteria-
Acidobacteria_bacterium_94968032:0.55572687834136180740,(Bacteria-
Acidothermus_cellulolyticus_117927294:0.42552197700432364824,Bacteria-
Kineococcus_radiotolerans_152964880:0.44996977605165727754)96:0.24061360211682270016)33:0.10137755836007822374)
100:0.54893669051642424161)20:0.11053776145768633155)12:0.14981302032309104511,((Bacteria-
Propionibacterium_acnes_50842892:1.14484327888339731949,(((Bacteria-
Thermosinus_carboxydivorans_121534244:0.57702270705083547053,(((Bacteria-
Streptococcus_pyogenes_15674507:0.36930691381141489416,Bacteria-
Lactococcus_lactis_15672589:0.43654638381137789160)98:0.23299417362100510731,((((Bacteria-
Bacillus_anthracis_47530101:0.35359244477590090483,Bacteria-
Geobacillus_thermodenitrificans_138896271:0.32941066590981155082)80:0.13557949521894671618,(Bacteria-
Staphylococcus_saprophyticus_73662967:0.64712631082215699152,Bacteria-
Listeria_monocytogenes_153174298:0.47406936365309587655)18:0.06408995543297524300)30:0.08212365173903397875,((
Bacteria-Pediococcus_pentosaceus_116492486:0.66382818035260604539,(Bacteria-
Leuconostoc_mesenteroides_116618817:0.22701875499365606337,Bacteria-
Oenococcus_oeni_116490953:0.32396495223503091188)98:0.25948574721249251240)43:0.06842821843300329887,Bacteria-
Lactobacillus_sakei_81428990:0.44903872106397946240)37:0.10652512694222419920)36:0.15454571708468906044,(Bacteri
a-Aster_yellows_85057856:0.93260659426281200535,Bacteria-
Mycoplasma_mycoides_42561043:0.55150174913450267233)89:0.34872674281900534199)37:0.04592830668525778315)94:0
.33109881591162881875,(Bacteria-Thermoanaerobacter_ethanolicus_114844162:0.67361161979914863096,((Bacteria-
Halothermothrix_orenii_89211290:0.84178350052728700970,Bacteria-
Alkaliphilus_metalliredigens_150389425:0.52848360096795432561)25:0.18839453290047747380,Bacteria-
Clostridium_thermocellum_125973737:0.41706649386745736630)12:0.05860925317509126792)13:0.08138979131394716293)
4:0.02244234980425836509)4:0.07686276988165489932,(Bacteria-
Desulfitobacterium_hafniense_109647669:0.70935206303894360325,(Bacteria-
Pelotomaculum_thermopropionicum_147678286:0.52937054544058304018,Bacteria-
Desulfotomaculum_reducens_134299475:0.41512878205055236958)84:0.14662344677880079225)22:0.13908086940750979995
4)1:0.10557101268897527147,(Chlamydia-Candidatus_Protochlamydia_46447426:0.92156331319143691694,(((Bacteria-
Sphingomonas_sp._94495543:0.18217964218994481262,(Bacteria-
Novosphingobium_aromaticivorans_87200913:0.15662890590418379611,Bacteria-
Erythrobacter_litoralis_85373248:0.07384122506423942967)100:0.22978541718055103815)100:0.44604897880129246168,(Ba
cteria-Stappia_aggregata_118589655:0.32931118923083141370,(Bacteria-
Mesorhizobium_sp._110636002:0.12629663791298056941,(Bacteria-
Ochrobactrum_anthropi_153007507:0.17485140805562840449,Bacteria-
Sinorhizobium_medicae_150395253:0.22847506348804819121)85:0.09549287582971827115)72:0.10926316638464639297)10
0:0.25315084353899774960)93:0.18466976986122193849,Bacteria-
Chloroflexus_aggregans_118047421:0.42198382666346351266)98:0.29788893535797283940)31:0.10595086761432440103)3:
0.01095278045943741396)2:0.09732996278831225478,(((Bacteria-
Carboxydothermus_hydrogenoformans_78042655:0.72028239122530113292,Bacteria-
Solibacter_usitatus_116624472:0.80869627823101664088)17:0.12767930923015197164,Bacteria-
Bacteroides_capillosus_154500974:0.73676011678354114132)9:0.14192493273122722131,((Chromalveolata-
Phaeodactylum_tricornutum_49451:1.14967336750889836949,(((Bacteria-
Clavibacter_michiganensis_148272114:0.40240265561523097526,(Bacteria-
Ruminococcus_obeum_153810011:0.17477175999785565796,Bacteria-
Dorea_longicatena_153855701:0.25747499283874347720)100:0.46630412019465800055)100:0.32275216370466430771,(Bact
eria-Mycobacterium_vanbaalenii_120405985:0.46691806516974215802,Bacteria-
Saccharopolyspora_erythraea_134097236:0.15066509400041186439)98:0.25384226147245153582)85:0.209025040515264881
60,Bacteria-
Caulobacter_sp._113932427:0.76678943172657476612)78:0.26970454412538463762)100:0.53440015619337144503,(((Chrom
alveolata-Karenia_brevis_Kb_CAGO_R_113A2_5:1.05093196904105523615,Chromalveolata-
Karenia_brevis_Contig3917_5:1.19692268496635101371)92:0.32244442152635216514,(((Plantae-
Chlamydomonas_reinhardtii_108655:0.38703630202269662108,Plantae-
Volvox_carteri_99057_jgi:0.22738406357961757354)100:0.34779958741244931808,(Plantae-
Arabidopsis_thaliana_30691669:0.29573182301575756670,Plantae-
Oryza_sativa_115454647:0.41250981659806373880)100:0.37033113788287053136)55:0.11041002788898843234,(Plantae-
Ostreococcus_tauri_34264:0.30779389684984098663,Plantae-
Ostreococcus_lucimarinus_87970:0.18495135486275460224)100:0.57595711166689678162)91:0.15925519714087962853)60:0
.11780070882323498416,Chlamydia-

Candidatus_Protochlamydia_46445775:0.57494225216356176222)96:0.31280392100797094201)29:0.05078220366216251030)
5:0.10263535166475198923)0:0.04594825262515587344)3:0.10244389417896207495,(Bacteria-
Moorella_thermoacetica_83590588:0.70320778075898771053,(((Bacteria-
Nitrosomonas_europaea_30248779:0.37288464954391359329,Bacteria-
Nitrosospira_multiformis_82702991:0.42125200677919505399)99:0.27458646247920293071,Bacteria-
Azoarcus_sp._119898184:0.35612484356745172098)99:0.49888039469057116060,(((Cyanobacteria-
Thermosynechococcus_elongatus_BP-1_22299511:0.43633732215937720555,((Cyanobacteria-
Synechococcus_elongatus_PCC_6301_56750360:0.00000121823947223981,Cyanobacteria-
Synechococcus_elongatus_PCC_7942_81300008:0.00000121823947223981)100:0.29646373318512297956,((Cyanobacteria-
Gloeobacter_violaceus_PCC_7421_37520123:0.63664193544652336598,Cyanobacteria-Synechococcus_sp._JA-3-
3Ab_86605783:0.38304435359422545071)83:0.14612846097921897148,((Cyanobacteria-
Cyanothece_sp._CCY0110_126656411:0.09667707751094833935,Cyanobacteria-
Crocosphaera_watsonii_WH_8501_67925525:0.12891681451025902017)100:0.30657104562070797149,(Cyanobacteria-
Lyngbya_sp._PCC_8106_119493555:0.22388376313547622698,(Cyanobacteria-
Trichodesmium_erythraeum_IMS101_113476712:0.38799497190661957902,(Cyanobacteria-
Nodularia_spumigena_CCY9414_119513673:0.08918929201710205923,(Cyanobacteria-
Nostoc_punctiforme_PCC_73102_23124776:0.09387945187762782029,(Cyanobacteria-
Anabaena_variabilis_ATCC_29413_75907658:0.03443398066835195309,Cyanobacteria-
Nostoc_sp._PCC_7120_17227671:0.01414980405669547317)98:0.10260591778166291976)48:0.01085040361032947945)99:0.
20280357371779494935)65:0.04570921502689470617)59:0.06092482816396885964)81:0.10620141132024704667)27:0.04778
290932111091405)72:0.12430369712964216278)75:0.08415066254420985881,Cyanobacteria-
Synechocystis_sp._PCC_6803_16330947:0.42659666416943398115)92:0.12076188596887604820,(((Cyanobacteria-
Prochlorococcus_marinus_str._MIT_9211_84517586:0.35188017615647504410,(Cyanobacteria-
Prochlorococcus_marinus_str._MIT_9303_124023679:0.01230165780105957861,Cyanobacteria-
Prochlorococcus_marinus_str._MIT_9313_33862607:0.01399508479101467355)100:0.18158394259549093253)50:0.05699753
632774577639,((Cyanobacteria-Synechococcus_sp._BL107_116072328:0.00999830485272037821,Cyanobacteria-
Synechococcus_sp._CC9902_78185098:0.02544555266516766376)100:0.17885650380226636447,Cyanobacteria-
Synechococcus_sp._WH_8102_33866165:0.15848641243488834074)99:0.13204480732879306237)69:0.11774714333949184
986,(Cyanobacteria-Prochlorococcus_marinus_str._MIT_9515_123966693:0.11542609295354341881,Cyanobacteria-
Prochlorococcus_marinus_str._MIT_9312_78779779:0.12611672877298429896)100:0.57777596473583170411)100:0.4522181
0706935205992)99:0.15838701958105746992)15:0.08904453592310360155)6:0.05312112441128374191)41:0.1863225905755
2416626)74:0.18643928786078389770,Bacteria-
Roseiflexus_sp._148655189:0.39138070609766395513)41:0.11761923555797794416,Bacteria-
Herpetosiphon_aurantiacus_113938219:0.51808026243504312625);

Plantae-Arabidopsis_thaliana_30697049
(Bacteria-Rhodococcus_sp._111023207:0.26954128731445242684,(((Bacteria-
Actinomyces_odontolyticus_154507745:0.25544888619693628673,Bacteria-
Corynebacterium_glutamicum_19553579:0.36378628746957447637)70:0.11334927681437731439,(Bacteria-
Streptomyces_avermitilis_29829979:0.08698818084586261434,(Bacteria-
Frankia_alni_111224976:0.15462580990860316787,(Bacteria-
Thermobifida_fusca_72160496:0.08260304668793524985,Bacteria-
Saccharopolyspora_erythraea_134100212:0.10046207544738200435)31:0.02230911303592901865)29:0.041402964206110136
34)45:0.04743105732380006878)41:0.05097735526708448844,(((Bacteria-
Arthrobacter_aurescens_119962447:0.15438994842265998941,(Bacteria-
Mycobacterium_ulcerans_118619609:0.12634069366214575880,Bacteria-
Nocardioides_sp._119717800:0.05328539218544626338)63:0.02418785085502070387)89:0.06791509036615815675,Bacteria-
Propionibacterium_acnes_50843200:0.18383086648748611647)99:0.08202765289635809143,((((Bacteria-
Coxiella_burnetii_153208837:0.21420834196092017332,(Bacteria-
Legionella_pneumophila_148359893:0.26789489915545294796,Bacteria-
Rickettsiella_grylli_94493367:0.30038661263181137828)68:0.07044873315215723075)62:0.07322134730577398165,Bacteria-
Leptospira_borgpetersenii_116328112:0.29206224652800671926)81:0.09310128937015266226,((((Plantae-
Ostreococcus_tauri_34202:0.08371569765623629311,Plantae-
Ostreococcus_lucimarinus_42336:0.02380223280568109431)100:0.17885704174424976731,((Plantae-
Volvox_carteri_76573_jgi:0.02389741212884383126,(Plantae-
Chlamydomonas_reinhardtii_192083:0.00000121823947223981,Plantae-
Chlamydomonas_reinhardtii_80866:0.00000121823947223981)100:0.02392483454571408749)100:0.23415969412304282748,(
(Plantae-Oryza_sativa_115477843:0.07238806404546845041,(Plantae-
Arabidopsis_thaliana_42573724:0.00000121823947223981,(Plantae-
Arabidopsis_thaliana_30697051:0.00000121823947223981,Plantae-
Arabidopsis_thaliana_30697049:0.00000121823947223981)100:0.00000121823947223981)100:0.05368049613764808159)93:0
.06174140163066667991,(Plantae-Physcomitrella_patens_93518:0.05245094329542387274,(Plantae-

Physcomitrella_patens_77520:0.01852637974181954930,Plantae-
Physcomitrella_patens_74635:0.01727973175478444631)63:0.01792837180631134258)93:0.09163382924177340683)91:0.083
71034233787695567)45:0.03799231938093408645)95:0.34023293099146234564,(Chlamydia-
Candidatus_Protochlamydia_46447406:0.27166977387573304492,((Chlamydia-
Chlamydia_muridarum_15835270:0.03750331502686102270,(Chlamydia-
Chlamydia_trachomatis_15605100:0.00000121823947223981,Chlamydia-
Chlamydia_trachomatis_76789106:0.00306061955492072926)100:0.02986305494617023590)84:0.06432692111687671188,(((
Chlamydia-Chlamydophila_pneumoniae_15836559:0.00000121823947223981,Chlamydia-
Chlamydophila_pneumoniae_33242398:0.00000121823947223981)12:0.00000121823947223981,(Chlamydia-
Chlamydophila_pneumoniae_15618936:0.00000121823947223981,Chlamydia-
Chlamydophila_pneumoniae_16751997:0.00000121823947223981)13:0.00000121823947223981)100:0.0978909711479981209
4,(Chlamydia-Chlamydophila_abortus_62185314:0.00625008682481718349,(Chlamydia-
Chlamydophila_felis_89898089:0.03545572177015206272,Chlamydia-
Chlamydophila_caviae_29840491:0.04064646350735189040)82:0.02614301933731567537)96:0.09303229338134870463)79:0.
04708092152764903338)100:0.50726454940785059566)79:0.09214751199325797726)75:0.13749756593737028076,Bacteria-
Magnetococcus_sp._117924121:0.41179452038678188153)18:0.05834596972230360334)44:0.18981163501835893181,(Amoe
bozoa-Hartmannella_vermiformis_Contig900_1:0.24748289225722838736,(((((Excavata-
Trichomonas_vaginalis_123431031:0.19379546675936573652,Excavata-
Trichomonas_vaginalis_123448526:0.16045600908079180047)95:0.15454057933729312135,((Excavata-
Trichomonas_vaginalis_123398810:0.12346305374301029534,(Excavata-
Trichomonas_vaginalis_123428711:0.09906014569959374627,((Excavata-
Trichomonas_vaginalis_123438067:0.01716010899157103392,(Excavata-
Trichomonas_vaginalis_123404233:0.02079985956716734127,Excavata-
Trichomonas_vaginalis_123407394:0.01747251675807407617)55:0.00998749880268716590)78:0.02283936416833731373,((E
xcavata-Trichomonas_vaginalis_123431390:0.00619368243221045216,Excavata-
Trichomonas_vaginalis_123431388:0.00000121823947223981)100:0.05406331170128867575,Excavata-
Trichomonas_vaginalis_123369283:0.02391036228386695522)53:0.01220305032106345708)74:0.05368348803823506932)50:
0.04781040006588388458)100:0.41343095757940917512,((Cyanobacteria-
Nodularia_spumigena_CCY9414_119509419:0.00000121823947223981,Cyanobacteria-
Nostoc_punctiforme_PCC_73102_23126205:0.17838469602391748836)100:1.39400681882674581935,Cyanobacteria-
Nostoc_punctiforme_PCC_73102_23125612:1.58208945300840886361)97:1.09533192124691636238)35:0.1925414831970948
2595)33:0.12120205078698076395,(Excavata-Giardia_lamblia_157435221:0.00000121823947223981,Excavata-
Giardia_lamblia_159113785:0.00000121823947223981)100:0.70411913678266402350)21:0.11689789213782735000,(((Excava
ta-Malawimonas_jakobiformis_Contig704_3:0.36284037428165699657,((Chromalveolata-
Tetrahymena_thermophila_118383251:0.35166757921741076398,(Chromalveolata-
Paramecium_tetraurelia_124401717:0.00000121823947223981,(Chromalveolata-
Paramecium_tetraurelia_124424507:0.01755403557491340763,Chromalveolata-
Paramecium_tetraurelia_124396669:0.15223064171649175469)99:0.03652499558932743168)99:0.29237176318723784307)97:
0.23119241777750057509,((Amoebozoa-Entamoeba_histolytica_67482981:0.63743002339351795271,(((Amoebozoa-
Entamoeba_histolytica_67469755:0.00000121823947223981,Amoebozoa-
Entamoeba_histolytica_67464753:0.00000121823947223981)32:0.00000121823947223981,Amoebozoa-
Entamoeba_histolytica_67469777:0.00000121823947223981)100:0.02618335940898701419,Amoebozoa-
Entamoeba_histolytica_67480659:0.00640222680434992392)100:0.35768835978575275192)89:0.28809761491861851646,Exc
avata-
Euglena_gracilis_Contig1722_3:0.56061634404105142870)8:0.08228770431176357647)3:0.04516457636698956168)2:0.0510
4614835839981629,((((Plantae-Physcomitrella_patens_195276:0.09621671102359186101,((Plantae-
Oryza_sativa_115482534:0.05457330648623415909,(Plantae-
Arabidopsis_thaliana_15219721:0.03850920669607321017,Plantae-
Arabidopsis_thaliana_15239843:0.01818368590165811427)88:0.02803088077906777317)57:0.03242205062692736506,(Planta
e-Oryza_sativa_115459790:0.23844968723958515078,Plantae-
Arabidopsis_thaliana_15241923:0.21869015212185366481)55:0.06151346972203061703)44:0.02718273609648394831)92:0.0
7315074261231063746,((Plantae-Ostreococcus_lucimarinus_87414:0.09071924378962076263,Plantae-
Ostreococcus_tauri_4610:0.16354171582919674321)100:0.32351492031847206565,(Plantae-
Volvox_carteri_109769_jgi:0.05338958280728389044,(Plantae-
Chlamydomonas_reinhardtii_118222:0.00000121823947223981,Plantae-
Chlamydomonas_reinhardtii_158129:0.00000121823947223981)100:0.08230808728982398625)100:0.15293705469109447614
)74:0.08103652322363837091)73:0.17880517520670721754,(Excavata-
Jakoba_libera_Contig382_3:0.34366381462701572902,((((Chromalveolata-
Paramecium_tetraurelia_124398937:0.50921475974365415507,(((Chromalveolata-
Paramecium_tetraurelia_124414336:0.01084029211729969737,Chromalveolata-
Paramecium_tetraurelia_124409410:0.02559586469974904632)100:0.57500629595618080181,Chromalveolata-
Tetrahymena_thermophila_118363871:0.36956781303371644132)96:0.18240615135015963988,Chromalveolata-

Tetrahymena_thermophila_118362698:0.25523245898129770515)55:0.03405057671148571663)95:0.22430881413763420484,(
Chromalveolata-Thalassiosira_pseudonana_41425:0.72753578535516016945,Chromalveolata-
Phytophthora_sojae_109232:0.38406531035187246870)19:0.10171491769813516270)4:0.04229649888394183127,((Chromalv
eolata-Isochrysis_galbana_Contig990_2:0.31556843115165134073,(Plantae-
Porphyra_yezoensis_Contig147_1:0.43055914084834040478,(Plantae-
Galdieria_sulphuraria_HET_42B11_3:0.28944195385918730645,Plantae-
Cyanidioschyzon_merolae_CMT611C:0.44070208277646027994)60:0.08172849776951193745)53:0.08481015993475798931)
38:0.08515408969275950479,((Excavata-Trypanosoma_brucei_74025248:0.09284419636753474259,(Excavata-
Trypanosoma_cruzi_71405868:0.00000121823947223981,Excavata-
Trypanosoma_cruzi_71411668:0.02488075723718237431)100:0.32028156789206857491)100:0.40456226327346750393,(Chro
malveolata-Pavlova_lutheri_Contig469_1:0.00000121823947223981,Chromalveolata-
Pavlova_lutheri_PLE00001528_1:0.05644149911742422920)99:0.49167722072689312718)13:0.07228704506481985204)11:0.
05411682544322539423)9:0.04330726549580108525,(Opisthokonta-
Danio_rerio_115529401:0.70565387056882356021,Opisthokonta-
Mus_musculus_40254153:0.73115467814221557497)100:1.14303509301995509517)7:0.02847432099701051322)5:0.0286670
3084225760353)2:0.02972427036447304605,((((Opisthokonta-
Apis_mellifera_66506786:0.20430035332103507750,Opisthokonta-
Drosophila_melanogaster_24583394:0.15668618743736287269)95:0.14715975530831956752,(Opisthokonta-
Mus_musculus_31982178:0.12575298388038988473,(Opisthokonta-
Danio_rerio_41053939:0.14814398944337323072,Opisthokonta-
Danio_rerio_41053921:0.07689564473581039783)88:0.05696963655041471786)99:0.11330321651543807937)85:0.110420262
41539694420,(Amoebozoa-Acanthamoeba_castellanii_Contig362_3:0.25464769542577891936,((Excavata-
Malawimonas_californiana_Contig208_1:0.30031977360067968474,(Excavata-
Malawimonas_jakobiformis_Contig1102_1:0.02208512472057182208,Excavata-
Malawimonas_jakobiformis_Contig1448_3:0.02711477183341675160)93:0.09650344953468760478)100:0.1789361205385614
2174,Amoebozoa-
Hartmannella_vermiformis_Contig1413_1:0.34410128648636362225)44:0.07154876291570842761)6:0.0531963870670393163
0)4:0.04322837445047359589,(Amoebozoa-Acanthamoeba_castellanii_Contig1063_2:0.38974981495958138655,Excavata-
Naegleria_gruberi_83065-
estExt_fgeneshNG_pm.C_150011:0.22098085850115176121)18:0.08494081708590495161)2:0.02571346534068047820)0:0.01
682876690993876517)9:0.08250765855195150988,Rhizaria-
Reticulomyxa_filosa_EE664964_4:0.39205538608763762509)24:0.02833184885802074449)32:0.06896774592858997965,(((((
Bacteria-Azoarcus_sp._56479267:0.23394563608189120063,Bacteria-
Chromobacterium_violaceum_34496517:0.05990630092970310183)54:0.04351658772400360947,((Bacteria-
Xylella_fastidiosa_28198404:0.11793064999717803309,(Bacteria-
Stenotrophomonas_maltophilia_119879233:0.03066290941044075985,Bacteria-
Xanthomonas_oryzae_58580594:0.03130235874121916478)86:0.02870645108990083766)99:0.05972773571267514514,Opisth
okonta-
Apis_mellifera_66557775:0.24481805019869365414)72:0.05673682770299486033)21:0.02425558188627079462,((Bacteria-
Bordetella_bronchiseptica_33602659:0.13289622584366009295,(Bacteria-
Polynucleobacter_sp._145588941:0.08503265184589015035,((Bacteria-
Ralstonia_pickettii_153887608:0.06178763330143214466,((Bacteria-
Methylibium_petroleiphilum_124267359:0.04808948897925909016,(Bacteria-
Rhodoferax_ferrireducens_89900592:0.07606233256845935931,(Bacteria-
Verminephrobacter_eiseniae_121611270:0.10703811536095265977,Bacteria-
Polaromonas_sp._91789449:0.00000121823947223981)78:0.02073327670359154801)92:0.04577418381086270638)76:0.02448
817545348611763,(Bacteria-Acidovorax_avenae_120610880:0.01488262007756183287,(Bacteria-
Comamonas_testosteroni_118050993:0.01817755268714634637,Bacteria-
Delftia_acidovorans_118732119:0.02985764423744473317)100:0.04318629385224369288)92:0.03599210850494088954)89:0.
04554672253173984442)68:0.03774381030375807689,(Bacteria-
Janthinobacterium_sp._152980043:0.02277702411949962535,Bacteria-
Herminiimonas_arsenicoxydans_134094986:0.01785586538373707538)100:0.05585650618884058921)46:0.037028671560325
90978)38:0.02391106150755131776)75:0.05047978143699610731,(Bacteria-
Limnobacter_sp._149926167:0.13492969488524389488,(Bacteria-
Xanthobacter_autotrophicus_154245617:0.25101531874272953182,Bacteria-
Burkholderia_phytofirmans_118036548:0.05946607423187577623)75:0.03789001502477941374)56:0.03901143441846210286
)14:0.01977047423262588699)83:0.11592436901928457327,(Bacteria-
Nitrosomonas_eutropha_114331619:0.30861781517571451605,Bacteria-
Nitrosospira_multiformis_82701993:0.21850077605399567671)96:0.08566749192601270935)73:0.06778837819903107964,((B
acteria-Thermus_thermophilus_46198476:0.23274461666819196148,Bacteria-
Deinococcus_geothermalis_94986260:0.15538473633711746835)49:0.03893087659052595073,(((Bacteria-
Halorhodospira_halophila_121997621:0.17605549478029661192,Bacteria-

Alkalilimnicola_ehrlichei_114319648:0.1175795745449369905 7)61:0.04376378977039738216,(((Bacteria-
Psychrobacter_cryohalolentis_93006868:0.1508778267789628591 0,((Bacteria-
Saccharophagus_degradans_90021304:0.13235495361225610433,Bacteria-
Alcanivorax_borkumensis_110834109:0.08932689870349048988)49:0.04189048297601399157,Bacteria-
marine_gamma_119503152:0.17483582448013940125)42:0.03235072724081115847)27:0.02153802883062925014,Bacteria-
Desulfotalea_psychrophila_51244513:0.45938889223328255307)40:0.03163511239855926888,(Bacteria-
Methylococcus_capsulatus_53805062:0.37560771990999142211,Bacteria-
Oceanospirillum_sp._89095300:0.38497579302629170162)64:0.12172269783972868018)14:0.01064429453258698872)18:0.04
279006539520975594,((Plantae-Cyanophora_paradoxa_Contig541_5:0.28181934175689721256,Amoebozoa-
Acanthamoeba_castellanii_Contig914_2:0.54118679680931314202)74:0.25050027560234172652,(((Plantae-
Galdieria_sulphuraria_A4_12H05_3:0.43929480368533918577,(((((((Plantae-
Chondrus_crispus_Contig256_2:0.11073348167324396918,Plantae-
Gracilaria_changii_Contig518_4:0.06505255024790258800)89:0.07973426711216621832,Plantae-
Porphyra_yezoensis_Contig542_2:0.16784592977344281461)81:0.07201615315462844835,Plantae-
Porphyra_yezoensis_Contig321_2:0.27120079142919867321)78:0.11506472895004221657,Plantae-
Cyanidioschyzon_merolae_CMP193C:0.24466063070663934820)43:0.06552628833231206873,Plantae-
Chondrus_crispus_Contig317_1:0.20197716076951738318)57:0.07363154559007042965,Plantae-
Porphyra_yezoensis_AV437295_1:0.18262228660740847941)73:0.02970170960968150295,Amoebozoa-
Acanthamoeba_castellanii_Contig971_1:0.31944105641882641899)32:0.05711049993401617897)20:0.0807814008692773755
1,((Excavata-Naegleria_gruberi_31160-e_gw1.8.144.1:0.39644554501800094437,Excavata-Naegleria_gruberi_60960-
fgeneshNG_pm.scaffold_70000001:0.32324442318880580727)34:0.09578253399203888463,((Excavata-
Malawimonas_jakobiformis_Contig1332_2:0.15976360689203317200,(Plantae-
Porphyra_yezoensis_AU195687_1:4.74988470624374325979,Excavata-
Malawimonas_californiana_110030533_3:0.00000121823947223981)11:0.19163597654016670258)11:0.233861199858103885
33,((Excavata-Jakoba_bahamiensis_Contig14_3:0.48791236740582105691,Excavata-
Jakoba_libera_Contig518_3:0.14894355423548766293)98:0.17788094723446298828,(Rhizaria-
Reticulomyxa_filosa_EE664753_5:0.43265464443357326552,(Chromalveolata-
Tetrahymena_thermophila_118346673:0.21941125838520544877,((Chromalveolata-
Paramecium_tetraurelia_124392513:0.00124694160901298165,Chromalveolata-
Paramecium_tetraurelia_124418922:0.11244336755927747395)100:0.07766635915396410961,Chromalveolata-
Paramecium_tetraurelia_124391972:0.00000121823947223981)100:0.29655324505450408878)88:0.08047974688641859609)3
6:0.06600749286404428151)17:0.01193047364202728944)8:0.06098733573312454387)2:0.03806558218075224709)0:0.01515
040496022437670,Plantae-
Glaucocystis_nostochinearum_Contig579_1:0.28677389271936154502)0:0.00000121823947223981)2:0.084852535159247233
22)2:0.03556081228016452073)3:0.03016024548699093935)5:0.05410211346224129414)10:0.02850097009689400718)13:0.0
6860734962660189340)94:0.08205684258815251764)89:0.07481591386523032960)38:0.05389998666658464554,Bacteria-
Rubrobacter_xylanophilus_108804129:0.36110948988743912791);

Plantae-Arabidopsis_thaliana_42563306

(((((Bacteria-Cytophaga_hutchinsonii_110638646:0.74571960964810812822,((((((Bacteria-
Lactobacillus_casei_116495592:0.07402353056880323079,Bacteria-
Pediococcus_pentosaceus_116493438:0.12065510695494599536)90:0.12948012210924284004,(Bacteria-
Oenococcus_oeni_116490407:0.66252156819998497816,Bacteria-
Leuconostoc_mesenteroides_116618953:0.22208673522220540031)19:0.04570576842392530537)25:0.0902822698940178580
7,Bacteria-Streptococcus_mutans_24379074:0.31029182040683489507)20:0.04797302138512623410,(Bacteria-
Neisseria_meningitidis_15794692:0.27178315476500558567,Bacteria-
Listeria_monocytogenes_153175470:0.16204601393115411478)48:0.07710287344794797271)12:0.06329695363907199279,(B
acteria-Staphylococcus_haemolyticus_70725637:0.47164335580526822733,(((((Opisthokonta-
Drosophila_melanogaster_85725270:0.00000121823947223981,Opisthokonta-
Drosophila_melanogaster_24650981:0.00000121823947223981)34:0.00000121823947223981,Opisthokonta-
Drosophila_melanogaster_85725272:0.00000121823947223981)100:0.07331388311924832979,Opisthokonta-
Apis_mellifera_66550890:0.19227031365590077638)74:0.07854943313924009352,Opisthokonta-
Drosophila_melanogaster_24646216:0.15107746601520438690)100:0.35612569534494264989,((((Bacteria-
Nitrosomonas_europaea_30249740:0.22469369625381735434,Bacteria-
Chlorobium_phaeobacteroides_119356462:0.20693486511044065224)33:0.05699803589839209927,Bacteria-
Alcanivorax_borkumensis_110834630:0.23508665681556806248)36:0.03617671580864570413,(((Bacteria-
Treponema_denticola_42527203:0.21889469300260300244,Bacteria-
Clostridium_phytofermentans_106885052:0.21733351955170293612)92:0.16296070418647312650,(((Bacteria-
Klebsiella_pneumoniae_152969325:0.01380082572983078529,(Bacteria-
Yersinia_pestis_45440859:0.07378300892372072206,Bacteria-
Enterobacter_sp._146310904:0.03167612414610425364)54:0.00918594450411409739)70:0.02597212996679712158,Bacteria-
Salmonella_enterica_16759697:0.00000121823947223981)40:0.01506274521181573053,((Bacteria-

Shigella_boydii_82543175:0.00000121823947223981,Bacteria-
Escherichia_coli_157155502:0.00506400687972225275)92:0.01024572777482796182,Bacteria-
Citrobacter_koseri_157146620:0.01535598709502820208)42:0.00000121823947223981)100:0.16370278691089690248)29:0.1
0461272658424262960,((((((((Chromalveolata-Phytophthora_sojae_108887:0.29901231971929292941,Chromalveolata-
Phytophthora_sojae_108482:0.16917546226039725799)48:0.06163175296668376574,Rhizaria-
Bigelowiella_natans_DR040394_3:0.18680294620580134568)40:0.04090815395754641287,(Chromalveolata-
Phaeodactylum_tricornutum_33839:0.15243436638631943647,Chromalveolata-
Thalassiosira_pseudonana_27850:0.12513197606060411093)98:0.17451143904663457551)45:0.05209387583376126618,(Chro
malveolata-Phaeodactylum_tricornutum_17086:0.16583447767658823779,Chromalveolata-
Thalassiosira_pseudonana_28350:0.22480954830786445409)91:0.08797143643742859342)66:0.08181035892196494852,(Chro
malveolata-Toxoplasma_gondii_92399533:0.16462430461118596203,Chromalveolata-
Plasmodium_falciparum_124804024:0.15854730808881700721)99:0.15717211509974912675)20:0.05362996532873295225,(E
xcavata-Naegleria_gruberi_35141-estExt_gwp_gw1.C_40123:0.21621103566841573529,Amoebozoa-
Acanthamoeba_castellanii_Contig316_3:0.19635264154088372113)12:0.02909814804315494219)2:0.02528654104245429621,
Bacteria-Borrelia_garinii_51598913:0.34506025623759012388)0:0.01291504702078752863,Bacteria-
Thermoanaerobacter_ethanolicus_114844470:0.20153817780006649807)1:0.00000121823947223981)3:0.06427968241682466
277)4:0.07778596788856152744,((Chromalveolata-Phytophthora_sojae_157951:1.78540968212313422292,Chromalveolata-
Thalassiosira_pseudonana_17651:0.44698561477398301989)7:0.39387798260532480565,(((Bacteria-
Actinomyces_odontolyticus_154508490:0.21474462423105333819,((Bacteria-
Corynebacterium_jeikeium_68537006:0.24030671637437114874,Bacteria-
Mycobacterium_smegmatis_118471051:0.15327851590353414046)48:0.05786370277869509748,(Bacteria-
Rhodococcus_sp._111019063:0.14125109637896066106,(Bacteria-
Saccharopolyspora_erythraea_134103393:0.05982311256265924332,(Bacteria-
Streptomyces_avermitilis_29830522:0.16923089325030807317,Bacteria-
Frankia_sp._86739183:0.19300990418843563812)48:0.07664340274668084152)41:0.08044289520611168365)13:0.015664867
91240480694)15:0.06850103477528070306)13:0.03085593132649268322,Bacteria-
Propionibacterium_acnes_50841849:0.28287306596025607597)28:0.05219969174245660259,(Bacteria-
Tropheryma_whipplei_28493622:0.48360749714692280454,Bacteria-
Nitratiruptor_sp._152990666:0.53458527751299567132)39:0.12621390420663353149)62:0.14725121088987058737)1:0.06153
411752547125607)1:0.03446189467094527142)2:0.07342767023935359294)0:0.00846278692296353365)1:0.03175856525032
464545,(Bacteria-Desulfovibrio_vulgaris_46581339:0.43168437574593104822,Chromalveolata-
Aureococcus_anophagefferens_14005:0.50741907464567614916)35:0.15912443203380544077)8:0.13355191816114217462)15
:0.11746851883671627359,(((Bacteria-Myxococcus_xanthus_108762943:0.47323809708562747378,(Bacteria-
Parvibaculum_lavamentivorans_154254001:0.21484463673729109479,(((Opisthokonta-Sporobolomyces_roseus_12139-
e_gw1.4.676.1:0.29050142663701988832,Opisthokonta-
Ustilago_maydis_71022513:0.26740217678493349851)61:0.11252265804130802407,Opisthokonta-
Schizosaccharomyces_pombe_19115801:0.27450837295849145336)36:0.09209407469706175353,(Bacteria-
Stappia_aggregata_118590507:0.26320167941338923345,((Bacteria-
Methylobacterium_chloromethanicum_156450008:0.28997458194965458622,(((Bacteria-
Mesorhizobium_sp._110635917:0.25557838548125250133,(Bacteria-
Bartonella_bacilliformis_121602512:0.35325969564330644834,(Bacteria-
Ochrobactrum_anthropi_153008660:0.02589239349261963224,Bacteria-
Brucella_ovis_148558100:0.02733161056885962531)87:0.02760104985029088187)65:0.03954844264688067712)41:0.039352
41653266863260,(Bacteria-Aurantimonas_sp._90420658:0.13403200001173209976,Bacteria-
Fulvimarina_pelagi_114705033:0.22286919365940630056)53:0.06214044732291201800)37:0.01708905318412380289,(Bacter
ia-Rhizobium_etli_86355829:0.08908941365317381289,Bacteria-
Sinorhizobium_medicae_150398561:0.05240695615582870864)100:0.16106251845336752582)71:0.07648396144773368655)6
0:0.04922390972760716521,(Bacteria-Bradyrhizobium_sp._146337326:0.07325609463822609080,(Bacteria-
Nitrobacter_hamburgensis_92115791:0.13837999193150465893,Bacteria-
Rhodopseudomonas_palustris_90421860:0.07293087622615671006)83:0.03887759929366417511)89:0.12036476124769043827)66:0.10759221699271409589)68:0.04326826552342563637)28:0.07195141279318165162)100:0.24636356738226380947)40
:0.08662246981042130700,Bacteria-
Pseudoalteromonas_atlantica_109898907:0.71590876363133582050)32:0.03702740540476123776,(Cyanobacteria-
Gloeobacter_violaceus_PCC_7421_37521444:0.68196367400537249992,(Cyanobacteria-
Gloeobacter_violaceus_PCC_7421_37521165:0.26396085919252121332,Cyanobacteria-
Cyanothece_sp._CCY0110_126660142:0.22639067568288456789)100:0.37662202111764220280)7:0.05349551095937061379)
15:0.08018311446021707256)85:0.31439225995094871591,((Chlamydia-
Chlamydophila_pneumoniae_15836396:0.00511188029789079110,((Chlamydia-
Chlamydophila_pneumoniae_33242223:0.00000121823947223981,Chlamydia-
Chlamydophila_pneumoniae_15618772:0.00000121823947223981)37:0.00000121823947223981,Chlamydia-
Chlamydophila_pneumoniae_16752176:0.00000121823947223981)61:0.00000121823947223981)100:0.1083363834795755265
0,((Chlamydia-Chlamydophila_felis_89897917:0.02010236211879717905,(Chlamydia-

Chlamydophila_abortus_62185474:0.05455294433710617535,Chlamydia-
Chlamydophila_caviae_29840659:0.06103568877801975046)27:0.01043603530089079814)98:0.10863685427751854085,(Chla
mydia-Chlamydia_muridarum_15834720:0.05188530228108870906,(Chlamydia-
Chlamydia_trachomatis_15605455:0.00000121823947223981,Chlamydia-
Chlamydia_trachomatis_76789462:0.00000121823947223981)100:0.05493554911927019807)86:0.10654289280523768224)23:
0.01889775017620376474)97:0.40352109604939789822)46:0.02321846860401242940,Chlamydia-
Candidatus_Protochlamydia_46445795:0.28346984817713705151)99:0.25209026061770145599,(Plantae-
Oryza_sativa_115448715:0.07214536406906355004,(Plantae-
Arabidopsis_thaliana_42563306:0.10918049267574343741,Plantae-
Arabidopsis_thaliana_18395083:0.01911942972398899057)73:0.03444524166706939422)64:0.03255425373127491390,Plantae
-Physcomitrella_patens_130367:0.19787975861845366610);

Plantae-Arabidopsis_thaliana_42565237
(Chromalveolata-Phaeodactylum_tricornutum_47290:0.34152919394495162564,(Chromalveolata-
Phaeodactylum_tricornutum_44055:0.95271387889918479619,(Chlamydia-
Candidatus_Protochlamydia_46447632:0.69359612361918365764,((Bacteria-
Geobacter_lovleyi_118746507:0.65442028957446385373,((Bacteria-
Lactococcus_lactis_125625220:0.96194363194432563891,Bacteria-
Alkaliphilus_metalliredigens_150391752:0.40321798597096575323)39:0.14219237658763467236,Bacteria-
Leptospira_borgpetersenii_116330374:1.07411587877184566686)79:0.34067002824936926109)100:1.10023884843271768652,
((Plantae-Volvox_carteri_41894_jgi:1.06945273665824647225,((Plantae-
Arabidopsis_thaliana_42565237:0.26029680203216709611,Plantae-
Oryza_sativa_115441879:0.18019732725013840047)100:0.19728885615446642432,Plantae-
Physcomitrella_patens_12476:0.32833235083920692121)91:0.16807624259941653100)56:0.11696457071197216560,Plantae-
Ostreococcus_lucimarinus_24318:1.13958298946834690746)43:0.06314004813302181995)51:0.11804192217291262224)89:0.
27149475894526869935)100:0.46707084436994178445,Chromalveolata-
Thalassiosira_pseudonana_22499:0.30945465669579114154);

Plantae-Arabidopsis_thaliana_42573275
(Plantae-Arabidopsis_thaliana_42571387:0.00000121823947223981,((Plantae-
Arabidopsis_thaliana_30690721:0.09965413760414761069,Plantae-
Arabidopsis_thaliana_30690718:0.04369681644756376326)94:0.12484573190855233205,((Plantae-
Arabidopsis_thaliana_30681012:0.00490719908498091217,(Plantae-
Arabidopsis_thaliana_15223003:0.00000121823947223981,Plantae-
Arabidopsis_thaliana_30680348:0.00000121823947223981)93:0.00000121823947223981)98:0.00000121823947223981,(Planta
e-Oryza_sativa_115445445:0.51327194493731742586,((Plantae-
Chlamydomonas_reinhardtii_184947:0.09241428906044540925,Plantae-
Volvox_carteri_57561_jgi:0.06240046567344433720)100:0.40644742126317451314,(Plantae-
Physcomitrella_patens_17202:0.26284567370357997396,((Bacteria-
Coxiella_burnetii_95926268:0.84015897779089565578,(((Chlamydia-
Chlamydia_trachomatis_76789051:0.00990798765098578962,Chlamydia-
Chlamydia_trachomatis_15605050:0.00000121823947223981)97:0.12348541359549242147,Chlamydia-
Chlamydia_muridarum_15835220:0.13459947972603389377)100:0.67964406274546784736,(Chlamydia-
Chlamydophila_caviae_29840323:0.04963035427680887596,Chlamydia-
Chlamydophila_felis_89898244:0.03367252079861638575)100:0.41239433844530848372)100:0.47644168939807757246)100:
0.28120461990085754422,(Plantae-Ostreococcus_tauri_19960:0.20384843195120580517,Plantae-
Ostreococcus_lucimarinus_6627:0.18633783263658251772)100:0.43044873047130266519)73:0.05766759435403657347)76:0.
09634993364842300845)100:0.32634506093523413250)99:0.18278387110720609177)100:0.12516149466726569917)100:0.05
036588438322806005,Plantae-Arabidopsis_thaliana_42573275:0.00632030907275514357);

Plantae-Arabidopsis_thaliana_42573371
(Plantae-Physcomitrella_patens_130288:0.15561103289878525091,((((Bacteria-
Magnetococcus_sp._117925780:0.72294899061622519554,(((Bacteria-
Paracoccus_denitrificans_119385429:0.13358962430206333671,Bacteria-
alpha_proteobacterium_114769823:0.35203273503811954859)65:0.06360088980344873189,(((Bacteria-
Roseobacter_denitrificans_110677715:0.28728360537468200508,Bacteria-
Silicibacter_sp._99078433:0.12839904121879430754)42:0.05184309605630278706,(Bacteria-
Oceanicola_granulosus_89070052:0.16920738531570397867,Bacteria-
Sagittula_stellata_126732700:0.14279236034392236987)19:0.02659467887493660240)27:0.03766726863714606188,Bacteria-
Roseovarius_sp._149201317:0.19427314875183437914)52:0.04408117866750572988)82:0.08780917002712713970,Bacteria-
Jannaschia_sp._89052599:0.38288049538314655740)99:0.29617985468172203234)36:0.07276681439553572828,(((Bacteria-
Xanthobacter_autotrophicus_154248069:0.40188852117534140662,(Bacteria-

Solibacter_usitatus_116620283:0.24502620784983614177,Bacteria-
Acidiphilium_cryptum_148259679:0.54860776204958439095)47:0.06549883401658887494)86:0.16488897912380851007,((((
Bacteria-Maricaulis_maris_114569716:0.73628417412767166450,(Bacteria-
Sphingomonas_wittichii_148553167:0.21755904348901536349,(Bacteria-
Sphingopyxis_alaskensis_103486768:0.15573139637078958764,(Bacteria-
Erythrobacter_sp._149184573:0.28141502247966321271,Bacteria-
Novosphingobium_aromaticivorans_87199937:0.25901174384979019916)52:0.02921218878776942265)87:0.10945753647715
014689)96:0.22665998466865075467)18:0.02014642238327119667,Bacteria-
Hyphomonas_neptunium_114797544:0.51618873669500819901)54:0.06117586377119555580,(Plantae-
Ostreococcus_tauri_6912:0.25312615945879302082,Plantae-
Ostreococcus_lucimarinus_8182:0.10198962811834798303)100:0.61811515362124347384)40:0.08263771616220511040,(((Ba
cteria-Rhodopseudomonas_palustris_90421758:0.08578285394573988143,(Bacteria-
Bradyrhizobium_sp._146337509:0.06056366555825030973,Bacteria-
Nitrobacter_hamburgensis_92119051:0.20565949345952211358)99:0.13822406838223075254)100:0.24804811603294221745,(
Bacteria-Methylobacterium_sp._149121730:0.22844552343318821896,Bacteria-
Bordetella_bronchiseptica_33599395:0.38203848070759899302)63:0.07727950093467679527)96:0.13982680282603901500,(B
acteria-Stappia_aggregata_118592049:0.51967659151000544782,(((((Bacteria-
Rhizobium_etli_86359681:0.12503314138722704252,Bacteria-
Agrobacterium_tumefaciens_15889755:0.13830242006638993058)89:0.05600628761146698303,Bacteria-
Sinorhizobium_medicae_150398377:0.09699786792820926096)85:0.09429047411982004190,(Bacteria-
Mesorhizobium_sp._110635552:0.12903495388011987366,Bacteria-
Ochrobactrum_anthropi_153008408:0.16940278126827620908)43:0.03983106301703009944)58:0.06890045566201770388,(B
acteria-Fulvimarina_pelagi_114705845:0.26258149571911831854,Bacteria-
Aurantimonas_sp._90421028:0.14988931042143696648)96:0.10547211515391546888)29:0.05564133368425496634,Bacteria-
Bartonella_bacilliformis_121601926:0.26604161377660046073)98:0.15386715573614759278)23:0.04269141349292665533)95
:0.17268650278488589800)34:0.09429048667801814831)25:0.08202146237771361370,(Bacteria-
Parvibaculum_lavamentivorans_154253501:0.62537528982102907893,(((Excavata-
Jakoba_libera_Contig148_1:0.69173313460204477465,((Opisthokonta-
Magnaporthe_grisea_39945258:0.79592664139997526540,(Chromalveolata-
Paramecium_tetraurelia_124426827:0.00000121823947223981,((Chromalveolata-
Paramecium_tetraurelia_124410881:0.01856064797712060968,Chromalveolata-
Paramecium_tetraurelia_124429701:0.05150255628139407960)97:0.07874278861536984642,(Chromalveolata-
Paramecium_tetraurelia_124390987:0.06051444386002048648,((Chromalveolata-
Paramecium_tetraurelia_124390937:0.05314636713832138898,Chromalveolata-
Paramecium_tetraurelia_124403883:0.07524460060895186986)89:0.03629634240203735707,Chromalveolata-
Paramecium_tetraurelia_124401814:0.04391288823261182278)99:0.13308356440051838376)33:0.07928557756026465009)33:
0.05813151185644862412)98:0.52637364337963565841)100:0.95330535911953884209,((Bacteria-
Desulfitobacterium_hafniense_89894274:0.38113359230796867161,(Bacteria-
Wolinella_succinogenes_34556729:0.31736644631650101633,Bacteria-
Caminibacter_mediatlanticus_149194049:0.38248946269044659108)65:0.09779853114984186913)100:0.281503116629519822
83,(((Plantae-Glaucocystis_nostochinearum_Contig1080_1:0.47535244173766799669,Chlamydia-
Candidatus_Protochlamydia_46446564:0.45100684643274968622)100:0.37110737814028177395,(Bacteria-
Pseudomonas_aeruginosa_116053823:0.50539382576422053894,(((Cyanobacteria-
Synechococcus_elongatus_PCC_7942_81300256:0.00000121823947223981,Cyanobacteria-
Synechococcus_elongatus_PCC_6301_56750119:0.00414074717136988981)100:0.20854458474133161228,((Cyanobacteria-
Synechocystis_sp._PCC_6803_16330758:0.13252933925275467431,(((Cyanobacteria-
Cyanothece_sp._CCY0110_126658594:0.03861451416032900957,Cyanobacteria-
Cyanothece_sp._CCY0110_126658699:0.10908040661381583625)71:0.02311368936881630365,Cyanobacteria-
Crocosphaera_watsonii_WH_8501_67923261:0.02252090343181326060)79:0.03127317179410203651)85:0.081497091001712
11029,Cyanobacteria-
Nostoc_punctiforme_PCC_73102_23128982:0.15663386049404254274)36:0.04805181469264865196)92:0.1150139724486760
6062,Cyanobacteria-
Anabaena_variabilis_ATCC_29413_75908384:0.26493617470581670714)99:0.17167251851440090693)93:0.19616801372603
667741)87:0.16881112878232498731,((Bacteria-Arcobacter_butzleri_157736514:0.26892396878205859778,Chlamydia-
Candidatus_Protochlamydia_46446692:0.61194639203927325699)49:0.11798060691366446529,(Bacteria-
Nitratiruptor_sp._152990078:0.29816910620197228932,Excavata-
Malawimonas_californiana_Contig11_2:0.67810052867647585551)29:0.06414974088208970360)96:0.22506607912811152361
)53:0.05796334448572122017)62:0.08401589870684411654)74:0.17356517444585264909)43:0.12881922935593273505,(((Ba
cteria-Desulfovibrio_vulgaris_46580187:0.31424608263727399349,((Bacteria-
Bdellovibrio_bacteriovorus_42523716:0.67054972905898480029,(Bacteria-
Ralstonia_metallidurans_94311016:0.39585284729476521992,Bacteria-
Methylococcus_capsulatus_53804236:0.72932495539340425061)30:0.04376000999967930710)7:0.04810909804325881611,Ba

cteria-
Lawsonia_intracellularis_94986910:0.63074678471585976514)15:0.03530621922430558085)19:0.07913259769113771547,((B
acteria-Pelobacter_carbinolicus_77919584:0.33869941705857142811,Bacteria-
Geobacter_lovleyi_118746697:0.29006382478554176840)23:0.04673296043333222127,Bacteria-
Desulfuromonas_acetoxidans_95931147:0.30616218038335463181)5:0.02263713507839801645)23:0.03621031946247991323,
Bacteria-
marine_gamma_119475057:0.81361925721371242126)31:0.09766629363505491268)10:0.07262149275372910273,(Bacteria-
Psychromonas_sp._90408545:0.51133524465210811183,Bacteria-
Coxiella_burnetii_154705747:0.72917211447256946588)25:0.09120379269785360010)4:0.07312705787014260606)3:0.00000
121823947223981)8:0.12279874759534535611)100:0.38492601941148785194,((((Plantae-
Arabidopsis_thaliana_30696223:0.00803752847037786204,Plantae-
Arabidopsis_thaliana_79320225:0.00000121823947223981)66:0.00000121823947223981,Plantae-
Arabidopsis_thaliana_30696219:0.01908711930629697004)100:0.48005246483644536948,((Plantae-
Arabidopsis_thaliana_79326253:0.00401055872759721599,Plantae-
Arabidopsis_thaliana_18418245:0.00000121823947223981)100:0.24660174743104343320,Plantae-
Oryza_sativa_115479631:0.24868063507149881786)52:0.03133831760797286398)99:0.36904186798046506590,((Plantae-
Oryza_sativa_115438793:0.40075373462797819757,((Plantae-
Arabidopsis_thaliana_15220853:0.14621227638349557076,(Plantae-
Arabidopsis_thaliana_15223141:0.00000121823947223981,(Plantae-
Arabidopsis_thaliana_42572057:0.00000121823947223981,Plantae-
Arabidopsis_thaliana_30698715:0.00000121823947223981)33:0.00000121823947223981)100:0.09161512858767549561)99:0.
17753802904649695904,(((Plantae-Arabidopsis_thaliana_79327881:0.00000121823947223981,Plantae-
Arabidopsis_thaliana_79327873:0.00835957168366795178)59:0.00000121823947223981,(Plantae-
Arabidopsis_thaliana_42573371:0.00000121823947223981,Plantae-
Arabidopsis_thaliana_30685030:0.00000121823947223981)67:0.00000121823947223981)99:0.03320697211177443126,(Planta
e-Arabidopsis_thaliana_79295433:0.03964940278400410495,(Plantae-
Arabidopsis_thaliana_30678347:0.00000121823947223981,(Plantae-
Arabidopsis_thaliana_30678350:0.00000121823947223981,Plantae-
Arabidopsis_thaliana_30678353:0.00000121823947223981)27:0.00000121823947223981)95:0.00000121823947223981)94:0.0
5900746426971138076)100:0.16556016541124052299)86:0.12498085505555804609)84:0.16561767681007563691,(Plantae-
Physcomitrella_patens_205864:0.16950143353395050183,Plantae-
Physcomitrella_patens_162454:0.10771061149221833331)100:0.36772104629164398260)35:0.04490140841366047769)71:0.1
8765846101532637769)99:0.20173688700015024455,Plantae-
Physcomitrella_patens_147703:0.31061355874891777296)76:0.06737760204312501222,Plantae-
Physcomitrella_patens_123406:0.15367015059478159045);

Plantae-Chlamydomonas_reinhardtii_104193
(Cyanobacteria-Cyanothece_sp._CCY0110_126658167:0.06825500980045920429,(((((Cyanobacteria-
Nostoc_sp._PCC_7120_17228722:0.01205630852563517597,Cyanobacteria-
Anabaena_variabilis_ATCC_29413_75906868:0.01583521251112155262)97:0.01833548008834672352,(Cyanobacteria-
Nodularia_spumigena_CCY9414_119509215:0.05366903712111954078,Cyanobacteria-
Nostoc_punctiforme_PCC_73102_23130352:0.03731931054360528716)42:0.00668387104419814696)100:0.087805518748214
48943,(Cyanobacteria-Thermosynechococcus_elongatus_BP-1_22298534:0.10930303387252081337,(((Bacteria-
Prosthecochloris_vibrioformis_145219156:0.04733748722683246701,(Bacteria-
Pelodictyon_luteolum_78186164:0.07916317931282931819,Bacteria-
Chlorobium_phaeobacteroides_119358060:0.19576701636332677348)75:0.05474044510736555957)100:0.5017809571041587
0911,(((Chlamydia-Candidatus_Protochlamydia_46447621:1.16991085293136665157,(((Chlamydia-
Candidatus_Protochlamydia_46447213:0.63551342894129780881,((Plantae-
Ostreococcus_tauri_10633:0.23089778000282901171,Plantae-
Ostreococcus_lucimarinus_31136:0.09832583542072138860)100:0.57003376249287907029,(Plantae-
Chlamydomonas_reinhardtii_104193:0.30468449541285258508,Plantae-
Volvox_carteri_108215_jgi:0.21940800228216100765)100:0.25811468879567117662)100:0.26535528426780302658)99:0.358
14892966481892689,((Bacteria-Marinomonas_sp._152997627:0.78369919172557933607,(Bacteria-
Psychrobacter_sp._148654121:0.59025747546271634114,(Bacteria-
Alcanivorax_borkumensis_110835023:0.43366052195557247551,(((Bacteria-
Ralstonia_eutropha_73541045:0.12805629193434961421,Bacteria-
Burkholderia_phytofirmans_118044115:0.13217777051779097675)100:0.53467859605574730164,((Bacteria-
Delftia_acidovorans_118729031:0.21765234568106889279,Bacteria-
Acidovorax_avenae_120609942:0.12340203083271560069)100:0.15748583373907051874,Bacteria-
Polaromonas_naphthalenivorans_121604209:0.22388901285814627462)100:0.17195089304227115234)84:0.152246650370833
40291,(Bacteria-Shewanella_denitrificans_91793740:0.67375372291582869000,Bacteria-
marine_gamma_119504827:0.71080329394531249765)14:0.06647818577003902774)29:0.11300006847936368282)48:0.15298

823288108859275)90:0.21431855869996810360)100:0.46952175281178853972,Bacteria-
Desulfuromonas_acetoxidans_95931069:1.26425217687355062601)43:0.11002433528065330703)100:0.31607388181411721906,(Bacteria-Herpetosiphon_aurantiacus_113940803:0.22814177529892171181,(Bacteria-
Roseiflexus_sp._148655022:0.15815889759622275834,Bacteria-
Chloroflexus_aggregans_118047790:0.22321047221984283149)74:0.08806659182583756462)100:0.48810228386710519111)90:0.16648277969064184667)17:0.03903838609645404051,Bacteria-
Saccharopolyspora_erythraea_134103510:0.87138781116199104382)10:0.04236182968162195694,((Bacteria-
Anaeromyxobacter_sp._153006747:0.72145394718088207142,(Bacteria-
Treponema_denticola_42528231:0.93027393256217705275,(Bacteria-
Geobacter_bemidjiensis_145618691:0.38486562630378684524,Bacteria-
Pelobacter_carbinolicus_77919588:0.50423913470314130070)92:0.17988801967670833837)20:0.03768375038305546432)14:0.09788338492079197428,((((Bacteria-Dechloromonas_aromatica_71909277:0.34976299779481895280,Bacteria-
Hahella_chejuensis_83648649:0.39536951778399320734)100:0.29053064289128638364,(Bacteria-
Sphingomonas_wittichii_148553172:0.40265105278913299047,((Bacteria-
Rhodobacter_sphaeroides_126462226:0.24553499547496440703,((Bacteria-
Roseobacter_denitrificans_110679975:0.34389842521249847085,Bacteria-
Silicibacter_sp._99081204:0.22157678754714374425)82:0.07561444271836682829,(Bacteria-
Paracoccus_denitrificans_119386836:0.45291427587194726945,(Bacteria-
Roseovarius_sp._114762893:0.11176152006391681937,Bacteria-
Sagittula_stellata_126731334:0.11466600934260579026)99:0.08474724374294387297)37:0.05045319501596661621)99:0.15519767921812158140)67:0.09094427056918014118,(Bacteria-
Methylobacterium_chloromethanicum_156449364:0.45086964891693404045,(Bacteria-
Ochrobactrum_anthropi_153009400:0.42368588200042534009,Bacteria-
Aurantimonas_sp._90419633:0.40093764951730520574)94:0.16849006306851641024)80:0.09670609464643914466)100:0.33428465584668087818)98:0.17468007016009354238)98:0.25918679961707835613,Bacteria-
delta_proteobacterium_94266587:0.92820852252944774374)1:0.03101341242125732478,(Bacteria-
Moorella_thermoacetica_83589019:0.56389673115279770332,(((Bacteria-
Caldicellulosiruptor_saccharolyticus_146295635:0.78295608467838195832,Bacteria-
Carboxydothermus_hydrogenoformans_78044326:0.30672099215066678246)11:0.11967231556087867816,(Bacteria-
Desulfotomaculum_reducens_134298050:0.31384301057240820976,Bacteria-
Pelotomaculum_thermopropionicum_147676594:0.23475684817638045598)43:0.05200873504569256123)8:0.07362264680806250572,((Bacteria-Thermosinus_carboxydivorans_121535055:0.29658709850469472391,((Bacteria-
Alkaliphilus_metalliredigens_150392209:0.28009213127456561443,(Bacteria-
Clostridium_difficile_126701180:0.31672558470646494655,Bacteria-
Thermoanaerobacter_ethanolicus_114843410:0.26863363689672614676)48:0.05825035781620388486)90:0.08656188161577296758,(Bacteria-Ruminococcus_torques_153815945:0.22570677394354041190,Bacteria-
Dorea_longicatena_153853506:0.14673720968531359099)100:0.25095302917422024347)75:0.10814147538041442298)25:0.05325072330710769325,(Bacteria-Symbiobacterium_thermophilum_51894293:0.51825985107974315014,(Bacteria-
Bacillus_sp._126653300:0.13327701629996044352,(Bacteria-
Lactobacillus_brevis_116333187:0.50775536032259693808,Bacteria-
Listeria_welshimeri_116871611:0.12733122813421085540)83:0.11186662314798716256)100:0.42439885817205563745)32:0.10979488575773400927)16:0.04198625198472247244)14:0.07755088465631516936)19:0.08306623423233726322)1:0.02217298469497267638)1:0.05595814399103027359)47:0.11574910212601376147)100:0.50587940733714198416,Cyanobacteria-
Synechococcus_sp._JA-2-3Ba2-
13_86609094:0.18812354076065163078)54:0.10764327985079905070)52:0.04158375519825259492)70:0.05572041112191995077,Cyanobacteria-
Lyngbya_sp._PCC_8106_119486469:0.18450532910954361987)95:0.07229941572680512185,Cyanobacteria-
Synechocystis_sp._PCC_6803_16331567:0.21219157691640291863)100:0.10740116367117155671,Cyanobacteria-
Crocosphaera_watsonii_WH_8501_67924974:0.06683644606227977236);

Plantae-Chlamydomonas_reinhardtii_139405

((Bacteria-Saccharopolyspora_erythraea_134101758:0.37673833554219932074,(Bacteria-
Thermobifida_fusca_72162442:0.29343200148379378067,(((Bacteria-
Roseiflexus_sp._148656197:0.41196510397812818871,(Bacteria-
Candidatus_Desulfococcus_121541644:0.74898747062233062000,Bacteria-
Anaeromyxobacter_dehalogenans_86157471:0.58941096489265643044)28:0.05632323142631570634)48:0.09884213763850956125,(((((Bacteria-Pedobacter_sp._149275976:0.34161856339362273172,Bacteria-
Cytophaga_hutchinsonii_110637940:0.28351988297825903018)27:0.03610178416519307931,Bacteria-
Microscilla_marina_124002514:0.38187732479145453102)22:0.04740676001211944351,((Bacteria-
Desulfuromonas_acetoxidans_95929912:0.18324950844089263335,(Bacteria-
Bacteroides_caccae_153807144:0.13866791283576704319,Bacteria-
Parabacteroides_distasonis_150009644:0.11825387634302750306)97:0.08064301727138974141)95:0.08984739636825415932,

((Bacteria-Flavobacterium_johnsoniae_146298313:0.15552941091020192066,Bacteria-
Flavobacteria_bacterium_89889440:0.30721891551689284316)100:0.12144344341684246147,(Bacteria-
unidentified_eubacterium_149371964:0.16449430441522380764,Bacteria-
Gramella_forsetii_120434482:0.16918290896667670387)90:0.05774047669220284951)92:0.07474480903684375954)64:0.045
24582266612631881)25:0.04981587034372766259,Bacteria-
Algoriphagus_sp._126645189:0.21570245953804395578)100:0.31513764490592166556,((Chromalveolata-
Phytophthora_sojae_124508:0.58360473857182393331,(Excavata-Naegleria_gruberi_75743-
fgeneshNG_pg.scaffold_84000052:0.61610043258329438842,(Opisthokonta-
Apis_mellifera_66529862:0.53642056448404329405,Opisthokonta-
Drosophila_melanogaster_20130383:0.31593210172346808395)100:0.41729975106802280616)92:0.14646083235511353493)9
3:0.12777406466202098634,(((Chlamydia-Chlamydia_muridarum_15834952:0.07900959204166853322,(Chlamydia-
Chlamydia_trachomatis_15604781:0.00000121823947223981,Chlamydia-
Chlamydia_trachomatis_76788775:0.00245763674943383879)100:0.06294776464235349311)100:0.18379629161599339571,((
((Chlamydia-Chlamydophila_pneumoniae_33241703:0.00000121823947223981,Chlamydia-
Chlamydophila_pneumoniae_16752679:0.00000121823947223981)54:0.00000121823947223981,Chlamydia-
Chlamydophila_pneumoniae_15618276:0.00247534638026705896)59:0.00000121823947223981,Chlamydia-
Chlamydophila_pneumoniae_15835894:0.00247915831819011919)100:0.26632264762956869841,(Chlamydia-
Chlamydophila_abortus_62185046:0.06392626358809498643,(Chlamydia-
Chlamydophila_felis_89898384:0.09116440202215184141,Chlamydia-
Chlamydophila_caviae_29840193:0.07645104664389383575)68:0.03683029345224593520)99:0.13151733368357845788)91:0.
12214419015883486164)100:0.65394635780442844641,(Chlamydia-
Candidatus_Protochlamydia_46446803:0.37391748802002988672,(((Plantae-
Chlamydomonas_reinhardtii_139405:0.07571051401325484820,Plantae-
Volvox_carteri_83275_jgi:0.06625724818795990234)100:0.22159457657125741781,((Plantae-
Ostreococcus_lucimarinus_36202:0.36274282247423245140,Plantae-
Ostreococcus_tauri_15385:0.38266792558155909854)75:0.10279851448540978931,((Plantae-
Oryza_sativa_115436768:0.18595602604582367468,Plantae-
Arabidopsis_thaliana_15232946:0.20800849256209519300)97:0.10829535332297797467,Plantae-
Physcomitrella_patens_62167:0.22506668285492004156)99:0.13308055595481135813)48:0.04737390342660465631)99:0.124
65171412370484871,Plantae-
Cyanidioschyzon_merolae_CMQ275C:0.42445701878243352612)89:0.07867285237234430961)100:0.17757232206271941766
)90:0.10953718386196957535)87:0.08307993333544186343)22:0.05230780585672085981)90:0.10108371562795821774,((((B
acteria-Geobacillus_thermodenitrificans_138896346:0.19489921102264093355,Bacteria-
Bacillus_cereus_152977028:0.17387772779174559545)54:0.04695238718209549661,((Bacteria-
Lactococcus_lactis_125623269:0.16048610858067158258,Bacteria-
Streptococcus_pyogenes_15674319:0.15380067189661861260)100:0.38535978323257452649,(((Bacteria-
Lactobacillus_gasseri_116628855:0.20381073846091657398,Bacteria-
Leuconostoc_mesenteroides_116619116:0.42252876977223030597)100:0.15863567054327554207,Bacteria-
Listeria_welshimeri_116873028:0.24518626830270809358)100:0.08388294827292092082,Bacteria-
Staphylococcus_saprophyticus_73662343:0.36261461295859953591)97:0.07366322550918499856)55:0.03333804137200781920)34:0.06173163726216262387,Bacteria-
Desulfitobacterium_hafniense_109647650:0.40259254568064856139)64:0.07514289781448102445,((Bacteria-
Psychromonas_sp._90407450:0.21382375800481112638,((Bacteria-
marine_gamma_119502753:0.32517630087011251128,(Bacteria-
Oceanospirillum_sp._89095105:0.16891408553095080269,Bacteria-
Oceanobacter_sp._94502122:0.17816903838105624391)51:0.02699936071836281024)49:0.03548703723923325726,Bacteria-
Marinomonas_sp._152995102:0.25752998560574041909)94:0.10789865906003974494)100:0.17160090770394212378,((((Bact
eria-Sodalis_glossinidius_85059426:0.12212224548517520983,(Bacteria-
Yersinia_pseudotuberculosis_153948792:0.03887763127984072975,Bacteria-
Serratia_proteamaculans_157370459:0.07103224279401640362)73:0.01967791330129379720)85:0.03068061685390450002,(((
Bacteria-Shigella_sonnei_74312035:0.00000121823947223981,Bacteria-
Escherichia_coli_15802051:0.00000121823947223981)100:0.02840761241829359643,Bacteria-
Salmonella_enterica_16760464:0.00700321016219394334)67:0.01413741623180944477,((Bacteria-
Klebsiella_pneumoniae_152970527:0.01204356056366379102,Bacteria-
Citrobacter_koseri_157145898:0.01349684696850844516)36:0.00546383457302799819,Bacteria-
Enterobacter_sakazakii_156934169:0.03626465502296627452)38:0.00841100610423431774)83:0.04236593775324004607)100
:0.11519916497790358423,(Bacteria-Vibrio_angustum_90577965:0.00111459564801568334,Bacteria-
Photobacterium_sp._89076033:0.02497914225496796273)100:0.20804580702812414117)99:0.11166955493225012852,((Bacte
ria-Chromobacterium_violaceum_34498527:0.16799908378433328626,Bacteria-
Dechloromonas_aromatica_71908788:0.15956224837431706720)82:0.09467069698615879214,Bacteria-
Neisseria_meningitidis_15677671:0.25068433988589783334)49:0.04419399145402747725)51:0.06094691309737498147)100:
0.27326615286404420946)85:0.06568061873839217690)100:0.19976901566311144887)68:0.05910002487070996346)84:0.06

753955671189229570,(((Bacteria-Clavibacter_michiganensis_148273171:0.25980771557431414642,Bacteria-
Arthrobacter_aurescens_119960707:0.17173807386202008529)98:0.16094430761618089276,(Bacteria-
Actinomyces_odontolyticus_154509115:0.30348026828647839404,Bacteria-
Bifidobacterium_adolescentis_154488639:0.50588681445364513944)74:0.09003141643948722739)74:0.080665026514263160
62,Bacteria-Streptomyces_avermitilis_29833001:0.16956219438793604382)87:0.08481710853126794847,Bacteria-
Nocardioides_sp._119716211:0.33018995341914536557);

Plantae-Chlamydomonas_reinhardtii_155606

(Bacteria-Ralstonia_eutropha_73542207:0.12002709172980793872,(((Bacteria-
Rhodoferax_ferrireducens_89900860:0.11689334269063432481,Bacteria-
Polaromonas_naphthalenivorans_121604633:0.11200987903843655225)100:0.16782649671817773118,Bacteria-
Polynucleobacter_sp._145588803:0.15645032067118255781)63:0.05889323789995537517,((((Bacteria-
Nitratiruptor_sp._152990309:0.57169009804870274394,(((Bacteria-
Dorea_longicatena_153853240:0.09021290018809877131,Bacteria-
Ruminococcus_obeum_153812754:0.17475966259795480684)100:0.23046153099308319678,Bacteria-
Clostridium_difficile_126698375:0.38986153904307635498)80:0.09660917020010532930,((((Chromalveolata-
Aureococcus_anophagefferens_14447:0.61205163933036776935,(Chromalveolata-
Thalassiosira_pseudonana_34698:0.20277436580712665526,Chromalveolata-
Phaeodactylum_tricornutum_13791:0.15489989821730881792)100:0.35131866123348098885)92:0.23015188355539351051,(P
lantae-Cyanidioschyzon_merolae_CMQ012C:1.21253683804141809688,((Plantae-
Volvox_carteri_120889_jgi:0.16207150121947361265,Plantae-
Chlamydomonas_reinhardtii_155606:0.02642021793998287210)100:0.45486808551719676297,((Plantae-
Physcomitrella_patens_74110:0.24369479138092864368,(Plantae-Oryza_sativa_115464935:0.25749540799366049670,Plantae-
Arabidopsis_thaliana_18400953:0.10502002140538994890)100:0.42774453861230216090)98:0.24962691153114069387,(Plant
ae-Ostreococcus_tauri_32784:0.24670619197491508445,Plantae-
Ostreococcus_lucimarinus_31615:0.06951524047271036288)100:0.42413018123195811437)33:0.05577035551997758245)70:0
.18803664605945025201)55:0.09102573976162051106)81:0.17402749558897012694,(Chlamydia-
Candidatus_Protochlamydia_46446962:0.28933712441839404272,((Chlamydia-
Chlamydophila_pneumoniae_33241926:0.00000121823947223981,((Chlamydia-
Chlamydophila_pneumoniae_15618484:0.00000121823947223981,Chlamydia-
Chlamydophila_pneumoniae_15836104:0.00000121823947223981)28:0.00000121823947223981,Chlamydia-
Chlamydophila_pneumoniae_16752465:0.00000121823947223981)19:0.00000121823947223981)99:0.06943985166831251332
,((Chlamydia-Chlamydia_muridarum_15835357:0.01544973360270749350,(Chlamydia-
Chlamydia_trachomatis_15605184:0.00855745650215312612,Chlamydia-
Chlamydia_trachomatis_76789193:0.00000121823947223981)98:0.01057927530901202513)100:0.08919909898033856477,((C
hlamydia-Chlamydophila_caviae_29839936:0.03419371535137689933,Chlamydia-
Chlamydophila_abortus_62184810:0.03954097846598216004)45:0.01628079887864936418,Chlamydia-
Chlamydophila_felis_89898645:0.05179450452983178771)69:0.04143122164786104678)87:0.09639737163463399372)98:0.32
774060304922508768)92:0.14487622958862142464)90:0.32599586241951750143,(((Bacteria-
Roseovarius_sp._114762980:0.05269170319049649998,((Bacteria-
Dinoroseobacter_shibae_118734753:0.03534498076352769053,(Bacteria-
Rhodobacterales_bacterium_126727914:0.09580548614221770509,((Bacteria-
alpha_proteobacterium_114771672:0.20514942023210219757,Bacteria-
Jannaschia_sp._89055871:0.09299735597403335774)52:0.03460538248615783324,Bacteria-
Oceanicola_granulosus_89071234:0.07245453636964062361)12:0.01835312479321055470)29:0.03702518351631740173)60:0.
04933783067567026198,Bacteria-
Roseobacter_sp._149914396:0.07064247113291546865)51:0.04348509632945464498)100:0.15057970210425425384,((((Bacte
ria-Nitrobacter_hamburgensis_92118985:0.08920028293750946469,(Bacteria-
Bradyrhizobium_japonicum_27376645:0.06334888746984651542,Bacteria-
Rhodopseudomonas_palustris_115526759:0.08326321131727680569)52:0.01788981773168135322)100:0.12795228916599746
261,(Bacteria-Methylobacterium_sp._149124304:0.18532062900096912816,Bacteria-
Xanthobacter_autotrophicus_154246991:0.10234006669440925963)77:0.05363428099197331544)56:0.0448659493935252770
6,Bacteria-Parvibaculum_lavamentivorans_154252561:0.24737915396088877573)97:0.14729358099345840616,(((Bacteria-
Ochrobactrum_anthropi_153008531:0.08905077337884818867,Bacteria-
Bartonella_bacilliformis_121602842:0.15134999060343273802)28:0.02123940771371084571,Bacteria-
Mesorhizobium_sp._110635521:0.07339706320165960418)47:0.02734950698152237292,(Bacteria-
Aurantimonas_sp._90418962:0.15014648353444121076,(Bacteria-
Sinorhizobium_medicae_150397839:0.03810989144907962756,(Bacteria-
Agrobacterium_tumefaciens_15891221:0.06998633509954672871,Bacteria-
Rhizobium_leguminosarum_116253722:0.08589561886863739115)38:0.01981159096945931189)73:0.06048071397034363028
)42:0.01990886189899582215)94:0.09134040698155190818)84:0.08537884473065641966)100:0.31543697082441068957,((Ba
cteria-Bacillus_subtilis_16079834:0.27407910869862117664,Bacteria-

Geobacillus_thermodenitrificans_138896161:0.20000395993709960130)85:0.12311986112632597457,((Bacteria-
Leuconostoc_mesenteroides_116618556:0.17733433115377611466,Bacteria-
Oenococcus_oeni_116490816:0.12064690698052762952)100:0.19185410411072409409,Bacteria-
Lactobacillus_acidophilus_58337048:0.29420598866684205541)97:0.17905004469117247190)66:0.09644661126907344562)1
3:0.02122383543296823064)10:0.04452979429572453962)44:0.06622468713777901472)18:0.04980963049883913724,(Bacter
ia-delta_proteobacterium_94264072:0.39206797458310199689,(Bacteria-
Geobacter_bemidjiensis_145620538:0.33691282383933451872,((Bacteria-
Myxococcus_xanthus_108761477:0.27547811375397673883,Bacteria-
Anaeromyxobacter_sp._153003533:0.26026464052918629744)100:0.31187841090320156390,((Bacteria-
Petrotoga_mobilis_145622827:0.23708515584437933610,(Bacteria-
Thermosipho_melanesiensis_150020877:0.10633745417023938995,Bacteria-
Fervidobacterium_nodosum_154249785:0.14309001574593685491)100:0.18545640564121132488)100:0.18403052788462526
124,(((Bacteria-Frankia_sp._86740074:0.25372504293521397356,Bacteria-
Acidothermus_cellulolyticus_117928553:0.15649071162385533307)60:0.07313462820760281702,(Bacteria-
Salinispora_arenicola_119881213:0.12609585577189488803,(Bacteria-
Mycobacterium_smegmatis_118467795:0.14130291380663614897,Bacteria-
Thermobifida_fusca_72162495:0.09562943288794556462)39:0.03085312665313836786)25:0.01035169940904161548)35:0.04
088232348755105328,Bacteria-
Leifsonia_xyli_50954745:0.23331118232294623116)99:0.44288311357547721636)37:0.06595040357007352083)22:0.0486931
7247929583203)12:0.04229280978763352156)10:0.02526998987301152036)65:0.06706350520444957775,Bacteria-
Magnetococcus_sp._117923793:0.31220555690723167386)64:0.04431183567145597496,(Bacteria-
Methylococcus_capsulatus_53804482:0.27249000372040682594,Bacteria-
Pseudomonas_putida_119859094:0.26844028199169384097)77:0.08060871179104714401)100:0.18909992716924919898)46:0
.03825472659003174847,Bacteria-Bordetella_bronchiseptica_33600856:0.22312992907458584213);

Plantae-Chlamydomonas_reinhardtii_196649
(((((Bacteria-Algoriphagus_sp._126646068:0.15575604660265945922,(Bacteria-
Flavobacterium_psychrophilum_150024270:0.14796761571776068100,(Bacteria-
Gramella_forsetii_120437698:0.09574000104277471945,Bacteria-
Psychroflexus_torquis_91215426:0.13280915447875490942)97:0.07020617432347621423)94:0.10021655536697728073)63:0.
05296117993942617286,(Bacteria-Cytophaga_hutchinsonii_110636534:0.19909470170729670624,(Bacteria-
Flavobacteria_bacterium_126662278:0.33018079713062153946,Bacteria-
Microscilla_marina_124008182:0.13911099243720165264)74:0.06984458628979485495)29:0.02409252045202556897)23:0.02
511397875388277079,(((Bacteria-Symbiobacterium_thermophilum_51892185:0.19098186152271809379,((((Bacteria-
Actinobacillus_pleuropneumoniae_126207739:0.20361478919077966032,((Bacteria-
Serratia_proteamaculans_157368330:0.08689535653442635732,Bacteria-
Enterobacter_sakazakii_156935949:0.01699264189751282031)68:0.02028855493542333979,Bacteria-
Sodalis_glossinidius_85057995:0.09987009083466447112)72:0.01627529362924208869)100:0.12298292589452806955,Bacter
ia-Deinococcus_geothermalis_94984937:0.12751111945786594570)94:0.07132054123216727270,(((Bacteria-
Chromohalobacter_salexigens_92115375:0.19601754642317395505,(Bacteria-
Stenotrophomonas_maltophilia_119876252:0.11286803472829394501,Bacteria-
Pseudomonas_putida_126357451:0.12890991294770323683)38:0.03916065536348765919)43:0.03588274643190552532,(Bact
eria-Bordetella_bronchiseptica_33599841:0.21654244364303890102,Bacteria-
Delftia_acidovorans_118732644:0.14945603357625278362)20:0.04229829942675721738)37:0.03625386086256694729,Bacteri
a-
Aeromonas_hydrophila_117621382:0.15996267716272433157)96:0.08076122264139121154)81:0.05449699976967482123,((E
xcavata-Naegleria_gruberi_69926-fgeneshNG_pg.scaffold_37000082:0.79200530515320299862,Bacteria-
Lactococcus_lactis_116511268:0.30957329149564355442)5:0.02177437859093940206,(((Chlamydia-
Chlamydia_trachomatis_76789016:0.00000121823947223981,Chlamydia-
Chlamydia_trachomatis_15605015:0.00000121823947223981)100:0.53075244196666493401,(Chromalveolata-
Phytophthora_sojae_109207:0.22107618959944419501,(Chromalveolata-
Aureococcus_anophagefferens_59085:0.33167926729989805068,Plantae-
Galdieria_sulphuraria_HET_2D8_1:0.33057111784468867777)86:0.13167834705424180664)65:0.08074832964420923520)100
:0.30331490417505663215,(Bacteria-Borrelia_burgdorferi_15594498:0.65128658957394025553,Bacteria-
Thermus_thermophilus_46198497:0.18361527785099310273)63:0.13666837890834079849)14:0.03469724902483240642)4:0.0
5924328743506868805)5:0.03405343516668986326)3:0.05138251999150016819,((((Chromalveolata-
Aureococcus_anophagefferens_6356:0.51254697646805280620,((Chromalveolata-
Isochrysis_galbana_Contig1364_3:0.10618233816330516217,(Chromalveolata-
Karenia_brevis_Kb_AUAO_R_45G22_4:0.05071757569150456973,Chromalveolata-
Karenia_brevis_Contig4287_5:0.20135304123669414689)88:0.06562077290453725842)100:0.21048615229555833750,Chrom
alveolata-
Pavlova_lutheri_PLE00008434_5:0.19521548631102719762)35:0.04677477770140254371)21:0.09107810395660648206,((Bac

teria-Chloroflexus_aggregans_118047452:0.17942406036972377570,((Plantae-
Cyanidioschyzon_merolae_CMT028C:0.68887522778399135071,Bacteria-
Clostridium_difficile_126699235:0.22936225125871836927)16:0.09999539556051023625,Bacteria-
Lactobacillus_sakei_81428506:0.35828893554437135638)17:0.12038799518188057769)5:0.03334345419174688380,(((Plantae
-Porphyra_yezoensis_Contig2003_2:0.24829382072036640383,(Plantae-
Galdieria_sulphuraria_Contig577_1:0.16205831852840624219,Plantae-
Cyanidioschyzon_merolae_CMN023C:0.15561498665705986166)83:0.06504241320228314194)56:0.04690222534648424979,(
Plantae-Chlamydomonas_reinhardtii_53941:0.05815831289560240841,Plantae-
Volvox_carteri_108150_jgi:0.06831349159069179111)98:0.17853061115744320309)52:0.03819163794488018154,((Chromalv
eolata-Phaeodactylum_tricornutum_12583:0.08520030662823217804,Chromalveolata-
Thalassiosira_pseudonana_32874:0.03432356119821358686)100:0.44049604233391814745,(((Plantae-
Chlamydomonas_reinhardtii_196647:0.00000121823947223981,Plantae-
Chlamydomonas_reinhardtii_196649:0.00000121823947223981)100:0.29786325801819196757,Plantae-
Volvox_carteri_72831_jgi:0.35772469027329006908)100:0.48049772008803154355,(Plantae-
Chlamydomonas_reinhardtii_115390:0.29723820788339061494,(Plantae-
Volvox_carteri_91989_jgi:0.36076035064062889068,Plantae-
Chlamydomonas_reinhardtii_114882:0.00501517444038986331)92:0.32730391485454263245)97:0.34834931390833667741)5
0:0.08690916885712866857)8:0.04829525022152209718)18:0.09142517440345258872)1:0.01379917342989368974)2:0.01853
942519111505260,Bacteria-Salinibacter_ruber_83816288:0.41805360138541336479)1:0.01735246099070226339,((Bacteria-
Herpetosiphon_aurantiacus_113942066:0.45965311855197737989,Chromalveolata-
Emiliania_huxleyi_Contig23_6:0.75457790172312921850)4:0.02478074325008397527,(Bacteria-
Roseiflexus_sp._148657343:0.21489060915260474460,(Bacteria-
Acidobacteria_bacterium_94970720:0.19010555133902998226,((Cyanobacteria-
Trichodesmium_erythraeum_IMS101_113477925:0.34214056695028710608,Cyanobacteria-
Nostoc_punctiforme_PCC_73102_23126478:0.13409593924081308902)39:0.05487547166265345500,((Cyanobacteria-
Crocosphaera_watsonii_WH_8501_67920506:0.34453517342581357896,Bacteria-
Rhodopseudomonas_palustris_115522220:0.44302678335520517816)12:0.03298749944323655509,(Cyanobacteria-
Gloeobacter_violaceus_PCC_7421_37520251:0.18609206109757744918,((Cyanobacteria-
Anabaena_variabilis_ATCC_29413_75907667:0.02580554924236232867,((Cyanobacteria-
Nostoc_punctiforme_PCC_73102_53688908:0.09500906666368788556,Cyanobacteria-
Nodularia_spumigena_CCY9414_119510926:0.09383341101107575688)98:0.09877985614074462350,Cyanobacteria-
Nostoc_sp._PCC_7120_17227566:0.00000121823947223981)24:0.00311366950611134327)97:0.13805665279842593840,Cyan
obacteria-Thermosynechococcus_elongatus_BP-
1_22297580:0.44454757391206756179)14:0.05136948959634868306)11:0.04753614316817034863)3:0.02578294552960375130)9:0.04256043892658664674)17:0.06328966452008680244)6:0.02596392694171790333)2:0.04685169510057847486)3:0.03
23329421942497238 8)28:0.06565425948743766615,(((Excavata-
Jakoba_bahamiensis_109793011_2:0.33742110228461907884,Excavata-
Malawimonas_californiana_Contig63_3:0.40389842487582855446)100:0.20449445574300584294,(((Bacteria-
Methylibium_petroleiphilum_124267667:0.04719757332269441247,(Bacteria-
Dechloromonas_aromatica_71908825:0.14342864381362760429,(Bacteria-
Acidovorax_avenae_120611237:0.01460309994602879829,Bacteria-
Verminephrobacter_eiseniae_121611788:0.06540001026227849901)99:0.08205356340401029347)45:0.03569834539568596948)100:0.22042096387593534956,Bacteria-
Shewanella_loihica_127512762:0.15897682672168791940)85:0.09662480106639011190,((Bacteria-
Roseobacter_denitrificans_110679070:0.12182431380799403631,Bacteria-
alpha_proteobacterium_114771855:0.12220122915156426957)100:0.38615842900488833722,(Plantae-
Arabidopsis_thaliana_15241373:0.33208615761826504320,Plantae-
Volvox_carteri_109532_jgi:0.20734160926765388133)98:0.16126741858519472173)47:0.08889110191227519997)63:0.06482
500369804222728)100:0.23437232213936223801,(Bacteria-
Dichelobacter_nodosus_146328771:0.36117248151729558492,((Bacteria-
Vibrio_vulnificus_27364653:0.10647237262489958609,Bacteria-
Vibrionales_bacterium_148977227:0.13540179474461280784)95:0.09854990151478337790,Bacteria-
Photobacterium_sp._89073346:0.31139365224539328203)82:0.04995645977048478875)99:0.16648153555222330868)26:0.04
090497253298213537)1:0.02485245288796085819)7:0.05014159309643249907,(((Bacteria-
Pedobacter_sp._149277715:0.33681518758920270251,(Bacteria-
Xanthomonas_oryzae_58582451:0.22309296628265667950,(Bacteria-
Xylella_fastidiosa_28198774:0.18670863306578891483,(Cyanobacteria-
Synechococcus_sp._RS9917_87124639:0.31420074740088499432,Cyanobacteria-
Synechococcus_sp._WH_5701_87301174:0.35089662558423495575)14:0.05840121199526431689)12:0.03953913444710036723)20:0.04313594154049504165)4:0.02982855714536089553,(Bacteria-
Wolinella_succinogenes_34558145:0.16599335960281746960,Bacteria-
Caulobacter_crescentus_16126021:0.29372113657618037497)49:0.08485746198360256953)13:0.07873305995461761519,Bact

eria-
Hyphomonas_neptunium_114798697:0.15146090126771336037)40:0.13344198117771918777)70:0.05879896406264115832,(
Bacteria-Alkalilimnicola_ehrlichei_114320262:0.18413603381655768709,Bacteria-
Halorhodospira_halophila_121998934:0.14914624673342477545)97:0.09575217498134919181,Bacteria-
Thiomicrospira_crunogena_78484582:0.19304202848175625040);

Plantae-Chlamydomonas_reinhardtii_503
((Bacteria-Halothermothrix_orenii_89210654:0.34831690857568275987,(((Bacteria-
Thermosinus_carboxydivorans_121534014:0.40117679951692586382,Bacteria-
Caldicellulosiruptor_saccharolyticus_146295820:0.40496183295933019242)65:0.08320177093086948694,(Bacteria-
Desulfotomaculum_reducens_134299316:0.36946119919657954833,Bacteria-
Bacillus_cereus_42784071:0.44297918359022470103)25:0.05481261198587542016)35:0.05958697188658354515,(((Bacteria-
Bacteroides_capillosus_154500435:0.67143902285021661580,(Bacteria-
Ruminococcus_obeum_153810465:0.13355459251350737526,(Bacteria-
Eubacterium_ventriosum_154483643:0.15861422353133533547,(Bacteria-
Dorea_longicatena_153853052:0.11287727783627912737,Bacteria-
Clostridium_phytofermentans_106885561:0.26591728759846827712)37:0.03761827343996564793)47:0.041281734615911717
94)100:0.23593848542804274349)87:0.17235317879856074108,(((Chlamydia-
Candidatus_Protochlamydia_46447230:0.64163089758488056624,(Plantae-
Ostreococcus_lucimarinus_48511:0.45416846067857735703,Plantae-
Chlamydomonas_reinhardtii_185710:0.31948132717157706750)100:0.44104958845280356483)100:0.60901219663262040616,
((((Bacteria-Roseiflexus_castenholzii_156742747:0.55634449853890632909,Bacteria-
Psychromonas_ingrahamii_119946006:0.72958680789435703673)76:0.17355621378032592972,(Bacteria-
Solibacter_usitatus_116619448:0.48938523488721064059,Bacteria-
Acidobacteria_bacterium_94970376:0.50498392493820754012)75:0.11844797950908288187)33:0.06151990922079473428,(((
Bacteria-Stigmatella_aurantiaca_115374944:0.16850586602246625523,Bacteria-
Myxococcus_xanthus_108760408:0.18734490355256042116)100:0.39305807652080626546,((((Bacteria-
Desulfuromonas_acetoxidans_95930377:0.37774004536226707396,Bacteria-
Thiomicrospira_crunogena_78484857:0.37251505162434755958)100:0.29526353956430123615,Bacteria-
Nitrosococcus_oceani_77164289:0.49719277315823406926)73:0.09402041565859942107,((Bacteria-
Halorhodospira_halophila_121997895:0.26385425299998610527,Bacteria-
Alkalilimnicola_ehrlichei_114320117:0.21147155245912033217)100:0.38623684944367175920,Bacteria-
Methylococcus_capsulatus_53804456:0.42361059410706586315)25:0.03674047031690754611)39:0.07883187315811837625,((
Bacteria-Beggiatoa_sp._153876592:0.53071780762713194335,((Bacteria-
Nitrosomonas_eutropha_114330623:0.43531828172770181684,Bacteria-
Methylobacillus_flagellatus_91775825:0.43324459012114263645)75:0.12531777192590037862,Bacteria-
Azoarcus_sp._56479379:0.49931460544841593796)65:0.06209609190975688697)51:0.08464022631503050065,((((Bacteria-
Burkholderia_xenovorans_91778822:0.36353382030390163360,Bacteria-
Ralstonia_solanacearum_17548463:0.42976262400181403089)100:0.38154288306350253590,((((Bacteria-
Fulvimarina_pelagi_114705952:0.58419684480533828896,(Bacteria-
Rhizobium_leguminosarum_116253854:0.16590599203388867178,Bacteria-
Agrobacterium_tumefaciens_15890897:0.18147608849769156225)100:0.22896202286639288914)91:0.1229454362036675602
0,(Bacteria-Sagittula_stellata_126728011:0.37807629406959081253,Bacteria-
Roseobacter_denitrificans_110680078:0.26229175089836104240)100:0.18717116968338190497)55:0.11127499978029847805,
(Bacteria-Bradyrhizobium_japonicum_27381570:0.36883575641639998466,(Bacteria-
Rhodopseudomonas_palustris_39933459:0.26645933425659340532,Bacteria-
Acidiphilium_cryptum_148259152:0.47765751678017809301)51:0.05747762118453695473)90:0.13770219644692283101)99:
0.18677071342496687212,(Bacteria-Polaromonas_naphthalenivorans_121604016:0.44542700544353314163,(Bacteria-
Yersinia_enterocolitica_123444184:0.35033819270499805265,Bacteria-
Actinobacillus_pleuropneumoniae_126207833:0.49628545905646376246)100:0.24260626585384378884)88:0.1201102846550
8594154)29:0.04861882685115313102)17:0.03030663131039865177,Bacteria-
Rhodospirillum_rubrum_83593580:0.62120960361143962025)7:0.04240154278635720198,(Bacteria-
Xanthomonas_campestris_78046007:1.01313158075658726887,Bacteria-
Magnetospirillum_magneticum_83311210:0.47799940565590226527)10:0.09042783462690998086)22:0.053109795540230528
29)47:0.08546726922689563966)96:0.22399479218015108661)18:0.08313668235555285446,(Bacteria-
Anaeromyxobacter_sp._153003016:0.39908287529603664323,(((Plantae-
Chlamydomonas_reinhardtii_112896:1.08085587099091262608,(((Plantae-
Volvox_carteri_72820_jgi:0.03624867844632156944,Plantae-
Chlamydomonas_reinhardtii_503:0.04706311164605241765)100:0.22987152508862587763,(Plantae-
Ostreococcus_tauri_28387:0.08137131838220253155,Plantae-
Ostreococcus_lucimarinus_49880:0.01967319645392744815)100:0.27028779118670975823)100:0.13304528581812136157,((P
lantae-Arabidopsis_thaliana_15223331:0.16489529294081650690,(Plantae-

Oryza_sativa_115466210:0.20729319701987394819,Plantae-
Oryza_sativa_115471703:0.22037075344039233626)49:0.03632799992236265818)85:0.06528043415176397468,(Plantae-
Physcomitrella_patens_120571:0.04717653408134966225,Plantae-
Physcomitrella_patens_70198:0.06783649822973052135)100:0.19328586377719966793)100:0.16720718802992284857)61:0.0
5610414331069329269)52:0.05760834790203648442,(Cyanobacteria-
Crocosphaera_watsonii_WH_8501_67924488:0.48507824751678352282,Chromalveolata-
Guillardia_theta_57335306:0.77343471280454301198)55:0.07057284022913362653)100:0.36431511544157213489,(((Plantae-
Ostreococcus_lucimarinus_2524:0.25973671645088963755,Plantae-
Ostreococcus_tauri_27007:0.06766158104560743292)100:0.24366149453186816687,(((Plantae-
Oryza_sativa_115482170:0.16237091727226110605,(Plantae-
Arabidopsis_thaliana_15232051:0.11230420444202507402,(Plantae-
Oryza_sativa_115467234:0.11629914529782223176,Plantae-
Oryza_sativa_115448649:0.10138025528326408131)99:0.09564631359563958990)39:0.04136551683118586814)100:0.102605
24713028251753,(Plantae-Physcomitrella_patens_202950:0.04176670782835284479,(Plantae-
Physcomitrella_patens_177976:0.06102117475509648281,Plantae-
Physcomitrella_patens_222462:0.03628309612438367082)35:0.01090984171310534386)100:0.17490018850323213462)82:0.0
9477153823851139358,((Plantae-Chlamydomonas_reinhardtii_162226:0.00000121823947223981,Plantae-
Chlamydomonas_reinhardtii_109372:0.00000121823947223981)100:0.06969980708597257879,Plantae-
Volvox_carteri_103172_jgi:0.03863044480039982270)100:0.22097949418781742681)37:0.07109663420469217177)100:0.281
18849863363448716,(Plantae-Chlamydomonas_reinhardtii_106158:0.53143453809817653433,((Plantae-
Ostreococcus_tauri_8953:0.05694794929934261785,Plantae-
Ostreococcus_lucimarinus_41777:0.06626489655744181861)100:0.25112201770639619447,((Plantae-
Arabidopsis_thaliana_15237934:0.16912118603432754749,Plantae-
Oryza_sativa_115466564:0.16577028932944273820)98:0.11344937734677418284,(Plantae-
Physcomitrella_patens_200110:0.00000121823947223981,Plantae-
Physcomitrella_patens_200113:0.00000121823947223981)100:0.19711691098678693623)89:0.10175670448823818937)100:0.
12704132484587804530)88:0.12005443729089129712)100:0.13825417506694906433)100:0.40747495184870147211)33:0.089
55141179287880171)14:0.06997710302914640634)29:0.08320798662868557349,Bacteria-
Desulfovibrio_vulgaris_46580649:0.92089981623897432250)35:0.05461137703869503590)44:0.06516903381400621087,(Cya
nobacteria-Crocosphaera_watsonii_WH_8501_67925328:0.33921337875287882468,((Cyanobacteria-
Prochlorococcus_marinus_str._MIT_9211_84517893:0.14946652329608453580,(Cyanobacteria-
Synechococcus_sp._RS9916_116074565:0.05036889282309751220,(Cyanobacteria-
Synechococcus_sp._WH_7805_88808387:0.02219486633158112426,Cyanobacteria-
Synechococcus_sp._WH_7803_148239362:0.01860677076255752571)99:0.03830488591611434790)97:0.03992244576256100
130)100:0.23548210033887320702,Cyanobacteria-
Synechococcus_elongatus_PCC_7942_81301327:0.16891485040866585643)89:0.13701824623320865704)100:0.54059361907
757630394)89:0.21267638156179172193)8:0.04411372052441931563,(Bacteria-
Collinsella_aerofaciens_139436881:0.51226521347251330329,(Bacteria-
Lactococcus_lactis_15672681:0.47855039828467332841,Bacteria-
Lactobacillus_plantarum_28376995:0.52431905018944791053)100:0.15613709255962371358)53:0.07927024337980775881)1
3:0.07743520890489888342)50:0.12804921710306893523)100:0.43289618558426212758,(Bacteria-
Thermosipho_melanesiensis_150019985:0.22127714254375036029,Bacteria-
Fervidobacterium_nodosum_154249003:0.25854061474961687050)94:0.10359575191297025476,Bacteria-
Thermotoga_petrophila_148269177:0.18741728540471236375);

Plantae-Cyanidioschyzon_merolae_CMO211C
(((Bacteria-Alkaliphilus_metalliredigens_150392425:0.16011133435855798290,((((Bacteria-
Desulfitobacterium_hafniense_109647925:0.34238547066474583902,(Bacteria-
Caldicellulosiruptor_saccharolyticus_146296542:0.44220540040780770275,((((Cyanobacteria-
Synechococcus_sp._RCC307_148241898:0.26294766638714772711,(((Cyanobacteria-
Prochlorococcus_marinus_str._NATL1A_124025652:0.00348741284277507028,Cyanobacteria-
Prochlorococcus_marinus_str._NATL2A_72382116:0.01366284719298549270)100:0.41768846951051380456,(Cyanobacteria-
Prochlorococcus_marinus_str._MIT_9211_84518235:0.15587949855640192975,(((Cyanobacteria-
Prochlorococcus_marinus_str._AS9601_123968458:0.02159676893156325245,Cyanobacteria-
Prochlorococcus_marinus_str._MIT_9301_126696261:0.00000121823947223981)64:0.02551421479444889936,Cyanobacteria-
Prochlorococcus_marinus_str._MIT_9312_78779248:0.00000121823947223981)100:0.77408127580655283762,Cyanobacteria-
Prochlorococcus_marinus_subsp._marinus_str._CCMP1375_33240211:0.13861440678646597124)26:0.176370328911372831
9)18:0.12293345629326032920)32:0.09829543583279055918,((Cyanobacteria-
Synechococcus_sp._RS9916_116074639:0.12953512990610518973,(Cyanobacteria-
Synechococcus_sp._CC9311_113954920:0.11253352815373739160,(((Cyanobacteria-
Synechococcus_sp._BL107_116070734:0.14011981356333574888,Cyanobacteria-
Synechococcus_sp._CC9605_78212714:0.04646914808815328507)30:0.01402098769486279442,(Cyanobacteria-

Synechococcus_sp._WH_8102_33865582:0.14232414310729971096,Cyanobacteria-
Synechococcus_sp._CC9902_78184860:0.10711466228247666643)36:0.02300624226685270626)80:0.08438337874070170330
,(Cyanobacteria-Synechococcus_sp._RS9917_87124265:0.09160476561145111662,(Cyanobacteria-
Synechococcus_sp._WH_7805_88808447:0.04789640966192963700,Cyanobacteria-
Synechococcus_sp._WH_7803_148239421:0.01173541667244351432)75:0.04023051580419601675)89:0.06608095943018631
180)28:0.03922133340651318828)25:0.03459611960026173078)80:0.11062613862209577653,(Cyanobacteria-
Prochlorococcus_marinus_str._MIT_9303_124023305:0.00982038214515238740,Cyanobacteria-
Prochlorococcus_marinus_str._MIT_9313_33862905:0.01478887761258880669)99:0.18509125458999406422)49:0.042902377
57816767195)41:0.17083439570618083603)94:0.36469459565012990465,(((Bacteria-
Thermus_thermophilus_55980197:0.61293258489926094601,Bacteria-
Anaeromyxobacter_sp._153003131:0.47209000466988304545)27:0.03836102500839477097,(Bacteria-
Desulfuromonas_acetoxidans_95931032:0.56713590383273293494,((Plantae-
Ostreococcus_lucimarinus_34446:0.82717821809530300836,(((((Bacteria-
Algoriphagus_sp._126645751:0.12437902048408934486,Bacteria-
Flavobacterium_johnsoniae_146301237:0.14936567591091581830)91:0.09916949409094409640,Bacteria-
unidentified_eubacterium_149372591:0.18424491498668271738)39:0.04193564553701406572,((Bacteria-
Psychroflexus_torquis_91215704:0.25651098958502382974,(Bacteria-
Gramella_forsetii_120435456:0.14317755129671180803,Bacteria-
Flavobacteria_bacterium_89891453:0.24899258346547673004)49:0.06791006431926535636)35:0.04416028684586571079,(Ba
cteria-Parabacteroides_distasonis_150008694:0.07656820608462885924,Bacteria-
Bacteroides_thetaiotaomicron_29346972:0.27605857194858218762)93:0.15929232059555442635)18:0.0515443774528881598
6)40:0.04903474694238520865,Bacteria-
Pedobacter_sp._149279619:0.28880940258383436259)52:0.07451424780445185803,Bacteria-
Cytophaga_hutchinsonii_110639170:0.30573262833887904222)85:0.32161911674761334279)39:0.08849953801637923390,(B
acteria-Acidobacteria_bacterium_94967055:0.71244319214981055577,(Bacteria-
Pelobacter_propionicus_118582011:0.23381827694206888668,Bacteria-
Geobacter_bemidjiensis_145617559:0.21902442225116489083)92:0.13764331940164362122)40:0.09616598005810413075)15
:0.06004554559884989207)15:0.10944432400688089424)11:0.11465229954540066826,(((Bacteria-
Chromobacterium_violaceum_34495972:0.14236027666091455313,Bacteria-
Neisseria_meningitidis_15677847:0.25435884045879358917)91:0.12270406653706109057,(Bacteria-
Nitrosomonas_eutropha_114331598:0.39879805521043637517,((Bacteria-
Polaromonas_sp._91787849:0.38290225435764618789,(Bacteria-
Ralstonia_solanacearum_17546914:0.10504326388289758620,Bacteria-
Herminiimonas_arsenicoxydans_134093811:0.19845896493093467816)42:0.02943637679866331971)21:0.0209792094648524
2952,Bacteria-
Burkholderia_mallei_100263801:0.10933319186442541637)34:0.09493462412273680207)48:0.13562864689846851340)56:0.1
2824773615490714374,(Bacteria-Legionella_pneumophila_148358907:0.53622343725858701013,(Bacteria-
Beggiatoa_sp._153875294:0.35728630594127508413,Bacteria-
marine_gamma_119477458:0.29346671424122700023)77:0.12521636564066437880)93:0.14709201594664902957)99:0.23677
196485265544768)1:0.03672108604472445331)17:0.08672929715820207330,((Chlamydia-
Candidatus_Protochlamydia_46447342:0.37173050804871604624,(Chromalveolata-
Phaeodactylum_tricornutum_45238:0.35340635729239672225,Chromalveolata-
Aureococcus_anophagefferens_59907:0.62490666231858993385)92:0.28408597405957386473)93:0.21242593804040860173,P
lantae-
Cyanidioschyzon_merolae_CMO211C:0.52432946366816490791)97:0.25526931632401500538)24:0.09416678988008989060,(
Bacteria-Thermotoga_lettingae_157364139:0.75094919517465252845,Bacteria-
Syntrophomonas_wolfei_114567973:0.49356317337289590252)15:0.03418533578537009260)28:0.07049598482517585585)35
:0.09001821388718955763)13:0.07940263875955021200,Bacteria-
Carboxydothermus_hydrogenoformans_78045158:0.20762070659355921776)10:0.04397554750673934865,(Bacteria-
Clostridium_thermocellum_125974811:0.28225696123357146838,((Bacteria-
Desulfotomaculum_reducens_134301122:0.35878058333358653353,Bacteria-
Pelotomaculum_thermopropionicum_147679192:0.20482507277433806281)49:0.06921036275864422826,(Bacteria-
Halothermothrix_orenii_89210925:0.30546834071385803311,Bacteria-
Thermosinus_carboxydivorans_121533966:0.20125785557839290241)65:0.07826399181468080002)25:0.04594328339597877
897)12:0.04203906042003188043)17:0.06216908043181513382,(Bacteria-
Staphylococcus_haemolyticus_70725024:0.28039006943027167118,(((Bacteria-
Lactococcus_lactis_30024073:0.41689118693469484622,((Bacteria-
Oenococcus_oeni_118587245:0.37417259262775076634,(Bacteria-
Streptococcus_thermophilus_116628680:0.13171740688573263522,Bacteria-
Leuconostoc_mesenteroides_116619018:0.12217308954985908742)96:0.10554892549002442559)35:0.05593915650977510784
4,(Bacteria-Lactobacillus_delbrueckii_104773351:0.16570197412363310563,Bacteria-
Pediococcus_pentosaceus_116493557:0.15328607866499133205)42:0.04205821741534690461)37:0.03264191840913173437)5

2:0.12871033787830282735,Bacteria-
Listeria_monocytogenes_153165323:0.09923058749693522418)26:0.06436507651185681411,Bacteria-
Bacillus_pumilus_157694418:0.07774096991336482132)30:0.06700660801893863761)23:0.10325124386295690682)25:0.044
87221353285297809)59:0.05711412075930479698,Bacteria-
Ruminococcus_obeum_153812746:0.25495283135036195254)36:0.02919858421285811273,(Bacteria-
Dorea_longicatena_153853186:0.08452707072257709120,Bacteria-
Bifidobacterium_adolescentis_119025480:0.19929856685874250410)37:0.03852162626054670552,Bacteria-
Eubacterium_ventriosum_154483673:0.14930700791711035147);

Plantae-Cyanidioschyzon_merolae_CMR313C

((Bacteria-Sagittula_stellata_126729314:0.09875640091427689837,(Bacteria-
Silicibacter_sp._99080410:0.05531968267835998820,Bacteria-
Roseobacter_sp._126738727:0.06449492615587248956)100:0.06409821871367760160)99:0.07430575491570107216,(((Bacteri
a-Granulibacter_bethesdensis_114327031:0.31029437427399086946,((Bacteria-
Salinibacter_ruber_83814939:0.44180148808848890285,(Bacteria-
delta_proteobacterium_94263231:0.42593515243740293075,Bacteria-
Syntrophobacter_fumaroxidans_116747980:0.35685419778308435923)93:0.11879243137987315071)97:0.11566284227561304
809,(Bacteria-Helicobacter_pylori_15611489:0.83059154280799540881,((Bacteria-
Petrotoga_mobilis_145622188:0.77911598956587213571,(Bacteria-
Psychrobacter_sp._148653878:1.34959908086905366886,(((Bacteria-
Borrelia_burgdorferi_15594593:0.60654422560176712853,Bacteria-
Treponema_denticola_42528139:0.56283336979189702021)54:0.10548138738745099774,(((Bacteria-
Anaeromyxobacter_dehalogenans_86158477:0.99651278667891229901,(((((Bacteria-
Bacillus_subtilis_16078219:0.20251538634617063939,Bacteria-
Geobacillus_thermodenitrificans_138894374:0.13595890916035524576)76:0.06311948347505652590,Bacteria-
Staphylococcus_haemolyticus_70726958:0.48055702746560274852)88:0.07827305222354996594,(Bacteria-
Listeria_monocytogenes_153175453:0.26401972675792434453,((Bacteria-
Streptococcus_pneumoniae_148992885:0.15550993971661636328,Bacteria-
Lactococcus_lactis_125624692:0.30138655199381775285)100:0.29512156344867157554,(((Bacteria-
Pediococcus_pentosaceus_116493361:0.86027398281548173031,Bacteria-
Oenococcus_oeni_116491177:0.32357486393308920736)59:0.08951046426521337696,Bacteria-
Leuconostoc_mesenteroides_116617560:0.32425473992573022874)66:0.07336901250195182467,Bacteria-
Lactobacillus_plantarum_28378823:0.39589080340636134681)33:0.05794209391990419455)100:0.14347117545742626987)8
9:0.09376892329475179644)94:0.12025061193907360391,((Bacteria-
Eubacterium_ventriosum_154484570:0.36203124095156363760,Bacteria-
Bacteroides_capillosus_154496475:0.45051529097446696426)95:0.16183762955461231114,(Bacteria-
Thermosinus_carboxydivorans_121535192:0.44044959921107551537,(((Bacteria-
Desulfotomaculum_reducens_134300296:0.54428240482408152268,Bacteria-
Desulfitobacterium_hafniense_89894637:0.36456349934851106642)20:0.03237183314890552943,(Bacteria-
Syntrophomonas_wolfei_114567434:0.35370341894143375594,(Bacteria-
Thermoanaerobacter_ethanolicus_114845101:0.26354205013318776629,Bacteria-
Clostridium_thermocellum_125974937:0.27756633299400085280)51:0.05127004459203819697)37:0.05469459810143258160)
28:0.03145615157225715997,Bacteria-
Halothermothrix_orenii_89209725:0.33905056150238038759)19:0.02036081698999543318)49:0.03486199748686655969)72:0
.06768098149295932731)31:0.05711904316060973513,(((Bacteria-
Chloroflexus_aggregans_118045798:0.29327620808907806094,Bacteria-
Roseiflexus_sp._148655405:0.22238344773390492093)99:0.15871526358651219524,(Bacteria-
Deinococcus_geothermalis_94985188:1.01350464515492033613,Bacteria-
Herpetosiphon_aurantiacus_113939858:0.42508863861132129003)42:0.08947335961459633447)53:0.12331198391902693789,
Bacteria-
Symbiobacterium_thermophilum_51891875:0.25401313363519362198)37:0.08799426514281584455)15:0.0473907706664969
3276)16:0.07307484735596110614,Bacteria-
Alkaliphilus_metalliredigens_150391496:0.50803203521113771135)40:0.08272986893230142857,(Plantae-
Cyanidioschyzon_merolae_CMR313C:0.48609834524497125274,(Plantae-
Porphyra_yezoensis_AU188724_1:0.68396300510337326717,(Chlamydia-
Candidatus_Protochlamydia_46446812:0.42677413565085509095,((((Chlamydia-
Chlamydophila_pneumoniae_33241472:0.00000121823947223981,Chlamydia-
Chlamydophila_pneumoniae_15618060:0.00000121823947223981)27:0.00000121823947223981,Chlamydia-
Chlamydophila_pneumoniae_15835671:0.00000121823947223981)15:0.00000121823947223981,Chlamydia-
Chlamydophila_pneumoniae_16752907:0.00000121823947223981)100:0.15604204885248038059,((Chlamydia-
Chlamydophila_felis_89898174:0.06151249573918710772,(Chlamydia-
Chlamydophila_abortus_62185225:0.08542347200831854059,Chlamydia-

Chlamydophila_caviae_29840399:0.04939893807188608377)99:0.05484670629533869995)100:0.09662203645327899215,(Chl
amydia-Chlamydia_muridarum_15835007:0.04685053050286965698,(Chlamydia-
Chlamydia_trachomatis_76788827:0.00371715201284112578,Chlamydia-
Chlamydia_trachomatis_15604831:0.00576264968194992698)100:0.08825675130318515194)100:0.21899523853347188651)8
3:0.08201432699132746307)100:0.49493658916110955248)58:0.07802662702904668290)25:0.03759120008003925123)61:0.1
4915794694026304756)54:0.13439907046991045325)30:0.06651481483214967405,Bacteria-
Mycoplasma_capricolum_83319855:1.27932760406907330974)56:0.13227039240112656304)100:0.55076001533050877867)8
4:0.17791341203760852041,(Bacteria-Thermus_thermophilus_46199470:0.67481512790503861421,(Bacteria-
Pelobacter_propionicus_118579806:0.38348528598248277088,Bacteria-
Geobacter_metallireducens_78222509:0.19804070196801065729)100:0.37080396076459731791)87:0.11406204823762801370
)74:0.10861130750161955560)99:0.09709506390150415833)100:0.39054439447078775904)100:0.12252332922364828072,(B
acteria-Parvibaculum_lavamentivorans_154252499:0.22508774178880514016,(Bacteria-
Stappia_aggregata_118588384:0.23645328189050585088,(Bacteria-
Sinorhizobium_medicae_150397882:0.11698836808679199950,(Bacteria-
Rhizobium_leguminosarum_116253806:0.08366520937235608080,Bacteria-
Agrobacterium_tumefaciens_17937470:0.09653377748110909817)95:0.05460383163854092697)100:0.131565145975451824 8
8)99:0.08534463000432271351)99:0.09071101393640473853)100:0.21288255495592753319,(Bacteria-
Paracoccus_denitrificans_119384569:0.17270411400703553073,Bacteria-
Rhodobacter_sphaeroides_146277609:0.11281443061204574074)81:0.03786287543596083410)62:0.02773060022259357660,B
acteria-Oceanicola_granulosus_89070420:0.12160970667222667507);

Plantae-Oryza_sativa_115438861
((((Chromalveolata-Emiliania_huxleyi_UI-EH-IJ0-abt-n-11-0-UI.s1_6:0.23047447664383410859,(((((Plantae-
Physcomitrella_patens_140664:0.02510167706613995048,Plantae-
Physcomitrella_patens_196617:0.03937882086530785503)100:0.13161882115260878368,((Plantae-
Arabidopsis_thaliana_15220099:0.03633138319337018279,Plantae-
Arabidopsis_thaliana_15218214:0.03759808791518301496)100:0.16054541206434394529,(Plantae-
Oryza_sativa_115444955:0.10520507061220525646,Plantae-
Oryza_sativa_115438861:0.07235542107288762959)100:0.07360655384795977696)99:0.08070197677895336874)100:0.11246
192990200001849,(Plantae-Chlamydomonas_reinhardtii_188027:0.60706545823268909245,(Plantae-
Volvox_carteri_79605_jgi:0.02751830574544889690,Plantae-
Chlamydomonas_reinhardtii_195230:0.03064408364440703719)100:0.14723808151102366071)73:0.10138763192405389479)
98:0.15418658924048336822,((((((Plantae-Chlamydomonas_reinhardtii_115246:0.23721849602259090228,Plantae-
Volvox_carteri_89266_jgi:0.15712927260768996818)100:0.72623847456782064658,(Plantae-
Ostreococcus_lucimarinus_2522:0.07484343232009735281,Plantae-
Ostreococcus_tauri_24503:0.11944858495866868264)100:0.69520713364333097672)100:0.66281010400739415189,(Chlamydi
a-Candidatus_Protochlamydia_46445875:0.43205993724195596029,(Chlamydia-
Candidatus_Protochlamydia_46445874:0.41262106058993525126,(Chlamydia-
Candidatus_Protochlamydia_46446977:1.33066800497786985602,((Chlamydia-
Chlamydia_muridarum_15835396:0.14228083741485542113,(Chlamydia-
Chlamydia_trachomatis_76789232:0.00000121823947223981,Chlamydia-
Chlamydia_trachomatis_15605223:0.00000121823947223981)100:0.08840374243529418308)100:0.24375810850095402937,((
Chlamydia-Chlamydophila_abortus_62184770:0.04130185571638444325,(Chlamydia-
Chlamydophila_felis_89898687:0.11645769769931092752,Chlamydia-
Chlamydophila_caviae_29839894:0.02797599771027259319)74:0.02254506666869851919)95:0.08749796740704897380,(((Ch
lamydia-Chlamydophila_pneumoniae_15618524:0.00000121823947223981,Chlamydia-
Chlamydophila_pneumoniae_16752426:0.00000121823947223981)21:0.00000121823947223981,Chlamydia-
Chlamydophila_pneumoniae_33241969:0.00000121823947223981)21:0.00000121823947223981,Chlamydia-
Chlamydophila_pneumoniae_15836146:0.00000121823947223981)100:0.17097429511661627055)82:0.0693365705459209752
4)100:0.75737344764938407948)58:0.13769011855403465172)61:0.09663761345211796527)97:0.26471770030979352439)63
:0.11389391366327271393,Chlamydia-
Candidatus_Protochlamydia_46445884:0.32104912368399474154)21:0.05417297868706313219,(((Chlamydia-
Chlamydia_trachomatis_76788778:0.00409350316437316012,Chlamydia-
Chlamydia_trachomatis_15604784:0.00000121823947223981)100:0.01520013300026723004,Chlamydia-
Chlamydia_muridarum_15834955:0.02253969283366556720)100:0.07223361776511226517,((Chlamydia-
Chlamydophila_felis_89898380:0.04582876383242573404,(Chlamydia-
Chlamydophila_abortus_62185050:0.02026952478092169593,Chlamydia-
Chlamydophila_caviae_29840197:0.01540499926200294446)98:0.03722087180702916137)97:0.04345721492223079269,(Chla
mydia-Chlamydophila_pneumoniae_15835884:0.00201447081706041062,((Chlamydia-
Chlamydophila_pneumoniae_15618266:0.00000121823947223981,Chlamydia-
Chlamydophila_pneumoniae_33241692:0.00000121823947223981)40:0.00000121823947223981,Chlamydia-
Chlamydophila_pneumoniae_16752690:0.00000121823947223981)60:0.00000121823947223981)100:0.1317072127891931177

0)54:0.05426092173968377619)100:0.28186568744895734717)34:0.05490038257173490699,((Chromalveolata-
Thalassiosira_pseudonana_25177:1.05642639474183308579,(Chromalveolata-
Phaeodactylum_tricornutum_11615:0.15388176241357928720,Chromalveolata-
Thalassiosira_pseudonana_9045:0.30989513674205493299)100:0.52837950759014329360)100:0.39190386786453640378,((Ba
cteria-Orientia_tsutsugamushi_148284316:0.38310941384333457860,Bacteria-
Rickettsia_bellii_157826528:0.27032246624782257927)100:0.41081347251992927916,Bacteria-
Lawsonia_intracellularis_94986452:0.66312243019526861953)100:0.20157235930296837911)76:0.09898199743431317088)95
:0.19993246569471120888)61:0.06790442189931639172,Plantae-
Glaucocystis_nostochinearum_Contig953_3:0.33496349324974500350)47:0.04371767975286375979)31:0.0746748153267146
8858,((Plantae-Porphyra_yezoensis_Contig1958_1:0.22113323445235028020,Plantae-
Gracilaria_changii_120459344_1:0.28444416922683490867)69:0.07334718390212209560,((Plantae-
Galdieria_sulphuraria_HET_12D10_2:0.37241535572500050755,(Plantae-
Porphyra_yezoensis_Contig1219_3:0.43782171857972268514,Plantae-
Cyanidioschyzon_merolae_CMI040C:0.18261184801349383822)26:0.00560019816958707596)16:0.00399076128583467776,P
lantae-
Galdieria_sulphuraria_HET_38E05_1:0.20270386334174386733)52:0.08505492033999483004)36:0.06111064436651941767)4
7:0.08411117606973374239,(Excavata-Euglena_gracilis_Contig2104_2:0.24360180320882615645,Chromalveolata-
Aureococcus_anophagefferens_27252:0.40246033105123357743)58:0.09724568416473272636)99:0.15879662947800185502,C
hromalveolata-Phaeodactylum_tricornutum_49533:0.22502553471482278358,Chromalveolata-
Thalassiosira_pseudonana_36429:0.14583568106563904454);

Plantae-Oryza_sativa_115452347
((Plantae-Arabidopsis_thaliana_15226817:0.08871496284263542809,((Plantae-
Arabidopsis_thaliana_30694529:0.00000121823947223981,Plantae-
Arabidopsis_thaliana_18410591:0.00000121823947223981)29:0.00000121823947223981,Plantae-
Arabidopsis_thaliana_30694526:0.00000121823947223981)100:0.12198439455420173605)100:0.30881007330819687517,((((P
lantae-Oryza_sativa_115438550:0.46287557917256993978,(Plantae-
Arabidopsis_thaliana_42572443:0.00000121823947223981,Plantae-
Arabidopsis_thaliana_15232459:0.00000121823947223981)100:0.48362869181231726845)98:0.23792449794267844787,((Plan
tae-Physcomitrella_patens_46799:0.11803969040486304853,Plantae-
Physcomitrella_patens_44234:0.00922220511361430267)99:0.41248635631238234067,((((((Plantae-
Oryza_sativa_115448765:1.27325821758301405140,((Plantae-
Arabidopsis_thaliana_18411885:0.00000121823947223981,Plantae-
Arabidopsis_thaliana_42572157:0.10635215512468224053)99:0.37370912741604278562,Plantae-
Arabidopsis_thaliana_18395106:0.23308085192513683381)88:0.34965275794778710727)37:0.18508472559920777223,(((Plan
tae-Oryza_sativa_115441383:0.28270134432996196772,((Plantae-
Arabidopsis_thaliana_18395177:0.06505637343108161552,(Plantae-
Arabidopsis_thaliana_42571597:0.05534878506854476465,Plantae-
Arabidopsis_thaliana_42571595:0.06802518344024294228)52:0.02292271712897111721)100:0.64116461192044404971,(Plant
ae-Arabidopsis_thaliana_42571735:0.00000121823947223981,Plantae-
Arabidopsis_thaliana_42562492:0.01225853217848552404)99:0.69458111042279180314)41:0.25847053203630310225)34:0.2
3918466726422149549,(Plantae-Arabidopsis_thaliana_15226439:1.13535777149765926275,Plantae-
Oryza_sativa_115442349:0.64709125427474201153)32:0.16396013984117122075)15:0.20212056725055979611,(((Plantae-
Arabidopsis_thaliana_18394923:0.13121019064098576878,Plantae-
Arabidopsis_thaliana_22330665:0.18650182647164317840)58:0.13415099756755685667,(Plantae-
Oryza_sativa_115469006:0.57639345166008504329,Plantae-
Oryza_sativa_115448273:0.35602942726762415449)46:0.11986238016440194099)48:0.22353394401833076577,(Plantae-
Physcomitrella_patens_17452:0.00000121823947223981,(Plantae-
Physcomitrella_patens_19427:0.01398158940077614230,((Plantae-
Arabidopsis_thaliana_79315018:0.00000121823947223981,Plantae-
Arabidopsis_thaliana_15232579:0.03713110608739571966)100:0.91825675664149397637,Plantae-
Oryza_sativa_115452347:0.52302215609760038806)97:0.43287319527206125080)50:0.10884362939804749504)55:0.2010132
7512763744743)14:0.18564401274813166487)14:0.14498470545916095076)77:0.45967741618736335241,((Plantae-
Arabidopsis_thaliana_15238805:0.85821039419788147651,Plantae-
Arabidopsis_thaliana_15238826:0.93778079273122572079)93:1.05914585599396726678,(((Opisthokonta-
Danio_rerio_125851554:0.02980189131636820990,Opisthokonta-
Danio_rerio_35902819:0.17659353712169906236)97:0.57993429407839980350,Opisthokonta-
Danio_rerio_47086303:0.53108928281152267825)33:0.11638999889645408603,(Opisthokonta-
Danio_rerio_90093320:0.50533955704532662612,Opisthokonta-
Apis_mellifera_66517026:0.74602879066100524774)33:0.08879067449667261969)99:0.75195923742586523453)19:0.155680
83216125708224)40:0.35375201509016718227,Plantae-
Arabidopsis_thaliana_15231350:1.93575901510326642274)81:0.64768853521033042941,((((Opisthokonta-

Danio_rerio_113673508:0.18546320578394076550,(((Opisthokonta-
Mus_musculus_94388214:0.02638418185370853750,Opisthokonta-
Mus_musculus_94388216:0.00000121823947223981)53:0.02418739691030184075,((Opisthokonta-
Mus_musculus_82957134:0.03435727126657848324,((Opisthokonta-
Mus_musculus_82957130:0.01836434252966131705,Opisthokonta-
Mus_musculus_94383817:0.00000121823947223981)100:0.16742226031647447493,Opisthokonta-
Mus_musculus_82918359:0.01127177657403180197)38:0.01021801430391905061)9:0.01071116074347117607,Opisthokonta-
Mus_musculus_94400771:0.00610558640163008908)15:0.00051387157079053111)89:0.05422716230551461597,(((Opisthoko
nta-Mus_musculus_94388212:0.02469933371499502539,(Opisthokonta-
Mus_musculus_94371445:0.00000121823947223981,(Opisthokonta-
Mus_musculus_94383812:0.00000121823947223981,(Opisthokonta-
Mus_musculus_82957137:0.01846133652712166756,Opisthokonta-
Mus_musculus_82918361:0.00000121823947223981)48:0.00638074935114063373)25:0.00612910137401890230)16:0.000001
21823947223981)79:0.01252126100144676860,Opisthokonta-
Mus_musculus_94371441:0.00000121823947223981)90:0.04865890719009709209,((Opisthokonta-
Mus_musculus_94400769:0.00000121823947223981,(Opisthokonta-
Mus_musculus_94400767:0.00000121823947223981,Opisthokonta-
Mus_musculus_94400765:0.00000121823947223981)38:0.00000121823947223981)90:0.01231623497945024716,(((((Opisthok
onta-Mus_musculus_94388210:0.01850498581802377182,(Opisthokonta-
Mus_musculus_94398611:0.00000121823947223981,Opisthokonta-
Mus_musculus_51769129:0.00000121823947223981)98:0.01226463351323901427)26:0.00000121823947223981,Opisthokonta
-Mus_musculus_31559916:0.00000121823947223981)0:0.00000121823947223981,(Opisthokonta-
Mus_musculus_94371439:0.00000121823947223981,(Opisthokonta-
Mus_musculus_37674277:0.00000121823947223981,Opisthokonta-
Mus_musculus_94371443:0.00000121823947223981)11:0.00000121823947223981)0:0.00000121823947223981)0:0.00000121
823947223981,Opisthokonta-
Mus_musculus_82801284:0.00000121823947223981)13:0.00000121823947223981,(Opisthokonta-
Mus_musculus_82918363:0.00000121823947223981,Opisthokonta-
Mus_musculus_82918357:0.00613626106404713354)72:0.00612056572175310749)42:0.00000121823947223981)46:0.013198
41820039201891)20:0.01605043283049284800)95:0.13848056993028581840)100:0.53272472014100646920,(((Opisthokonta-
Apis_mellifera_110762180:0.38218200216233916278,((Opisthokonta-
Drosophila_melanogaster_24582366:0.00000121823947223981,Opisthokonta-
Drosophila_melanogaster_24582368:0.00000121823947223981)33:0.00000121823947223981,Opisthokonta-
Drosophila_melanogaster_17136728:0.00000121823947223981)100:0.39385911240002347000)100:0.43305163772485699702,
(Opisthokonta-Mus_musculus_18875324:0.00000121823947223981,(Opisthokonta-
Danio_rerio_125828935:0.47640126914017877358,Opisthokonta-
Danio_rerio_125828898:0.50018201641506709887)100:1.04541006518286594407)100:0.73082402826222969683)94:0.369637
03298930811192,((((Opisthokonta-Danio_rerio_125826898:0.10568739970639355608,Opisthokonta-
Mus_musculus_6671602:0.33070424535859760384)92:0.09337352721694769375,(Opisthokonta-
Mus_musculus_7710036:0.08552802186750134428,Opisthokonta-
Danio_rerio_47087353:0.18416181120430030749)100:0.28442964486475896813)96:0.07701179411001930764,((Opisthokonta
-Danio_rerio_67972636:0.11188913859113763793,Opisthokonta-
Danio_rerio_47086935:0.24582123271215433613)43:0.02370811608050448285,Opisthokonta-
Mus_musculus_6754222:0.26085578372181994489)48:0.09654706824292320910)100:0.70288456455500991638,(Opisthokont
a-Schizosaccharomyces_pombe_19111886:0.93061157915029124599,((Opisthokonta-
Drosophila_melanogaster_17864096:0.05468827384392714597,Opisthokonta-
Drosophila_melanogaster_28571891:0.00000121823947223981)100:0.71577666838440723307,((Opisthokonta-
Mus_musculus_17157989:0.07234153821099742476,((Opisthokonta-
Danio_rerio_68380500:0.03199038236941911806,Opisthokonta-
Danio_rerio_41056104:0.00000121823947223981)81:0.07685722310364519194,((Opisthokonta-
Danio_rerio_47086453:0.00000121823947223981,Opisthokonta-
Danio_rerio_125828089:1.03847281524669132757)95:0.08348304246139412466,Opisthokonta-
Mus_musculus_6678940:0.37751099009845728860)48:0.04432733343792558123)46:0.04775214300950151453)72:0.1215822
8226047158860,Opisthokonta-
Drosophila_melanogaster_62484464:0.41059631209698010945)85:0.22387340103706379613)96:0.27845479592838073835)41
:0.06762430789925967523)28:0.12954833726741254996)32:0.14036127183705671140)64:0.08655009133723511050,(((Planta
e-Cyanidioschyzon_merolae_CMR392C:1.84924695365251667845,(((Plantae-
Physcomitrella_patens_115524:0.00000121823947223981,Plantae-
Physcomitrella_patens_115456:0.00000121823947223981)100:0.04386176968298083345,(Plantae-
Physcomitrella_patens_113207:0.18885889129457530955,Plantae-
Physcomitrella_patens_139747:0.07048846463509099436)68:0.05213726536921092780)99:0.51612264717051703489,(Plantae
-Arabidopsis_thaliana_15220810:0.65292537488481383612,Plantae-

Oryza_sativa_115445085:0.31307631591272788718)60:0.13127979695197936061)76:0.19650601865545186397)19:0.0379874
3835980054190,((Plantae-Oryza_sativa_115482462:0.37857669263342874633,(Plantae-
Arabidopsis_thaliana_22328624:0.23422224359206372801,Plantae-
Arabidopsis_thaliana_15226106:0.28373465698878957975)71:0.12641479973883559818)94:0.18446778308215663889,(((((Pla
ntae-Arabidopsis_thaliana_42573682:0.00000121823947223981,Plantae-
Arabidopsis_thaliana_18423760:0.00000121823947223981)32:0.00000121823947223981,Plantae-
Arabidopsis_thaliana_30696616:0.00000121823947223981)100:0.12425411146604761758,(Plantae-
Arabidopsis_thaliana_18416794:0.03758129734856704290,Plantae-
Arabidopsis_thaliana_79325275:0.13592695655859313164)98:0.11703459560943997586)99:0.21304567519273970300,(((Plan
tae-Arabidopsis_thaliana_18422817:0.00000121823947223981,Plantae-
Arabidopsis_thaliana_30695248:0.00000121823947223981)100:0.41799235142394775000,(Plantae-
Arabidopsis_thaliana_18398061:0.00000121823947223981,Plantae-
Arabidopsis_thaliana_30680456:0.00000121823947223981)100:0.24438539421519214767)18:0.04186507117616029838,(Plant
ae-Oryza_sativa_115473043:0.35926494308039308434,Plantae-
Oryza_sativa_115486383:0.15439089346409037851)42:0.06180950857604069343)27:0.08312880870732244909)62:0.1146397
3536204256909,((Plantae-Physcomitrella_patens_119381:0.00000121823947223981,Plantae-
Physcomitrella_patens_119432:0.00000121823947223981)100:0.06220510430466007906,Plantae-
Physcomitrella_patens_19879:0.00000121823947223981)100:1.10880609335487423373)57:0.13419902421695412142)63:0.19
606597267271452800)14:0.08425208929294016047,Chromalveolata-
Phytophthora_sojae_133840:0.58131387030202785748)42:0.04318400827808421827)88:0.36297606803003640286,((((Plantae
-Physcomitrella_patens_19797:0.30475519142882651069,(Plantae-
Oryza_sativa_115489714:0.33338752783636660393,((Plantae-
Arabidopsis_thaliana_15235002:0.01452149470785730687,Plantae-
Arabidopsis_thaliana_30692258:0.04738503792282666022)93:0.10347924030437280485,(Plantae-
Arabidopsis_thaliana_30692254:0.06748049826844781940,Plantae-
Arabidopsis_thaliana_30692256:0.00000121823947223981)78:0.04523785713191716407)100:0.21860187538514400107)56:0.
17813005148787222631)38:0.12717860576631218050,(Plantae-
Physcomitrella_patens_118845:0.37258555558135791896,Plantae-
Arabidopsis_thaliana_15231557:1.43312770535005662431)89:0.22369897072280961470)94:0.32723600048699813536,((Plant
ae-Arabidopsis_thaliana_30682622:0.00000121823947223981,Plantae-
Arabidopsis_thaliana_15236359:0.00000121823947223981)100:0.42191383313324187787,(((Amoebozoa-
Acanthamoeba_castellanii_Contig272_3:1.28409210584016642009,Plantae-
Glaucocystis_nostochinearum_Contig208_2:1.03022202150932318254)66:0.63942629872180856143,(Plantae-
Arabidopsis_thaliana_15221187:0.73010506819226161923,(Plantae-
Oryza_sativa_115473215:1.07529535933197872843,Plantae-
Oryza_sativa_115488680:0.19436564029646499763)67:0.25824601511149386512)62:0.20894750972913397891)4:0.00000121
823947223981,(((Plantae-Arabidopsis_thaliana_15229525:0.00000121823947223981,Plantae-
Arabidopsis_thaliana_30687226:0.00000121823947223981)100:0.38417308976645209162,Plantae-
Oryza_sativa_115441831:0.72864049650707862771)10:0.08571753990857734762,(Plantae-
Physcomitrella_patens_208328:0.61738219278582040506,Plantae-
Physcomitrella_patens_37877:0.57049312051470557616)88:0.44730857868152285040)9:0.11302062620923720992)17:0.1072
3554245530877993)16:0.11487063685296840243)57:0.32653823543865079237,((Chlamydia-
Candidatus_Protochlamydia_46446527:1.73281370359313036111,Plantae-
Physcomitrella_patens_228226:1.58489720616420592947)68:0.40561494164297073883,(Excavata-
Malawimonas_jakobiformis_Contig1132_3:1.84187636714956082251,Plantae-
Porphyra_yezoensis_Contig824_2:1.38289456049889003530)57:0.32369566069105781825)7:0.17530107898999894300)80:0.4
6671048137059123473)79:0.35513829776414829187)55:0.51129419010054155681,(Plantae-
Physcomitrella_patens_8989:0.00000121823947223981,Plantae-
Physcomitrella_patens_44443:0.08579957083343298097)100:0.26080783985188005225)45:0.21643213293932725416)16:0.05
557768829139853040)39:0.28795879953191233502,Plantae-
Oryza_sativa_115460844:0.70183059329811348093)30:0.22094279660103297025,(Plantae-
Arabidopsis_thaliana_30681615:0.00000121823947223981,Plantae-
Arabidopsis_thaliana_18399877:0.00000121823947223981)100:1.89369357097448487437)86:0.05374086624744971868,Planta
e-Oryza_sativa_115435828:0.35898776957963013379);

Plantae-Oryza_sativa_115460026
(Chlamydia-Candidatus_Protochlamydia_46447393:0.78822149303898536576,((((Chromalveolata-
Aureococcus_anophagefferens_59136:0.29871843293187966273,Chromalveolata-
Phaeodactylum_tricornutum_15852:0.36266127921881013974)100:0.29426638483818501335,(Chromalveolata-
Alexandrium_tamarense_Contig43_6:0.43248677020223269540,(((Opisthokonta-
Mus_musculus_8393066:0.16202440118942573744,Opisthokonta-
Danio_rerio_125821778:0.15487010655483884891)71:0.13884250039346157646,(((Opisthokonta-

Saccharomyces_cerevisiae_6323681:0.90538199512116734891,Opisthokonta-
Magnaporthe_grisea_39939918:0.34635488149270249325)78:0.21976876321067304043,(Plantae-
Cyanophora_paradoxa_CP_aaw_i17_6:0.00000121823947223981,Plantae-
Cyanophora_paradoxa_CP_aar_m06_6:0.00000121823947223981)100:0.54839677805237019292)100:0.43353408860486941645,((Plantae-Physcomitrella_patens_37064:0.64816170836250031062,(Plantae-
Oryza_sativa_115460026:0.20130746145170352879,((Plantae-
Physcomitrella_patens_204256:0.04749619798042505753,Plantae-
Physcomitrella_patens_154825:0.07744291358249806578)100:0.34495003491281905905,((Plantae-
Arabidopsis_thaliana_79317821:0.00000121823947223981,Plantae-
Arabidopsis_thaliana_18391404:0.00000121823947223981)26:0.00000121823947223981,Plantae-
Arabidopsis_thaliana_79317794:0.00000121823947223981)100:0.17783885195725127049)95:0.09414615791006121504)93:0.19251243899174702578)92:0.19899969481657253656,(Plantae-
Ostreococcus_lucimarinus_5907:0.17703616408160888218,Plantae-
Ostreococcus_tauri_37994:0.53120516342137169552)99:0.38426649405797008718)72:0.13926441230035263574)93:0.27327864945334517932)56:0.06949785289300761115,(Opisthokonta-
Apis_mellifera_110776824:0.35165248399846227301,Opisthokonta-
Drosophila_melanogaster_24652342:0.33429343980617842735)56:0.10594267648772591472)100:0.48547833607106893883)36:0.06146652548560953278)31:0.11755407222632564823,(((Plantae-
Physcomitrella_patens_176592:0.09736285669221975136,Plantae-
Physcomitrella_patens_162557:0.08049757175458470415)97:0.12025172661635682991,((Plantae-
Cyanophora_paradoxa_Contig1919_6:0.41643275510497951286,Plantae-
Oryza_sativa_115453035:0.10522366058406303069)30:0.06737818762171222942,Plantae-
Arabidopsis_thaliana_15223944:0.17621884872605211858)31:0.09199167346092515518)33:0.13046005757493076005,((Plantae-Ostreococcus_tauri_22592:0.05990146714630699420,Plantae-
Ostreococcus_lucimarinus_28559:0.06755136136905699751)99:0.22810012668443474526,((Plantae-
Arabidopsis_thaliana_18401659:0.04025943731863500935,Plantae-
Oryza_sativa_115477837:0.07859994989661880926)95:0.12300790278584931847,(Plantae-
Physcomitrella_patens_63006:0.00708296836881524162,Plantae-
Physcomitrella_patens_85183:0.02237370251872236593)87:0.08554617899262730030)92:0.11629429210499139802)90:0.12720769739636783591)56:0.15725695810200365776)33:0.10434207804143109943,Amoebozoa-
Acanthamoeba_castellanii_Contig405_3:0.31320801677883108871)25:0.07241561224412659814,Plantae-
Glaucocystis_nostochinearum_Contig554_2:0.32689024382027653814);

Plantae-Oryza_sativa_115463187

((Cyanobacteria-Synechococcus_sp._RCC307_148241893:0.39244421335794216210,(((((Cyanobacteria-
Prochlorococcus_marinus_str._MIT_9515_123966418:0.04199189717745591827,(Cyanobacteria-
Prochlorococcus_marinus_subsp._pastoris_str._CCMP1986_33861650:0.07801596512250348614,(Cyanobacteria-
Prochlorococcus_marinus_str._AS9601_123968733:0.03005126059024704199,(Cyanobacteria-
Prochlorococcus_marinus_str._MIT_9301_126696539:0.04749142780554165000,Cyanobacteria-
Prochlorococcus_marinus_str._MIT_9312_78779488:0.02939140185121569271)57:0.01431387012727610222)100:0.19579978853480611845)26:0.02886209180664987481)100:0.66912042659423531799,(Cyanobacteria-
Prochlorococcus_marinus_str._NATL2A_72382493:0.01109260382765148503,Cyanobacteria-
Prochlorococcus_marinus_str._NATL1A_124026192:0.01338448629752304712)100:0.36077881153582302964)29:0.17421788190215620196,(Cyanobacteria-Prochlorococcus_marinus_str._MIT_9211_84517841:0.26088558069350548108,Cyanobacteria-
Prochlorococcus_marinus_subsp._marinus_str._CCMP1375_33240551:0.37389193419538552243)56:0.03910010693903052253)18:0.10444997183018484010,(Cyanobacteria-
Prochlorococcus_marinus_str._MIT_9303_124022704:0.02738009930418877635,Cyanobacteria-
Prochlorococcus_marinus_str._MIT_9313_33863326:0.01639141562587607959)100:0.30224951255425652752)37:0.16759864775709998330,((Cyanobacteria-Synechococcus_sp._WH_7805_88808907:0.05647917608321376126,Cyanobacteria-
Synechococcus_sp._WH_7803_148239865:0.06800683869133990200)100:0.18551099511057089764,((Cyanobacteria-
Synechococcus_sp._CC9311_113953749:0.29802810877124286115,(Cyanobacteria-
Synechococcus_sp._WH_8102_33865466:0.12746007499219977332,((Cyanobacteria-
Synechococcus_sp._BL107_116072462:0.06470748885826323182,Cyanobacteria-
Synechococcus_sp._CC9902_78184968:0.08487786700366398152)100:0.12873078117376954288,Cyanobacteria-
Synechococcus_sp._CC9605_78213160:0.12874823269826901617)46:0.01078587971714741779)87:0.06504476323539405691)71:0.04965747455297725432,(Cyanobacteria-
Synechococcus_sp._RS9917_87124746:0.22032713141487580977,Cyanobacteria-
Synechococcus_sp._RS9916_116073039:0.15211194630887786561)90:0.06304648686361989662)65:0.05247616463034354001)84:0.08686911340191072473)25:0.06592538671367657865)39:0.12465501941510484518,((Chromalveolata-
Thalassiosira_pseudonana_268136:0.88238859657180235274,Chromalveolata-
Phaeodactylum_tricornutum_26565:0.74214375662802589684)82:0.32918642075670689895,((((((Bacteria-
Brucella_ovis_148558612:0.22018060342174616828,Bacteria-

Bartonella_bacilliformis_121602233:0.32164868059868612082)97:0.17437898017030059594,(Bacteria-
Agrobacterium_tumefaciens_15890494:0.13704736704618381005,(Bacteria-
Sinorhizobium_medicae_150397980:0.14423756785019498095,Bacteria-
Rhizobium_etli_86359228:0.10337979244520358046)82:0.05247748575795290610)99:0.12583291807883803526)56:0.066162
54438241279956,(Bacteria-Maricaulis_maris_114570227:0.43241941725818716646,((((Bacteria-
Novosphingobium_aromaticivorans_146275644:0.22616324346754257402,Bacteria-
Sphingomonas_wittichii_148556561:0.21095585984322734463)42:0.10470403949251910758,Bacteria-
Caulobacter_crescentus_16125312:0.31627364327239698705)67:0.08376883913203950205,Bacteria-
Erythrobacter_litoralis_85374502:0.20711553721376738113)83:0.07535793214152161634,((Bacteria-
Paracoccus_denitrificans_119383602:0.15826888088521412490,Bacteria-
Dinoroseobacter_shibae_118735313:0.18741732310671291306)56:0.04852384557617404892,((Bacteria-
Oceanicola_granulosus_89069926:0.22808876670688935095,(Bacteria-
Sagittula_stellata_126730032:0.11535819475781181054,(Bacteria-
Roseovarius_sp._149200958:0.12367574137084848263,(Bacteria-
Silicibacter_sp._99078405:0.11965498463167710541,Bacteria-
Roseobacter_sp._126740971:0.10380659214442396199)100:0.13143071681282375573)38:0.02023108633407772611)85:0.052
94479194063277105)48:0.04660142881872744725,Bacteria-
Rhodobacterales_bacterium_126724596:0.29156476877113535195)22:0.01499344044084153019)100:0.150142684370471873
8)78:0.08245268599327816583)47:0.06398751372381611402)17:0.02758593040473476463,(Bacteria-
Acidovorax_avenae_120611219:0.46845608897405666138,(Bacteria-
Rhodoferax_ferrireducens_89900746:0.23626130273001316739,Bacteria-
Polaromonas_sp._91788797:0.18273708663280441300)86:0.08923641673042775890)92:0.17812956696431714310)24:0.05312
910417115626616,((((Bacteria-Candidatus_Pelagibacter_91763202:0.88830155643768016205,(Chromalveolata-
Phytophthora_sojae_138769:1.11974374607640791979,((Plantae-
Physcomitrella_patens_14245:1.19862203602285588566,Chlamydia-
Chlamydophila_felis_89898803:0.94087286936588232500)100:0.74059813951522857067,Chromalveolata-
Phytophthora_sojae_156743:2.09272068596494476012)77:0.33500878448215765104)43:0.14580816775506177940)13:0.1704
0733861877563338,(((Plantae-Arabidopsis_thaliana_18405495:0.16306491233331207868,Plantae-
Oryza_sativa_115463187:0.09428201284814177741)98:0.12933034951554264746,(Plantae-
Physcomitrella_patens_23028:0.17339526522140880083,Plantae-
Physcomitrella_patens_149438:0.07196372596768679797)100:0.14784217314047135017)100:0.47445497688776028244,Bacte
ria-
Methylophilales_bacterium_118594988:0.38378484422208014593)9:0.01788872390898912576)0:0.07984910007031048584,((
Bacteria-Fulvimarina_pelagi_114706561:0.29699978953987571373,Bacteria-
Aurantimonas_sp._90420453:0.30192655707297555434)100:0.47079226642010413295,(((Cyanobacteria-
Anabaena_variabilis_ATCC_29413_75909037:0.02549617248317801593,Cyanobacteria-
Nostoc_sp._PCC_7120_17227880:0.01828760117083517989)100:0.10425518804940911433,(Cyanobacteria-
Nodularia_spumigena_CCY9414_119512665:0.12029538496139993708,Cyanobacteria-
Nostoc_punctiforme_PCC_73102_23126099:0.13797994838666621797)93:0.04962665219996909710)95:0.0923456053666410
0650,(((Cyanobacteria-Synechococcus_elongatus_PCC_7942_81300701:0.00000121823947223981,Cyanobacteria-
Synechococcus_elongatus_PCC_6301_56752212:0.00000121823947223981)100:0.75638472488573282870,Cyanobacteria-
Synechocystis_sp._PCC_6803_16331326:0.43794891041598682513)44:0.25034762504729002330,(Cyanobacteria-
Cyanothece_sp._CCY0110_126660557:0.04671551285180221280,Cyanobacteria-
Crocosphaera_watsonii_WH_8501_67920027:0.17522728074372667662)100:0.18162511129774169816)15:0.05649396453794
499479)16:0.13573940771867365473)4:0.04339531848235149425)2:0.03155263637869933080,((Bacteria-
Pseudomonas_stutzeri_146282054:0.14206663909587841088,Bacteria-
Chromohalobacter_salexigens_92112223:0.12738327964841644757)99:0.16064565989770690502,((Bacteria-
Limnobacter_sp._149928068:0.35026789313149053751,((Bacteria-
Francisella_tularensis_110670639:0.21674906766994606055,((((Bacteria-
Oceanospirillum_sp._89095287:0.18131807603682870145,(Bacteria-
Psychrobacter_cryohalolentis_93005241:0.27520224711570295817,(Bacteria-
Moritella_sp._149909254:0.01650672629820057816,Bacteria-
Psychromonas_sp._90408848:0.08592411017966775089)100:0.13379587429197509207)66:0.06653398085090732328)29:0.02
476945703394052220,(Bacteria-Shewanella_loihica_127512709:0.13962624599696121797,(Bacteria-
Marinomonas_sp._152996554:0.11910540955361630100,Bacteria-
Alteromonadales_bacterium_119471951:0.19285566969506803825)12:0.01465207914705830838)53:0.049462919520850440l
9)33:0.03586629190086929042,(Bacteria-Hahella_chejuensis_83643417:0.18580490499086060874,((Bacteria-
alpha_proteobacterium_114772438:0.13049494449757023662,Bacteria-
Pseudoalteromonas_atlantica_109897289:0.09411434624028604257)99:0.11300465123725175753,(Bacteria-
Vibrio_cholerae_121590743:0.16504156455824176009,(Bacteria-
Photobacterium_profundum_90413309:0.11592643093599343251,Bacteria-
Vibrionales_bacterium_148975250:0.08647686675800081435)75:0.04306735238647844966)85:0.05490693033501362758)75:

0.05136112821773113069)23:0.01343586764076101780)23:0.02991926535237659709,((Bacteria-
marine_gamma_119476504:0.22390454899774397957,Bacteria-
Oceanobacter_sp._94499154:0.10896463502474014462)62:0.04158972469073236172,(Bacteria-
Marinobacter_aquaeolei_120555922:0.19863253840034414410,Bacteria-
Alcanivorax_borkumensis_110834114:0.28347241535797773615)17:0.05403143191414891411)20:0.03611290822755493929)
3:0.01920177433755913474)75:0.10883824307955254629,Bacteria-
Saccharophagus_degradans_90020959:0.17278288321552678908)27:0.02298807423072613682)19:0.06144318227813677646,
Bacteria-
Methylobacillus_flagellatus_91776671:0.22000059206450800486)4:0.02800489934038208339)13:0.07205663255747929430)3
:0.06472361068184327715)1:0.04766413206371511446,(Bacteria-
Deinococcus_radiodurans_15806120:0.36056852507098158256,Bacteria-
Acinetobacter_baumannii_126642739:0.33025295043378999349)92:0.26588764355910898818)29:0.12324045731173732809)2
8:0.10774076427219232699,Cyanobacteria-Synechococcus_sp._WH_5701_87303426:0.42631268716390302576);

Plantae-Oryza_sativa_115470767
((((Bacteria-Thermus_thermophilus_55981108:0.67174961713697178833,(((Cyanobacteria-
Crocosphaera_watsonii_WH_8501_67921975:0.18891335094025052399,(Cyanobacteria-
Nostoc_sp._PCC_7120_17231888:0.00271010936252192181,Cyanobacteria-
Anabaena_variabilis_ATCC_29413_75909486:0.00231102126622733306)100:0.17145229299238248788)100:0.557349484030
41795206,(Bacteria-Carboxydothermus_hydrogenoformans_78044607:0.34249151985222608952,(Bacteria-
Symbiobacterium_thermophilum_51892669:0.36833028827791858273,((Bacteria-
Thermoanaerobacter_ethanolicus_114843672:0.25358063124653662523,(Bacteria-
Clostridium_difficile_126698917:0.30708742564980595624,Bacteria-
Alkaliphilus_metalliredigens_150388828:0.37557834851459859227)98:0.09233096500903718862)84:0.07399274143251892422,((Bacteria-Bacillus_amyloliquefaciens_154686086:0.17676188922002858406,(Bacteria-
Staphylococcus_haemolyticus_70726640:0.35823558506526176348,Bacteria-
Listeria_welshimeri_116872762:0.22779866511461710488)89:0.06863100155405069125)100:0.18241891703705739869,Bacte
ria-
Halothermothrix_orenii_89209752:0.41338364684234091273)29:0.03969266790695510222)46:0.04981403974332196394)16:0
.02485269622217069554)90:0.08490979690019735682)81:0.06130744275021392242,Bacteria-
Roseiflexus_castenholzii_156743526:0.52610553535019100035)70:0.05942522155750567386)95:0.11885033496695750210,(((
Plantae-Oryza_sativa_115470767:0.14381111842233954001,Plantae-
Arabidopsis_thaliana_30678905:0.21055066878864081570)100:0.44467059973466865896,((Chromalveolata-
Phaeodactylum_tricornutum_20608:0.28950054351117882057,Chromalveolata-
Thalassiosira_pseudonana_268644:0.23695385034379690126)100:0.50897450935737253985,(Plantae-
Ostreococcus_lucimarinus_34347:0.12333550667612915019,Plantae-
Ostreococcus_tauri_8234:0.10836589540456373348)100:0.69724497109939298500)46:0.07165117216698098368)65:0.077134
63146438666318,(Plantae-Cyanidioschyzon_merolae_CMH146C:0.79394139927205820140,(Chlamydia-
Candidatus_Protochlamydia_46446277:0.34409854758790670015,((Chlamydia-
Chlamydia_muridarum_15834850:0.03402197604718684709,(Chlamydia-
Chlamydia_trachomatis_76789587:0.00475302746983388008,Chlamydia-
Chlamydia_trachomatis_15605577:0.00159380734999843327)100:0.06686720577882356020)100:0.17219505510398067094,((
(Chlamydia-Chlamydophila_pneumoniae_15618907:0.00000121823947223981,(Chlamydia-
Chlamydophila_pneumoniae_16752027:0.00000121823947223981,Chlamydia-
Chlamydophila_pneumoniae_15836530:0.00000121823947223981)35:0.00000121823947223981)72:0.00160959260776999619
,Chlamydia-Chlamydophila_pneumoniae_33242368:0.00000121823947223981)100:0.20223000578081035128,(Chlamydia-
Chlamydophila_felis_89898059:0.02699215918218582713,(Chlamydia-
Chlamydophila_caviae_29840519:0.04932852983216146764,Chlamydia-
Chlamydophila_abortus_62185343:0.04795694025095856988)49:0.01187614918527076999)100:0.12990980154160189364)10
0:0.09576295811393453761)100:0.31019662105429418597)98:0.12730940471836102446)66:0.07682732181167699348)100:0.
29069426035207468484)47:0.05030305280196544632,(Bacteria-
Treponema_denticola_42526551:0.71164048078437036615,Bacteria-
Anaeromyxobacter_dehalogenans_86157534:0.51394550026910557072)51:0.09651091753414334229)31:0.02329686188900648852,(Bacteria-Magnetococcus_sp._117926996:0.43719467044967436076,(((Bacteria-
Novosphingobium_aromaticivorans_87200495:0.11568392502383499643,(Bacteria-
Sphingomonas_wittichii_148556711:0.12401506349918139938,Bacteria-
Sphingopyxis_alaskensis_103486036:0.08563566314462769946)51:0.05192686815385648413)100:0.29228674287188816105,(
(Bacteria-Rhodobacter_sphaeroides_126463534:0.08124477034851775026,Bacteria-
Paracoccus_denitrificans_119385483:0.09804481509643002413)100:0.22838625223940792197,(Bacteria-
Acidiphilium_cryptum_148259386:0.33886220792983146044,(Bacteria-
Parvibaculum_lavamentivorans_154254063:0.21392924546487751547,(Bacteria-
Xanthobacter_autotrophicus_154244245:0.14309470685512121202,((Bacteria-

Methylobacterium_chloromethanicum_156448768:0.15941126887952794533,(Bacteria-
Nitrobacter_hamburgensis_92115668:0.05281834908797536365,(Bacteria-
Rhodopseudomonas_palustris_91974709:0.05387696479011178097,Bacteria-
Bradyrhizobium_japonicum_27375890:0.03109584381018016749)75:0.02672600107922738083)100:0.1568128919194186066
1)79:0.04069576261666615902,(((Bacteria-Fulvimarina_pelagi_114705224:0.08526932789697382054,Bacteria-
Aurantimonas_sp._90420519:0.07438287007046809340)100:0.04783900841539606708,(((Bacteria-
Bartonella_bacilliformis_121602640:0.22004836909318456484,(Bacteria-
Brucella_ovis_148559480:0.03843896640821546640,Bacteria-
Ochrobactrum_anthropi_153008079:0.03087249935424353497)99:0.03427087231444421456)17:0.01146480919353291307,Ba
cteria-Mesorhizobium_sp._110636259:0.09376562794161261150)36:0.02534746034388667482,((Bacteria-
Agrobacterium_tumefaciens_15887437:0.03917423230772128229,Bacteria-
Rhizobium_leguminosarum_116249886:0.04635385024939367438)100:0.02994818031949327691,Bacteria-
Sinorhizobium_medicae_150398632:0.05371926103561236454)100:0.06149979092004122933)40:0.01912918477848045071)1
00:0.07324247236804311278,Bacteria-
Stappia_aggregata_118590504:0.17496964576920123746)80:0.03089598365958282772)31:0.03231310267089725075)96:0.056
24641866497724629)80:0.05984157770127314369)41:0.04565750976704874287)58:0.05072118323987200722)100:0.1468906
4699656337321,((((Bacteria-Ralstonia_eutropha_113867066:0.08662440385849115576,Bacteria-
Polynucleobacter_sp._145589238:0.17079739555845338539)100:0.06990484168931654363,Bacteria-
Burkholderia_xenovorans_91782608:0.12483355233616848234)99:0.06418904977246314703,(Bacteria-
Bordetella_bronchiseptica_33602856:0.17595390039765454460,(Bacteria-
Janthinobacterium_sp._152979779:0.02769440118316077171,Bacteria-
Herminiimonas_arsenicoxydans_134095034:0.03044805177290103859)100:0.13525158834986000800)82:0.046272288042795
74781)100:0.19297824375016978116,((Bacteria-Haemophilus_influenzae_16272192:0.31039810449413768323,Bacteria-
Saccharophagus_degradans_90022349:0.35232715573761697048)82:0.08621208270993339917,(((Bacteria-
Beggiatoa_sp._153864819:0.23058200659297697910,Bacteria-
Methylococcus_capsulatus_53804618:0.21898176902127827748)88:0.07492474283450119288,Bacteria-
Nitrosococcus_oceani_77165581:0.32099362700688882644)27:0.03239416980347233771,(Bacteria-
Alkalilimnicola_ehrlichei_114321093:0.13810596221230123093,Bacteria-
Halorhodospira_halophila_121998524:0.17932035781500807459)100:0.10260699430067353466)51:0.06250173075197637540
)43:0.05272540869886695875)99:0.13595782797316427470)49:0.05536259919244219951)100:0.15063595882464506093,Bac
teria-Chlorobium_chlorochromatii_78189646:0.69198522741288603921);

Plantae-Oryza_sativa_115474235
(Bacteria-Frankia_alni_111222491:0.71990046672226171154,(((Bacteria-
Carboxydothermus_hydrogenoformans_78043171:0.35450921029975734911,(Bacteria-
Pelotomaculum_thermopropionicum_147677725:0.30897499942282780072,Bacteria-
Desulfotomaculum_reducens_134299650:0.24571193084714018640)92:0.12101913597897122343)97:0.2367093740213372654
6,((((Plantae-Ostreococcus_tauri_9971:0.38050263556420260702,Plantae-
Ostreococcus_lucimarinus_29883:0.22135231442772396182)100:1.92407384803687997454,((Chlamydia-
Candidatus_Protochlamydia_46446032:0.56417122709910638712,(Plantae-
Physcomitrella_patens_109660:0.36185574454241747899,(Plantae-
Oryza_sativa_115474235:0.19714897014951313081,((Plantae-
Arabidopsis_thaliana_18398265:0.00000121823947223981,Plantae-
Arabidopsis_thaliana_42572325:0.00000121823947223981)26:0.00000121823947223981,Plantae-
Arabidopsis_thaliana_42572327:0.00000121823947223981)100:0.18076874981241183860)93:0.09595113721435770404)98:0.
26575582121379892619)100:0.84303349660788073905,(Bacteria-
Syntrophus_aciditrophicus_85858828:0.53726308711829451337,Bacteria-
Cytophaga_hutchinsonii_110637336:0.79315249824066624829)86:0.18794587191143169447)74:0.20935521567721529501)10
:0.08608680056445651430,((((Chlamydia-Chlamydophila_pneumoniae_15618814:0.00000121823947223981,Chlamydia-
Chlamydophila_pneumoniae_16752131:0.00000121823947223981)23:0.00000121823947223981,Chlamydia-
Chlamydophila_pneumoniae_15836438:0.00000121823947223981)47:0.00000121823947223981,Chlamydia-
Chlamydophila_pneumoniae_33242268:0.00267920730169324576)100:1.09101629868527316702,(Bacteria-
Tropheryma_whipplei_28493472:1.02123757931467551430,(Plantae-
Volvox_carteri_108127_jgi:2.40853115292260433478,((((((Bacteria-
Thermobifida_fusca_72161037:0.32978238038913165742,Bacteria-
Nocardioides_sp._119717515:0.27377165209926157541)40:0.04874445391408709083,Bacteria-
Acidothermus_cellulolyticus_117928793:0.41337950981116389215)53:0.06802100528980573046,((Bacteria-
Mycobacterium_vanbaalenii_120403135:0.22242658172355087021,Bacteria-
Corynebacterium_glutamicum_145295462:0.50913942192415262422)96:0.14870259711255950918,Bacteria-
Salinispora_tropica_145593823:0.27561028344484589381)86:0.08938854494089125413)77:0.17044358171369503041,(Bacteri
a-Clavibacter_michiganensis_148272526:0.45570465838107854228,Bacteria-
Bifidobacterium_adolescentis_119025204:0.47969881195616215219)30:0.10028938805806615620)58:0.207650653650517158

51,(((((Bacteria-Prosthecochloris_vibrioformis_145219530:0.28772431884572041705,Bacteria-
Chlorobium_phaeobacteroides_119357463:0.27481354710628669302)100:0.41047391321989479263,((Bacteria-
Lactococcus_lactis_116511200:0.22681982111407336666,Bacteria-
Streptococcus_mutans_24379077:0.19818422806538910086)100:0.56917203412140937591,(Bacteria-
Geobacillus_thermodenitrificans_138893874:0.44805496944470496468,(Bacteria-
Herpetosiphon_aurantiacus_113941000:0.36175979172480993151,((((Cyanobacteria-
Prochlorococcus_marinus_subsp._marinus_str._CCMP1375_33240829:0.26370560176891250093,Cyanobacteria-
Prochlorococcus_marinus_str._MIT_9211_84517578:0.28974264828026358165)32:0.09392032476517098161,(Cyanobacteria-
Synechococcus_sp._RS9916_116073344:0.19065321322631195722,(Cyanobacteria-
Prochlorococcus_marinus_str._MIT_9303_124023693:0.18852085636274143465,(Cyanobacteria-
Synechococcus_sp._WH_5701_87302973:0.25315393589468848656,Cyanobacteria-
Synechococcus_sp._WH_8102_33866175:0.22212323852872867258)81:0.08724634575919967827)49:0.04036257187869030788)86:0.19060952419500945543)58:0.16817843191182654805,Cyanobacteria-
Prochlorococcus_marinus_str._AS9601_123969037:0.63868239189870967820)100:0.31543999013799028930,Bacteria-
Microscilla_marina_124009981:0.69053198838009455240)29:0.07822056853786421571)15:0.06111224182197464577)13:0.09597651646781155121)12:0.03618855295382991666)6:0.05312537980383272990,(Bacteria-
Alkaliphilus_metalliredigens_150392272:0.29026018374210138528,Bacteria-
Clostridium_sp._106893883:0.24766938243772573802)100:0.22836768285748837437)6:0.05062596114926629781,(((((Bacteria-Pseudomonas_fluorescens_70732508:0.20482963280834812214,(((Bacteria-
Candidatus_Blochmannia_33519925:0.58863854815030780543,Bacteria-
Baumannia_cicadellinicola_94676718:0.18996046361957322213)85:0.09748189642098098462,(Bacteria-
Serratia_proteamaculans_157369610:0.06669753303444196646,Bacteria-
Yersinia_enterocolitica_123443414:0.12977813658999565183)80:0.04016554531299375980)47:0.01378677541307849093,(Bacteria-Enterobacter_sakazakii_156935075:0.06415668421025043366,((Bacteria-
Salmonella_enterica_16759358:0.03111657679711026664,((Bacteria-
Escherichia_coli_124528906:0.00000121823947223981,Bacteria-
Shigella_dysenteriae_82775721:0.00747079010115343691)100:0.04877949164472526050,Bacteria-
Citrobacter_koseri_157147020:0.02417027846735252464)40:0.01882459698269554546)99:0.05254645531745543580,Bacteria-
Klebsiella_pneumoniae_152968896:0.04542203296190773509)100:0.09143886521754557295)94:0.06827895831173694252)75:0.04334428386192125393)82:0.04449676350638968880,(Bacteria-
Chromobacterium_violaceum_34497927:0.20561355127697900080,(Bacteria-
Stenotrophomonas_maltophilia_119876202:0.11446856299960968262,Bacteria-
Xanthomonas_campestris_78045802:0.15427853916521475086)100:0.11224800875132286138)74:0.05257043565382341077)99:0.22775715794277331949,(Bacteria-Bdellovibrio_bacteriovorus_42522180:0.47553228821131809934,Bacteria-
Lactobacillus_brevis_116333877:0.62315294622161654381)66:0.10995351331596911471)32:0.04178467310371975635,(Bacteria-Aeromonas_hydrophila_117619780:1.24389760769666235518,Bacteria-
Coxiella_burnetii_153208936:0.45667009066124614192)36:0.14372798583083915003)22:0.10989179661608967919,Bacteria-
Legionella_pneumophila_148358653:0.80631211867369279034)29:0.15680052545907038897)3:0.02181610241610616036,(Bacteria-Treponema_denticola_42527886:0.54207849162427634848,Bacteria-
Borrelia_afzelii_111115023:0.86606840896194614565)40:0.15838532992939580168)1:0.03848846901308733415)3:0.05664504790218485158,(Bacteria-Desulfitobacterium_hafniense_109647094:0.13061834858431853323,Bacteria-
Streptomyces_coelicolor_21222011:0.15132127431762237846)100:0.83819238011948093980)10:0.03234267204154604725)5:0.07075719540306157906)9:0.03153479266740121661)35:0.13268209391708454814)27:0.05819610231788893695,Bacteria-
Bacillus_cereus_30020677:0.93911575409545489102)45:0.13399850136100463494)56:0.18873018810014033364,Bacteria-
Fervidobacterium_nodosum_154250263:0.68822770474256700357)97:0.50424479552219647616,Bacteria-
Propionibacterium_acnes_50843773:0.57511524275823600050);

Plantae-Oryza_sativa_115476574

(Chromalveolata-Thalassiosira_pseudonana_18741:0.00000121823947223981,((Chromalveolata-
Phytophthora_sojae_138165:0.39438456482963807348,((Chlamydia-
Candidatus_Protochlamydia_46447472:0.58663934484741031561,(Excavata-Naegleria_gruberi_3696-gw1.17.131.1:0.56192861117724213216,(Bacteria-
Mycoplasma_genitalium_12045126:2.61641875628180686775,(((Chlamydia-
Chlamydophila_pneumoniae_33241975:0.00000121823947223981,Chlamydia-
Chlamydophila_pneumoniae_15836150:0.00000121823947223981)22:0.00000121823947223981,(Chlamydia-
Chlamydophila_pneumoniae_15618528:0.00000121823947223981,Chlamydia-
Chlamydophila_pneumoniae_16752422:0.00000121823947223981)28:0.00000121823947223981)100:0.25716486548789668776,((Chlamydia-Chlamydia_muridarum_15835400:0.09487732348593913567,(Chlamydia-
Chlamydia_trachomatis_15605228:0.00000121823947223981,Chlamydia-
Chlamydia_trachomatis_76789236:0.00000121823947223981)100:0.21093646716412628450)98:0.33131377763597658070,(Chlamydia-Chlamydophila_caviae_29839890:0.05007631492247521798,(Chlamydia-

Chlamydophila_abortus_62184766:0.07082435995534572259,Chlamydia-
Chlamydophila_felis_89898691:0.10163685826509374910)61:0.01828698835725073996)89:0.18589736233036860402)29:0.05
978850433960048588)70:0.28048077542819249697)82:0.26388013272451099622)76:0.21333048516382666082)52:0.1082138
6500981333700,(Chromalveolata-Aureococcus_anophagefferens_25163:0.89687064732822319169,((Plantae-
Physcomitrella_patens_196421:0.26059966160191466722,((Amoebozoa-
Acanthamoeba_castellanii_EC100974_1:0.59214594166194467473,(Plantae-
Ostreococcus_tauri_19448:0.43078898013915478682,Plantae-
Ostreococcus_lucimarinus_34954:0.23864978112004087141)99:0.55520633782489103858)59:0.26539210657187478626,(Plant
ae-Volvox_carteri_103956_jgi:0.14814790956195025839,Plantae-
Chlamydomonas_reinhardtii_116449:0.19813030870117062654)100:0.40577408996129654684)25:0.17611095826970224465)
18:0.08391804515321076019,(Plantae-Arabidopsis_thaliana_15228560:0.29509303153010746357,Plantae-
Oryza_sativa_115476574:0.40078700356398183624)48:0.15436985445888101731)9:0.07924764511816116686)39:0.09939978
399215429472)31:0.07253417213292533139)100:0.37226413155113935449,Chromalveolata-
Phaeodactylum_tricornutum_12459:0.49603932214760959596)100:0.34120870679042369922,Chromalveolata-
Thalassiosira_pseudonana_36995:0.00000121823947223981);

Plantae-Oryza_sativa_115483650
((Plantae-Oryza_sativa_115483650:0.17588687136955932777,(((Plantae-
Volvox_carteri_81121_jgi:0.07150385539209132280,Plantae-
Chlamydomonas_reinhardtii_130292:0.10981883837095350598)100:0.42578019785021120613,((((Chromalveolata-
Plasmodium_falciparum_124513124:1.16300291785902532027,(((((Chlamydia-
Chlamydophila_felis_89898011:0.04427992865052023802,Chlamydia-
Chlamydophila_abortus_62185386:0.04383372055034895354)80:0.02450328418793887991,Chlamydia-
Chlamydophila_caviae_29840568:0.03324615520311596101)100:0.12296038705614797948,(((Chlamydia-
Chlamydophila_pneumoniae_15618865:0.00000121823947223981,Chlamydia-
Chlamydophila_pneumoniae_15836491:0.00000121823947223981)27:0.00000121823947223981,Chlamydia-
Chlamydophila_pneumoniae_33242326:0.00000121823947223981)16:0.00000121823947223981,Chlamydia-
Chlamydophila_pneumoniae_16752074:0.00000121823947223981)100:0.15079405828794806288)94:0.0659278040257992858
9,((Chromalveolata-Emiliania_huxleyi_Contig713_4:5.11295226738724384319,(Chlamydia-
Chlamydia_trachomatis_15605541:0.00299713203145520043,Chlamydia-
Chlamydia_trachomatis_76789550:0.00000121823947223981)99:0.00000121823947223981)16:0.06490251567342009142,Chla
mydia-
Chlamydia_muridarum_15834812:0.02447246182372898177)16:0.11679461042829521189)20:0.34597337933284844080)10:0.
19084477475576722139,((Chromalveolata-Aureococcus_anophagefferens_3183:0.64848742269031556251,(Chromalveolata-
Phaeodactylum_tricornutum_3262:0.28145628265308147986,Chromalveolata-
Thalassiosira_pseudonana_263714:0.22350776130134741848)97:0.41755133267170413403)23:0.09742855396757832409,Chla
mydia-
Candidatus_Protochlamydia_46446952:0.48284171105267847945)29:0.06619194118988205866)50:0.13793402065230170317,
(Plantae-Gracilaria_changii_120462693_4:0.46297052090093471222,Plantae-
Cyanidioschyzon_merolae_CMJ027C:0.51997274318420239059)95:0.29077336162873373970)96:0.17697534546161303171,(
Plantae-Ostreococcus_lucimarinus_30035:0.19062594011099556757,Plantae-
Ostreococcus_tauri_15515:0.22442427775927412115)99:0.49246147161474757015)95:0.18287085808564265688)97:0.244888
44601977916016,Plantae-
Physcomitrella_patens_216994:0.35301044851518176282)100:0.15824981223074108261)100:0.21622227155734660697,Plant
ae-Arabidopsis_thaliana_15222600:0.00000121823947223981,Plantae-
Arabidopsis_thaliana_30692655:0.00000121823947223981);

Plantae-Ostreococcus_lucimarinus_31465
((Chlamydia-Chlamydia_muridarum_15834836:0.09339975341008771581,((((Plantae-
Cyanidioschyzon_merolae_CML043C:0.97731558116961736449,(((Plantae-
Arabidopsis_thaliana_15217683:0.76799590534159478672,Plantae-
Arabidopsis_thaliana_15222150:1.25642629122455984358)69:0.21978149666746757407,Plantae-
Physcomitrella_patens_162062:0.71225743852059297812)68:0.33466603831060698360,Plantae-
Oryza_sativa_115461911:0.92118814268906090703)67:0.42492432280352360197)100:2.32461450772293254374,(((Chlamydi
a-Chlamydophila_pneumoniae_15836517:0.00000121823947223981,Chlamydia-
Chlamydophila_pneumoniae_16752041:0.00000121823947223981)25:0.00000121823947223981,Chlamydia-
Chlamydophila_pneumoniae_33242354:0.00000121823947223981)25:0.00000121823947223981,Chlamydia-
Chlamydophila_pneumoniae_15618894:0.00000121823947223981)99:0.20453946719844337543)33:0.18261785207692052269
,((Chlamydia-Chlamydophila_abortus_62185355:0.12505578731910019230,Chlamydia-
Chlamydophila_caviae_29840532:0.06521437744445113260)57:0.05225892072518905501,Chlamydia-
Chlamydophila_felis_89898046:0.11928205227070079464)50:0.09928023483904174773)24:0.05636796131469919841,((Chro
malveolata-Phaeodactylum_tricornutum_43446:1.09295055296153886815,(Plantae-

Ostreococcus_tauri_32641:0.29000985979903426415,Plantae-
Ostreococcus_lucimarinus_31465:0.19760702602810564787)100:0.81049112324942762520)85:0.35940735347168834002,Plan
tae-
Ostreococcus_tauri_33167:1.01811423507933018762)92:0.81753838258854361065)100:0.18015244543780792319)100:0.0947
816020412378668 1,Chlamydia-Chlamydia_trachomatis_76789574:0.00000121823947223981,Chlamydia-
Chlamydia_trachomatis_15605564:0.00000121823947223981);

Plantae-Ostreococcus_tauri_18978
(((Cyanobacteria-Gloeobacter_violaceus_PCC_7421_37520371:0.39293393018078770496,Cyanobacteria-
Thermosynechococcus_elongatus_BP-1_22297624:0.21871993868222217028)26:0.04390937062054067058,(((Cyanobacteria-
Trichodesmium_erythraeum_IMS101_113476080:0.17326183463666922080,Cyanobacteria-
Lyngbya_sp._PCC_8106_119484717:0.10713516362069719356)95:0.09347766161114749728,((((Cyanobacteria-
Cyanothece_sp._CCY0110_126658523:0.05634216742010335344,Cyanobacteria-
Crocosphaera_watsonii_WH_8501_67920270:0.04011016438438030907)100:0.17817427350480091741,Cyanobacteria-
Synechocystis_sp._PCC_6803_16331965:0.18165399794023515123)90:0.09211556709952632704,(Cyanobacteria-
Synechococcus_elongatus_PCC_6301_56751072:0.00000121823947223981,Cyanobacteria-
Synechococcus_elongatus_PCC_7942_81299266:0.00000121823947223981)100:0.15860280264215811941)17:0.03734676744
522014841,Cyanobacteria-
Anabaena_variabilis_ATCC_29413_75909959:0.14731720936539341449)19:0.03950699004556953303)35:0.06297662772290
188449,((((((Bacteria-Anaeromyxobacter_dehalogenans_86157853:0.33843396977434714312,Bacteria-
Myxococcus_xanthus_108761817:0.58625660561410630756)100:0.47194172015364066475,((((Chromalveolata-
Aureococcus_anophagefferens_21140:0.34851573869769270075,(Plantae-
Cyanidioschyzon_merolae_CMM013C:0.29392545294687200119,(Plantae-
Ostreococcus_lucimarinus_42734:0.20349312785880546706,Plantae-
Ostreococcus_tauri_18978:0.13942506132701648625)100:0.35863875891117247274)39:0.05891824141299967926)56:0.07540
320163837810341,(Chromalveolata-Thalassiosira_pseudonana_36917:0.21580010177660025050,(Chromalveolata-
Thalassiosira_pseudonana_1283:0.31254645019767707259,Chromalveolata-
Phaeodactylum_tricornutum_24792:0.21087052382065149536)70:0.07926503422709033075)100:0.12741441402948339157)88
:0.10207062167783192974,(Chlamydia-Candidatus_Protochlamydia_46446428:0.33042461993420368938,(Chlamydia-
Chlamydia_muridarum_15835083:0.13667034986616721781,((((Chlamydia-
Chlamydophila_pneumoniae_33241558:0.00000121823947223981,Chlamydia-
Chlamydophila_pneumoniae_15618143:0.00000121823947223981)7:0.00000121823947223981,Chlamydia-
Chlamydophila_pneumoniae_15835754:0.00000121823947223981)25:0.00000121823947223981,Chlamydia-
Chlamydophila_pneumoniae_16752821:0.00000121823947223981)100:0.17315041365893862890,(Chlamydia-
Chlamydophila_caviae_29840339:0.03429658998681517357,(Chlamydia-
Chlamydophila_abortus_62185176:0.05589202601248773744,Chlamydia-
Chlamydophila_felis_89898226:0.04311018211161270902)38:0.01336713248775750137)64:0.04745928672889750816)90:0.09
00415581899194211 7)100:0.53300772528624984492)52:0.09896967190288009919)100:0.60161861037568609145,Bacteria-
Thermotoga_petrophila_148270361:0.66326459416379979217)26:0.05850406901193302978)16:0.08239361639977153817,(Ba
cteria-Dehalococcoides_sp._147668701:0.62338291566842818359,(Bacteria-
Herpetosiphon_aurantiacus_113938472:0.49931432233313999269,((Bacteria-
Halothermothrix_orenii_89211718:0.30869893589536145928,((((Bacteria-
Desulfotomaculum_reducens_134299523:0.21810176724457486230,Bacteria-
Pelotomaculum_thermopropionicum_147677367:0.15640285234004408510)65:0.06011420616823744123,Bacteria-
Moorella_thermoacetica_83590530:0.32868206925269760621)64:0.06196706314171749469,(Bacteria-
Desulfitobacterium_hafniense_89895209:0.23897139851934531829,Bacteria-
Thermosinus_carboxydivorans_121535583:0.21322213601555065066)15:0.03369210255289135231)17:0.03745529251159074
313,((Bacteria-Thermoanaerobacter_ethanolicus_114844820:0.24484552542198409486,((Bacteria-
Clostridium_difficile_126700418:0.11126136700613127595,Bacteria-
Alkaliphilus_metalliredigens_150390106:0.13885424824334682836)86:0.06696240453330844222,(((Bacteria-
Bacillus_subtilis_16079824:0.08635388710553076885,Bacteria-
Geobacillus_thermodenitrificans_138896155:0.11108722619651449637)87:0.04096103742909298906,(((Bacteria-
Streptococcus_gordonii_157150691:0.06121503183539143272,Bacteria-
Lactococcus_lactis_116510984:0.11734213991932729293)100:0.08637074754366312612,(Bacteria-
Pediococcus_pentosaceus_116493017:0.12217310668634071003,Bacteria-
Lactobacillus_casei_116494315:0.17070192426850258616)76:0.04059560118154880576)100:0.12147349867305595739,Bacter
ia-
Listeria_monocytogenes_153166979:0.06398977052390678044)88:0.06678178565285075585)97:0.08906746284467263741,Ba
cteria-
Staphylococcus_saprophyticus_73662429:0.21090686146079090646)99:0.11982831575468599894)39:0.04454297623185873 8
56)18:0.04098895389850593790,(Bacteria-
Caldicellulosiruptor_saccharolyticus_146295324:0.33380154596128891464,Bacteria-

Symbiobacterium_thermophilum_51892304:0.28262444374050427198)28:0.09635710915179156177)3:0.01136329816667950
438)26:0.04187813879964322888)67:0.10362637874702315677,((Bacteria-
Acidobacteria_bacterium_94967175:0.58448749799314803077,(Bacteria-
Desulfovibrio_desulfuricans_78357885:0.60933062975881002732,Bacteria-
Stigmatella_aurantiaca_115379882:0.41416463581863044441)26:0.08760815003406945134)23:0.07073161174245568006,((((
Bacteria-Magnetococcus_sp._117923915:0.36976018719744585539,(Bacteria-
Erythrobacter_litoralis_85373948:0.29969382263145499801,(Bacteria-
Rhodobacterales_bacterium_126726306:0.14031165286621685717,(((Bacteria-
Silicibacter_sp._99080611:0.07380189124693480041,Bacteria-
Roseobacter_sp._126735583:0.09156504027867928830)64:0.02386924405210730585,Bacteria-
Roseovarius_sp._149202809:0.13895658314436459824)38:0.03153690738901929885,(Bacteria-
Sagittula_stellata_126729583:0.14851459399392580307,Bacteria-
Paracoccus_denitrificans_119384626:0.12917014903242415857)47:0.03914997927129240662)42:0.05589684235341548652)1
00:0.12803363138621701167)100:0.29239127002811587941)61:0.09281433037857450552,((((Bacteria-
Pseudomonas_syringae_66044476:0.18091151472187816385,Bacteria-
Marinobacter_algicola_149375024:0.15976335241624772809)36:0.02803235036099648719,Bacteria-
Psychrobacter_sp._148653724:0.35986245981304171293)68:0.06921399453685761483,(Bacteria-
Thiomicrospira_crunogena_78485677:0.25415113325775195996,(Bacteria-
Beggiatoa_sp._153875174:0.20392492649810459171,Bacteria-
Methylococcus_capsulatus_53805144:0.28479782323087782236)42:0.05603045109816904429)35:0.03910290180819098876)3
8:0.07393623586149360161,Bacteria-
Halorhodospira_halophila_121998478:0.24109667492353145657)100:0.25777290319850554301)32:0.09393452448559649892,
(Bacteria-Mariprofundus_ferrooxydans_114777994:0.44017485133369405048,Bacteria-
Desulfuromonas_acetoxidans_95929300:0.24086999685473259314)7:0.05905506591659890475)11:0.05974593828791495420,
(Bacteria-delta_proteobacterium_94263087:0.45053162867139162628,((Bacteria-
Pelobacter_propionicus_118580220:0.22532610198918440192,Bacteria-
Geobacter_lovleyi_118744126:0.25379912189157149971)97:0.13264717695618902238,Bacteria-
Syntrophus_aciditrophicus_85860047:0.57526599254013310869)19:0.06073050387678822354)15:0.06402371059492424632)8
:0.04525532808413595259)50:0.10603993850176145208)37:0.08861968354045718366)9:0.01564676431459196063)25:0.0802
8222817113192522)55:0.12569662033135853285,Bacteria-
Rubrobacter_xylanophilus_108804169:0.61351508643651808672)77:0.09288315615691232485,Bacteria-
Thermus_thermophilus_46199978:0.44954925518992966404)100:0.22797101903719785287,((((((Cyanobacteria-
Synechococcus_sp._CC9902_78183849:0.00699252464264535443,Cyanobacteria-
Synechococcus_sp._BL107_116071540:0.01712121619916786436)100:0.07569586730964400489,(Cyanobacteria-
Synechococcus_sp._CC9605_78211790:0.04792490800763465897,Cyanobacteria-
Synechococcus_sp._WH_8102_33864780:0.05817302638034517470)97:0.04010324033546866507)99:0.07206624861490923406,((Cyanobacteria-Prochlorococcus_marinus_str._MIT_9303_124024179:0.00226029290327897214,Cyanobacteria-
Prochlorococcus_marinus_str._MIT_9313_33864129:0.00953603763896866685)100:0.09226837664142471807,((Cyanobacteri
a-Prochlorococcus_marinus_str._NATL1A_124025063:0.00881640539092576510,Cyanobacteria-
Prochlorococcus_marinus_str._NATL2A_72383473:0.00569271774856764789)100:0.18778081750779013204,(Cyanobacteria-
Prochlorococcus_marinus_subsp._marinus_str._CCMP1375_33239755:0.21217612055046033981,(Cyanobacteria-
Prochlorococcus_marinus_str._MIT_9515_123965539:0.10512039074014695172,(Cyanobacteria-
Prochlorococcus_marinus_str._AS9601_123967830:0.01391457890948992297,Cyanobacteria-
Prochlorococcus_marinus_str._MIT_9301_126695632:0.02965455951994204475)100:0.05702191830336936218)100:0.335558
3377326O285158)60:0.04355814951584106864)96:0.12397210163828230323)80:0.09316984169417534545)25:0.01458545207
185867228,(Cyanobacteria-Synechococcus_sp._CC9311_113953818:0.09316115935334695619,((Cyanobacteria-
Synechococcus_sp._WH_7803_148238624:0.02539798930266220361,Cyanobacteria-
Synechococcus_sp._WH_7805_88808038:0.02105651103073242006)100:0.08893128442652190047,Cyanobacteria-
Synechococcus_sp._RS9917_87125118:0.05789818216254626060)47:0.03260963697667216415)61:0.02235945026272818936
)41:0.04449515671674351275,Cyanobacteria-
Synechococcus_sp._WH_5701_87301968:0.15738387334114620209)100:0.14175272409641795357,Cyanobacteria-
Synechococcus_sp._RCC307_148243410:0.17974677890409521130)100:0.18126327316754167751)55:0.09363020904327822
025)30:0.04422022325643615998)100:0.29150097560569054878,Cyanobacteria-Synechococcus_sp._JA-3-
3Ab_86607442:0.09007656040629970218,Cyanobacteria-Synechococcus_sp._JA-2-3Ba2-
13_86608004:0.01460359306670292075);

Plantae-Ostreococcus_tauri_21840
(((((((Bacteria-Candidatus_Desulfococcus_121542771:0.50580432965369548359,((Bacteria-
Syntrophobacter_fumaroxidans_116751069:0.45729688522538841644,Bacteria-
delta_proteobacterium_94265985:0.68532283050959719173)54:0.13505840544102884482,(Bacteria-
Desulfotalea_psychrophila_51246587:0.67656163295012927694,(Bacteria-
Desulfuromonas_acetoxidans_95930458:0.63540511545487410316,Bacteria-

Pelobacter_carbinolicus_77919601:0.38636400153873967644)60:0.08994947640615481854)40:0.08633518439079701512)62:0
.10830307440435128774)86:0.14454590812974424385,((((Bacteria-
Dehalococcoides_ethenogenes_57234783:0.96958475852956305108,((Bacteria-
Listeria_monocytogenes_153172836:0.31873851549886539347,(Bacteria-
Bacillus_sp._126654250:0.21339656452581898249,Bacteria-
Geobacillus_thermodenitrificans_138893718:0.14727293969188853806)73:0.05676168791357853044)56:0.0752891132787198
4360,(Bacteria-Staphylococcus_haemolyticus_70727515:0.33543867227020546240,Bacteria-
Streptococcus_mutans_24380339:0.60090623634419515486)51:0.12703007758340578714)89:0.18897645546915017856)23:0.
07882281554604111951,((((Bacteria-Clostridium_beijerinckii_150015284:0.39086097809105846412,Bacteria-
Caldicellulosiruptor_saccharolyticus_146297223:0.38764927380264940782)51:0.07647588868369288584,Bacteria-
Thermoanaerobacter_ethanolicus_114844602:0.57703111169784104817)30:0.09925963754962570385,(Bacteria-
Carboxydothermus_hydrogenoformans_78044899:0.62236188646600598684,(Bacteria-
Pelotomaculum_thermopropionicum_147676431:0.40326888787841702610,Bacteria-
Desulfotomaculum_reducens_134297973:0.36272955891870090062)65:0.09758886906518764170)35:0.0877645700279274659
8)3:0.02370495335361340328,Bacteria-
Syntrophomonas_wolfei_114565639:0.50542495637270834496)7:0.06370386093326410593)15:0.08904898744134433219,(((C
yanobacteria-Gloeobacter_violaceus_PCC_7421_37519671:0.32074370156623149830,Cyanobacteria-
Lyngbya_sp._PCC_8106_119485100:0.36640097704202789775)99:0.15939734263731838992,((Cyanobacteria-
Synechococcus_sp._RCC307_148242413:0.36909496337479713590,Cyanobacteria-
Synechococcus_sp._WH_5701_87302769:0.20664979388876356103)98:0.08487849343006849401,((((Cyanobacteria-
Prochlorococcus_marinus_str._MIT_9313_33862893:0.02140898116587524810,Cyanobacteria-
Prochlorococcus_marinus_str._MIT_9303_124023320:0.01814102207658989732)100:0.15272302320479921867,(((Cyanobacte
ria-Prochlorococcus_marinus_str._NATL1A_124025655:0.01143750686827168031,Cyanobacteria-
Prochlorococcus_marinus_str._NATL2A_72382119:0.01058864319700243868)100:0.32136389502894013859,((Cyanobacteria-
Prochlorococcus_marinus_str._MIT_9312_78779251:0.01376529449014437557,(Cyanobacteria-
Prochlorococcus_marinus_str._AS9601_123968461:0.02258911526621447743,Cyanobacteria-
Prochlorococcus_marinus_str._MIT_9301_126696264:0.03122623575575363131)93:0.00751681236199532119)100:0.1096011
0914538991056,(Cyanobacteria-
Prochlorococcus_marinus_str._MIT_9515_123966248:0.04159570339838733477,Cyanobacteria-
Prochlorococcus_marinus_subsp._pastoris_str._CCMP1986_33861489:0.07323491428278591786)100:0.1588341258989264259
7)100:0.38247823473245595727)96:0.22900773907347302116,(Cyanobacteria-
Prochlorococcus_marinus_str._MIT_9211_84518232:0.17303137464864407069,Cyanobacteria-
Prochlorococcus_marinus_subsp._marinus_str._CCMP1375_33240214:0.24873417377522860638)60:0.0566154477213102053
1)78:0.12247556777162706132)40:0.03410798696884049380,((Cyanobacteria-
Synechococcus_sp._CC9311_113955450:0.16719226473438031366,Cyanobacteria-
Synechococcus_sp._RS9917_87124271:0.09591458408731375063)91:0.03762373995521550718,(Cyanobacteria-
Synechococcus_sp._WH_7803_148239701:0.09754601469968866845,Cyanobacteria-
Synechococcus_sp._WH_7805_88808455:0.10562038494492301255)99:0.13644709010789549120)96:0.106416587637117657
23)34:0.03557255572731609500,(Cyanobacteria-
Synechococcus_sp._WH_8102_33865587:0.13451784349492013071,((Cyanobacteria-
Synechococcus_sp._CC9902_78184855:0.04527988298414252488,Cyanobacteria-
Synechococcus_sp._BL107_116070729:0.02849048796983612991)100:0.12379257893099687715,Cyanobacteria-
Synechococcus_sp._CC9605_78212719:0.11302549084352898900)45:0.03941248648391498799)88:0.05252948334178433182
)64:0.12667186155937851244)100:0.44392061804960036842)72:0.13151750935283246235,(((Bacteria-
Streptomyces_coelicolor_21221586:0.35756711743049401919,((Bacteria-
marine_actinobacterium_88856862:0.12351366251748431557,(Bacteria-
Clavibacter_michiganensis_148273551:0.15041241463475199525,Bacteria-
Leifsonia_xyli_50955321:0.13061150165862348005)56:0.02864165299431507133)100:0.19400724439862035431,Bacteria-
Arthrobacter_aurescens_119960939:0.37742815411766006406)100:0.20073981828456680110)84:0.09374359821886843469,B
acteria-Nocardioides_sp._119718075:0.46122598634581268850)100:0.38211728909589659509,Bacteria-
Acidobacteria_bacterium_94971567:0.98124624125228399052)31:0.11015679484271953537)9:0.08351544055248498466)5:0.
07329131447291850621,Bacteria-
Desulfitobacterium_hafniense_109647798:0.62318194604832555239)6:0.06924907450159699385)20:0.1015240961624266635
7,((Bacteria-Francisella_tularensis_110669898:0.80086783939436723134,(((((Bacteria-
Ralstonia_pickettii_121529190:0.20133671980147566094,Bacteria-
Burkholderia_xenovorans_91781714:0.30514701479684980256)44:0.05927161222464966128,(Bacteria-
Herminiimonas_arsenicoxydans_134096052:0.06047797748221732383,Bacteria-
Janthinobacterium_sp._152980074:0.07527345939197145619)100:0.30035779974866877451)43:0.05625648065892469712,(Ba
cteria-Polaromonas_naphthalenivorans_121603809:0.28662747980258751479,Bacteria-
Comamonas_testosteroni_118052186:0.21105684619447345751)99:0.32016414253030522907)38:0.07233124639714712256,B
acteria-Bordetella_bronchiseptica_33599889:0.36947078599635330143)70:0.13723609142080681056,((Bacteria-
Pseudomonas_syringae_71737559:0.37414937630649008904,((Bacteria-

Shewanella_putrefaciens_146291903:0.3635657876940670552O,(Bacteria-
Aeromonas_salmonicida_145298190:0.29194511611555007402,Bacteria-
Pseudoalteromonas_tunicata_88861341:0.45498763258640162466)22:0.03208079875524270236)39:0.04867937318368160265,
((Bacteria-Hahella_chejuensis_83644564:0.36194336754197720118,Bacteria-
Marinobacter_algicola_149377998:0.30854528791040580771)61:0.13195441913860342376,(Bacteria-
Klebsiella_pneumoniae_152970784:0.28542007461253837697,((Bacteria-
Haemophilus_somnus_113461140:0.20590224713573532234,Bacteria-
Actinobacillus_succinogenes_152979409:0.14617664174342423489)99:0.10314581772368348889,Bacteria-
Photobacterium_profundum_90412787:0.30437623369829985842)71:0.06572043350720080390)74:0.04958085207212831191)
30:0.05639270311216848519)26:0.04924448074787884932)35:0.07476972781447245586,(Bacteria-
Alkalilimnicola_ehrlichei_114319444:0.38447718296670646554,Bacteria-
Methylococcus_capsulatus_53804650:0.43358261414262544431)16:0.06004612554152993775)56:0.07653165285716197197)9
8:0.35649264760873400348)71:0.13496298711335599774,(Bacteria-
Mariprofundus_ferrooxydans_114777711:0.86798601813777853664,Bacteria-
Magnetococcus_sp._117924127:0.55093953360104630512)58:0.18050525446607301738)69:0.09174854235373090805)44:0.1
8350785676940264968,Bacteria-
Parabacteroides_merdae_154492215:1.06547468201954775857)95:0.29709505110820549900,((((Chlamydia-
Candidatus_Protochlamydia_46447223:0.44921766561080794800,(Plantae-
Gracilaria_changii_120463315_2:0.37083443146026595638,Plantae-
Cyanidioschyzon_merolae_CMS444C:0.61824056561239670859)21:0.02263565118511486757)62:0.13485445926638431335,(
Chromalveolata-Phaeodactylum_tricornutum_51700:0.22602676629827855859,Chromalveolata-
Thalassiosira_pseudonana_31907:0.15686252627009036886)100:0.29697232093384340335)45:0.04790055995429806646,((Pla
ntae-Physcomitrella_patens_190580:0.18773310521669753226,(Plantae-
Arabidopsis_thaliana_15225820:0.14415777880428071178,Plantae-
Oryza_sativa_115440529:0.09944200235971600854)53:0.07017749654344011823)100:0.20218945913073016474,((Plantae-
Ostreococcus_lucimarinus_4287:0.05213456444895723380,Plantae-
Ostreococcus_tauri_21840:0.08033420933287499510)99:0.08329626807719568704,Plantae-
Chlamydomonas_reinhardtii_137673:0.30359840380890551481)99:0.16619447265233358579)99:0.16741160004036145792)5
5:0.10230775830196663090,Chromalveolata-
Aureococcus_anophagefferens_65516:0.87422770944854388819)90:0.14929111412709844542)100:0.67308853931310064223,
(((Chlamydia-Chlamydia_trachomatis_15605538:0.00404898169859768155,Chlamydia-
Chlamydia_trachomatis_76789547:0.00217559110076925378)100:0.13435708006937135961,Chlamydia-
Chlamydia_muridarum_15834807:0.14272277342418571155)100:0.46001705091069894360,(Chlamydia-
Chlamydophila_caviae_29840572:0.03841739282171486441,(Chlamydia-
Chlamydophila_felis_89898006:0.08001616830047572626,Chlamydia-
Chlamydophila_abortus_62185389:0.11495450980610819047)76:0.04427373394204736634)99:0.25418537735695362345)85:0
.19058786548442960274)100:0.24699119325640550571,Chlamydia-
Chlamydophila_pneumoniae_33242322:0.00000121823947223981,Chlamydia-
Chlamydophila_pneumoniae_15836487:0.00000121823947223981);

Plantae-Ostreococcus_tauri_31283
((((((Chromalveolata-Aureococcus_anophagefferens_6675:0.85203265156447327566,(Plantae-
Cyanophora_paradoxa_Contig299_5:0.57218442224666288265,(((Chlamydia-
Chlamydia_muridarum_15835243:0.04687978548083194391,(Chlamydia-
Chlamydia_trachomatis_76789075:0.00000121823947223981,Chlamydia-
Chlamydia_trachomatis_15605072:0.00000121823947223981)100:0.04482649177031228921)100:1.53198524721926387926,(E
xcavata-Trypanosoma_cruzi_71663480:0.36193474545110981833,Excavata-
Trypanosoma_brucei_74025836:0.46173491336111033290)97:0.53253713018251824796)79:0.17131890605544786155,Plantae
-
Gracilaria_changii_Contig101_3:1.02246356512522473814)29:0.05874593306059323200)72:0.14817464271407104759)18:0.1
2382927978239226252,(Chromalveolata-Isochrysis_galbana_Contig924_2:1.62338249018437474902,Plantae-
Cyanidioschyzon_merolae_CMB088C:0.15122287865648995075)62:0.28113559468836613053)14:0.05428537195469642501,(
Plantae-Physcomitrella_patens_181169:0.32766760369412328835,((Plantae-
Arabidopsis_thaliana_22328154:0.27505612098752674211,(Plantae-
Oryza_sativa_115455047:0.44120194940417073459,(Plantae-
Arabidopsis_thaliana_42568283:0.26094914338486341343,Plantae-
Oryza_sativa_115485855:0.26617594482524764565)46:0.03670945223099039006)41:0.01843419449599981164)70:0.0899633
7085552512380,Plantae-
Oryza_sativa_115451707:0.27399184956375616240)88:0.09998131379382058093)71:0.10925314915144369865)35:0.2050457
0076162234593,(Plantae-Volvox_carteri_88280_jgi:0.19110328273624857531,Plantae-
Chlamydomonas_reinhardtii_123186:0.05659878113698368907)100:0.41140691028497994308)20:0.09309560024514033916,

Chromalveolata-Phaeodactylum_tricornutum_5720:0.68832653779179286957)98:0.39666526354851938674,Plantae-
Ostreococcus_tauri_31283:0.15136325762946944984,Plantae-Ostreococcus_lucimarinus_29918:0.13829880922374471197);

Plantae-Ostreococcus_tauri_37113
(Bacteria-Thiomicrospira_crunogena_78486478:0.70001517611978636424,(Bacteria-
Desulfovibrio_vulgaris_120601318:0.59765588696877280928,((((Chlamydia-
Chlamydophila_abortus_62185185:0.08135156024156894872,(Chlamydia-
Chlamydophila_felis_89898215:0.03382340629683759647,Chlamydia-
Chlamydophila_caviae_29840354:0.03922467246425751647)62:0.01511453150795305858)100:0.15686560291286688607,(((C
hlamydia-Chlamydophila_pneumoniae_15835770:0.00000121823947223981,Chlamydia-
Chlamydophila_pneumoniae_15618159:0.00000121823947223981)20:0.00000121823947223981,Chlamydia-
Chlamydophila_pneumoniae_16752803:0.00000121823947223981)21:0.00000121823947223981,Chlamydia-
Chlamydophila_pneumoniae_33241576:0.00000121823947223981)100:0.22204366049162535535)100:0.625556712001614889
65,(((Plantae-Oryza_sativa_115465429:0.13925877668355529648,Plantae-
Arabidopsis_thaliana_30695393:0.06258397151319997076)69:0.08241921346245760505,Plantae-
Physcomitrella_patens_154565:0.18488831689583162809)92:0.22851388485651796811,(Plantae-
Ostreococcus_tauri_37113:0.36482070329131716102,Plantae-
Ostreococcus_lucimarinus_27757:0.17290245718026803212)100:1.04694272074952765550)98:0.16625564619966884394)99:0
.20303648383192782201,Bacteria-
Parabacteroides_distasonis_150007177:0.43808515576337214714)58:0.07750752973525382172)93:0.14681323984527344662,
Bacteria-Saccharophagus_degradans_90021703:0.48231988085753468853);

Plantae-Ostreococcus_tauri_8662
(Bacteria-Bradyrhizobium_sp._148257427:0.18830442920055975864,(((Bacteria-
Brucella_ovis_148559451:0.02561471497346131179,Bacteria-
Ochrobactrum_anthropi_153009066:0.03576570522629236859)100:0.41943904701710404970,Bacteria-
Parvibaculum_lavamentivorans_154252855:0.26636390271310439459)67:0.09737711252267608475,(Bacteria-
Methylobacterium_sp._149126077:0.25268511364403067887,(Bacteria-
Magnetococcus_sp._117924071:0.45772855017366781416,(((Bacteria-
Lawsonia_intracellularis_94987539:0.42399666508409711296,Bacteria-
Desulfovibrio_vulgaris_120601783:0.30741764404826976698)100:0.35016980638814920734,((((Bacteria-
Chlorobium_chlorochromatii_78188031:0.73557126098905800671,(Chlamydia-
Candidatus_Protochlamydia_46445945:0.54393669157648005807,((Plantae-
Ostreococcus_tauri_8662:0.13154848376700092882,Plantae-
Ostreococcus_lucimarinus_33240:0.10196415445367824903)100:0.34353969069628714772,((Plantae-
Physcomitrella_patens_43703:0.32744130855985609241,(Plantae-Oryza_sativa_115444103:0.24520569070820524837,Plantae-
Arabidopsis_thaliana_15238896:0.26666422791418198379)100:0.30347374071173299770)95:0.13247732322162297791,Planta
e-
Volvox_carteri_97159_jgi:0.40463708107246698642)88:0.15998729220921753535)97:0.13394748264162953388)100:0.25917
187940504876620)33:0.10005426850793520954,(Bacteria-
Carboxydothermus_hydrogenoformans_78043538:0.33461148407271290584,((Bacteria-
Symbiobacterium_thermophilum_51892339:0.32315853449993481306,((((Bacteria-
Clostridium_difficile_126700272:0.21729846120089046924,Bacteria-
Alkaliphilus_metalliredigens_150390647:0.23888295661388697644)91:0.10261740866761664970,(Bacteria-
Thermoanaerobacter_ethanolicus_114844128:0.23713079660971730922,(Bacteria-
Eubacterium_ventriosum_154485082:0.13924137754317317262,(Bacteria-
Dorea_longicatena_153854700:0.13446768708633621836,Bacteria-
Ruminococcus_obeum_153809802:0.14572820558666005342)89:0.04714852280729148193)100:0.27695895548458898672)21
:0.03189089615552404000)31:0.05584869469067176018,(Bacteria-
Caldicellulosiruptor_saccharolyticus_146295951:0.50601172060226862204,Bacteria-
Thermosinus_carboxydivorans_121535927:0.31011291640825450200)41:0.09347100199745787819)30:0.05386936612261301
188,((Bacteria-Desulfotomaculum_reducens_134298535:0.25508591430115984400,((Bacteria-
Bacillus_pumilus_157692188:0.16727017541836272652,(Bacteria-
Geobacillus_thermodenitrificans_138894646:0.21141603185405652421,(((Bacteria-
Streptococcus_thermophilus_116628390:0.16177987910699889729,Bacteria-
Lactococcus_lactis_125624479:0.16089838705167677779)100:0.13203877422120449681,Bacteria-
Staphylococcus_haemolyticus_70726737:0.27700223653455691153)42:0.05923774646800541305,(Bacteria-
Listeria_monocytogenes_153175268:0.15482300715607855146,(Bacteria-
Pediococcus_pentosaceus_116492952:0.14632060252381340337,(Bacteria-
Oenococcus_oeni_116491168:0.53031448015175175481,Bacteria-
Lactobacillus_salivarius_90962030:0.12436112504046190064)88:0.06885833851706314734)92:0.13139743584601851212)21:
0.02641755000356947602)40:0.06841300235274973274)12:0.02710771999947868618)100:0.26433442415349311938,Bacteria

-
Desulfitobacterium_hafniense_109645973:0.34780725804099604792)36:0.08356065982786746449)20:0.0699387885172292195
52,(Bacteria-Pelotomaculum_thermopropionicum_147678204:0.27807779927582926272,Bacteria-
Moorella_thermoacetica_83589686:0.45868806836417602701)34:0.08137662304285063486)0:0.05080407203568686192)5:0.0
7554162728410106220)2:0.02366744425507467942,(Bacteria-
Bacteroides_capillosus_154500741:0.42025151505049107570,(Bacteria-
Arthrobacter_sp._116670123:0.33028412767809556261,Bacteria-
Mycobacterium_ulcerans_118618804:0.48655004459776535208)100:0.38207668371068542745)8:0.03955804509511984257)1
2:0.04734604076561860914)56:0.08514658351523093915)16:0.05820284244196003570,(Bacteria-
Pelobacter_propionicus_118581697:0.25735569043375644549,Bacteria-
Geobacter_bemidjiensis_145619002:0.23474239531312826590)100:0.20738901428056030762)18:0.03927716004997061638,((
Bacteria-Syntrophus_aciditrophicus_85858517:0.49489663273416878431,(Bacteria-
Dehalococcoides_sp._147668966:0.49500915141159024246,(Bacteria-
Herpetosiphon_aurantiacus_113940985:0.46055103613187881040,Bacteria-
Roseiflexus_sp._148657877:0.28180993079869276485)72:0.05456687998023467684)71:0.07367151286678083610)20:0.04926
363861883172740,Bacteria-
Syntrophobacter_fumaroxidans_116750882:0.61445000765568658085)3:0.02458698224754185188)5:0.0369649908387999631
7)66:0.10444425758223803424,(Bacteria-Methylococcus_capsulatus_53803411:0.42431592905480391975,(Bacteria-
Nitrosococcus_oceani_77166320:0.38519544689560331063,(((Bacteria-
Oceanospirillum_sp._89095267:0.24726403065902205491,Bacteria-
Marinomonas_sp._152996635:0.28674569095837926236)100:0.15348151463868545119,(Bacteria-
Saccharophagus_degradans_90020487:0.35074055406267423685,Bacteria-
Oceanobacter_sp._94501904:0.23915904676412380603)73:0.09985932427529956024)71:0.07035300917707913715,Bacteria-
Pseudomonas_aeruginosa_107099995:0.23585054505439728700)85:0.10779609812988259765)48:0.07320996046409400382)1
00:0.23529520068582029935)47:0.03341801244317009478)100:0.30046887773932312582)25:0.04214765026090690897)100:
0.22019251949052939810,Bacteria-Rhodopseudomonas_palustris_90423689:0.17940765660077634402);

Plantae-Physcomitrella_patens_218794
(Chlamydia-Chlamydophila_pneumoniae_15835695:0.00000121823947223981,((Chlamydia-
Chlamydophila_pneumoniae_16752883:0.00000121823947223981,((((Chromalveolata-
Plasmodium_falciparum_124804314:1.20835944402427086786,(Excavata-
Euglena_gracilis_Contig2354_1:0.65776072851010347620,((Plantae-
Physcomitrella_patens_218994:0.02840593516292887893,Plantae-
Physcomitrella_patens_55781:0.07329692330673313883)100:0.13779040653901267199,(Plantae-
Oryza_sativa_115476012:0.21690635052098250490,((Plantae-Oryza_sativa_115467858:0.02719337114943972192,Plantae-
Oryza_sativa_115448277:0.05328965741988367366)80:0.03447890622746425937,(Plantae-
Arabidopsis_thaliana_15223082:0.02935045022377573420,Plantae-
Arabidopsis_thaliana_15218074:0.04970177054475031359)100:0.09478282791516762229)91:0.07498586477095083025)100:0
.12243104718999257841)100:0.25592669895784081469)95:0.10913495811223823673)39:0.10343280211110286726,((((Excav
ata-Trichomonas_vaginalis_123420894:1.21909879107291274636,(Bacteria-
Stigmatella_aurantiaca_115378537:0.61376593628079045839,((Excavata-
Jakoba_libera_Contig381_2:0.48645606345440328688,Excavata-Naegleria_gruberi_35679-
estExt_gwp_gw1.C_90072:0.43084175631161658604)40:0.18858554331968882534,Bacteria-
Thermotoga_petrophila_148269759:0.40694584305908954036)40:0.23846108349969244444)62:0.22345168435925846784)10
0:0.58008323827338348355,(((Bacteria-Rubrobacter_xylanophilus_108804902:0.65232478864045095879,(Bacteria-
Pelotomaculum_thermopropionicum_147677721:0.28811780114510043838,Bacteria-
Carboxydothermus_hydrogenoformans_78043322:0.52091615886288977766)99:0.29916111925108168634)98:0.310801525469
29201707,(Bacteria-Thermoanaerobacter_ethanolicus_114845141:0.39380387353847357002,(Bacteria-
Algoriphagus_sp._126649002:0.38232757011217027854,Bacteria-
Flavobacteria_bacterium_89891192:0.37520191820463227872)96:0.20113624446856398498)100:0.27562260361882839899)9
6:0.29922671031084568938,((Bacteria-Frankia_sp._86741530:0.24675822594304100410,Bacteria-
Kineococcus_radiotolerans_152968217:0.23659070153296457573)100:0.40526649529022990004,(Amoebozoa-
Entamoeba_histolytica_67482265:0.01351768071770072591,Amoebozoa-
Entamoeba_histolytica_67476240:0.01345232844176335110)100:0.64913031306954738220)100:0.76811374983616564904)95
:0.31585570453958761172)100:0.58457613375038830483,Plantae-
Glaucocystis_nostochinearum_Contig1177_1:0.68810681518361138664)38:0.26096913477782046620,((Chlamydia-
Candidatus_Protochlamydia_46446514:0.36774782867225425109,(Chromalveolata-
Plasmodium_falciparum_124506857:0.69936245261267904194,((Bacteria-
Candidatus_Desulfococcus_121542302:0.68379366537588248676,Bacteria-
Syntrophobacter_fumaroxidans_116750796:0.57847041543877475434)83:0.16517140621590609140,(((Chlamydia-
Chlamydophila_felis_89898202:0.03178775445954938605,(Chlamydia-
Chlamydophila_abortus_62185197:0.02137145226229079931,Chlamydia-

Chlamydophila_caviae_29840366:0.02299196847978217037)89:0.01337380207862569684)100:0.07461998652903611973,((C
hlamydia-Chlamydia_trachomatis_15604925:0.00000121823947223981,Chlamydia-
Chlamydia_trachomatis_76788927:0.00431462692513677384)99:0.04367052235165817808,Chlamydia-
Chlamydia_muridarum_15835095:0.05127556433343457426)100:0.17623073118569523854)99:0.06843159391452642570,(Ch
lamydia-Chlamydophila_pneumoniae_15835743:0.00000121823947223981,(Chlamydia-
Chlamydophila_pneumoniae_16752833:0.00000121823947223981,(Chlamydia-
Chlamydophila_pneumoniae_33241547:0.00000121823947223981,Chlamydia-
Chlamydophila_pneumoniae_15618132:0.00000121823947223981)17:0.00000121823947223981)14:0.00000121823947223981
)100:0.11696917454467022079)100:0.50340852439963656995)15:0.03727220110849934626)10:0.03492382689511989297)2:
0.04779708589352683890,((((Excavata-Euglena_gracilis_109786964_3:0.00000121823947223981,Excavata-
Euglena_gracilis_109785016_3:0.00090564044648994819)73:0.42792717051502787706,Chromalveolata-
Paramecium_tetraurelia_124429824:0.32376913961006664744)47:0.13008438762506582820,((((Plantae-
Porphyra_yezoensis_Contig890_1:0.19224285512545297161,Plantae-
Cyanidioschyzon_merolae_CMH052C:0.33147141815533232023)43:0.08972070520682014216,(Plantae-
Galdieria_sulphuraria_Contig417_1:0.12946655498172079946,Plantae-
Galdieria_sulphuraria_Contig132_3:0.17244675509608620168)98:0.10798556065223262335)38:0.05402253198857343008,Pla
ntae-Porphyra_yezoensis_AV434269_2:0.26013547243401208275)81:0.10558727632981135258,((Plantae-
Oryza_sativa_115467370:0.07175210634280602962,(Plantae-
Arabidopsis_thaliana_15221156:0.07384059582093772944,Plantae-
Arabidopsis_thaliana_30679628:0.08382321595916689128)77:0.03884561183910403837)93:0.07607022823313240589,(Planta
e-Physcomitrella_patens_188815:0.09286380994341331130,(Plantae-
Physcomitrella_patens_107676:0.06041498389452922696,Plantae-
Physcomitrella_patens_218794:0.02214676552498514445)99:0.07113571985159253264)99:0.09551418830081596922)99:0.28
378067995053007921)15:0.06461995072742353352)6:0.04378403467608589505,((Plantae-
Gracilaria_changii_120463673_3:0.63268836420419916067,(Excavata-
Giardia_lamblia_159114566:0.00000121823947223981,Excavata-
Giardia_lamblia_157435613:0.00892185987353322912)100:0.47697965499317424021)53:0.09941846340149131100,(Rhizaria
-Reticulomyxa_filosa_EE664370_3:0.94057023501206393767,((Amoebozoa-
Entamoeba_histolytica_67474879:0.37863747550124970953,(Bacteria-
Parabacteroides_distasonis_150008153:0.08643206896319155508,Bacteria-
Bacteroides_vulgatus_150004823:0.06574193839386427429)100:0.19999722323774454802)57:0.07282860170087231133,(Bac
teria-Treponema_denticola_42527058:0.23801612506535121949,Bacteria-
Borrelia_garinii_51598283:0.38541136189984648297)27:0.05860673777964019865)49:0.05808589775835382857)9:0.0279911
5924378565295)5:0.05149992633840592277)7:0.03692221734310761200)2:0.02824074580174385993)10:0.032410122343325
33298)100:0.58518529528823415298,(Chlamydia-Chlamydia_muridarum_15835097:0.05409221692176190144,(Chlamydia-
Chlamydia_trachomatis_15604927:0.00200440026545794028,Chlamydia-
Chlamydia_trachomatis_76788929:0.01086427552752633127)100:0.04111449695160136059)100:0.13427136625720703655)6
8:0.09014046601054108432,(Chlamydia-Chlamydophila_abortus_62185199:0.03713148852215986812,(Chlamydia-
Chlamydophila_caviae_29840368:0.01879302003410709965,Chlamydia-
Chlamydophila_felis_89898200:0.03911749406847455507)73:0.02109761020470437773)99:0.07997213384312837348)100:0.1
3618133009352834106)10:0.00000121823947223981,Chlamydia-
Chlamydophila_pneumoniae_15618084:0.00000121823947223981)12:0.00000121823947223981,Chlamydia-
Chlamydophila_pneumoniae_33241496:0.00000121823947223981);

Plantae-Physcomitrella_patens_219372

(((((Bacteria-Petrotoga_mobilis_145621604:0.47080870913021899415,((Bacteria-
Alkaliphilus_metalliredigens_150388901:0.33136722162371573530,Bacteria-
Thermoanaerobacter_ethanolicus_114843849:0.42937605994712368940)45:0.04886530893105391071,Bacteria-
Clostridium_thermocellum_125973390:0.32378853665551993180)57:0.07799405634370656926)46:0.10186032292350613582,
(((Cyanobacteria-Cyanothece_sp._CCY0110_126656374:0.73276719849006766161,Bacteria-
Syntrophomonas_wolfei_114565915:0.85506296731785436105)16:0.16592627356213923706,(((Bacteria-
Arthrobacter_sp._116670755:0.33596287196402596642,(Bacteria-
Acidothermus_cellulolyticus_117928174:0.35967432572744512109,(((Bacteria-
Saccharopolyspora_erythraea_134098264:0.32273026689605044393,(Bacteria-
Mycobacterium_gilvum_145223544:0.40366731801884259134,Bacteria-
Rhodococcus_sp._111018141:0.25842538617452576188)88:0.10344473808743326337)70:0.06394708156102459073,Bacteria-
Salinispora_tropica_145595813:0.35664351644668390584)52:0.06438354119760374139,Bacteria-
Thermobifida_fusca_72161428:0.34986782986341846691)41:0.06780411571231807766)41:0.05754177901229842740)100:0.4
2971231754804406489,(((Chromalveolata-Phaeodactylum_tricornutum_14230:0.00000121823947223981,Chromalveolata-
Phaeodactylum_tricornutum_28937:0.00000121823947223981)100:0.45306915673459041694,Chromalveolata-
Aureococcus_anophagefferens_31792:0.60135019330328287612)97:0.24464953650458670653,((Chromalveolata-
Phytophthora_sojae_120969:0.50158102343309141080,Plantae-

Cyanidioschyzon_merolae_CMC098C:0.60133069957033247199)26:0.06249365812189198316,((Chlamydia-
Chlamydophila_caviae_29840321:0.06801771111648878543,Chlamydia-
Chlamydophila_felis_89898246:0.04053847275349523743)100:0.73124373272629517029,((Plantae-
Chlamydomonas_reinhardtii_126030:0.42401980434019914679,(Plantae-
Ostreococcus_lucimarinus_32105:0.04068163113361368544,Plantae-
Ostreococcus_tauri_33342:0.11304616415661168649)100:0.28261777627430251236)94:0.13082207764897643698,(Plantae-
Physcomitrella_patens_219372:0.28184505315604718723,(Plantae-
Oryza_sativa_115450443:0.24378971550594233375,Plantae-
Arabidopsis_thaliana_15238711:0.16837830637349712748)100:0.18581728143543477239)100:0.17390926224877437112)92:0
.10668094854224084711)58:0.06639305019106879480)23:0.02896964424602786747)93:0.11620728603184846128)73:0.1062
6995357206808768,Bacteria-
Shewanella_sp._114047031:0.86662592277759198378)30:0.06533308603515051993)13:0.05707253451286126850,(((((Bacteri
a-Carboxydothermus_hydrogenoformans_78044554:0.51735977806399424672,Bacteria-
Desulfotomaculum_reducens_134298128:0.41685511518010054388)78:0.13143074670462032150,Bacteria-
Moorella_thermoacetica_83590187:0.43936435871349066584)9:0.03359904590045203338,Bacteria-
Acidobacteria_bacterium_94970911:0.53117942057480116436)2:0.06420551922937715872,(((Bacteria-
Chloroflexus_aggregans_118046823:0.18491966502615692081,Bacteria-
Roseiflexus_castenholzii_156742126:0.24431533106114050602)100:0.23492283901147886782,Bacteria-
Dehalococcoides_sp._147669912:0.48561669145712410556)72:0.08323466787098347808,Bacteria-
Symbiobacterium_thermophilum_51892547:0.39245185586495490160)18:0.09012422512172765798)3:0.04026089729810835
433,((Bacteria-Rubrobacter_xylanophilus_108804914:0.46311139170362386475,((Bacteria-
Ralstonia_solanacearum_17548902:0.46063126704871920625,((Bacteria-
Xanthobacter_autotrophicus_154248296:0.25268861677939202792,(Bacteria-
Rhodopseudomonas_palustris_86749914:0.11594418726660288277,(Bacteria-
Nitrobacter_hamburgensis_92117280:0.11838009034551030818,Bacteria-
Bradyrhizobium_japonicum_27379920:0.10787106195460544056)74:0.02855322647826223983)97:0.09679076206867751053)
94:0.09677871407237413626,((Bacteria-Methylobacterium_extorquens_153900379:0.42935063038892323783,(Bacteria-
Agrobacterium_tumefaciens_15888992:0.20158693509073680183,Bacteria-
Sinorhizobium_medicae_150396622:0.20170486659404524010)100:0.09143829508904564862)62:0.05672414945021995308,((
Bacteria-Aurantimonas_sp._90419612:0.22091240792819263894,Bacteria-
Fulvimarina_pelagi_114707420:0.28999131710553882613)100:0.15935837766105553914,Bacteria-
Mesorhizobium_sp._110633990:0.20149858572196943651)59:0.04188630841425510648)44:0.04923298417737447669)29:0.0
4150368834278563707)95:0.10615686042835803526,(Bacteria-
Parvibaculum_lavamentivorans_154253611:0.31393388571211999771,(Bacteria-
Rhodospirillum_rubrum_83593231:0.22695650133258643555,Bacteria-
Magnetospirillum_magneticum_83311961:0.36450625661345542605)62:0.09520647672159243680)80:0.056887193832533641
81)95:0.17303260017947899585)74:0.11422712452061327404,Bacteria-
Anaeromyxobacter_dehalogenans_86160470:0.49271849067448358905)16:0.06151128011113523447)6:0.08054447965872281
456)11:0.02560687683212867563)99:0.20036795554115127693,(Bacteria-
Chromobacterium_violaceum_34497628:0.25708652967999151562,((((Bacteria-
Azoarcus_sp._56477814:0.19468916297222446654,Bacteria-
Dechloromonas_aromatica_71909088:0.13322511547766335838)92:0.06561558584975797637,(Bacteria-
Stenotrophomonas_maltophilia_119878293:0.14673042387419793497,Bacteria-
Xylella_fastidiosa_28198103:0.28659017609244030744)100:0.13839562410160477501)86:0.04732712538467952201,Bacteria-
Methylibium_petroleiphilum_124268645:0.25467725510858563176)99:0.12191393940102766835,Bacteria-
Neisseria_meningitidis_121634768:0.34946453281855860418)46:0.03272657979135049772)100:0.21681828293120444795)97
:0.09788012121410423483,Bacteria-
Thiomicrospira_crunogena_78484613:0.42697954869079879359)59:0.05894053974653503530,((Bacteria-
Beggiatoa_sp._153870024:0.27461044045871862984,(Bacteria-
Nitrosococcus_oceani_77165954:0.25851843138599511773,Bacteria-
Methylococcus_capsulatus_53803265:0.33781353390532503633)49:0.07104307861950788505)63:0.08369653801250236114,B
acteria-Alkalilimnicola_ehrlichei_114321397:0.36129045173261215007)37:0.06602939417398331901,Bacteria-
Candidatus_Ruthia_118602164:0.55985667412328687931);

Plantae-Physcomitrella_patens_55802

(((Chlamydia-Chlamydophila_pneumoniae_33241718:0.00000121823947223981,Chlamydia-
Chlamydophila_pneumoniae_15618288:0.00000121823947223981)22:0.00000121823947223981,((Chlamydia-
Chlamydophila_abortus_62185038:0.05239000026607414096,(Chlamydia-
Chlamydophila_caviae_29840185:0.03696886539815637251,Chlamydia-
Chlamydophila_felis_89898391:0.05115358339316190134)75:0.02299821135682846018)96:0.05276988733583318908,((Chla
mydia-Chlamydia_muridarum_15834947:0.08807184671693175926,(Chlamydia-
Chlamydia_trachomatis_15604776:0.00000121823947223981,Chlamydia-

Chlamydia_trachomatis_76788770:0.00000121823947223981)100:0.11296171593422218993)100:0.12473427301168686643,((
Chlamydia-Candidatus_Protochlamydia_46446374:0.22676148576007132407,((((Plantae-
Arabidopsis_thaliana_30697397:0.00000121823947223981,Plantae-
Arabidopsis_thaliana_30697395:0.00000121823947223981)100:0.03974465016138740980,Plantae-
Oryza_sativa_115447171:0.06029029023595906811)98:0.07216927413880408482,((Plantae-
Physcomitrella_patens_55802:0.00584754315397417995,Plantae-
Physcomitrella_patens_233105:0.02793994918039225533)70:0.01981462798426785951,Plantae-
Physcomitrella_patens_130936:0.06004041858988539565)89:0.04182949962624766399)97:0.08297689164305356579,(((((Rhi
zaria-Bigelowiella_natans_Contig164_3:1.28024526102074909240,Chromalveolata-
Isochrysis_galbana_ISE00009753_2:1.99867693247502797860)36:0.08529732505760778682,Chromalveolata-
Emiliania_huxleyi_UI-EH-HG1-aae-c-17-0-
UI.s1_4:0.00000121823947223981)35:0.28426151688126277683,((Chromalveolata-
Aureococcus_anophagefferens_71112:0.14497251919294062983,(Chromalveolata-
Thalassiosira_pseudonana_29228:0.09262475555184658538,Chromalveolata-
Phaeodactylum_tricornutum_44955:0.04155527940142136195)100:0.15410545031534197968)59:0.06450122377697323217,C
hromalveolata-
Guillardia_theta_88770650:0.10718946621869424041)25:0.03063895482300561801)43:0.06807606955963736717,((Chromalv
eolata-Plasmodium_falciparum_124802547:0.67528966108900168042,Plantae-
Chlamydomonas_reinhardtii_55268:0.00802574977597945473)44:0.03541914679832490476,Plantae-
Volvox_carteri_63610_jgi:0.03339180168904673507)60:0.10415385210792293957)35:0.04154506274941901361,(Plantae-
Ostreococcus_lucimarinus_12863:0.03292112463491071728,Plantae-
Ostreococcus_tauri_19063:0.02836769076305388146)100:0.16600107222599339019)42:0.06934442046947635052)68:0.17728
566990944727433)73:0.07194578433481391144,((((Bacteria-
Bacteroides_thetaiotaomicron_29347927:0.24398854700204575252,Bacteria-
Parabacteroides_distasonis_150009758:0.15513074071193816428)100:0.26116619335154644954,((Bacteria-
Cytophaga_hutchinsonii_110638588:0.10648403841196653341,(Bacteria-
Microscilla_marina_124008572:0.14325248033658002922,Bacteria-
Algoriphagus_sp._126647633:0.12016732319960958530)55:0.04631796945774341312)61:0.07053321981051641210,Bacteria-
Pedobacter_sp._149278639:0.16471178954300738639)93:0.07125851122093652956)98:0.11511436831950944137,(Bacteria-
Leptospira_interrogans_24215859:0.31437624421081450388,(Bacteria-
Chlorobium_chlorochromatii_78188327:0.06122127519179303839,(Bacteria-
Prosthecochloris_vibrioformis_145220416:0.08935965600383269369,Bacteria-
Pelodictyon_luteolum_78187812:0.07967571668026340870)96:0.05145623597652759784)100:0.42660866932889335290)62:0.
07482942926906087366)83:0.09174590141764177309,((((Plantae-
Chondrus_crispus_62994624_2:0.15014798525792133477,Plantae-
Galdieria_sulphuraria_Contig443_1:0.13374503147374863787)48:0.04229841894512781364,(Plantae-
Cyanidioschyzon_merolae_CML284C:0.04902865835267034705,Plantae-
Porphyra_yezoensis_Contig1839_3:0.24326050301007931531)82:0.04485182340421266928)80:0.05967786270720680519,(((((
(Plantae-Porphyra_yezoensis_CX874586_3:0.55276864258209679193,Cyanobacteria-Synechococcus_sp._JA-3-
3Ab_86607013:0.00000121823947223981)31:0.03710182328882509484,Cyanobacteria-Synechococcus_sp._JA-2-3Ba2-
13_86607623:0.00812731792465570466)34:0.20249402386640871776,(((((((Cyanobacteria-
Prochlorococcus_marinus_str._MIT_9515_123965984:0.01250082833838241266,Cyanobacteria-
Prochlorococcus_marinus_subsp._pastoris_str._CCMP1986_33861233:0.01566136011441973674)56:0.01391098066796059296
,(Cyanobacteria-Prochlorococcus_marinus_str._AS9601_123968266:0.00000121823947223981,(Cyanobacteria-
Prochlorococcus_marinus_str._MIT_9312_78779061:0.00616314125394170752,Cyanobacteria-
Prochlorococcus_marinus_str._MIT_9301_126696067:0.00308172240920981649)41:0.00305644013494659351)78:0.01495435
445206058818)100:0.16546408230142201079,((Cyanobacteria-
Prochlorococcus_marinus_str._NATL1A_124025441:0.00000121823947223981,Cyanobacteria-
Prochlorococcus_marinus_str._NATL2A_72381949:0.00000121823947223981)100:0.10667979011433369185,(Cyanobacteria-
Prochlorococcus_marinus_subsp._marinus_str._CCMP1375_33240465:0.06120215857275387167,Cyanobacteria-
Prochlorococcus_marinus_str._MIT_9211_84518151:0.05006621250687218194)89:0.04312445380271118928)89:0.058066011
69736672063)90:0.08095861998657805092,(Cyanobacteria-
Prochlorococcus_marinus_str._MIT_9303_124023138:0.01509119081724520813,Cyanobacteria-
Prochlorococcus_marinus_str._MIT_9313_33863049:0.01404774731413222302)100:0.05686037925711446422)73:0.04206371
620788174059,(((Cyanobacteria-Synechococcus_sp._WH_8102_33865708:0.05118547793700545939,Cyanobacteria-
Synechococcus_sp._CC9605_78212828:0.03967866079753849112)66:0.01583802362734687899,(Cyanobacteria-
Synechococcus_sp._BL107_116070627:0.00256447041523655186,Cyanobacteria-
Synechococcus_sp._CC9902_78184752:0.00380965020643024696)100:0.09134205605714458553)66:0.02023619526324405335
2,((Cyanobacteria-Synechococcus_sp._RS9917_87125792:0.02898757971199794614,(Cyanobacteria-
Synechococcus_sp._RS9916_116074926:0.03765756303312582653,(Cyanobacteria-
Synechococcus_sp._WH_7803_148239811:0.00962364140228628463,Cyanobacteria-
Synechococcus_sp._WH_7805_88808860:0.00958567824122011011)100:0.03804487923913215525)57:0.01011719663113377

736)67:0.01438574443075633924,Cyanobacteria-
Synechococcus_sp._CC9311_113952754:0.08134213261532743933)89:0.04103910203926392636)97:0.0716976918705666682
6)51:0.05426372847852895670,Cyanobacteria-
Synechococcus_sp._WH_5701_87302162:0.05885200921940707958)32:0.02763601470892435366,Cyanobacteria-
Synechococcus_sp._RCC307_148242561:0.06879003938813696428)82:0.07652782980612948815)12:0.056646067333917173
03,(Cyanobacteria-Synechococcus_elongatus_PCC_6301_56750826:0.00000121823947223981,Cyanobacteria-
Synechococcus_elongatus_PCC_7942_81299524:0.00000121823947223981)100:0.08899761277099861589)8:0.039771860609
50454322,(Cyanobacteria-Lyngbya_sp._PCC_8106_119489558:0.05994154545055321859,((Cyanobacteria-
Synechocystis_sp._PCC_6803_16330309:0.08195210875746701540,((Cyanobacteria-
Nodularia_spumigena_CCY9414_119510040:0.04496207129685535903,Cyanobacteria-
Nostoc_punctiforme_PCC_73102_23128723:0.03089236527865393128)54:0.00584873781124384869,(Cyanobacteria-
Anabaena_variabilis_ATCC_29413_75906658:0.00627656658702608651,Cyanobacteria-
Nostoc_sp._PCC_7120_17229993:0.00000121823947223981)99:0.03031954302944691801)94:0.04343035746147681786)24:0.
01259578267913413936,((Cyanobacteria-
Trichodesmium_erythraeum_IMS101_113477916:0.08433993170096726333,(Cyanobacteria-
Cyanothece_sp._CCY0110_126656414:0.02665380352224306845,Cyanobacteria-
Crocosphaera_watsonii_WH_8501_67925456:0.04181001157079160258)78:0.01699646392881423024)46:0.007340398990700
23684,(Cyanobacteria-Thermosynechococcus_elongatus_BP-1_22298539:0.13296427055483051038,Plantae-
Glaucocystis_nostochinearum_106806523_3:0.21484897294212820795)8:0.02278869229711502284)16:0.01725093441606295
394)8:0.01330336690769419461)21:0.03231208234013252523)8:0.04510309826262785121,Cyanobacteria-
Gloeobacter_violaceus_PCC_7421_37523191:0.32644642363021497689)34:0.03789604543091650707)100:0.36583774757352
716112,((Bacteria-Syntrophomonas_wolfei_114566426:0.47930449432777105789,(Bacteria-
Clostridium_perfringens_110803791:0.38611656778970793180,((Bacteria-
Parvibaculum_lavamentivorans_154252198:0.09800260921238615475,(Bacteria-
Caulobacter_crescentus_16125104:0.16829421847993322747,Bacteria-
Rhodospirillum_rubrum_83592086:0.22283747430117328836)59:0.07305285106755315949)100:0.61427829601914663016,(B
acteria-Salinibacter_ruber_83815534:0.70865835973119362112,(Bacteria-
Acidothermus_cellulolyticus_117928729:0.10035909574907100894,(((Bacteria-
Corynebacterium_jeikeium_68536242:0.17847770177032737382,(Bacteria-
Saccharopolyspora_erythraea_134102436:0.08541974494495729875,((Bacteria-
Clavibacter_michiganensis_148273340:0.19809174495862333365,Bacteria-
Kineococcus_radiotolerans_152965397:0.07233099368914318317)98:0.07692758160738523421,Bacteria-
Nocardioides_sp._119717426:0.05972321805337812289)84:0.07261754943346392099)51:0.04421643043318894406)59:0.055
82328987299336326,Bacteria-
Streptomyces_avermitilis_29829103:0.10249089585270312885)21:0.04120568978160127349,Bacteria-
Frankia_sp._86742254:0.14942232680067296657)47:0.05414361490938043864)100:0.36634761232474905013)60:0.12368297
031067951308)89:0.24105367977760630560)39:0.06880322623564624762)90:0.24206116233489449741,(Bacteria-
Thermus_thermophilus_46199979:0.35523229935275735247,((Bacteria-
Deinococcus_radiodurans_15805413:0.33268032123583574089,(Bacteria-
Acidobacteria_bacterium_94968451:0.21320640643030258632,Bacteria-
Solibacter_usitatus_116620316:0.23075896407654153397)99:0.14991459622353411896)59:0.06529353560589995498,(Bacteri
a-Francisella_tularensis_134302085:0.37305656666703118640,((((Bacteria-
Halorhodospira_halophila_121996941:0.32916240791267409316,Bacteria-
Alkalilimnicola_ehrlichei_114320617:0.17289104546851008504)94:0.09313922605434513113,((Bacteria-
Ralstonia_eutropha_73541774:0.19053153100629635630,(((Bacteria-
Comamonas_testosteroni_118051079:0.07553910602596915214,Bacteria-
Delftia_acidovorans_118729199:0.05351643322072237308)93:0.07154453282096211175,Bacteria-
Acidovorax_avenae_120610111:0.06168828848110684965)72:0.04785467901566505672,(Bacteria-
Methylibium_petroleiphilum_124267185:0.19706023807544190141,(Bacteria-
Polaromonas_sp._91788470:0.05439872155840635992,Bacteria-
Rhodoferax_ferrireducens_89901085:0.10308089234428824410)86:0.05781184976886138277)53:0.02487896382498836281)1
00:0.17004964986852710851)97:0.10794938925613041525,(Bacteria-
Azoarcus_sp._56476110:0.19148131511110383363,(Bacteria-
Chromobacterium_violaceum_34498993:0.19371086162575570921,(Bacteria-
Methylophilales_bacterium_118594984:0.46266494375756445079,Bacteria-
Methylobacillus_flagellatus_91775973:0.14952763128249094149)57:0.08163487604207864123)85:0.12897241184964361449)
22:0.06275621247226215171)12:0.03412026727142673105)22:0.04952418507905636397,((Bacteria-
Bradyrhizobium_sp._146337679:0.05448555849139453955,(Bacteria-
Rhodopseudomonas_palustris_115522212:0.09725248835324525054,Bacteria-
Nitrobacter_hamburgensis_92116239:0.06117003280112628949)68:0.05872362846406336406)100:0.17802055617071640969,(
((Bacteria-Aurantimonas_sp._90420294:0.08543272809972661530,Bacteria-
Fulvimarina_pelagi_114707340:0.08435395817842195054)73:0.04440178028079173889,Bacteria-

Brucella_ovis_148559689:0.1567159941906279274б)93:0.07690714764316959606,Bacteria-
Stappia_aggregata_118592124:0.18055017456633745687)57:0.03806396639497909573)100:0.20754487257301634795)28:0.03
743112850457501473,Bacteria-
Xanthomonas_oryzae_84623752:0.32749671102976551929)42:0.07191190564878416347)70:0.09481166841912302967)74:0.1
5350636879076348662)100:1.05262594329142000760)100:0.39232137832954033785)95:0.19276675500180476264)77:0.1291
8754697508413876)100:0.35557133979250582501)100:0.09742090448055749918)100:0.13687897283332373100)11:0.000001
21823947223981,Chlamydia-Chlamydophila_pneumoniae_15835906:0.0000121823947223981,Chlamydia-
Chlamydophila_pneumoniae_16752666:0.0000121823947223981);

Plantae-Physcomitrella_patens_60901

((Plantae-Arabidopsis_thaliana_15222822:0.23142233338865131342,Plantae-
Arabidopsis_thaliana_18402254:0.01620634124040129867)90:0.08128232690912072511,((Plantae-
Physcomitrella_patens_60901:0.06046854264305581705,Plantae-
Physcomitrella_patens_170499:0.07254883692066838030)100:0.14921903133351160919,(((((Bacteria-
Plesiocystis_pacifica_149917403:0.52078135688396576253,((((Chlamydia-
Chlamydophila_pneumoniae_33242384:0.0000121823947223981,Chlamydia-
Chlamydophila_pneumoniae_16752011:0.0000121823947223981)24:0.0000121823947223981,Chlamydia-
Chlamydophila_pneumoniae_15618923:0.0000121823947223981)17:0.0000121823947223981,Chlamydia-
Chlamydophila_pneumoniae_15836546:0.0000121823947223981)100:0.06982370958396429128,(((Chlamydia-
Chlamydophila_abortus_62185326:0.02218172281094602308,Chlamydia-
Chlamydophila_caviae_29840503:0.01852928829827963503)37:0.01648025683109773445,Chlamydia-
Chlamydophila_felis_89898077:0.02514162191140028105)81:0.06980836231929707369,(Chlamydia-
Chlamydia_muridarum_15834867:0.04758188609304316030,(Chlamydia-
Chlamydia_trachomatis_15605593:0.00597251998002922002,Chlamydia-
Chlamydia_trachomatis_76789604:0.00271110414428906224)100:0.05300098812056019265)100:0.26241623284546033990)7
4:0.05853940996475638647)100:0.75440197629244087896)34:0.09402793589752059256,((Bacteria-
Rhodopseudomonas_palustris_90423709:0.21172651499493877481,(Bacteria-
Bradyrhizobium_sp._148254401:0.25592518079139153775,(Bacteria-
Dechloromonas_aromatica_71906322:0.16637200869672816306,(Bacteria-
Geobacter_uraniumreducens_148264829:0.15811359524152615585,Bacteria-
Rhodoferax_ferrireducens_89900927:0.22572115927195515739)77:0.05509046125766407093)60:0.07494261641009009045)4
7:0.09764239563655169318)100:0.26866463771010712369,((Bacteria-
unidentified_eubacterium_149370019:0.58817965409751471473,Bacteria-
Flavobacteria_bacterium_126663904:0.72642196109272394455)43:0.12721854117099759551,(Bacteria-
Pseudomonas_mendocina_146308547:0.38940560144198116399,((((Bacteria-
Mycobacterium_ulcerans_118618650:0.81579857765893770694,Bacteria-
Rubrobacter_xylanophilus_108803863:0.64413247571358211996)99:0.49476582419196768470,Bacteria-
Fervidobacterium_nodosum_154249049:1.07415314010596651961)100:1.86373903069026858326,(((Bacteria-
Sulfurovum_sp._152993481:0.18008523330296752341,((Bacteria-
Campylobacter_concisus_157165221:0.23780591160080036262,(Bacteria-
Nitratiruptor_sp._152991083:0.15162927167301420983,Bacteria-
Caminibacter_mediatlanticus_149194571:0.16498175423097793946)100:0.19377067982250439915)61:0.072009707549243046
07,(Bacteria-Thiomicrospira_denitrificans_78776622:0.26115820891636043477,Bacteria-
Psychromonas_ingrahamii_119946307:0.33335524600082300228)32:0.04288630200688065691)76:0.09797273144468586492)
100:0.43597171430833697725,((Bacteria-Saccharophagus_degradans_90019714:0.18360882134675485244,Bacteria-
alpha_proteobacterium_114771503:0.20636317830638964410)79:0.03806969631559006806,(((Bacteria-
Nitrosococcus_oceani_77164983:0.25859812950835914247,Bacteria-
Mariprofundus_ferrooxydans_114778417:0.38277744054823764630)48:0.06717051200601126260,((Bacteria-
Rhodobacter_sphaeroides_126463418:0.40353301037019079223,Bacteria-
Francisella_tularensis_134301219:0.55521179395924968869)46:0.11022693786554209339,((Bacteria-
Desulfuromonas_acetoxidans_95929830:0.15801686392851263530,Bacteria-
Desulfotalea_psychrophila_51244700:0.19242112742750447363)95:0.08487239187584472155,Bacteria-
Pelobacter_carbinolicus_77917729:0.18951716625967168772)93:0.10357132011443084818)11:0.04695171584165250267)25:0
.07256989183877086969,((Bacteria-Marinobacter_sp._126668979:0.29262709256409552561,(((Bacteria-
Photobacterium_profundum_90412592:0.11580654197701611974,(Bacteria-
Vibrio_vulnificus_27367593:0.19764837055533107657,Bacteria-
Vibrionales_bacterium_148975285:0.09937258689203960771)68:0.05424571433287446454)100:0.17846759829562183763,(B
acteria-Moritella_sp._149907881:0.23586580417886063299,Bacteria-
Aeromonas_salmonicida_145299494:0.25109792080633019928)31:0.04965705237098005237)81:0.10078206526612015770)8
7:0.08806719720479307123,(Bacteria-Magnetococcus_sp._117926716:0.54069991196257227717,(Bacteria-
Candidatus_Ruthia_118602368:0.30633254273367965803,Bacteria-
Methylococcus_capsulatus_53803617:0.47180509863254732617)61:0.07506912086012415553)35:0.03316750018899106839)3

8:0.07431547884652829616)47:0.11528380396218590453)98:0.44913452739175291351)99:0.57720738910625790652,((Bacteria-Alcanivorax_borkumensis_110833099:0.43690115645288041257,Bacteria-
Oceanospirillum_sp._89093844:0.45314917059880555072)33:0.05854269300200089232,((Bacteria-
Pseudoalteromonas_tunicata_88861303:0.18152492069778863870,Bacteria-
Alteromonadales_bacterium_119472888:0.14790947472480278635)99:0.25299387284841912127,((Bacteria-
Oceanobacter_sp._94500402:0.31058174795745491226,Bacteria-
Hahella_chejuensis_83648420:0.40864672473988283841)96:0.17485780148637497522,Bacteria-
Shewanella_woodyi_118074216:0.69868604719402860326)56:0.12118324503546314130)20:0.05254801210367582992)100:0.98843381831793353243)62:0.23460353379395398887)100:0.72538875694468751831,Bacteria-
Parabacteroides_merdae_154494200:0.47938128528617435231)36:0.22049177062390062920)24:0.12542790638772305378)16:0.06862118514759218968)73:0.20448919390438277666)96:0.21891083941495606546,Plantae-
Cyanidioschyzon_merolae_CMJ093C:0.53795031639902390896)21:0.04307158226877681045,(Plantae-
Porphyra_yezoensis_AV438891_3:0.22120412878565220982,(Chromalveolata-
Thalassiosira_pseudonana_15027:0.08806474120732808719,Chromalveolata-
Phaeodactylum_tricornutum_42116:0.15139222668502144709)100:0.50488275740176924433)18:0.06189309212395430887)52:0.10209258817549647647,(Plantae-Volvox_carteri_57334_jgi:0.13366023487563716277,Plantae-
Chlamydomonas_reinhardtii_103745:0.12892680209170481742)100:0.56041094862438167734)89:0.14898779425530545018)87:0.07247589695393578946,Plantae-Oryza_sativa_115477946:0.22495630017219797603);

Plantae-Physcomitrella_patens_70762
((Chlamydia-Chlamydia_trachomatis_76789589:0.00712375847736827078,Chlamydia-
Chlamydia_trachomatis_15605579:0.00713435697748557567)99:0.02312890158673753388,((((Chlamydia-
Chlamydophila_pneumoniae_15618909:0.00000121823947223981,Chlamydia-
Chlamydophila_pneumoniae_16752024:0.00000121823947223981)100:0.00000121823947223981,(Chlamydia-
Chlamydophila_pneumoniae_15836532:0.00000121823947223981,Chlamydia-
Chlamydophila_pneumoniae_33242370:0.00000121823947223981)99:0.00000121823947223981)100:0.17078773887528556075,(Chlamydia-Chlamydophila_felis_89898061:0.03692771137155841099,(Chlamydia-
Chlamydophila_abortus_62185341:0.05522878766033753606,Chlamydia-
Chlamydophila_caviae_29840517:0.01569340449069358753)51:0.01133399993926952759)93:0.06870655664877810265)89:0.07858340560397150676,((((Plantae-Oryza_sativa_115468148:0.18514679251026577855,Plantae-
Arabidopsis_thaliana_15221490:0.09654230636952099154)94:0.25278399867105877385,Plantae-
Physcomitrella_patens_70762:0.32389702985771628985)61:0.12854420680518033326,((((Chromalveolata-
Thalassiosira_pseudonana_34714:0.43912264784414084540,Chromalveolata-
Phaeodactylum_tricornutum_7195:0.45098376669533435379)100:0.34020012204498412123,Plantae-
Galdieria_sulphuraria_A4_35D03_1:0.62093960598232300629)79:0.17996567501071641937,((((Bacteria-
Syntrophus_aciditrophicus_85857921:0.43578269835554106493,((Bacteria-
Dichelobacter_nodosus_146329465:0.66655483986009167463,Bacteria-
Coxiella_burnetii_153207852:0.37520210449481083970)26:0.11680035322764795169,Bacteria-
Maricaulis_maris_114569170:0.64390440370043366958)1:0.00277726949896890934)10:0.06183941707287197997,((((Bacteria-Ralstonia_metallidurans_94310515:0.35135671021306380535,(Bacteria-
Chromobacterium_violaceum_34498926:0.32209790827484402476,Bacteria-
Burkholderia_phymatum_118032682:0.36387275326833495859)37:0.08619979796075852296)43:0.05844632553898954780,Bacteria-Polynucleobacter_sp._145589597:0.33262785332709560171)30:0.07500127282399812745,(Bacteria-
Polaromonas_sp._91788312:0.28142203428306900026,((Bacteria-
Verminephrobacter_eiseniae_121609574:0.20426100564648888569,(Bacteria-
Delftia_acidovorans_118729403:0.21774763396721100173,Bacteria-
Comamonas_testosteroni_118050608:0.28890330941236486151)83:0.19362587636001352132)27:0.04759214895472174689,Bacteria-
Acidovorax_sp._121594624:0.16190054544784085455)20:0.03905286712168044155)29:0.09413641613880813219)53:0.07389922616567703995,(((Bacteria-Methylococcus_capsulatus_53802428:0.33619192050595508858,Bacteria-
Candidatus_Ruthia_118602884:0.74149857180890932362)13:0.00000121823947223981,((Bacteria-
Haemophilus_ducreyi_33152629:0.47274714152181657179,(Bacteria-
Thiomicrospira_crunogena_78485955:0.39541773062136498229,((Bacteria-
Vibrionales_bacterium_148981936:0.14003863131422081278,Bacteria-
Vibrio_shilonii_149190045:0.07266500702686264079)99:0.32370255544198839326,Bacteria-
Shewanella_sediminis_157376253:0.35860995512896604032)48:0.06098995635875383015)42:0.06842478832652129639)75:0.14659695072940498450,Bacteria-
Nitrosococcus_oceani_77166061:0.37696047723917197603)17:0.06084502999478938468)13:0.10587974457989959842,Bacteria-
Nitrosospira_multiformis_82702354:0.39585913429964292298)19:0.07782732605724190789)23:0.15879286063351383262)58:0.07886580565528274189,((((Bacteria-Pelotomaculum_thermopropionicum_147676360:0.29705861469541972042,Bacteria-
Desulfotomaculum_reducens_134297898:0.33327200800141354442)68:0.12849143095139164972,Bacteria-

Carboxydothermus_hydrogenoformans_78043781:0.31939091971337030262)30:0.13208833670724398623,(((Cyanobacteria-
Synechococcus_elongatus_PCC_7942_81300665:0.00000121823947223981,Cyanobacteria-
Synechococcus_elongatus_PCC_6301_56752247:0.00706443503013132884)100:0.35571444532795049032,(Cyanobacteria-
Gloeobacter_violaceus_PCC_7421_37520622:0.36767049470424906232,Cyanobacteria-
Nostoc_punctiforme_PCC_73102_23125821:0.42858588242258410794)58:0.04979753457394767852)100:0.46295655661169249351,(Bacteria-Moorella_thermoacetica_83588899:0.41018470524233818786,(Bacteria-
Syntrophomonas_wolfei_114565589:0.49957778398091606809,Bacteria-
Desulfitobacterium_hafniense_89892755:0.34346901928164053075)35:0.14226095377430372779)0:0.06556166191185873460
)0:0.00000121823947223981)12:0.08182143935842264848,((Bacteria-
Anaeromyxobacter_sp._153006601:0.38055921959856842118,(Bacteria-
Myxococcus_xanthus_108758559:0.12174940263156551479,Bacteria-
Stigmatella_aurantiaca_115378509:0.12559721540475332624)100:0.27354989132802309815)48:0.12269811195924791580,Ba
cteria-
Collinsella_aerofaciens_139439263:0.53613683262220257486)33:0.14674022147181875475)2:0.05687275598083507688)2:0.0
3552598730631863905,(((Bacteria-Thermobifida_fusca_72160431:0.28996120406971953587,Bacteria-
Streptomyces_avermitilis_29830723:0.11728920429337015563)97:0.25331831055003795061,Bacteria-
Rubrobacter_xylanophilus_108805662:0.55879734020894178403)44:0.13528317526556449257,((Bacteria-
Symbiobacterium_thermophilum_51891149:0.28819792511792602374,((Bacteria-
Flavobacteria_bacterium_126662495:0.14611535840769379613,Bacteria-
Flavobacterium_johnsoniae_146299285:0.10165454553281765004)85:0.14017116105718843699,Bacteria-
unidentified_eubacterium_149369606:0.11813082138609908023)100:0.71320752952563748472)23:0.08373031768267748254,
(((Bacteria-Staphylococcus_haemolyticus_70727433:0.38277154854919137605,(((Bacteria-
Lactobacillus_casei_116495716:0.43601170990784315906,(Bacteria-
Chlorobium_chlorochromatii_78188173:0.61205018231354157976,Bacteria-
Thermoanaerobacter_ethanolicus_114845498:0.27862963120402411965)41:0.09479797654943034058)50:0.18442921453851976454,(Bacteria-Alkaliphilus_metalliredigens_150387879:0.25439789481627000933,Bacteria-
Clostridium_sp._106895303:0.23015467999780719888)65:0.07732990460272902433)11:0.05863337307841201551,Bacteria-
Ruminococcus_torques_153815558:0.53232378078100883734)13:0.11730582955315314864)6:0.04319744551493163998,Bact
eria-Bacillus_sp._126652937:0.34091461897315639140)7:0.06495111164459893560,((Bacteria-
Pelobacter_carbinolicus_77917942:0.30456517337971061865,Bacteria-
Desulfuromonas_acetoxidans_95928326:0.20103950185442304166)97:0.13256477864988933102,Bacteria-
Geobacter_lovleyi_118746176:0.52242038792766798405)58:0.11084057572643503387)3:0.08277298270934640989)12:0.0678
9536538991432224)2:0.00000121823947223981)81:0.14644052271950500077)64:0.12180266468731583418,((Plantae-
Ostreococcus_tauri_17006:0.23105338938422689821,Plantae-
Ostreococcus_lucimarinus_37349:0.25135485056432443907)100:0.46519386217121155846,(Plantae-
Volvox_carteri_89616_jgi:0.89319994948684955371,Plantae-
Chlamydomonas_reinhardtii_74200:0.34135246875700619995)94:0.18500592890819073100)56:0.10109568330783454126)39:
0.10137131654080058785)76:0.16810628361934396025,Chlamydia-
Candidatus_Protochlamydia_46446282:0.50713941511201832135)100:0.33860341104082203323)98:0.10775939973411481632,Chlamydia-Chlamydia_muridarum_15834852:0.07072250252742939836);

Plantae-Volvox_carteri_99233_jgi

(((((((((Chromalveolata-Phytophthora_sojae_136539:2.47734357921934300606,Plantae-
Volvox_carteri_99233_jgi:1.72299151547716467014)20:0.18486231265585403860,((Plantae-
Arabidopsis_thaliana_30684506:0.27520078400377101513,((Plantae-
Oryza_sativa_115447277:0.27608974889448451018,Plantae-
Oryza_sativa_115459280:0.25154504452865156239)77:0.21263813651618854172,(Plantae-
Physcomitrella_patens_60819:0.24674421954783379296,Plantae-
Physcomitrella_patens_206734:0.26877166202868313372)100:0.72506826998508355775)51:0.17949581629111693859)100:1.
20703351491705745957,((Chromalveolata-Thalassiosira_pseudonana_16210:0.60356586115904287126,Chromalveolata-
Phaeodactylum_tricornutum_1199:0.66116577238989449050)100:0.89069287896144944128,Plantae-
Physcomitrella_patens_186728:3.38768663026300531627)24:0.30126778695810924047)34:0.33424091766775598789)40:0.37
042687802281937248,(Opisthokonta-Mus_musculus_19527158:0.10581277519521044284,Opisthokonta-
Danio_rerio_41393121:0.10643345648172930495)100:2.25510436999960850457)82:0.69231184777432774879,Chlamydia-
Candidatus_Protochlamydia_46446522:0.19689750236972877739)13:0.02287276113630387631,(((((Chlamydia-
Candidatus_Protochlamydia_46446910:0.13506234065540967393,(Chlamydia-
Candidatus_Protochlamydia_46446916:0.18165382665769688741,Chlamydia-
Candidatus_Protochlamydia_46446912:0.15357730047067200130)77:0.06530681735749058192)100:0.17044494493400971780,(Chlamydia-Candidatus_Protochlamydia_46447199:0.64701412675337721669,Chlamydia-
Candidatus_Protochlamydia_46447653:0.33830803412825444232)35:0.04554395991943925581)38:0.05691045963658872969,
((((Chlamydia-Candidatus_Protochlamydia_46445968:0.45904835089005519011,(Chlamydia-
Candidatus_Protochlamydia_46447197:0.26106611295816600782,Chlamydia-

Candidatus_Protochlamydia_46445970:0.15804201117533997478)90:0.05439214628794332429)57:0.05183492450049025785,
Chlamydia-Candidatus_Protochlamydia_46445969:0.32418430663791752977)39:0.03476718818221369994,(((Chlamydia-
Candidatus_Protochlamydia_46446906:0.33022791596401546155,Chlamydia-
Candidatus_Protochlamydia_46446377:0.35364726831957910003)23:0.01982119671265862235,(Chlamydia-
Candidatus_Protochlamydia_46447144:0.31088149586148106085,Chlamydia-
Candidatus_Protochlamydia_46447141:0.19814715071798688628)97:0.12959680665018613599)85:0.07324346317715040022,
(Chlamydia-Candidatus_Protochlamydia_46447283:0.23438279341823270441,Chlamydia-
Candidatus_Protochlamydia_46445682:0.35423883944037098948)100:0.30039751111057100275)23:0.016709622781102970
86)11:0.02166801831017479280,((Chlamydia-Candidatus_Protochlamydia_46447096:0.07920600297229504760,Chlamydia-
Candidatus_Protochlamydia_46447250:0.04938792991713483738)100:0.50126141587757178097,Chlamydia-
Candidatus_Protochlamydia_46447142:0.46163667205396624604)50:0.05086296444039087317)32:0.04784643931005619355)
81:0.09290463035958870797,(Chlamydia-Candidatus_Protochlamydia_46447626:0.30724041708315930066,Chlamydia-
Candidatus_Protochlamydia_46447300:0.21484415046800092730)78:0.07284836138984859821)24:0.02932660648320882871,
(Chlamydia-Candidatus_Protochlamydia_46447466:0.32261666050304388387,(Chlamydia-
Candidatus_Protochlamydia_46445833:0.41178508612405079203,Chlamydia-
Candidatus_Protochlamydia_46445832:0.12908878079789343207)77:0.05780511454100423219)49:0.05235245792258558239)
63:0.05553301231000767196)18:0.06635729147638902425,Chlamydia-
Candidatus_Protochlamydia_46446666:0.26784879167419584078)3:0.02704564776712329449,Chlamydia-
Candidatus_Protochlamydia_46447549:0.24465357477544452669)8:0.03822534342413851788,(Chlamydia-
Candidatus_Protochlamydia_46446831:0.25370152777808790967,Chlamydia-
Candidatus_Protochlamydia_46447588:0.33946947660608595143)15:0.02019233720088338055)3:0.02303880333052050686,(((
(Plantae-Chlamydomonas_reinhardtii_153692:4.30049106887062393412,Chlamydia-
Candidatus_Protochlamydia_46446667:0.26085807904368768506)15:0.05116647563619850653,(Chlamydia-
Candidatus_Protochlamydia_46446665:0.27202981367340145491,(Chlamydia-
Candidatus_Protochlamydia_46447554:0.24117207972327328602,Chlamydia-
Candidatus_Protochlamydia_46446659:0.25323769053458211609)41:0.03126720011166277124)15:0.03313787506069293398)
1:0.03459364223657935417,(((Chlamydia-Candidatus_Protochlamydia_46447548:0.20307020502974928999,Chlamydia-
Candidatus_Protochlamydia_46446776:0.17455482324273871586)92:0.08676045925611673704,((Chlamydia-
Candidatus_Protochlamydia_46447589:0.27449372145409595136,(Chlamydia-
Candidatus_Protochlamydia_46446626:0.20582206884481382692,(Chlamydia-
Candidatus_Protochlamydia_46447552:0.28786227814473519526,Chlamydia-
Candidatus_Protochlamydia_46447562:0.27996507480153459690)33:0.04980004664011779270)19:0.02558840729476399498)
13:0.02863445087802663283,((Chlamydia-Candidatus_Protochlamydia_46447550:0.34407108785901002745,Chlamydia-
Candidatus_Protochlamydia_46447560:0.22673053511522997527)73:0.05942003061114255885,(Chlamydia-
Candidatus_Protochlamydia_46446627:0.50002834539289853222,Chlamydia-
Candidatus_Protochlamydia_46447569:0.31342511418916463306)18:0.04612390141665463628)19:0.04774625364942158834)
1:0.03401050750737850958)9:0.01244367603769516196,(Chlamydia-
Candidatus_Protochlamydia_46447568:0.36802829580268520138,Chlamydia-
Candidatus_Protochlamydia_46447590:0.32498470248237132374)16:0.03779034844894482714)7:0.03181303577587088482)0
:0.01063523007309752182,Chlamydia-Candidatus_Protochlamydia_46447129:0.27538478965770235574);
