# Supplementary material for: Chlamydiae Has Contributed at Least 55 Genes to Plantae with Predominantly Plastid Functions
Source: PLoS One. 2008 May 21;3(5):e2205. doi: 10.1371/journal.pone.0002205 (PMC2376095; doi:10.1371/journal.pone.0002205)
Supplement: Table S3 — Complete set of taxa present in our local genome database that was used to search for chlamydial genes in Plantae. (0.24 MB PDF) [file pone.0002205.s003.pdf]

Table S3. Complete set of taxa present in our local genome database that was used to search for chlamydial genes in Plantae.

1	1	Amoebozoa-Acanthamoeba castellanii
2	2	Amoebozoa-Dictyostelium discoideum
3	3	Amoebozoa-Entamoeba histolytica
4	4	Amoebozoa-Hartmannella vermiformis
5	1	Chromalveolata-Alexandrium tamarense
6	2	Chromalveolata-Aureococcus anophagefferens
7	3	Chromalveolata-Emiliania huxleyi
8	4	Chromalveolata-Guillardia theta
9	5	Chromalveolata-Isochrysis galbana
10	6	Chromalveolata-Karenia brevis
11	7	Chromalveolata-Laminaria digitata
12	8	Chromalveolata-Paramecium tetraurelia
13	9	Chromalveolata-Pavlova lutheri
14	10	Chromalveolata-Phaeodactylum tricornutum
15	11	Chromalveolata-Phytophthora sojae
16	12	Chromalveolata-Plasmodium falciparum
17	13	Chromalveolata-Tetrahymena thermophila
18	14	Chromalveolata-Thalassiosira pseudonana
19	15	Chromalveolata-Toxoplasma gondii
20	1	Excavata-Euglena gracilis
21	2	Excavata-Giardia lamblia
22	3	Excavata-Jakoba bahamiensis
23	4	Excavata-Jakoba libera
24	5	Excavata-Leishmania major
25	6	Excavata-Malawimonas californiana
26	7	Excavata-Malawimonas jakobiformis
27	8	Excavata-Naegleria gruberi
28	9	Excavata-Trichomonas vaginalis
29	10	Excavata-Trypanosoma brucei
30	11	Excavata-Trypanosoma cruzi
31	1	Opisthokonta-Apis mellifera
32	2	Opisthokonta-Cryptococcus neoformans
33	3	Opisthokonta-Danio rerio
34	4	Opisthokonta-Drosophila melanogaster
35	5	Opisthokonta-Magnaporthe grisea
36	6	Opisthokonta-Mus musculus
37	7	Opisthokonta-Saccharomyces cerevisiae
38	8	Opisthokonta-Schizosaccharomyces pombe
39	9	Opisthokonta-Sporobolomyces roseus
40	10	Opisthokonta-Ustilago maydis
41	1	Plantae-Arabidopsis thaliana
42	2	Plantae-Chlamydomonas reinhardtii
43	3	Plantae-Chondrus crispus
44	4	Plantae-Cyanidioschyzon merolae
45	5	Plantae-Cyanophora paradoxa
46	6	Plantae-Galdieria sulphuraria
47	7	Plantae-Glaucocystis nostochinearum

48	8	Plantae-Gracilaria changii
49	9	Plantae-Oryza sativa
50	10	Plantae-Ostreococcus lucimarinus
51	11	Plantae-Ostreococcus tauri
52	12	Plantae-Physcomitrella patens
53	13	Plantae-Porphyra yezoensis
54	14	Plantae-Volvox carteri
55	1	Rhizaria-Bigelowiella natans
56	2	Rhizaria-Reticulomyxa filosa
57	1	Chlamydia-Candidatus Protochlamydia
58	2	Chlamydia-Chlamydia muridarum
59	3	Chlamydia-Chlamydia trachomatis
60	4	Chlamydia-Chlamydophila abortus
61	5	Chlamydia-Chlamydophila caviae
62	6	Chlamydia-Chlamydophila felis
63	7	Chlamydia-Chlamydophila pneumoniae
64	8	Chlamydia-Chlamydophila psittaci
65	1	Cyanobacteria-Anabaena variabilis
66	2	Cyanobacteria-Crocosphaera watsonii
67	3	Cyanobacteria-Cyanothece sp.
68	4	Cyanobacteria-Gloeobacter violaceus
69	5	Cyanobacteria-Leptolyngbya foveolarum
70	6	Cyanobacteria-Leptolyngbya sp.
71	7	Cyanobacteria-Leptolyngbya valderiana
72	8	Cyanobacteria-Lyngbya sp.
73	9	Cyanobacteria-Microcystis aeruginosa
74	10	Cyanobacteria-Nodularia spumigena
75	11	Cyanobacteria-Nostoc punctiforme
76	12	Cyanobacteria-Nostoc sp.
77	13	Cyanobacteria-Prochlorococcus marinus
78	14	Cyanobacteria-Synechococcus elongatus
79	15	Cyanobacteria-Synechococcus sp.
80	16	Cyanobacteria-Synechocystis sp.
81	17	Cyanobacteria-Thermosynechococcus elongatus
82	18	Cyanobacteria-Trichodesmium erythraeum
83	1	Bacteria-Acetobacter aceti
84	2	Bacteria-Achromobacter xylosoxidans
85	3	Bacteria-Acidiphilium cryptum
86	4	Bacteria-Acidiphilium multivorum
87	5	Bacteria-Acidithiobacillus caldus
88	6	Bacteria-Acidithiobacillus ferrooxidans
89	7	Bacteria-Acidobacteria bacterium
90	8	Bacteria-Acidothermus cellulolyticus
91	9	Bacteria-Acidovorax avenae
92	10	Bacteria-Acidovorax sp.
93	11	Bacteria-Acinetobacter baumannii
94	12	Bacteria-Actinobacillus actinomycetemcomitans

95	13	Bacteria-Actinobacillus pleuropneumoniae
96	14	Bacteria-Actinobacillus porcitonsillarum
97	15	Bacteria-Actinobacillus succinogenes
98	16	Bacteria-Actinomyces odontolyticus
99	17	Bacteria-Aeromonas bestiarum
100	18	Bacteria-Aeromonas hydrophila
101	19	Bacteria-Aeromonas punctata
102	20	Bacteria-Aeromonas salmonicida
103	21	Bacteria-Agrobacterium tumefaciens
104	22	Bacteria-Alcanivorax borkumensis
105	23	Bacteria-Algoriphagus sp.
106	24	Bacteria-Alkalilimnicola ehrlichei
107	25	Bacteria-Alkaliphilus metalliredigens
108	26	Bacteria-alpha proteobacterium
109	27	Bacteria-Alteromonadales bacterium
110	28	Bacteria-Anaeromyxobacter dehalogenans
111	29	Bacteria-Anaeromyxobacter sp.
112	30	Bacteria-Anaplasma marginale
113	31	Bacteria-Anaplasma phagocytophilum
114	32	Bacteria-Aquifex aeolicus
115	33	Bacteria-Arcanobacterium pyogenes
116	34	Bacteria-Arcobacter butzleri
117	35	Bacteria-Arthrobacter aurescens
118	36	Bacteria-Arthrobacter nitroguajacolicus
119	37	Bacteria-Arthrobacter sp.
120	38	Bacteria-Aster yellows
121	39	Bacteria-Aurantimonas sp.
122	40	Bacteria-Avibacterium paragallinarum
123	41	Bacteria-Azoarcus sp.
124	42	Bacteria-Bacillus amyloliquefaciens
125	43	Bacteria-Bacillus anthracis
126	44	Bacteria-Bacillus cereus
127	45	Bacteria-Bacillus coagulans
128	46	Bacteria-Bacillus licheniformis
129	47	Bacteria-Bacillus megaterium
130	48	Bacteria-Bacillus methanolicus
131	49	Bacteria-Bacillus mycoides
132	50	Bacteria-Bacillus pumilus
133	51	Bacteria-Bacillus sp.
134	52	Bacteria-Bacillus subtilis
135	53	Bacteria-Bacillus thuringiensis
136	54	Bacteria-Bacillus weihenstephanensis
137	55	Bacteria-Bacteroides caccae
138	56	Bacteria-Bacteroides capillosus
139	57	Bacteria-Bacteroides fragilis
140	58	Bacteria-Bacteroides thetaiotaomicron
141	59	Bacteria-Bacteroides vulgatus

142	60	Bacteria-Bartonella bacilliformis
143	61	Bacteria-Bartonella grahamii
144	62	Bacteria-Baumannia cicadellinicola
145	63	Bacteria-Bdellovibrio bacteriovorus
146	64	Bacteria-Beet leafhopper
147	65	Bacteria-Beggiatoa sp.
148	66	Bacteria-Bifidobacterium adolescentis
149	67	Bacteria-Bifidobacterium breve
150	68	Bacteria-Bifidobacterium longum
151	69	Bacteria-Bifidobacterium pseudocatenulatum
152	70	Bacteria-Bordetella bronchiseptica
153	71	Bacteria-Bordetella pertussis
154	72	Bacteria-Borrelia afzelii
155	73	Bacteria-Borrelia burgdorferi
156	74	Bacteria-Borrelia duttonii
157	75	Bacteria-Borrelia garinii
158	76	Bacteria-Bradyrhizobium japonicum
159	77	Bacteria-Bradyrhizobium sp.
160	78	Bacteria-Brucella ovis
161	79	Bacteria-Buchnera aphidicola
162	80	Bacteria-Burkholderia ambifaria
163	81	Bacteria-Burkholderia cenocepacia
164	82	Bacteria-Burkholderia cepacia
165	83	Bacteria-Burkholderia mallei
166	84	Bacteria-Burkholderia multivorans
167	85	Bacteria-Burkholderia phymatum
168	86	Bacteria-Burkholderia phytofirmans
169	87	Bacteria-Burkholderia pseudomallei
170	88	Bacteria-Burkholderia sp.
171	89	Bacteria-Burkholderia vietnamiensis
172	90	Bacteria-Burkholderia xenovorans
173	91	Bacteria-Butyrivibrio fibrisolvens
174	92	Bacteria-Caldicellulosiruptor saccharolyticus
175	93	Bacteria-Caminibacter mediatlanticus
176	94	Bacteria-Campylobacter coli
177	95	Bacteria-Campylobacter concisus
178	96	Bacteria-Campylobacter curvus
179	97	Bacteria-Campylobacter fetus
180	98	Bacteria-Campylobacter hominis
181	99	Bacteria-Campylobacter jejuni
182	100	Bacteria-Campylobacter lari
183	101	Bacteria-candidate division
184	102	Bacteria-Candidatus Blochmannia
185	103	Bacteria-Candidatus Carsonella
186	104	Bacteria-Candidatus Desulfococcus
187	105	Bacteria-Candidatus Pelagibacter
188	106	Bacteria-Candidatus Ruthia

189	107	Bacteria-Candidatus Sulcia
190	108	Bacteria-Candidatus Vesicomyosocius
191	109	Bacteria-Carboxydothermus hydrogenoformans
192	110	Bacteria-Caulobacter crescentus
193	111	Bacteria-Caulobacter sp.
194	112	Bacteria-Chlorobium chlorochromatii
195	113	Bacteria-Chlorobium ferrooxidans
196	114	Bacteria-Chlorobium limicola
197	115	Bacteria-Chlorobium phaeobacteroides
198	116	Bacteria-Chloroflexus aggregans
199	117	Bacteria-Chromobacterium violaceum
200	118	Bacteria-Chromohalobacter salexigens
201	119	Bacteria-Citrobacter koseri
202	120	Bacteria-Clavibacter michiganensis
203	121	Bacteria-Clostridium acetobutylicum
204	122	Bacteria-Clostridium beijerinckii
205	123	Bacteria-Clostridium botulinum
206	124	Bacteria-Clostridium cellulolyticum
207	125	Bacteria-Clostridium difficile
208	126	Bacteria-Clostridium kluyveri
209	127	Bacteria-Clostridium novyi
210	128	Bacteria-Clostridium perfringens
211	129	Bacteria-Clostridium phytofermentans
212	130	Bacteria-Clostridium sp.
213	131	Bacteria-Clostridium tetani
214	132	Bacteria-Clostridium thermocellum
215	133	Bacteria-Collinsella aerofaciens
216	134	Bacteria-Comamonas testosteroni
217	135	Bacteria-Corynebacterium diphtheriae
218	136	Bacteria-Corynebacterium glutamicum
219	137	Bacteria-Corynebacterium jeikeium
220	138	Bacteria-Corynebacterium renale
221	139	Bacteria-Corynebacterium sp.
222	140	Bacteria-Corynebacterium striatum
223	141	Bacteria-Coxiella burnetii
224	142	Bacteria-Cytophaga hutchinsonii
225	143	Bacteria-Dechloromonas aromatica
226	144	Bacteria-Dehalococcoides ethenogenes
227	145	Bacteria-Dehalococcoides sp.
228	146	Bacteria-Deinococcus geothermalis
229	147	Bacteria-Deinococcus radiodurans
230	148	Bacteria-Delftia acidovorans
231	149	Bacteria-delta proteobacterium
232	150	Bacteria-Desulfitobacterium hafniense
233	151	Bacteria-Desulfotalea psychrophila
234	152	Bacteria-Desulfotomaculum reducens
235	153	Bacteria-Desulfovibrio desulfuricans

236	154	Bacteria-Desulfovibrio vulgaris
237	155	Bacteria-Desulfuromonas acetoxidans
238	156	Bacteria-Dichelobacter nodosus
239	157	Bacteria-Dinoroseobacter shibae
240	158	Bacteria-Dorea longicatena
241	159	Bacteria-Edwardsiella ictaluri
242	160	Bacteria-Ehrlichia canis
243	161	Bacteria-Ehrlichia chaffeensis
244	162	Bacteria-Ehrlichia ruminantium
245	163	Bacteria-Enterobacter aerogenes
246	164	Bacteria-Enterobacteria phage
247	165	Bacteria-Enterobacter sakazakii
248	166	Bacteria-Enterobacter sp.
249	167	Bacteria-Enterococcus faecalis
250	168	Bacteria-Enterococcus faecium
251	169	Bacteria-Erwinia sp.
252	170	Bacteria-Erysipelothrix rhusiopathiae
253	171	Bacteria-Erythrobacter litoralis
254	172	Bacteria-Erythrobacter sp.
255	173	Bacteria-Escherichia coli
256	174	Bacteria-Escherichia sp.
257	175	Bacteria-Eubacterium ventriosum
258	176	Bacteria-Fervidobacterium nodosum
259	177	Bacteria-Flavobacteria bacterium
260	178	Bacteria-Flavobacterium johnsoniae
261	179	Bacteria-Flavobacterium psychrophilum
262	180	Bacteria-Francisella tularensis
263	181	Bacteria-Frankia alni
264	182	Bacteria-Frankia sp.
265	183	Bacteria-Fulvimarina pelagi
266	184	Bacteria-Fusobacterium nucleatum
267	185	Bacteria-Geobacillus thermodenitrificans
268	186	Bacteria-Geobacter bemidjiensis
269	187	Bacteria-Geobacter lovleyi
270	188	Bacteria-Geobacter metallireducens
271	189	Bacteria-Geobacter sp.
272	190	Bacteria-Geobacter uraniumreducens
273	191	Bacteria-Gluconobacter oxydans
274	192	Bacteria-Gramella forsetii
275	193	Bacteria-Granulibacter bethesdensis
276	194	Bacteria-Haemophilus ducreyi
277	195	Bacteria-Haemophilus influenzae
278	196	Bacteria-Haemophilus parasuis
279	197	Bacteria-Haemophilus somnus
280	198	Bacteria-Hafnia alvei
281	199	Bacteria-Hahella chejuensis
282	200	Bacteria-Halorhodospira halophila

283	201	Bacteria-Halothermothrix orenii
284	202	Bacteria-Helicobacter acinonychis
285	203	Bacteria-Helicobacter pylori
286	204	Bacteria-Herminiimonas arsenicoxydans
287	205	Bacteria-Herpetosiphon aurantiacus
288	206	Bacteria-Histophilus somni
289	207	Bacteria-Hydrogenobaculum sp.
290	208	Bacteria-Hyphomonas neptunium
291	209	Bacteria-Jannaschia sp.
292	210	Bacteria-Janthinobacterium sp.
293	211	Bacteria-Kineococcus radiotolerans
294	212	Bacteria-Klebsiella pneumoniae
295	213	Bacteria-Lactobacillus acidophilus
296	214	Bacteria-Lactobacillus brevis
297	215	Bacteria-Lactobacillus casei
298	216	Bacteria-Lactobacillus delbrueckii
299	217	Bacteria-Lactobacillus fermentum
300	218	Bacteria-Lactobacillus gasseri
301	219	Bacteria-Lactobacillus johnsonii
302	220	Bacteria-Lactobacillus plantarum
303	221	Bacteria-Lactobacillus reuteri
304	222	Bacteria-Lactobacillus sakei
305	223	Bacteria-Lactobacillus salivarius
306	224	Bacteria-Lactococcus lactis
307	225	Bacteria-Lawsonia intracellularis
308	226	Bacteria-Legionella pneumophila
309	227	Bacteria-Leifsonia xyli
310	228	Bacteria-Leptospira borgpetersenii
311	229	Bacteria-Leptospira interrogans
312	230	Bacteria-Leptospirillum ferrooxidans
313	231	Bacteria-Leuconostoc citreum
314	232	Bacteria-Leuconostoc mesenteroides
315	233	Bacteria-Limnobacter sp.
316	234	Bacteria-Listeria monocytogenes
317	235	Bacteria-Listeria welshimeri
318	236	Bacteria-Listonella anguillarum
319	237	Bacteria-Magnetococcus sp.
320	238	Bacteria-Magnetospirillum gryphiswaldense
321	239	Bacteria-Magnetospirillum magneticum
322	240	Bacteria-Maricaulis maris
323	241	Bacteria-marine actinobacterium
324	242	Bacteria-marine gamma
325	243	Bacteria-Marinobacter algicola
326	244	Bacteria-Marinobacter aquaeolei
327	245	Bacteria-Marinobacter sp.
328	246	Bacteria-Marinococcus halophilus
329	247	Bacteria-Marinomonas sp.

330	248	Bacteria-Mariprofundus ferrooxydans
331	249	Bacteria-Mesorhizobium loti
332	250	Bacteria-Mesorhizobium sp.
333	251	Bacteria-Methylibium petroleiphilum
334	252	Bacteria-Methylobacillus flagellatus
335	253	Bacteria-Methylobacterium chloromethanicum
336	254	Bacteria-Methylobacterium extorquens
337	255	Bacteria-Methylobacterium sp.
338	256	Bacteria-Methylococcus capsulatus
339	257	Bacteria-Methylophilales bacterium
340	258	Bacteria-Micromonospora rosaria
341	259	Bacteria-Microscilla marina
342	260	Bacteria-Moorella thermoacetica
343	261	Bacteria-Moraxella sp.
344	262	Bacteria-Moritella sp.
345	263	Bacteria-Mycobacterium avium
346	264	Bacteria-Mycobacterium bovis
347	265	Bacteria-Mycobacterium gilvum
348	266	Bacteria-Mycobacterium smegmatis
349	267	Bacteria-Mycobacterium sp.
350	268	Bacteria-Mycobacterium tuberculosis
351	269	Bacteria-Mycobacterium ulcerans
352	270	Bacteria-Mycobacterium vanbaalenii
353	271	Bacteria-Mycoplasma agalactiae
354	272	Bacteria-Mycoplasma capricolum
355	273	Bacteria-Mycoplasma genitalium
356	274	Bacteria-Mycoplasma mobile
357	275	Bacteria-Mycoplasma mycoides
358	276	Bacteria-Mycoplasma penetrans
359	277	Bacteria-Mycoplasma pulmonis
360	278	Bacteria-Mycoplasma sp.
361	279	Bacteria-Mycoplasma synoviae
362	280	Bacteria-Myxococcus xanthus
363	281	Bacteria-Neisseria meningitidis
364	282	Bacteria-Neorickettsia sennetsu
365	283	Bacteria-Nitratiruptor sp.
366	284	Bacteria-Nitrobacter hamburgensis
367	285	Bacteria-Nitrobacter winogradskyi
368	286	Bacteria-Nitrosococcus oceani
369	287	Bacteria-Nitrosomonas europaea
370	288	Bacteria-Nitrosomonas eutropha
371	289	Bacteria-Nitrosospira multiformis
372	290	Bacteria-Nocardioides sp.
373	291	Bacteria-Novosphingobium aromaticivorans
374	292	Bacteria-Oceanicola granulosus
375	293	Bacteria-Oceanobacter sp.
376	294	Bacteria-Oceanospirillum sp.

377	295	Bacteria-Ochrobactrum anthropi
378	296	Bacteria-Oenococcus oeni
379	297	Bacteria-Orientia tsutsugamushi
380	298	Bacteria-Pantoea citrea
381	299	Bacteria-Parabacteroides distasonis
382	300	Bacteria-Parabacteroides merdae
383	301	Bacteria-Paracoccus denitrificans
384	302	Bacteria-Paracoccus methylutens
385	303	Bacteria-Paracoccus pantotrophus
386	304	Bacteria-Parvibaculum lavamentivorans
387	305	Bacteria-Pasteurella multocida
388	306	Bacteria-Pasteurella trehalosi
389	307	Bacteria-Pediococcus acidilactici
390	308	Bacteria-Pediococcus pentosaceus
391	309	Bacteria-Pedobacter sp.
392	310	Bacteria-Pelobacter carbinolicus
393	311	Bacteria-Pelobacter propionicus
394	312	Bacteria-Pelodictyon luteolum
395	313	Bacteria-Pelotomaculum thermopropionicum
396	314	Bacteria-Petrotoga mobilis
397	315	Bacteria-Photobacterium damselae
398	316	Bacteria-Photobacterium profundum
399	317	Bacteria-Photobacterium sp.
400	318	Bacteria-Plesiocystis pacifica
401	319	Bacteria-Polaromonas naphthalenivorans
402	320	Bacteria-Polaromonas sp.
403	321	Bacteria-Polynucleobacter sp.
404	322	Bacteria-Propionibacterium acidipropionici
405	323	Bacteria-Propionibacterium acnes
406	324	Bacteria-Prosthecochloris vibrioformis
407	325	Bacteria-Proteus vulgaris
408	326	Bacteria-Pseudoalteromonas atlantica
409	327	Bacteria-Pseudoalteromonas sp.
410	328	Bacteria-Pseudoalteromonas tunicata
411	329	Bacteria-Pseudomonas aeruginosa
412	330	Bacteria-Pseudomonas alcaligenes
413	331	Bacteria-Pseudomonas entomophila
414	332	Bacteria-Pseudomonas fluorescens
415	333	Bacteria-Pseudomonas fulva
416	334	Bacteria-Pseudomonas mendocina
417	335	Bacteria-Pseudomonas putida
418	336	Bacteria-Pseudomonas resinovorans
419	337	Bacteria-Pseudomonas sp.
420	338	Bacteria-Pseudomonas stutzeri
421	339	Bacteria-Pseudomonas syringae
422	340	Bacteria-Psychrobacter cryohalolentis
423	341	Bacteria-Psychrobacter sp.

424	342	Bacteria-Psychroflexus torquis
425	343	Bacteria-Psychromonas ingrahamii
426	344	Bacteria-Psychromonas sp.
427	345	Bacteria-Rahnella sp.
428	346	Bacteria-Ralstonia eutropha
429	347	Bacteria-Ralstonia metallidurans
430	348	Bacteria-Ralstonia pickettii
431	349	Bacteria-Ralstonia solanacearum
432	350	Bacteria-Rhizobium etli
433	351	Bacteria-Rhizobium leguminosarum
434	352	Bacteria-Rhizobium sp.
435	353	Bacteria-Rhodobacterales bacterium
436	354	Bacteria-Rhodobacter blasticus
437	355	Bacteria-Rhodobacter sphaeroides
438	356	Bacteria-Rhodococcus erythropolis
439	357	Bacteria-Rhodococcus opacus
440	358	Bacteria-Rhodococcus rhodochrous
441	359	Bacteria-Rhodococcus sp.
442	360	Bacteria-Rhodoferax ferrireducens
443	361	Bacteria-Rhodopseudomonas palustris
444	362	Bacteria-Rhodospirillum rubrum
445	363	Bacteria-Rhodothermus marinus
446	364	Bacteria-Rickettsia akari
447	365	Bacteria-Rickettsia bellii
448	366	Bacteria-Rickettsia canadensis
449	367	Bacteria-Rickettsia conorii
450	368	Bacteria-Rickettsia felis
451	369	Bacteria-Rickettsia massiliae
452	370	Bacteria-Rickettsia rickettsii
453	371	Bacteria-Rickettsia typhi
454	372	Bacteria-Rickettsiella grylli
455	373	Bacteria-Roseiflexus castenholzii
456	374	Bacteria-Roseiflexus sp.
457	375	Bacteria-Roseobacter denitrificans
458	376	Bacteria-Roseobacter sp.
459	377	Bacteria-Roseovarius sp.
460	378	Bacteria-Rubrobacter xylanophilus
461	379	Bacteria-Ruegeria sp.
462	380	Bacteria-Ruminococcus flavefaciens
463	381	Bacteria-Ruminococcus gnavus
464	382	Bacteria-Ruminococcus obeum
465	383	Bacteria-Ruminococcus torques
466	384	Bacteria-Saccharophagus degradans
467	385	Bacteria-Saccharopolyspora erythraea
468	386	Bacteria-Sagittula stellata
469	387	Bacteria-Salinibacter ruber
470	388	Bacteria-Salinispora arenicola

471	389	Bacteria-Salinispora tropica
472	390	Bacteria-Salmonella choleraesuis
473	391	Bacteria-Salmonella enterica
474	392	Bacteria-Salmonella enteritidis
475	393	Bacteria-Salmonella phage
476	394	Bacteria-Salmonella typhi
477	395	Bacteria-Salmonella typhimurium
478	396	Bacteria-Selenomonas ruminantium
479	397	Bacteria-Serratia entomophila
480	398	Bacteria-Serratia marcescens
481	399	Bacteria-Serratia proteamaculans
482	400	Bacteria-Shewanella amazonensis
483	401	Bacteria-Shewanella baltica
484	402	Bacteria-Shewanella denitrificans
485	403	Bacteria-Shewanella frigidimarina
486	404	Bacteria-Shewanella loihica
487	405	Bacteria-Shewanella oneidensis
488	406	Bacteria-Shewanella pealeana
489	407	Bacteria-Shewanella putrefaciens
490	408	Bacteria-Shewanella sediminis
491	409	Bacteria-Shewanella sp.
492	410	Bacteria-Shewanella woodyi
493	411	Bacteria-Shigella boydii
494	412	Bacteria-Shigella dysenteriae
495	413	Bacteria-Shigella flexneri
496	414	Bacteria-Shigella sonnei
497	415	Bacteria-Silicibacter sp.
498	416	Bacteria-Sinorhizobium medicae
499	417	Bacteria-Sodalis glossinidius
500	418	Bacteria-Solibacter usitatus
501	419	Bacteria-Sphingobium yanoikuyae
502	420	Bacteria-Sphingomonas sp.
503	421	Bacteria-Sphingomonas wittichii
504	422	Bacteria-Sphingopyxis alaskensis
505	423	Bacteria-Spiroplasma citri
506	424	Bacteria-Spiroplasma kunkelii
507	425	Bacteria-Staphylococcus aureus
508	426	Bacteria-Staphylococcus chromogenes
509	427	Bacteria-Staphylococcus epidermidis
510	428	Bacteria-Staphylococcus haemolyticus
511	429	Bacteria-Staphylococcus lugdunensis
512	430	Bacteria-Staphylococcus saprophyticus
513	431	Bacteria-Staphylococcus simulans
514	432	Bacteria-Staphylococcus sp.
515	433	Bacteria-Staphylococcus warneri
516	434	Bacteria-Stappia aggregata
517	435	Bacteria-Stenotrophomonas maltophilia

518	436	Bacteria-Stigmatella aurantiaca
519	437	Bacteria-Streptococcus agalactiae
520	438	Bacteria-Streptococcus gordonii
521	439	Bacteria-Streptococcus mutans
522	440	Bacteria-Streptococcus phage
523	441	Bacteria-Streptococcus pneumoniae
524	442	Bacteria-Streptococcus pyogenes
525	443	Bacteria-Streptococcus sanguinis
526	444	Bacteria-Streptococcus suis
527	445	Bacteria-Streptococcus thermophilus
528	446	Bacteria-Streptomyces avermitilis
529	447	Bacteria-Streptomyces clavuligerus
530	448	Bacteria-Streptomyces coelicolor
531	449	Bacteria-Streptomyces ghanaensis
532	450	Bacteria-Streptomyces laurentii
533	451	Bacteria-Streptomyces natalensis
534	452	Bacteria-Streptomyces phaeochromogenes
535	453	Bacteria-Streptomyces rochei
536	454	Bacteria-Streptomyces sp.
537	455	Bacteria-Streptomyces venezuelae
538	456	Bacteria-Streptomyces violaceoruber
539	457	Bacteria-Sulfurovum sp.
540	458	Bacteria-Symbiobacterium thermophilum
541	459	Bacteria-Syntrophobacter fumaroxidans
542	460	Bacteria-Syntrophomonas wolfei
543	461	Bacteria-Syntrophus aciditrophicus
544	462	Bacteria-Thermoanaerobacter ethanolicus
545	463	Bacteria-Thermoanaerobacterium thermosaccharolyticum
546	464	Bacteria-Thermobifida fusca
547	465	Bacteria-Thermosinus carboxydivorans
548	466	Bacteria-Thermosipho melanesiensis
549	467	Bacteria-Thermotoga lettingae
550	468	Bacteria-Thermotoga petrophila
551	469	Bacteria-Thermus thermophilus
552	470	Bacteria-Thiomicrospira crunogena
553	471	Bacteria-Thiomicrospira denitrificans
554	472	Bacteria-Treponema denticola
555	473	Bacteria-Tropheryma whipplei
556	474	Bacteria-uncultured bacterium
557	475	Bacteria-unidentified eubacterium
558	476	Bacteria-Verminephrobacter eiseniae
559	477	Bacteria-Vibrio alginolyticus
560	478	Bacteria-Vibrio angustum
561	479	Bacteria-Vibrio cholerae
562	480	Bacteria-Vibrio fischeri
563	481	Bacteria-Vibrio harveyi
564	482	Bacteria-Vibrionales bacterium

565	483	Bacteria-Vibrio parahaemolyticus
566	484	Bacteria-Vibrio shilonii
567	485	Bacteria-Vibrio sp.
568	486	Bacteria-Vibrio vulnificus
569	487	Bacteria-Weissella cibaria
570	488	Bacteria-Wolbachia endosymbiont
571	489	Bacteria-Wolinella succinogenes
572	490	Bacteria-Xanthobacter autotrophicus
573	491	Bacteria-Xanthomonas campestris
574	492	Bacteria-Xanthomonas citri
575	493	Bacteria-Xanthomonas euvesicatoria
576	494	Bacteria-Xanthomonas oryzae
577	495	Bacteria-Xylella fastidiosa
578	496	Bacteria-Yersinia enterocolitica
579	497	Bacteria-Yersinia pestis
580	498	Bacteria-Yersinia pseudotuberculosis
581	499	Bacteria-Yersinia ruckeri
582	500	Bacteria-Zymomonas mobilis
